# Comparative genomics and transcriptome analysis of *Aspergillus niger* and metabolic engineering for citrate production

**DOI:** 10.1038/srep41040

**Published:** 2017-01-20

**Authors:** Xian Yin, Hyun-dong Shin, Jianghua Li, Guocheng Du, Long Liu, Jian Chen

**Affiliations:** 1 Key Laboratory of Industrial Biotechnology, Ministry of Education, Jiangnan University, Wuxi 214122, China; 2 Key Laboratory of Carbohydrate Chemistry and Biotechnology, Ministry of Education, Jiangnan University, Wuxi 214122, China; 3 School of Chemical and Biomolecular Engineering, Georgia Institute of Technology, Atlanta, GA 30332, USA

## Abstract

Despite a long and successful history of citrate production in *Aspergillus niger*, the molecular mechanism of citrate accumulation is only partially understood. In this study, we used comparative genomics and transcriptome analysis of citrate-producing strains—namely, *A. niger* H915-1 (citrate titer: 157 g L^−1^), A1 (117 g L^−1^), and L2 (76 g L^−1^)—to gain a genome-wide view of the mechanism of citrate accumulation. Compared with *A. niger* A1 and L2, *A. niger* H915-1 contained 92 mutated genes, including a succinate-semialdehyde dehydrogenase in the γ-aminobutyric acid shunt pathway and an aconitase family protein involved in citrate synthesis. Furthermore, transcriptome analysis of *A. niger* H915-1 revealed that the transcription levels of 479 genes changed between the cell growth stage (6 h) and the citrate synthesis stage (12 h, 24 h, 36 h, and 48 h). In the glycolysis pathway, triosephosphate isomerase was up-regulated, whereas pyruvate kinase was down-regulated. Two cytosol ATP-citrate lyases, which take part in the cycle of citrate synthesis, were up-regulated, and may coordinate with the alternative oxidases in the alternative respiratory pathway for energy balance. Finally, deletion of the oxaloacetate acetylhydrolase gene in H915-1 eliminated oxalate formation but neither influence on pH decrease nor difference in citrate production were observed.

*Aspergillus niger*, a black aspergillus with “generally regarded as safe” status, is widely used in the biotechnological production of organic acids and industrial enzymes^1^. As a workhorse of organic acids, *A. niger* is a proficient producer of citrate, which is used extensively in the food and pharmaceutical industries owing to its safety, pleasant acidic taste, high water solubility, and chelating properties^2^.

If *A. niger* is to be used as a cell factory platform, its genetic background must be thoroughly understood. The available genome sequence of *A. niger* offers a new horizon for both scientific studies and biotechnological applications^3^. *A. niger* strains NRRL 3 and ATCC 1015, which can synthesize citrate, have undergone genome-wide analysis^1^,^3^, and a complete genome-scale metabolic model with genomic annotation has been constructed based on the genome sequence of *A. niger* ATCC 1015^4^. Nevertheless, the molecular mechanism of citrate accumulation remains only partially understood^5^,^6^. For example, 5 citrate synthases have been identified in ATCC 9029^7^; however, the primary gene and the timing of the activity of each enzyme during fermentation remain a mystery. Furthermore, the citrate transporter remains unknown even though the transport process is critical in the production of citrate. In addition, the role of alternative oxidases for energy balance during citrate production must be clarified^8^.

In this study, the genomes of three *A. niger* strains with different citrate production efficiencies were sequenced, among which the genome of industrial strain *A. niger* H915-1 was sequenced with third-generation sequencing, which provides much longer reads and unbiased genome coverage compared with those of second-generation sequencing. Moreover, transcriptome analysis during citrate fermentation by *A. niger* H915-1 was conducted to explore how the regulation of enzyme expression facilitates citrate accumulation. Finally, the oxaloacetate acetylhydrolase gene (*oah*) was deleted in *A. niger* H915-1 to exam the role of oxalate on pH decrease and citrate production. The results of this study deepen the understanding of citrate accumulation by *A. niger* and provide potential engineering targets for further improvement in citrate production.

## Materials and Methods

### Strains

*A. niger* strains H915-1, L2, and A1 were provided by Jiangsu Guoxin Union Energy Co., Ltd (Yixing, Jiangsu Province, China), the third citrate producer in China. Strain H915-1 is an industrial producer, which was generated through several round of compound mutation, whereas strains L2 and A1 are the degenerated isolates of *A. niger* H915-1 during subsequent culture, as filamentous fungi frequently and spontaneously degenerate during maintenance in artificial media due to chromosome instability^9^. *Escherichia coli* JM109 was used as a host for recombinant DNA manipulation.

### Citrate fermentation by *A. niger* in 50-mL flasks

For citrate fermentation, conidia were inoculated in seed culture broth (a mixture of corn steep liquor and corn starch with a total sugar content of 10% and total nitrogen content of 0.2%). Eighty milliliters of seed culture was placed in 500-mL flasks and mixed at 250 rpm and 35 °C for 24 h. With 10% inoculum, the fermentation was performed in culture medium (a mixture of corn steep liquor and corn starch with a total sugar content of 16% and total nitrogen content of 0.08%) at 250 rpm and 35 °C until the reducing sugar was exhausted.

### Fed-batch cultivations of citrate by *A. niger* H915-1 in a 3-L fermenter

Batch cultivations were performed in 3-L fermenters with a working volume of 1.5 L, and the temperature was maintained at 35 °C. For inoculation of the bioreactor, the stirring rate was adjusted to 900 rpm and aeration was set at 3.5 volumes of air per volume of fluid per minute (vvm).

### High-performance liquid chromatography analysis

For quantification of extracellular metabolites, a culture sample was centrifuged at 14,000 × *g* for 5 min. The supernatant was immediately filtered through a filter with a 0.45-μm pore size. The filtrate was kept at −20 °C until for analysis. Citrate and oxalate concentration was detected and quantified with ultraviolet light at 210 nm by using an Amethyst C18-H column (250 × 4.6 mm; Sepax Technologies, Newark, DE, USA). Elution was carried out at 30 °C with 0.01% H_3_PO_4_ at a flow rate of 0.8 mL/min.

### Reducing sugar assay

Reducing sugar was detected with the 3,5-dinitrosalicylic acid reducing sugar method^10^.

### Biomass determination

Five milliliter of sample was filtrated through Miracloth (Calbiochem, San Diego, CA, USA) to collect the hyphae and washed with distilled water. The hyphae in miracloth was heated at 105 °C until the weight did not change. For calculation of dry cell weight (DCW), the weight of Miracloth was measured previously and deducted from the total weight to generate net weight, then the net weight per unit volume was calculated as DCW.

### *A. niger* cultivation and DNA preparation for genome sequencing and annotation

Conidia (1 × 10^6^) were cultivated in 100 mL malt extract liquid medium (3% malt extract and 0.5% tryptone) for 2 days at 35 °C. The mycelia were harvested with Miracloth and frozen in liquid nitrogen. The genome DNA of *A. niger* was isolated with a DNeasy Plant Mini Kit (QIAGEN, Germantown, MD, USA) and subjected to quality control and library construction for genome sequencing.

### Next-generation sequencing and genome assembly

Genome sequencing of *A. niger* H915-1, L2, and A1 was performed with an Illumina Miseq 2000 system using paired-end libraries. After clean data were obtained, reads of *A. niger* A1 were assembled by using the Celera Assembler with the optimal linear combination algorithm^11^. The sequence data for *A. niger* A1 were deposited at DDBJ/EMBL/GenBank under the accession number LMYC00000000.

### Third-generation sequencing and genome assembly

Genome sequencing of *A. niger* H915-1 and L2 were performed by using a PacBio RS II system with a 20-kb library. Sequences were de novo assembled with the hierarchical genome assembly process^12^. The longest sequencing reads were selected as a seeding sequence data set, according to which, as a reference, shorter reads were recruited and preassembled by using basic local alignment with successive refinement^13^. These preassembled reads were assembled by using the Celera Assembler with the optimal linear combination algorithm^11^. Then, the assembly was refined by using initial read data, and accuracy was improved by using Quiver^12^. Subsequently, minimus2 was used to connect the contigs and generate the final consensus that represented the genome^14^. Finally, the sequences generated by next-generation sequencing were mapped to the assembled genome for error correction by using BWA. Sequence data for *A. niger* H915-1 and L2 were deposited at DDBJ/EMBL/GenBank under the accession numbers LLBX00000000 and LKBF00000000, respectively.

### Gene prediction and annotation

Several gene-finding software programs were used for predicting genes. AUGUSTUS^15^, SNAP^16^, and GeneMark + ES^17^ were used for ab initio prediction, and Genewise^18^ was used for homology prediction. Subsequently, EVidenceModeler was used to incorporate all of the predicted results to generate final general feature format file^19^.

Protein sequences of all predicted genes were mapped to a non-redundant protein database (nr, National Center for Biotechnology Information), SWISS-PROT, and the TREMBLE database by using BLASTP with an E-value of 1e-5. Gene ontology (GO) annotation was performed by using interproscan-5.4-47.0 to blast to quick GO from the Interpro database. The amino acid sequences of all predicted genes were mapped to the Eukaryotic Orthologous Group and Kyoto Encyclopedia of Genes and Genomes databases by using BLASTP with an E-value of 1e-5.

Prediction of transfer RNA (tRNA) was carried out by using tRNAscan-SE 1.23, which combines several detection programs and analysis models^20^. Annotation of ribosomal RNA (rRNA) was finished by using RNAmmer 1.2 with hidden Markov models^21^.

### Repeat element analysis

Repetitive sequences were detected according to Repbase by RepeatMasker by using the default parameters^22^. Then, RepeatProteinMask was executed to search against the transposable element protein database to identify repeat related proteins. In addition, the tandem repeats were annotated with using the Tandem Repeats Finder program.

### Prediction of ortholog group

Blastp was first used to generate the pairwise protein sequence with similarity of E-value less than 1e-5. Secondly, OrthoMCL was used to cluster similar genes by setting main inflation value 1.5 and other default parameters^23^.

### Variants analysis

Single-nucleotide polymorphisms (SNPs) and insertions and deletions (indels) were detected by using SAMtools and comparing the genomes of *A. niger* L2 and A1 to *A. niger* H915-1. Then, SnpEff was used to annotate the effects of the variants^24^. Structural variation (SV) was detected by using Pindel.

### RNA extraction and purification for RNA-seq

Mycelia were frozen and ground under liquid nitrogen by using a mortar and pestle. RNA was isolated with an RNeasy Plant Mini Kit (QIAGEN, Germantown, MD, USA) according to the manufacturer’s instructions and checked for RNA integrity by using an Agilent 2100 Bioanalyzer (Agilent Technologies, Santa Clara, CA, US). Qualified total RNA was further purified successively with an RNeasy micro kit (QIAGEN) and RNase-Free DNase Set (QIAGEN).

### Complementary DNA (cDNA) library preparation and sequencing

Purified total RNA was digested to eliminate rRNA. The RNA was then fragmented by heating at 94 °C and used to synthesize cDNA. The double-stranded cDNA was adenylated at the 3′ end, then ligated to the sequencing adapters. Pair-end sequencing was performed by using Illumina HiSeq 2500 according to the manufacturer’s protocols.

### Analysis of Illumina transcriptome sequencing results

Raw data were filtered to remove low-quality sequences by using FASTX (version 0.0.13; http://hannonlab.cshl.edu/fastx_toolkit/index.html) to generate clean data. Clean reads were mapped to the genome of *A. niger* H915-1 as a reference by using TopHat (version 2.0.9)^25^ according to the spliced mapping algorithm. The Cufflinks program (version 2.1.1)^26^ was used to calculate unigene expression with the using FPKM method (Fragments per kb per million reads). Gene expression differences among the samples were analyzed by using Cufflinks (version 2.1.1) with a false discovery rate of ≤0.05 and a fold change of ≥2. A Perl script was used to assign a functional class to each unigene and establish pathway associations between unigenes and the Kyoto Encyclopedia of Genes and Genomes database. The sequence data have been deposited in the DDBJ/EMBL/GenBank database under the GEO accession number GSE74544.

### Real-time Quantitative PCR (qPCR)

For verification of transcriptome data, qPCR was performed by LightCycler 480 system (Roche, Germany) with 2 × SYBR Premix Ex Taq (Takara, Dalian, China). The primers were designed by Beacon Designer 7 and the sequences were listed in Supplementary Table S1.

As the major genes of central metabolism were identified by transcriptome, these genes’ expression levels of H915-1 at 48 h and A1 at 60 h were compared. The primers used were also listed in Supplementary Table S1.

### Construction of the gene-knockout cassette for *oah*

The 2.3-kb sequence upstream of *oah*, called oahA5, was cloned by using primers (Supplementary Table S1) of oahA5-F and oahA5-R and the genome of *A. niger* H915-1 as a template. The 2.3-kb sequence downstream of *oah*, called oahA3, was cloned using primers of oahA3-F and oahA3-R. The sequence oahA3 was digested with *Spe* I and *Hin d*III and subsequently ligated to pAN7-1 (GenBank accession no. Z32698) and digested with *Xba* I and *Hin* dIII to construct plasmid pAN-oah3. pAN-oah3 was digested with *Xho* I and *Xba* I, ligated to the plasmid pSZH-XynB^27^, and digested with *Xho* I and *Nde* I to construct the plasmid pSZH-oah3. pSZH-oah3 was digested with *Xba* I and *Xho* I, ligated to oahA5, and digested with *Xba* I and *Sal* I to construct the plasmid pSZH-oahA. The gene-knockout cassette of *oah* was obtained through PCR with primer oahA5-F and oahA3-R by using pSZH-oahA as a template. The primers used to validate the recombinant genome were P1, P2, P3, and P4 (Supplementary Table S1).

### Transformation of *A. niger*

Protoplast formation and transformation was performed based on a method published by Blumhoff *et al*.^28^. Conidia (1 × 10^8^) were cultivated in 100 mL malt extract medium for 11 h at 35 °C. The mycelium was harvested via filtration through Miracloth (Calbiochem) and washed with deionized water. Protoplastation was achieved in the presence of 5 g L^−1^ lysing enzymes from *Trichoderma harzianum* (Sigma. Saint Louis, MO, USA), 0.075 U mL^−1^ chitinase from *Streptomyces griseus* (Sigma), and 460 U mL^−1^ glucuronidase from *Helix pomatia* (Sigma) in KMC (0.7 M KCl, 50 mM CaCl_2_, 20 mM Mes/NaOH, pH 5.8) for 2 h at 37 °C and 120 rpm. Protoplastation was monitored every 30 min with a microscope. The protoplasts were filtered through Miracloth and collected via centrifugation at 2,000 × *g* and 4 °C for 10 min. The protoplasts were washed with cold STC (1.2 M sorbitol, 10 mM Tris/HCl, 50 mM CaCl_2_, pH 7.5) and subsequently resuspended in 100 μL STC and directly used for transformation. Ten micrograms of linear knock-out cassette was mixed with 100 μL STC solution containing at least 10^7^ protoplasts and 330 μL freshly prepared polyethylene glycol (PEG) solution (25% PEG 6000, 50 mM CaCl_2_, 10 mM Tris/HCl, pH 7.5) and kept on ice for 20 min. After mixing with an additional 2 mL PEG solution and incubating at room temperature for 10 min, the protoplast mixture was diluted with 4 mL STC. The aliquots were mixed with 4 mL liquid top agar warmed to 50 °C, spread on bottom agar containing 180 μg mL^−1^ hygromycin, and incubated at 35 °C for 3–6 days. All transformants were purified three times via single-colony isolation on the selection medium. The correct integration was verified with PCR analysis by using specific genomic primers. The fermentation was performed for 3 times and the data of production and yield were analyzed for the statistical significance of differences by Tukey’s honestly significant difference text (T-text) using a standard package (SPSS for Windows, Version 17; SPSS Inc. Chicago, IL, USA).

## Results and Discussion

### Citrate production by *A. niger* H915-1, L2, and A1

**Figure 1:**
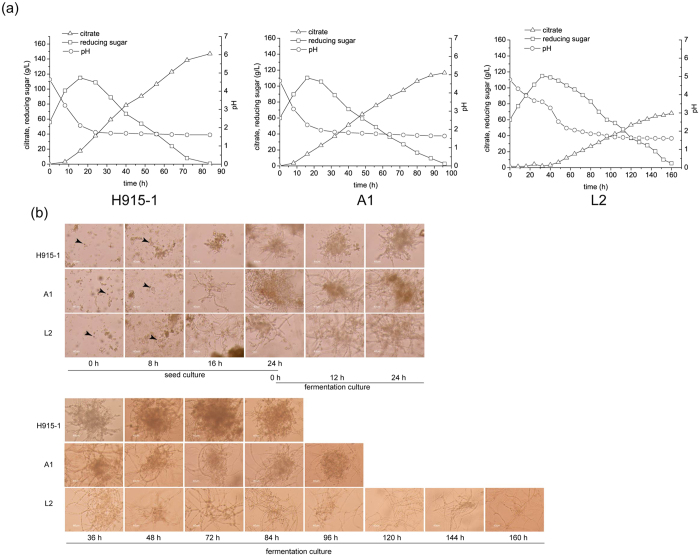
Citrate fermentation of different *A. niger* strains. (**a**) Citrate titer and fermentation time. Δ, citrate production. □, concentration of reducing sugar. ○, pH. (**b**) Morphology of different strains. Bar represents 40 μm. Black arrow pointed to a conidium.

**Figure 2:**
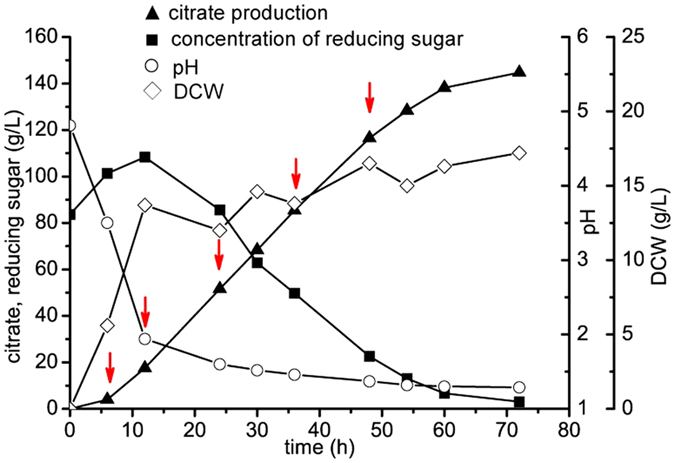
Fed-batch culture of *A. niger* H915-1 for citrate production. Aeration was maintained at 3.5 vvm. Sample time was shown in red arrows. ▲, citrate production. ■, concentration of reducing sugar. ○, pH. ◊, DCW.

Among *A. niger* strains with different citrate production efficiencies, *A. niger* H915-1 produced the highest citrate titer of 157 g L^−1^ in 85 h, whereas *A. niger* A1 produced 117 g L^−1^ in 92 h and *A. niger* L2 produced 76 g L^−1^ in 160 h (Fig. 1a). We found that during citrate fermentation, the mycelia of different strains aggregated in various forms (Fig. 1b), which indicated that citrate production was influenced by the morphology of the microcolonies^6^. *A. niger* H915-1 formed bulbous hyphae with short, swollen hyphal branches, whereas *A. niger* A1 formed less compact pellets with less hyphal branching and longer mycelia, and *A. niger* L2 formed mycelial clumps. These differences in morphology may influence the viscosity of the medium and further affect hyphal respiration^29^. The tight pellet form facilitated citrate formation, and the diffused filamentous form reduced citrate production and productivity. In addition, aeration rate was an important parameter for citrate production. When aeration was maintained at 3.5 vvm, the citrate production of *A. niger* H915-1 reached 145 g L^−1^ in 72 h (Fig. 2).

### General genome statistics and differences among the three strains

Three *A. niger* strains (H915-1, A1, and L2) capable of gradient citrate production were selected for genome sequencing. The genome sequences of *A. niger* H915-1 and L2 were obtained with a PacBio RS II system, and *A. niger* A1 was sequenced by using whole-genome shotgun paired-end sequencing. Sequences of *A. niger* H915-1 and L2 were further improved to high-quality assemblies of 30 finished contigs (Table 1). The assembled genome of *A. niger* A1 consisted of 319 contigs. The genome sizes of *A. niger* H915-1, L2, and A1 were 35.98 Mb, 36.45 Mb, and 34.64 Mb, respectively, and the number of predicted genes ranged from 10,123 to 10,433—approximately 26% smaller than *A. niger* CBS 513.88 and 10% smaller than *A. niger* SH2, both of which are enzyme production strains. The number of tRNA in *A. niger* H915-1 and L2 were more than 2-fold those of *A. niger* A1, CBS 513.88, and SH2. To investigate the reason of dramatic changes on tRNA number, the genome of H915-1 was assembled either de novo or using LLBX00000000 as reference only with the “next-generation” sequencing data. The de novo assembly genome was 35.5 M with 255 tRNA, and the genome assembled using LLBX00000000 as reference was 36.0 M with 667 tRNA, indicating that the problem of losing information by second-generation sequencing was not significant, but the assembly method influenced the genome result a lot and also the “next-generation” sequencing with different read lengths was importance for rectify assembly results^30^,^31^. The ortholog names was provided in Supplementary Table S2 for matching gene ID of H915-1 to gene ID of CBS513.88.

**Figure 3:**
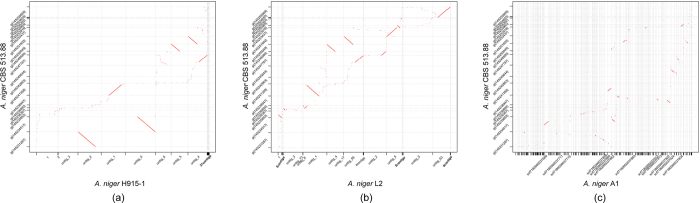
Syntenic dot plot of *A. niger* H915, *A. niger* L2 and *A. niger* A1 with *A. niger* CBS 513.88. (**a**) *A. niger* H915. (**b**) *A. niger* L2. (**c**) *A. niger* A1.

The Clusters of Orthologous Groups (COG) is a database where the orthologous gene products were classified. We searched the annotated sequences for the genes involved in COG classifications and more than 3,700 sequences had a COG classification. Compared with strain L2 and A1, there are more genes belonging to categories A (RNA processing and modification), C (Energy production and conversion), F (Nucleotide transport and metabolism), G (Carbohydrate transport and metabolism) and S (Intracellular trafficking, secretion, and vesicular transport) in strain H915-1 (Supplementary Table S3). The three strains shared 9097 gene groups (Supplementary Figure S1). Compared with *A. niger* L2 and A1, *A. niger* H915-1 contained only 1 unique gene group and lacked 58 groups of genes (Supplementary Table S4). Syntenic dot plot analysis of the three experimental strains and *A. niger* CBS 513.88 (Fig. 3) and extensive structural reorganizations were observed. When compared to genome of H915-1 as reference, the genome of L2 and A1 contained 1210 SNP/indel and 52 SVs, within which existed 57 non-synonymous SNPs (Supplementary Tables S5 and S6) and 35 SVs involving gene mutation (Supplementary Table S7).

For the genes mutated in strains of A1 and L2, the most notable was a succinate-semialdehyde dehydrogenase involved in γ-aminobutyric acid (GABA) shunt pathway for succinate supplement in TCA cycle, indicating that metabolite flux was modulated for citrate production. Also, a mutation was found in an aconitase family protein, which could relate to the production of citrate. Furthermore, mutations of a proline utilization trans-activator and a branched-chain-amino-acid aminotransferase indicated amino acid metabolism may relate to citrate synthesis. The synthesis of amino acids were started from organic acids in glycolysis and TCA cycle. Pyruvate, 3-phospho-D-glycerate, oxaloacetate and 2-oxoglutarate were all precursors of amino acids, as a result, amino acids synthesis pathway could drain metabolites used in organic acids synthesis. In addition, SVs were found in 60S ribosomal protein L5 relevant for mediating 5S rRNA binding^32^, DNA repair protein, DNA-directed RNA polymerase II subunit, RNA-splicing protein and mRNA transport regulator, suggesting changes on cell viability. As citrate is a primary metabolite for *A. niger*, the production of citrate may associate with the vegetative growth rate.

For citrate fermentation of *A. niger*, the morphological development was considered to start with the aggregation of conidia right after inoculation, then the conidia begun to germinate and formed mycelium^33^,^34^. The pathways for formation of hydrophobin and melanin (Supplementary Table S8), which induced conidia aggregation and subsequent germ tube aggregation^34^,^35^,^36^ had no differences, though the 3 strains showed different morphology of micro-colonies. Fungal cell wall consists of 80–90% polysaccharides, and most of the remainders are protein and lipid^37^. The functions of these diverse cell wall proteins included defense response^38^, maintaining cell surface hydrophobicity^39^, and regulation of morphogenesis^40^. The cell wall protein *lrx1* mutated *Arabidopsis thaliana* developed swell and branch root hairs^40^. In this study, a cell wall protein lacked in H915-1, and there existed the possibility to influence the cell morphology.

### Transcriptome analysis of H915-1 during citrate production

To obtain a general picture of *A. niger* cell physiology during citrate production, we studied the transcriptome of H915-1 during this process in a 3-L fermenter. In the case that aeration was maintained at 3.5 vvm, hyphae were sampled at 6 h, 12 h, 24 h, 36 h and 48 h. When fermented for 6 h, the medium pH dropped from 4.8 to 3.5, and the hyphae were still at growing age and citrate productivity was slow at 0.66 g L^−1^ h^−1^. Subsequently, citrate productivity increased to 2.34 g L^−1^ h^−1^, 3.26 g L^−1^ h^−1^, 2.75 g L^−1^ h^−1^, and 1.94 g L^−1^ h^−1^ at 12 h, 24 h, 36 h, and 48 h, respectively (Fig. 2).

**Figure 4:**
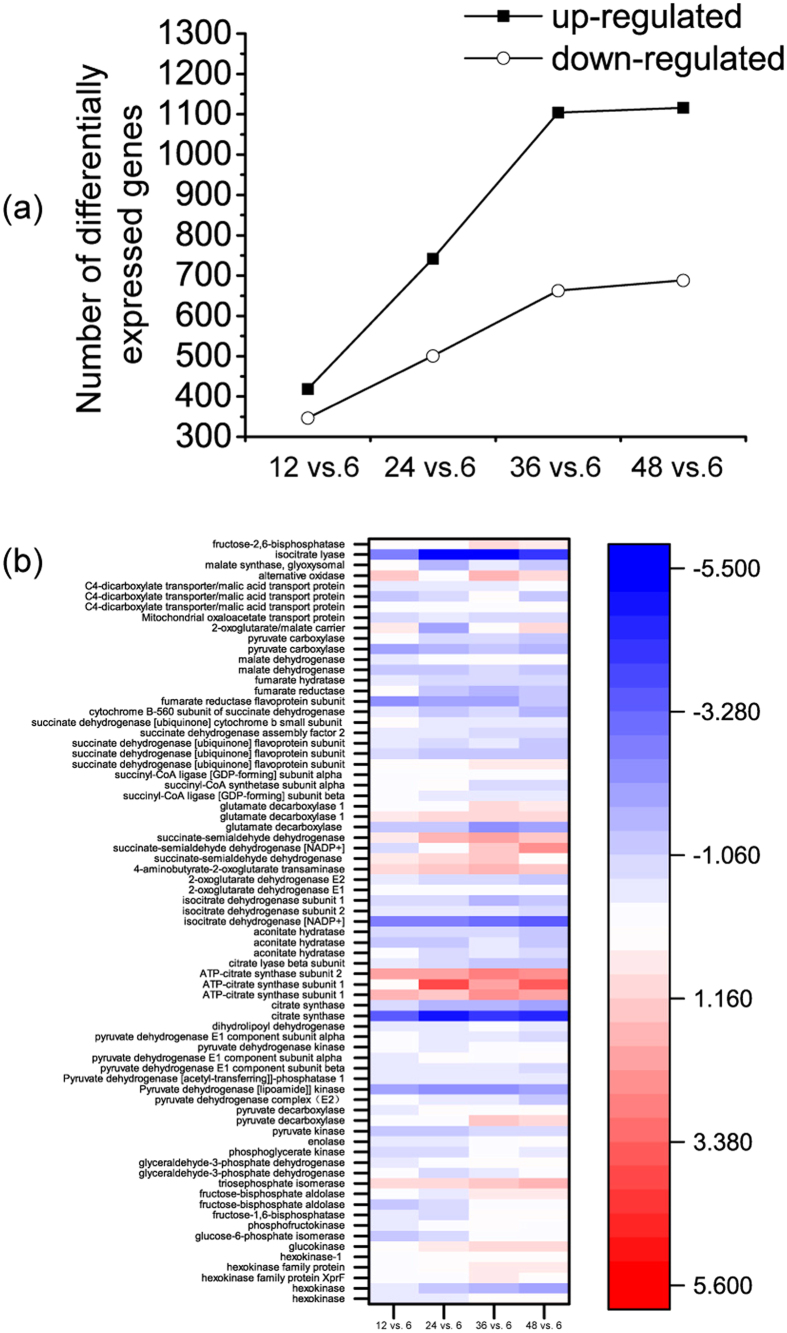
Transcriptional profile during citrate fermentation. (**a**) Number of differentially expressed genes during fermentation. (**b**) Expression profile of genes in glycolysis and TCA cycle.

Compared with expression at the cell growth stage (6 h), the expression of 479 of 9953 genes changed at all 4 time points during the citrate synthesis stage (12 h, 24 h, 36 h, and 48 h): 269 genes showed increased expression levels, and 210 genes were down-regulated (fold change ≥ 2 and false discovery rate ≤ 0.05). These results identified the key genes for citrate production. In addition, the number of differentially expressed genes increased with fermentation time (Fig. 4a), which indicated a strong cellular response to the changing conditions of citrate secretion.

For verification of the transcriptome data, the fermentation was repeated and sampled at the same time as that for transcriptome analysis. Several genes including genes in central metabolism and 2 other genes (evm.model.1.1208 and evm.model.unitig_3.181), which increased greatly during fermentation, were analyzed by qPCR and the fold change of gene expression level was compared with that of the transcriptome data (Supplementary Figure 2). The result showed similar trend by qPCR and transcriptome analysis. The expression level of evm.model.1.1208 and evm.model.unitig_3.181 all increased by similar folds at 12, 24, 36 and 48 h compared to 6 h in both experiments, and the expression level of genes in central metabolism were also up-regulated/down-regulated at similar levels in both experiments, confirming the result of transcriptome analysis.

### Regulation of central metabolism (glycolysis, TCA cycle, rTCA cycle and GABA shunt pathway) for citrate production

**Figure 5:**
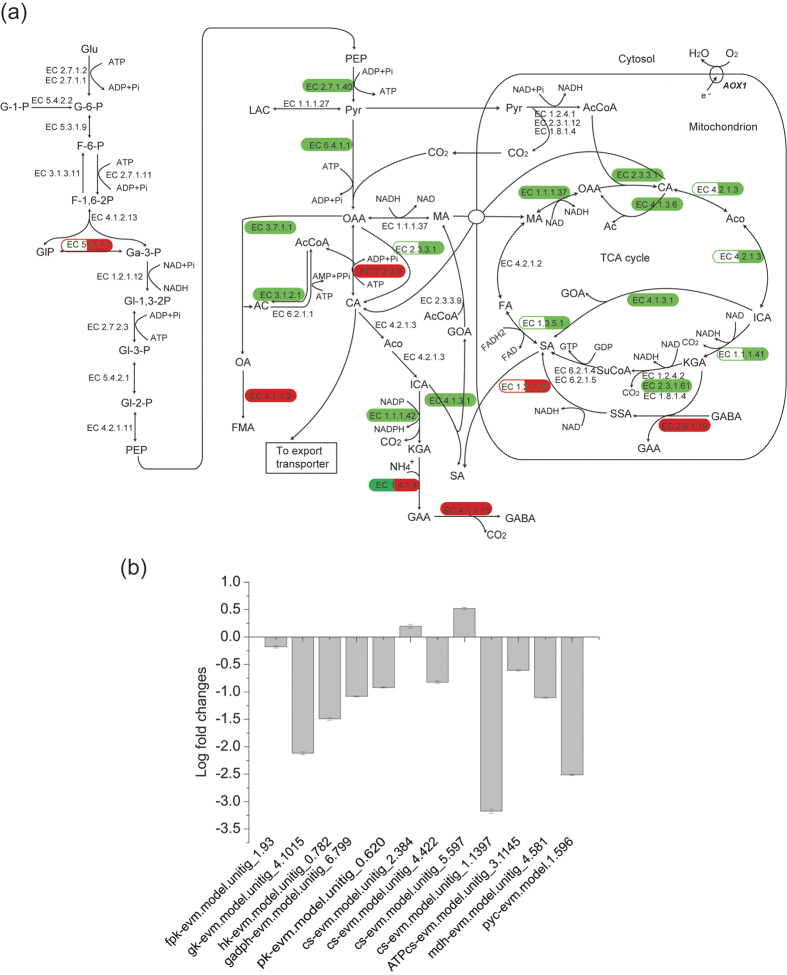
Transcription level regulation of genes in central metabolism. (**a**) Transcription level regulation of genes in glycolysis and TCA cycle of H915-1 during citrate production. Red ellipse means up-regulated genes. Green ellipse means down-regulated genes. Half blank ellipse means that expression levels of some isoenzymes were not changed, while the other isoenzymes were affected. Abbreviations: GABA, 4-aminobutanoate; AC, acetate; AcA, acetaldehyde; AcCoA, acetyl-CoA; Aco, cis-aconitate; AOX1, alternative oxidase; CA, citrate; F-1,6–2P, fructose 1,6-bisphosphate; ETH, ethonal; F-6-P, fructose 6-phosphate; FA, fumarate; FMA, formate; G-6-P, Glucose 6-phosphate; Ga-3-P, glyceraldehyde 3-phosphate; GAA, glutamate; Glu, glucose; GI-1,3–2P, 3-phospho-D-glyceroyl phosphate; GI-2-P, 2-phospho-D-glycerate; GI-3-P, 3-phospho-D-glycerate; GIP, dihydroxyacetone phosphate; GOA, glyoxylate; ICA, isocitrate; KGA, 2-oxoglutarate; MA, malate; OA, oxalate; OAA, oxaloacetate; PEP, phosphoenolpyruvate; Pyr, pyruvate; SA, succinate; SSA, succinate semialdehyde; SuCoA, succinyl-CoA. (**b**) qPCR of gene expression fold changes of A1 at 60 h compared to H915-1 at 48 h. Error bars represent three technical replicates.

Citrate is formed mainly via cytosolic glycolysis and the subsequent mitochondrial TCA cycle. The expression levels of genes involved in glycolysis were mostly unaffected, but many genes involved in the TCA cycle were down-regulated (Fig. 4b, Table 2). Six isoenzymes catalyze glucose phosphorylation in *A. niger* H915-1. Of these, 5 isoenzymes are hexokinases, the transcription levels of which did not change noticeably. Hexokinase activity was non-competitively inhibited by citrate^41^, and to compensate for metabolite flux, the expression level of glucokinase was up-regulated gradually during citrate production (Fig. 5a).

A triosephosphate isomerase was up-regulated 2-fold during citrate production. Because 1 mol d-fructose 1,6-bisphosphate decomposed into 1 mol dihydroxyacetone phosphate and 1 mol d-glyceraldehyde 3-phosphate, only d-glyceraldehyde 3-phosphate remained as the substrate for the next enzyme in glycolysis. The triosephosphate isomerase must be up-regulated to form additional d-glyceraldehyde 3-phosphate.

Phosphofructokinase (PFK1) was another key regulator in glycolysis. The expression level of PFK1 changed only slightly. PFK1 was inactivated by high concentrations of citrate, ATP, and manganese; however, the inhibition can be antagonized by NH_4_^+^ ions and fructose-2,6-bisphosphate^42^.

After glucose is catabolized to pyruvate, a part of the pyruvate is transported into the mitochondria to form acetyl-coenzyme A (acetyl-CoA), which was the substrate of citrate synthase. There are 2 mitochondrial citrate synthases, all of which were down-regulated. The convesion rate of citrate did not seem to be a rate-limiting step for citrate production, and flux in citrate synthesis stage might even be reduced compared to that at 6 h.

The reactions that followed citrate synthase in the TCA cycle were all down-regulated at the transcription level. Among 4 predicted aconitases, 2 mitochondrial enzymes were highly transcribed and their expression levels decreased. Of the 3 isocitrate dehydrogenases in *A. niger* H915-1, the cytosolic NADP^+^-dependent form and 1 mitochondrial NAD^+^-dependent form were down-regulated.

2-Oxoglutarate (KGA) dehydrogenase was repressed at both the transcriptional level and the post-translation level^6^, and the GABA shunt pathway was up-regulated for succinate acid supplementation. KGA and GABA were catalyzed to produce succinate semialdehyde, which was further transformed to succinate. This situation is identical to that observed in *A. niger* CBS 513.88, which depends completely on the alternative respiration pathway because the succinate-CoA ligase complex is absent during fermentation according to transcription data^4^. This pathway is also important for KGA production in *Yarrowia lipolytica*^43^.

The intermediate glutamate enhances acid tolerance by consuming intracellular protons via amino acid decarboxylation, which helps increase intracellular pH^44^,^45^. The pathway is also involved in the release of NH_4_^+^ ions into the cytosol, which antagonizes citrate inhibition of phosphofructokinase^6^.

The oxaloacetate was formed either from TCA cycle or rTCA cycle. The former way lose two moles of carbon dioxide, while the latter way associated with fixing CO_2_ to pyruvate by pyruvate carboxylase to form oxaloacetic acid (OAA), which is subsequently reduced to malate by malate dehydrogenase and enters the mitochondria via a malate-citrate antiporter. Subsequently, malate participates in the TCA cycle and forms citrate. *A. niger* H915-1 contained 2 genes for pyruvate carboxylase, and only the cytosolic gene was observed to be transcribed. This gene was down-regulated dramatically to one-third the level observed at 6 h compared with citrate-synthesis-stage. For the 3 cytosolic malate dehydrogenases, only one was dramatically transcribed during fermentation, and malate dehydrogenase located in the mitochondrion was down-regulated.

Most of the genes in central metabolism were reported to be down regulated during citrate fermentation compared to cell-growing-phase, and the explanation for this situation was that as the broth pH decreased to extreme low level, the basic cell metabolism was down regulated for response of low pH. Though the expression was down-regulated, the expression level of FPKM was still high for citrate formation. The noticeable high metabolism during cell-growing-phase was reasonable because the cell weight was increased dramatically during the first 12 h (Fig. 2). As the major genes of central metabolism were identified, the genes expression level of strain A1 at 60 h was analyzed by qPCR compared to H915-1 at 48 h (Fig. 5b) and most of the genes were depressed in A1, indicating the higher genes expression level was responsible for higher citrate production.

### Up-regulation of ATP-citrate lyases coordinate with the alternative respiratory pathway for energy balance

During citrate fermentation, energy balance was important because the conversion of citrate from glucose generated 1 mol ATP and 3 molecules of NADH, which was redundant and needed to be consumed^46^. The alternative pathway took charge of oxidizing excess NADH without forming ATP and the alternative oxidase was the core enzyme in the pathway^47^. In this study, the expression of alternative oxidase was mostly up-regulated at citrate-synthesis-phase compared to cell-growing-phase, confirming the importance of the pathway for citrate fermentation^8^. Nevertheless, the expression levels of alternative oxidase were low compared to other major genes of central metabolism, indicating that the other pathway for ATP consumption may be needed to help balance the energy. Notably, 2 cytosolic ATP-citrate lyases were up-regulated, which took part in a futile cycle. The mitochondrial citrate was transported to the cytoplasm, most of which was transported outside the cell and a small amount was catalyzed to generate OAA by ATP-citrate lyase with ATP consumption. Subsequently, OAA was transformed to malate and resupplied for mitochondrial citrate formation. This cycle consumed 1 mol ATP and the up-regulation of 2 cytosolic ATP-citrate lyases indicated that the pathway may help releaseing the pressure of the alternative respiration pathway for the recycling of NADH.

### Regulation of heteroacid formation genes

*A. niger* strives to produce - at a given pH - the organic acid that most efficiently acidifies the medium. *A. niger* can produce several organic acids, including gluconate, oxalate, lactate, malate, succinate, and citrate^48^. Gluconate and oxalate are the main heteroacids that affect citrate production under certain conditions^49^. The secretion of heteroacid acids helps to decrease medium pH to below 3.0, which is crucial for citrate production^6^,^48^, and after citrate formation was triggered, the major role for acidification the medium was played by citrate. The expression profiles of genes involved in the formation of the primary heteroacids are shown in Supplementary Table S9.

The oxaloacetate acetylhydrolase (evm.model.unitig_6.59) catalyzed the degradation of oxaloacetate to form oxalate and acetate. The *oah* gene was highly expressed at 6 h of fermentation, which was in agreement with the report by Ruijter *et al*. (1999) that oxalate is the preferred acid in a strain capable of producing organic acids, and in Andersen’s stoichiometric model of *A. niger* metabolism, that the simulations predicted oxalate as the only produced organic acid throughout the pH range from 1.5 to 6.5^48^.

Subsequently, the expression of *oah* gene was depressed and oxalate decarboxylase (evm.model.1.1180) was expressed at a constitutively high level during the citrate production period, thereby guaranteeing the elimination of oxalate after citrate begun to form and citrate became the major acid in the medium, and the situation also agreed with the Andersen’s stoichiometric model that citrate is the optimal acid for medium acidification when oxalate cannot be produced when pH ranged from 1.5 to 2.5^48^. The reason of the trait remains obscure, but it may be an evolutionary strategy and one of the hypotheses suggests that low pH helps degrade plant cell walls, facilitating saprotrophic living of fungus, that it inhibits the growth of competing organisms, and that the acids chelate trace metals, making them available to the fungus^50^.

At the same time of oxalic acid formation, the same amount of acetate was produced in cytosol by oxaloacetate hydrolase. In addition, high expression level of acetyl-CoA hydrolase (evm.model.unitig_6.429) at 6 h further increased the acetate synthesis in *A. niger*. Nevertheless, acetate accumulation did not occur in the medium, suggesting the catabolic rate of acetate was sufficient to prevent its formation as Ruijter *et al*. (1999) reported. The result was also in agreement with the simulations predict by Andersen *et al*. (2009) that it is more energetically efficient to re-metabolize acetate than to use it for acidification of the medium. However, in the later fermentation phase, the expression levels of both oxaloacetate acetylhydrolase and acetyl-CoA hydrolase were dramatically down-regulated, indicating the decrease of acetate pathway and a main metabolite flux into the TCA cycle for citrate production.

*A. niger* H915-1 had only 1 glucose oxidase (evm.model.unitig_0.36), which was an extracellular enzyme and catalyzed the formation of gluconic acid from glucose in broth. The enzyme was only expressed during the early stage of fermentation but silent later. This result was in agreement with the report that gluconic acid is an early product during citrate fermentation^51^. In addition, the enzyme was inactivated at pH values below 3.5, and this further guaranteed the elimination of gluconic acid formation when producing citrate^6^.

### Regulation of transporters

Elucidating the transport of both sugars and citrate is crucial to understanding citrate overproduction by *A. niger*. However, disagreement exists about the mechanisms underlying both the uptake of glucose and the secretion of citrate ions. Overall, 46 transporters were expressed differently at the transcriptional level during citrate production (Table 3).

Both simple diffusion and sugar transporters were found responding for glucose uptake during citrate producing^52^,^53^,^54^. In this study, 28 sugar transporters were expressed, among which a low-affinity glucose transporter (evm.model.unitig_0.1567) was constitutively expressed during fermentation at high level, and 5 high-affinity glucose transporters were expressed constitutively at low level, and a hexose transporter was up-regulated. The result supported the suggestion that a low-affinity glucose transporter has a major function in glucose catabolism among all sugar transporters^55^. Nevertheless, the low-affinity glucose transporter was formed when grown on a high (15% w/v) glucose concentration in previous report^55^, but in this work, the transporter could be constitutively expressed during citrate fermentation even at 48 h when the glucose concentration decreased to 8%. Furthermore, the roles of high-affinity glucose transporters could be confirmed as glucose can be consumed entirely in late stage^6^.

The mechanism for citrate transportation through the plasma membrane requires further study. Because a dramatic pH gradient exists between the plasma and the extracellular medium, citrate is produced mainly as citrate^2−^ in the cytosol (with a pH between 6.0 and 7.0) and as an undissociated form of acid in medium (with a pH below 2.0)^56^. Citrate^2−^ can be secreted from *A. niger* into broth, and the hyphae can take up citrate at the end of citrate fermentation, which indicates that both of the 2 forms of citrate can cross the cell wall. Nevertheless, no citrate transporter has yet been identified. In this study, 35 transporters were identified as being up-regulated during citrate fermentation, among which 3 organic anion transporters, a low-affinity iron transporter, and a monocarboxylate transporter were found. In addition, the transcription levels of 11 uncharacterized transporters increased. These transporters constituted candidate citrate transporters.

### Regulation of genes involving cell wall components

The main cell wall of *A. niger* contains β-1, 3-glucan, chitin, β-1, 6-glucan, α-1, 3-glucan, galactosaminogalactan, and galactomannan^57^. We found that expression levels of several genes involved in cell wall integrity were up-regulated during citrate production (Supplementary Table S10). Chitin synthase C was highly up-regulated. Pst1 was previously identified as a cell surface glycosylphosphatidylinositol-anchored protein important for cell wall integrity^58^,^59^ and the expression of *PST1* increased during fermentation, helping maintain cell wall strength for resistance to the low pH of the medium. The α-1,3-glucan usually exists on the cell wall of conidia and glues melanin to the conidial surface^60^, and it also plays an essential role in the aggregation of conidia^61^,^62^. The expression of alpha-1, 3-glucan synthase ags1 was up-regulated during citrate fermentation, which indicated that it might also play a role in the aggregation of hypha. In addition, UDP-*N*-acetylglucosaminyltransferase and *N*-acetylglucosaminidase were both up-regulated, which indicated the synthesis of various types of glycoproteins and *N*-glycans^63^. Furthermore, 3 endoglucanases were down-regulated to reduce cell wall degradation.

### Elimination of oxalate formation and increased citrate production through deletion of *oah* in *A. niger*

*A. niger* H915-1 expressed *oah* dramatically and resulted in oxalate synthesis early in the fermentation process. The role of oxalate on citrate production still needed to be confirmed. The production of oxalate may facilitated the acidification of medium and guarantee a low pH for citrate fermentation. However, the synthesis of oxalate may drain metabolites used for cell growth and influence subsequent citrate fermentation. As a result, the *oah* gene was knocked out to examine its role in citrate fermentation.

**Figure 6:**
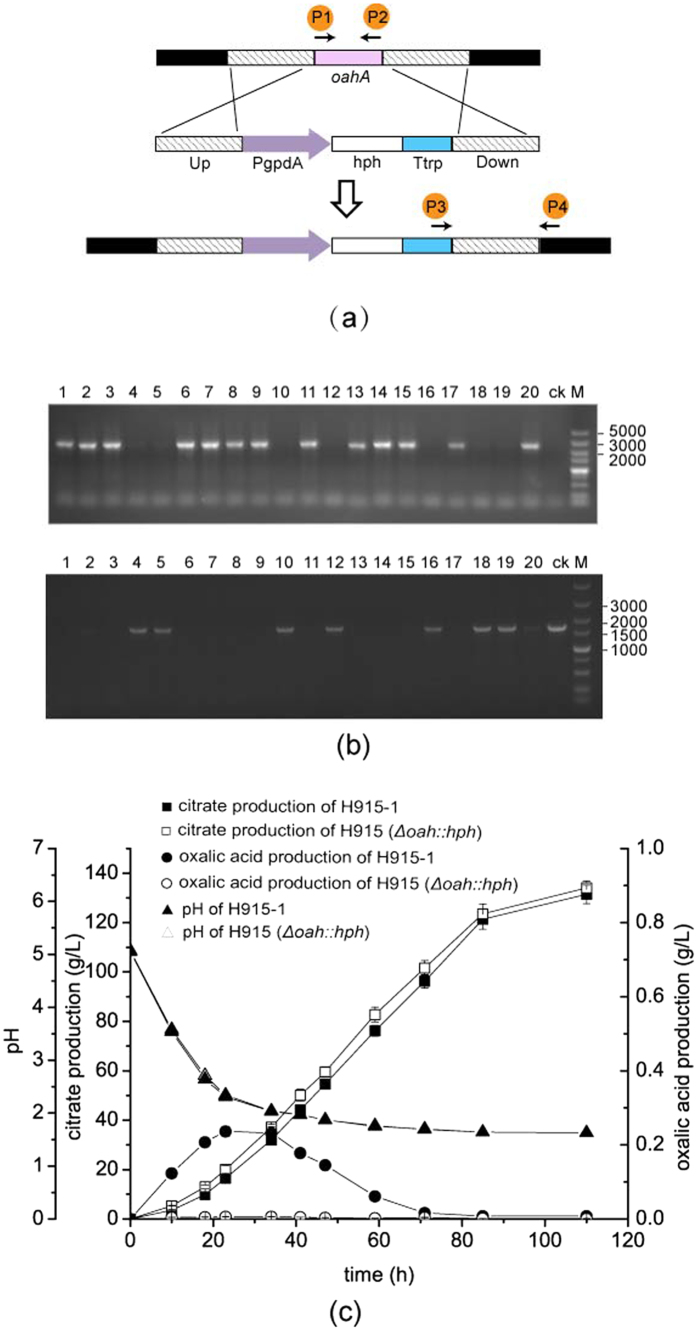
Deletion of *oah* in *A. niger* resulted in oxalate eliminated and slightly increased citrate production yield. (**a**) A graphical representation showing gene deletion of *oah* in *A. niger* and primers used for validation of recombinated genome. (**b**) PCR products of the sequences down-stream of *oah* using primers P3 and P4 (upper) and *oah* gene using primers P1 and P2 (below). Lane 1–20, *A. niger* transformants; Lane CK, wild type of H915-1; Lane M, DNA ladder. (**c**) Citrate and oxalate production of H915-1 and H915 (*Δ*oah::hph).

We obtained an *oah* gene deletion strain (Fig. 6a and b) and found that when the length of the flanking DNA reached 2.3 kb, the homologous recombination rate was 65%. During citrate fermentation in *A. niger* H915-1, oxalate was synthesized earlier than citrate and then eliminated later in the fermentation phase. When *oah* was deleted, the strain no longer synthesized oxalate (Fig. 6c). The pH during fermentation between H915-1 and H915 (*Δoah::hph*) was not different, indicating that though oxalate production had impact on pH decrease, it was not the decisive factor due to diverse organic acids synthesized by *A. niger*. In addition, the deletion of *oah* in H915-1 did not influence the biomass either, suggesting the extremely high expression of *oah* drained little flux from cell growth, which was the same with the phenomenon of oah-deleting *A. niger* strain for enzyme production^64^. Finally, the influence of citrate production through the deletion of *oah* was not significant. Though the citrate yield was up-regulated by 1.7% (Supplementary Table S11), the difference was not statistical significant by t-test.

## Conclusion

The genome of industrial citrate-producing *A. niger* strain H915-1 was sequenced and compared with genomes of 2 strains that produce citrate at lower levels. SNP analysis revealed differences at 57 sites and 35 SVs in various genes in the *A. niger* H915-1 genome. These genes were involved in the TCA cycle, GABA shunt pathway, and other pathways. Transcriptome analysis of *A. niger* H915-1 during citrate fermentation showed that among the genes expressed during the cell growth stage (6 h), 479 genes were expressed differently at all 4 time-points of the citrate production stage (12 h, 24 h, 36 h, and 48 h). The data showed the expression profile of the major genes in the involved pathways. The up-regulation of ATP-citrate synthase may coordinate with the alternative respiratory pathway for energy balance. Furthermore, 35 transporters were up-regulated, which identified previously unknown candidate citrate transporters. In addition, to solve the problems of abundantly expressed *oah* and oxalate formation during the early phase of fermentation, we knocked out *oah* in *A. niger* H915-1, which neither affected citrate production nor influenced acidification of medium. The results of this study revealed the mechanism of citrate production in *A. niger* and contribute to future rational metabolic design.

## Additional Information

**How to cite this article**: Yin, X. *et al*. Comparative genomics and transcriptome analysis of *Aspergillus niger* and metabolic engineering for citrate production. *Sci. Rep.* **7**, 41040; doi: 10.1038/srep41040 (2017).

**Publisher's note:** Springer Nature remains neutral with regard to jurisdictional claims in published maps and institutional affiliations.

## Supplementary Material

Supplementary Figures and Tables

Supplementary Information

## Figures and Tables

**Table 1:** General features of genomes of *A. niger* H915-1, L2 and A1.

	*A. niger* H915	*A. niger* L2	*A. niger* A1
Genome size (Mb)	35.98	36.45	34.64
Contigs	30	30	319
N50 (bp)	4,441,427	4,157,665	247,692
Sequencing depth	88.05	88.07	83
Gene models	10,318	10,433	10123
Protein length (amino acids)	498	501	498
Exons per gene	3	3	3
Average gene length (bp)	1,787	1,781	1,756
Number of tRNA	666	557	278

**Table 2:** Transcription levels of genes involving glucose metabolism during citrate fermentation.

EC number	gene ID	Gene function	FPKM-6h	FPKM-12h	FPKM-24h	FPKM-36h	FPKM-48h
		glycolysis					
EC 2.7.1.1	evm.model.unitig_0.782	hexokinase	335.407	263.428	264.615	398.452	396.328
EC 2.7.1.1	evm.model.unitig_1.1360	hexokinase	0.799634	0.518701	0.376546	0.25131	0.219428
EC 2.7.1.1	evm.model.unitig_2.1160	hexokinase family protein XprF	12.973	13.2989	12.8881	18.8512	14.8755
EC 2.7.1.1	evm.model.unitig_4.158	hexokinase family protein	49.2115	49.5622	61.4378	65.9747	75.2536
EC 2.7.1.1	evm.model.unitig_6.819	hexokinase-1	19.7688	20.2945	25.5583	24.9562	25.509
EC 2.7.1.2	evm.model.unitig_4.1015	glucokinase	127.778	150.188	203.7	242.765	250.044
EC 5.4.2.2	evm.model.unitig_0.1273	phosphoglucomutase	92.2402	108.093	119.862	216.005	192.424
EC 5.4.2.2	evm.model.unitig_0.474	phosphoglucomutase	45.8516	54.1326	57.7454	77.0618	68.9388
EC 5.3.1.9	evm.model.unitig_6.538	glucose-6-phosphate isomerase	185.76	83.2219	101.433	153.62	149.017
EC 2.7.1.11	evm.model.unitig_1.93	phosphofructokinase	332.854	231.523	273.895	381.057	321.602
EC 3.1.3.11	evm.model.1.362	fructose-1,6-bisphosphatase	185.489	126.953	103.246	196.543	215.136
EC 4.1.2.13	evm.model.unitig_0.1289	fructose-bisphosphate aldolase	1415.9	608.32	742.795	1381.51	1233.53
EC 4.1.2.13	evm.model.unitig_5.221	fructose-bisphosphate aldolase	4.14539	3.74066	2.58741	7.12841	6.59831
EC 5.3.1.1	evm.model.unitig_5.192	triosephosphate isomerase	781.339	500.828	658.134	1048.47	1070.47
EC 5.3.1.1	evm.model.unitig_0.1612	triosephosphate isomerase	99.5623	205.847	190.824	264.163	351.897
EC 1.2.1.12	evm.model.unitig_4.875	glyceraldehyde-3-phosphate dehydrogenase	289.264	257.795	158.841	222.297	289.137
EC 1.2.1.12	evm.model.unitig_6.799	glyceraldehyde-3-phosphate dehydrogenase	2149.06	1463.62	1729.79	2551.67	2242.18
EC 2.7.2.3	evm.model.unitig_1.643	phosphoglycerate kinase	1092.79	558.191	673.403	922.674	866.895
EC 5.4.2.1	evm.model.unitig_6.503	phosphoglycerate mutase	95.5362	36.1718	64.161	84.4621	65.0384
EC 5.4.2.1	evm.model.unitig_6.710	phosphoglycerate mutase	769.017	423.86	524.205	666.103	634.037
EC 4.2.1.11	evm.model.unitig_6.540	enolase	60.0857	45.7904	43.6861	57.3401	76.4556
EC 4.2.1.11	evm.model.unitig_1.422	enolase	1916.1	1019.34	1313.81	1840.59	1892.42
EC 2.7.1.40	evm.model.unitig_0.620	pyruvate kinase	1765.16	838.18	840.933	959.252	922.378
		**tricarboxylic acid cycle**					
EC 2.3.1.12	evm.model.unitig_0.153*	dihydrolipoyllysine-residue acetyltransferase	496.018	402.791	346.071	317.373	227.624
EC 1.2.4.1	evm.model.unitig_0.667*	pyruvate dehydrogenase E1 component subunit alpha	297.87	261.578	236.515	237.141	162.256
EC 1.2.4.1	evm.model.unitig_2.1112*	pyruvate dehydrogenase E1 component subunit beta	219.671	169.985	139.605	155.358	124.71
EC 1.2.4.1	evm.model.unitig_3.851	pyruvate dehydrogenase E1 component subunit alpha	13.0844	9.68933	14.6326	11.1757	15.6863
EC 1.8.1.4	evm.model.unitig_0.477*	dihydrolipoyl dehydrogenase	137.583	104.234	95.4635	111.925	88.9546
EC 2.3.3.1	evm.model.unitig_4.422*	citrate synthase	887.236	277.72	153.559	146.579	146.101
EC 2.3.3.1	evm.model.unitig_5.597*	citrate synthase	1783.01	918.481	542.466	513.1	486.779
EC 2.3.3.1	evm.model.unitig_1.1397	citrate synthase	166.357	13.8929	5.34897	9.72465	6.61541
EC 2.3.3.1	evm.model.unitig_5.827	citrate synthase	55.1809	8.74314	38.5607	12.3544	26.6205
EC 2.3.3.1	evm.model.unitig_2.384	citrate synthase	2.52289	3.07562	36.8849	10.2526	29.7107
EC 2.3.3.8	evm.model.unitig_3.1144	ATP-citrate synthase subunit 2	17.2562	71.315	67.7677	118.432	94.7237
EC 2.3.3.8	evm.model.unitig_3.1145	ATP-citrate synthase subunit 1	28.8278	94.1346	80.0228	159.645	118.98
EC 4.1.3.6	evm.model.unitig_2.489*	citrate lyase beta subunit	36.8118	26.2511	17.9149	15.3349	14.9401
EC 4.2.1.3	evm.model.unitig_1.1226*	aconitate hydratase	896.845	720.406	447.551	601.52	551.053
EC 4.2.1.3	evm.model.unitig_5.803*	aconitate hydratase	69.8504	41.158	39.9385	40.9545	29.8112
EC 4.2.1.3	evm.model.unitig_0.1022	aconitase	0.05719	0	0.219753	0.054301	0
EC 4.2.1.3	evm.model.unitig_6.517	aconitate hydratase	3.2308	1.44962	1.23429	2.01631	1.53043
EC 1.1.1.41	evm.model.unitig_1.461*	isocitrate dehydrogenase (NAD^+^) subunit 1	651.892	397.868	316.08	238.206	261.487
EC 1.1.1.41	evm.model.unitig_1.881*	isocitrate dehydrogenase (NAD^+^) subunit 2	117.139	91.5155	77.0455	74.0661	66.8811
EC 1.1.1.42	evm.model.unitig_0.935	isocitrate dehydrogenase (NADP^+^)	379.561	55.4311	53.793	43.56	36.8787
EC 1.2.4.2	evm.model.1.395*	2-oxoglutarate dehydrogenase E1	123.4	101.134	68.2161	101.575	82.1217
EC 1.2.4.2	evm.model.unitig_1.1359*	2-oxoglutarate dehydrogenase	0.175744	0	0.082844	0	0.146153
EC 2.3.1.61	evm.model.unitig_3.373*	dihydrolipoyllysine-residue succinyltransferase	906.116	585.033	461.624	440.09	430.991
EC 1.8.1.4	evm.model.unitig_0.477*	dihydrolipoamide dehydrogenase	137.583	104.234	95.4635	111.925	88.9546
EC 6.2.1.4	evm.model.unitig_1.691*	Succinyl-CoA synthetase subunit alpha	10.1584	8.35142	10.9483	6.28717	6.26733
EC 6.2.1.4	evm.model.unitig_6.212*	succinyl-CoA ligase (GDP-forming) subunit alpha	167.72	151.294	152.653	134.668	143.569
EC 6.2.1.5	evm.model.unitig_5.547*	succinyl-CoA ligase (GDP-forming) subunit beta	187.764	164.558	147.869	139.546	136.451
EC 1.3.5.1	evm.model.unitig_0.1278*	succinate dehydrogenase (ubiquinone) flavoprotein subunit	24.5134	26.5336	25.821	34.938	34.3454
EC 1.3.5.1	evm.model.unitig_5.222*	succinate dehydrogenase (ubiquinone) flavoprotein subunit	106.025	80.5352	64.0832	82.0902	49.7971
EC 1.3.5.1	evm.model.unitig_0.912*	succinate dehydrogenase (ubiquinone) flavoprotein subunit	300.998	183.431	129.831	141.769	117.838
EC 4.2.1.2	evm.model.unitig_4.976*	fumarate hydratase	216.899	165.403	133.22	129.303	125.578
		**malate supplementary pathway**					
EC 1.1.1.37	evm.model.unitig_0.152*	malate dehydrogenase	382.681	178.625	166.671	194.694	144.212
EC 1.1.1.37	evm.model.unitig_4.581	malate dehydrogenase	1180.59	937.119	1049.38	1328.52	1438
EC 1.1.1.37	evm.model.unitig_3.674	malate dehydrogenase	0	0	0	0	0
EC 1.1.1.37	evm.model.unitig_0.1439	malate dehydrogenase	0	0.733162	0.69599	1.80872	1.30924
EC 6.4.1.1	evm.model.1.596	pyruvate carboxylase	1072.72	247.724	344.37	407.141	385.564
EC 6.4.1.1	evm.model.unitig_4.358*	pyruvate carboxylase	3.10673	2.89939	1.60237	1.91294	1.46637
		**Alternative aeration**					
EC 1.6.5.9	evm.model.unitig_1.793	alternative NADH-ubiquinone oxidoreductase	22.4626	16.162	23.7158	14.4778	12.7033
	evm.model.unitig_3.600	alternative oxidase	0	0.287791	0	0.27723	0.237374
	evm.model.unitig_3.832	alternative oxidase	29.5249	76.824	27.4381	99.4477	54.1134
		**Glyoxylate cycle**					
EC 2.3.3.9	evm.model.unitig_4.427	malate synthase, glyoxysomal	144.411	152.274	43.8047	93.8427	68.3562
EC 4.1.3.1	evm.model.unitig_2.434	isocitrate lyase	1912.74	277.935	41.5601	47.8565	114.906
EC 4.1.3.1	evm.model.unitig_4.958*	isocitrate lyase	450.798	136.445	121.203	118.647	160.556
		**GABA shunt pathway**					
EC 2.6.1.19	evm.model.unitig_6.167*	4-aminobutyrate aminotransferase	44.1905	92.7175	120.197	163.909	105.917
EC 1.2.1.16	evm.model.1.554	succinate-semialdehyde dehydrogenase (NADP^+^)	32.9799	39.6433	41.2847	62.5501	43.2657
EC 1.2.1.16	evm.model.unitig_2.1270	Succinate-semialdehyde dehydrogenase (NAD(P)^+^)	18.7586	31.3531	37.5994	46.1063	23.1694
EC 1.2.1.16	evm.model.unitig_4.289	aldehyde dehydrogenase family protein	38.071	48.0361	28.8966	40.7511	50.4369
EC 1.2.1.16	evm.model.unitig_5.338	succinate-semialdehyde dehydrogenase (NADP)	19.1193	9.30071	18.9276	47.9931	107.202
EC 1.2.1.16	evm.model.unitig_5.445	Succinate-semialdehyde dehydrogenase (NAD(P)^+^)	4.14887	7.1628	15.265	16.603	9.76361
EC 1.2.1.16	evm.model.unitig_6.87	Glutarate-semialdehyde dehydrogenase	18.0798	36.4844	40.734	41.9151	61.8611
EC 4.1.1.15	evm.model.unitig_0.1336	glutamate decarboxylase	19.2535	8.99677	7.21094	3.60937	5.17597
EC 4.1.1.15	evm.model.unitig_1.1110	glutamate decarboxylase 1	2129.58	2923.29	3713.03	3881.53	4501.63
EC 4.1.1.15	evm.model.unitig_4.236*	glutamate decarboxylase 1	2.6946	2.51931	2.15859	5.39455	3.62821
EC 1.4.1.4	evm.model.1.692	NADP-specific glutamate dehydrogenase	81.1597	58.0026	33.3913	23.0803	29.9846
EC 1.4.1.4	evm.model.unitig_0.768	aminating glutamate dehydrogenases	86.8852	147.377	105.387	250.044	189.577

^*^Above the gene ID means the enzyme was located in mitochondrion.

**Table 3:** Transporters regulated on transcriptional level during citrate fermentation.

Gene ID	Gene function	FPKM-6h	FPKM-12h	FPKM-24h	FPKM-36h	FPKM-48h
evm.model.unitig_3.636	ABC transporter C family member	13.1977	35.3317	58.9041	46.401	37.9172
evm.model.unitig_3.637	Canalicular multispecific organic anion transporter 1	28.5373	87.9929	149.208	97.0309	73.4379
evm.model.unitig_3.640	Canalicular multispecific organic anion transporter 1	20.4793	107.35	223.035	296.068	305.751
evm.model.unitig_0.1084	Canalicular multispecific organic anion transporter 2	8.0014	40.5062	79.4721	84.8434	60.4354
evm.model.unitig_5.1021	Sodium/potassium-transporting ATPase subunit alpha-1	63.2289	130.481	286.188	272.621	353.328
evm.model.1.100	succinate/fumarate transporter	60.1747	7.32488	4.93716	3.10826	7.87887
evm.model.1.1208	Uncharacterized transporter	20.1262	657.514	638.877	605.893	604.348
evm.model.1.31	Probable metabolite transport protein	19.7919	47.7725	103.538	138.268	150.005
evm.model.1.352	plasma membrane fusion protein prm1	4.66831	21.8264	107.014	162.537	109.181
evm.model.1.69	amino acid transporter	26.4711	6.00714	4.01169	3.26287	4.30079
evm.model.1.857	Uncharacterized MFS-type transporter	262.407	35.7379	29.1139	28.839	18.9121
evm.model.unitig_0.1595	Uncharacterized transporter	7.69787	24.2853	42.5223	80.5382	81.6519
evm.model.unitig_0.437	quinate permease	2.52556	23.0903	55.1789	28.4854	22.4314
evm.model.unitig_1.1394	MFS multidrug transporter	1144.22	178.003	114.492	36.4788	28.1839
evm.model.unitig_1.578	Mitochondrial 2-oxodicarboxylate carrier 2	826.306	390.695	405.997	283.533	284.765
evm.model.unitig_2.1188	amino-acid permease inda1	157.845	440.541	549.456	578.762	548.658
evm.model.unitig_2.1237	MFS transporter	16.3143	61.9491	77.3993	77.3265	65.6228
evm.model.unitig_2.1258	Glutathione transporter 1	53.949	4.04985	2.2213	1.01079	0.489433
evm.model.unitig_2.1390	vacuolar calcium ion transporter	252.61	622.261	657.009	600.778	603.315
evm.model.unitig_2.1392	vacuolar calcium ion transporter	32.1809	68.8772	91.3865	94.9701	77.2152
evm.model.unitig_2.45	MFS transporter	38.4455	12.7329	3.27757	1.25471	0.357967
evm.model.unitig_2.532	calcium transporter	53.9308	119.391	153.595	189.557	130.659
evm.model.unitig_2.827	urea active transporter 1	15.9503	43.262	77.2426	53.5096	52.18
evm.model.unitig_2.971	corA family metal ion transporter	7.22867	189.826	347.136	300.912	301.19
evm.model.unitig_3.1075	galactose-proton symport	687.339	254.727	116.009	82.0497	93.8167
evm.model.unitig_3.1191	quinate permease	0	25.5055	36.3913	25.114	34.4627
evm.model.unitig_3.62	ABC transporter	39.1748	78.3932	116.238	125.325	118.615
evm.model.unitig_3.635	Oligopeptide transporter	14.6011	104.855	128.548	199.55	116.732
evm.model.unitig_4.1038	Uncharacterized permease	63.5294	161.174	243.495	213.192	141.02
evm.model.unitig_4.1122	Zinc-regulated transporter 1	26.221	868.686	1163.35	831.676	662.476
evm.model.unitig_4.29	MFS multidrug transporter	28.1368	65.3458	79.5197	108.998	84.1431
evm.model.unitig_4.558	sugar transporter	60.7082	28.922	24.2149	12.3733	23.8319
evm.model.unitig_4.58	zinc-regulated transporter 1	27.4409	86.7798	89.9594	104.495	106.612
evm.model.unitig_4.660	MFS allantoate transporter	9.26185	27.5775	126.026	91.9883	92.9983
evm.model.unitig_4.694	Transport protein particle 20 kDa subunit	71.691	35.4592	31.7326	35.1786	31.681
evm.model.unitig_4.966	copper transporter family protein	89.4315	386.8	247.155	674.724	409.464
evm.model.unitig_5.1028	Oligopeptide transporter	427.735	1076.43	1775.18	1632.03	1598.71
evm.model.unitig_6.240	corA family metal ion transporter	89.6403	201.674	232.638	230.226	243.676
evm.model.unitig_6.447	monocarboxylate transporter	50.1323	135.575	189.269	239.416	254.186
evm.model.unitig_6.486	low affinity iron transporter	3.69287	73.9308	190.217	325.134	557.337
evm.model.unitig_6.549	Purine-cytosine permease fcyB	59.0138	194.498	328.304	284.184	176.873
evm.model.unitig_6.821	MFS toxin efflux pump	49.4076	103.654	164.539	173.255	159.919
evm.model.unitig_6.853	Choline transport protein	3.65766	51.889	48.6358	93.561	47.9792
evm.model.unitig_6.875	MFS transporter	2.1797	23.2597	24.884	45.0643	33.2642
evm.model.unitig_3.110	Hexose transporter	5.8594	29.5108	54.735	74.547	275.829
evm.model.unitig_3.919	OPT oligopeptide transporter family	3.40145	42.7754	192.636	367.982	323.622
