# Supplementary material for: Comparative genomics and transcriptome analysis of Aspergillus niger and metabolic engineering for citrate production
Source: Sci Rep. 2017 Jan 20;7:41040. doi: 10.1038/srep41040 (PMC5247736; doi:10.1038/srep41040)
Supplement: Supplementary Figures and Tables [file srep41040-s1.pdf]

Comparative genomics and transcriptome analysis of *Aspergillus niger* and metabolic engineering for citrate production

Xian Yin[a, b], Hyun-dong Shin[c], Jianghua Li [a, b], Guocheng Du[a, b], Long Liu[a, b§], Jian Chen[a§§]

[a]Key Laboratory of Industrial Biotechnology, Ministry of Education, Jiangnan University, Wuxi 214122, China

[b]Key Laboratory of Carbohydrate Chemistry and Biotechnology, Ministry of Education, Jiangnan University, Wuxi 214122, China

[c]School of Chemical and Biomolecular Engineering, Georgia Institute of Technology, Atlanta, GA 30332, USA

Corresponding authors: [§]Long Liu, Tel.: +86-510-85918312, Fax: +86-510-85918309, E-mail: longliu@jiangnan.edu.cn; [§§]Jian Chen, Tel.: +86-510-85913661, Fax: +86-510-85910799, E-mail: jchen@jiangnan.edu.cn

Figure captions

Figure S1. Venn diagram showed the number of function gene groups for *Aspergillus niger* H915-1 (blue), *A. niger* A1 (green) and *A. niger* L2 (red)

Figure S2. Comparison of gene expression levels by qPCR and transcriptome analysis

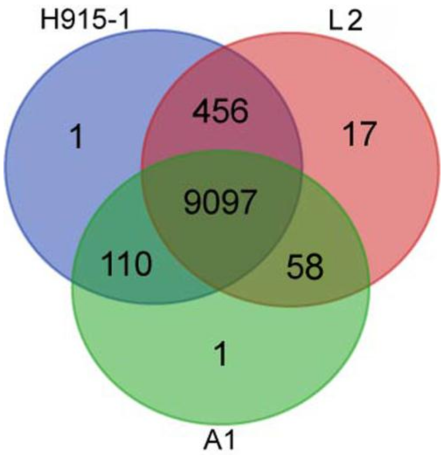

(Figure S1)

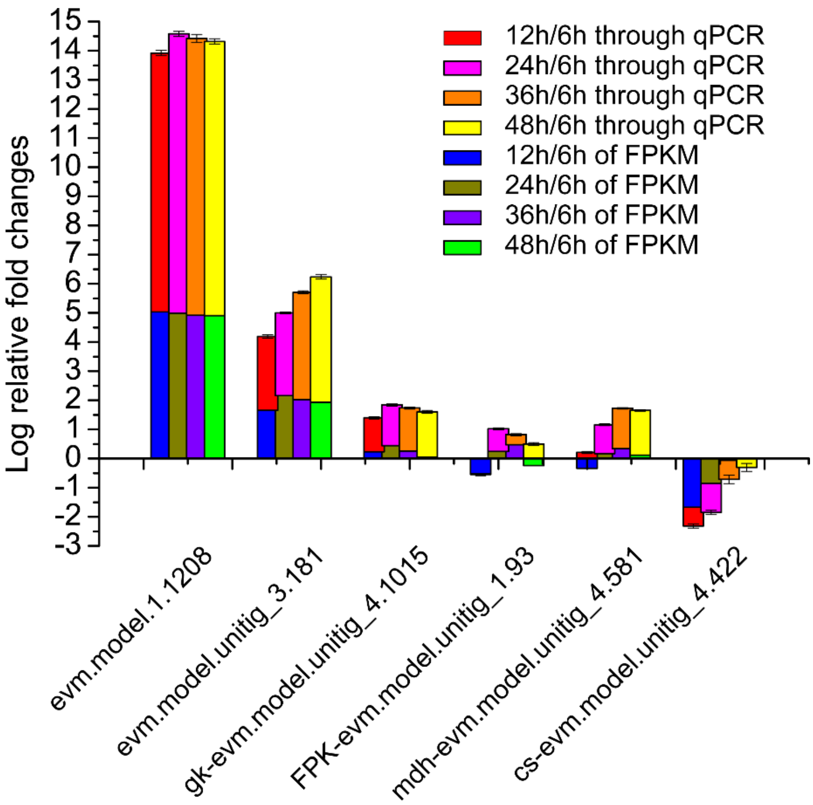
15
14
13
12
11
10
9
8
7
6
5
4
3
2
1
0
-1
-2
-3
Log relative fold changes
12h/6h through qPCR
24h/6h through qPCR
36h/6h through qPCR
48h/6h through qPCR
12h/6h of FPKM
24h/6h of FPKM
36h/6h of FPKM
48h/6h of FPKM
evm.model.1.1208
evm.model.unitig_3.181
gk-evm.model.unitig_4.1015
FPK-evm.model.unitig_1.93
mdh-evm.model.unitig_4.581
cs-evm.model.unitig_4.422

(Figure S2)

Supplementary Table S1. Primers used in this study

Primers	Sequence
oahA5-F	TCTAGATACCAAGGCCAGGCCGCAA
oahA5-R	GTCGACCCGCCAGCAACCAATATTCAT
oahA3-F	ACTAGTACAGAACACCCTGATCGACAGT
oahA3-R	AAGCTTTCTAGACCTGCTACAAATACATTGACCTC
P1	TGTCAACTGAAATCTAACACCCA
P2	CTCAAACAAGCCTCTTTCTCTCC
P3	CACAGGTACACTTGTTTAGAGGTAAT
P4	TCCACCCGAAGGTCACTGATT
**Primers for qPCR**	**Sequence**
Actin-F	CGGAACTTATTGGCTTGG
Actin-R	ACAGAACGATATTGAGGTAGA
evm.model.1.1208-F	GTCAAGGCTATCAGTTCAC
evm.model.1.1208-R	CGGAGGTATATGCGGATA
evm.model.unitig_3.181-F	GAACTGGAACAACTATACG
evm.model.unitig_3.181-R	GGAGTAATTGCTGATGAC
gk-evm.model.unitig_4.1015-F	ACTCCTTTCATTTCCCACCTT
gk-evm.model.unitig_4.1015-R	GAGAGCAGATGACATTATGACTGA
hk-evm.model.unitig_0.782-F	AACTATCCTTCTCTACTACCTCTT
hk-evm.model.unitig_0.782-R	GACGCTTAGGACCGATTC
fpk-evm.model.unitig_1.93-F	CAAACACAAGTTATCTCTG
fpk-evm.model.unitig_1.93-R	AGAAAGAATGGAGTAATGG
gpdph-evm.model.unitig_6.799-F	CTACATCTCCAAGGTTGA
gadph-evm.model.unitig_6.799-R	AATCAGTCTCACTCACAG
pk-evm.model.unitig_0.620-F	GTACCTCGACTACAAGAAC
pk-evm.model.unitig_0.620-R	GAAAGGATACCGTCATCAA
mdh-evm.model.unitig_4.581-F	ATAGGTGATATGCCAATA
mdh-evm.model.unitig_4.581-R	AGAATAACAACGATTAGC
pyc-evm.model.1.596-F	GGTTGGGTTTGGGTTATTTC
pyc-evm.model.1.596-R	ACTTGTCATTCGTATGGTTGA
cs-evm.model.unitig_2.384-F	GTTATCGTTTCGTCATAC
cs-evm.model.unitig_2.384-R	ACCTATCTACTCAAAAGC
cs-evm.model.unitig_4.422-F	AGGCATTGATGGACTTC
cs-evm.model.unitig_4.422-R	AGTTCTTCTTCACCAGTT
cs-evm.model.unitig_5.597-F	TACCGTGTCCTGATATACC
cs-evm.model.unitig_5.597-R	GCGAATGTGAACAACTTG
cs-evm.model.unitig_1.1397-F	TTCGTCTGATGAAGGTATGATAT
cs-evm.model.unitig_1.1397-R	ACGCCATCCTATCCACAT
ATPcs-evm.model.unitig_3.1145-F	TTCCTCTTACCATCCTAA
ATPcs-evm.model.unitig_3.1145-R	GGAGATATAGATAGGTGAG

Supplementary Table S3. COG classification of genomes of *A. niger* H915-1, L2 and A1

function	Gene number		
	H915-1	L2	A1
A: RNA processing and modification	93	92	88
B: Chromatin structure and dynamics	42	44	44
C: Energy production and conversion	279	277	275
D: Cell cycle control, cell division, chromosome partitioning	53	54	54
E: Amino acid transport and metabolism	223	224	221
F: Nucleotide transport and metabolism	43	42	41
G: Carbohydrate transport and metabolism	215	214	212
H: Coenzyme transport and metabolism	78	81	75
I: Lipid transport and metabolism	306	306	303
J: Translation, ribosomal structure and biogenesis	161	164	156
K: Transcription	108	110	107
L: Replication, recombination and repair	74	74	69
M: Cell wall/membrane/envelope biogenesis	68	68	70
N: Cell motility	2	2	2
O: Posttranslational modification, protein turnover, chaperones	275	278	275
P: Inorganic ion transport and metabolism	100	101	100
Q: Secondary metabolites biosynthesis, transport and catabolism	340	339	344
R: Signal transduction mechanisms	191	191	193
S: Intracellular trafficking, secretion, and vesicular transport	132	131	131
T: Defense mechanisms	32	33	32
U: Extracellular structures	2	3	2
V: Nuclear structure	7	7	7
W: Cytoskeleton	60	61	60
X: Function unknown	872	881	864

Supplementary Table S4. Unique or lacked genes in *A. niger* H915-1 genome compared to A1 and L2

gene family ID	gene function	gene ID of H915-1	gene ID of A1	gene ID of L2
Group_0372	hypothetical protein	evm.model.1.1046, evm.model.1.1047, evm.model.1.1048	-	-
Group_9159	unnamed protein product	-	evm.model.scf7180000037711.55	evm.model.unitig_63_quiver.338
Group_9229	hypothetical protein	-	evm.model.scf7180000037876.7	evm.model.unitig_0_quiver.1310
Group_9142	hypothetical protein	-	evm.model.scf7180000037681.24	evm.model.unitig_1_quiver.1075
Group_9277	hypothetical protein	-	evm.model.scf7180000037928.29	evm.model.unitig_0_quiver.932
Group_9274	hypothetical protein	-	evm.model.scf7180000037926.82	evm.model.unitig_63_quiver.638
Group_9124	hypothetical protein	-	evm.model.scf7180000037644.15	evm.model.unitig_1_quiver.15
Group_9126	unnamed protein product	-	evm.model.scf7180000037647.5	evm.model.unitig_63_quiver.534
Group_9206	hypothetical protein	-	evm.model.scf7180000037852.16	evm.model.unitig_1_quiver.827
Group_9264	unnamed protein product	-	evm.model.scf7180000037921.98	evm.model.unitig_63_quiver.831
Group_9249	hypothetical protein	-	evm.model.scf7180000037912.51	evm.model.unitig_0_quiver.1434
Group_9166	hypothetical protein	-	evm.model.scf7180000037719.17	evm.model.unitig_4_quiver.1058
Group_9174	glutathione S-transferase	-	evm.model.scf7180000037730.71	evm.model.unitig_0_quiver.1757
Group_9157	hypothetical protein	-	evm.model.scf7180000037696.21	evm.model.unitig_63_quiver.761
Group_9144	unnamed protein product	-	evm.model.scf7180000037681.52	evm.model.unitig_1_quiver.1103
Group_9231	hypothetical protein	-	evm.model.scf7180000037881.10	evm.model.unitig_3_quiver.724
Group_9189	hypothetical protein	-	evm.model.scf7180000037833.124	evm.model.unitig_1_quiver.293
Group_9182	hypothetical protein	-	evm.model.scf7180000037798.1	evm.model.unitig_5_quiver.788
Group_9283	hypothetical protein	-	evm.model.scf7180000037931.3	evm.model.unitig_0_quiver.63
Group_9287	serine/threonine protein kinase	-	evm.model.scf7180000037934.43	evm.model.unitig_5_quiver.745
Group_9233	hypothetical protein	-	evm.model.scf7180000037887.36	evm.model.unitig_5_quiver.434
Group_9270	hypothetical protein	-	evm.model.scf7180000037924.99	evm.model.unitig_3_quiver.836

gene family ID	gene function	gene ID of H915-1	gene ID of A1	gene ID of L2
Group_9180	hypothetical protein	-	evm.model.scf7180000037743.66	evm.model.unitig_1_quiver.84
Group_9267	hypothetical protein	-	evm.model.scf7180000037922.8	evm.model.unitig_0_quiver.1497
Group_9143	phosphoglycerate mutase family protein	-	evm.model.scf7180000037681.40	evm.model.unitig_1_quiver.1091
Group_9245	cell wall protein	-	evm.model.scf7180000037911.45	evm.model.unitig_0_quiver.477
Group_9225	hypothetical protein	-	evm.model.scf7180000037869.7	evm.model.unitig_0_quiver.1166
Group_9222	hypothetical protein	-	evm.model.scf7180000037867.102	evm.model.unitig_0_quiver.1274
Group_9273	hypothetical protein	-	evm.model.scf7180000037926.65	evm.model.unitig_63_quiver.657
Group_9145	hypothetical protein	-	evm.model.scf7180000037681.85	evm.model.unitig_1_quiver.1136
Group_9247	hypothetical protein	-	evm.model.scf7180000037912.26	evm.model.unitig_0_quiver.1409
Group_9265	hypothetical protein	-	evm.model.scf7180000037922.31	evm.model.unitig_0_quiver.1521
Group_9235	hypothetical protein	-	evm.model.scf7180000037894.19	evm.model.unitig_1_quiver.807
Group_9156	integral membrane protein (Pth11)	-	evm.model.scf7180000037694.23	evm.model.unitig_3_quiver.1184
Group_9121	hypothetical protein	-	evm.model.scf7180000037640.29	evm.model.unitig_0_quiver.1684
Group_9243	hypothetical protein	-	evm.model.scf7180000037908.28	evm.model.unitig_56_quiver.319
Group_9183	hypothetical protein	-	evm.model.scf7180000037798.56	evm.model.unitig_5_quiver.842
Group_9148	hypothetical protein	-	evm.model.scf7180000037683.33	evm.model.unitig_0_quiver.1132
Group_9284	hypothetical protein	-	evm.model.scf7180000037934.128	evm.model.unitig_5_quiver.658
Group_9207	zinc knuckle transcription factor	-	evm.model.scf7180000037855.24	evm.model.unitig_2_quiver.485
Group_9192	unnamed protein product	-	evm.model.scf7180000037833.130	evm.model.unitig_1_quiver.288
Group_9175	alcohol dehydrogenase	-	evm.model.scf7180000037730.87	evm.model.unitig_0_quiver.1772
Group_9127	unnamed protein product	-	evm.model.scf7180000037651.10	evm.model.unitig_0_quiver.178
Group_9181	similar to LOC100382988	-	evm.model.scf7180000037790.2	evm.model.unitig_5_quiver.507
Group_9250	hypothetical protein	-	evm.model.scf7180000037912.81	evm.model.unitig_0_quiver.1466

gene family ID	gene function	gene ID of H915-1	gene ID of A1	gene ID of L2
Group_9240	unnamed protein product	-	evm.model.scf7180000037901.19	evm.model.unitig_63_quiver.866
Group_9160	YCII-related domain protein	-	evm.model.scf7180000037711.90	evm.model.unitig_63_quiver.373
Group_9278	hypothetical protein	-	evm.model.scf7180000037928.40	evm.model.unitig_0_quiver.945
Group_9223	hypothetical protein	-	evm.model.scf7180000037867.74	evm.model.unitig_0_quiver.1246
Group_9152	unnamed protein product	-	evm.model.scf7180000037689.51	evm.model.unitig_0_quiver.555
Group_9149	hypothetical protein	-	evm.model.scf7180000037685.11	evm.model.unitig_1_quiver.1219
Group_9248	unnamed protein product	-	evm.model.scf7180000037912.50	evm.model.unitig_0_quiver.1433
Group_9179	hypothetical protein	-	evm.model.scf7180000037743.38	evm.model.unitig_1_quiver.111
Group_9171	hypothetical protein	-	evm.model.scf7180000037725.12	evm.model.unitig_0_quiver.305
Group_9138	hypothetical protein	-	evm.model.scf7180000037665.31	evm.model.unitig_2_quiver.1075
Group_9251	hypothetical protein	-	evm.model.scf7180000037912.89	evm.model.unitig_0_quiver.1475
Group_9184	hypothetical protein	-	evm.model.scf7180000037830.56	evm.model.unitig_63_quiver.1035
Group_9146	MFS monocarboxylate transporter	-	evm.model.scf7180000037681.86	evm.model.unitig_1_quiver.1137
Group_0374	hypothetical protein	-	evm.model.scf7180000037720.111	evm.model.unitig_55_quiver.21, evm.model.unitig_55_quiver.3

Supplementary Table S5. Variations in genomes of *A. niger* H915-1 from *A. niger* L2 and *A. niger* A1

	SNP/Indel	SV			
		insertion	deleteion	invertion	Tandom duplication
sites of variation	1210	29	6	12	5
variant genes	57	22	2	7	4

Table S6. Gene variations in genomes of *A. niger* L2 and *A. niger* A1 to *A. niger* H915-1 in SNP/indel analysis

contig ID	position	gene ID	Change type	Nucleic acid variation	AA variation	Gene name
1	1129361	evm.model.1.311	missense_variant	Tca/Gca	Ser598Ala	Proline utilization trans-activator
1	1624442	evm.model.1.443	missense_variant	Tgg/Ggg	Trp237Gly	ATP binding L-PSP endoribonuclease family protein
1	1977948	evm.model.1.561	missense_variant	Tcg/Gcg	Ser433Ala	unnamed protein product
1	2561390	evm.model.1.732	frameshift_variant	agc/agcA		branched-chain-amino-acid aminotransferase
1	2561388	evm.model.1.732	frameshift_variant	ccc/ccGc		branched-chain-amino-acid aminotransferase
1	253805	evm.model.1.76	missense_variant	Atc/Ctc	Ile675Leu	hypothetical protein
1	2760046	evm.model.1.766	frameshift_variant	cgg/cggT	Arg80_Gly81	UPD-GlcNAc transporter (Mnn2-2)
unitig_0	4280803	evm.model.unitig_0.1249	disruptive_inframe_deletion	AACACCGCCTCCATGCCGTTGGCGCCCCGAGCGGCCGGATCCCTCGTGGCAGGTGACGATG/A		Fork head protein homolog 2
unitig_0	4410423	evm.model.unitig_0.1291	frameshift_variant	ccc/Accc		hypothetical protein
unitig_0	4410421	evm.model.unitig_0.1291	frameshift_variant+stop_gained	tac/taAc		hypothetical protein
unitig_0	4587807	evm.model.unitig_0.1343	missense_variant	Gat/Tat	Asp225Tyr	histone chaperone asf1 involved in gene silencing
unitig_0	5838066	evm.model.unitig_0.1696	frameshift_variant	G/GT		hypothetical protein
unitig_0	5838061	evm.model.unitig_0.1696	frameshift_variant	ccg/ccgT		hypothetical protein
unitig_0	5905171	evm.model.unitig_0.1717	frameshift_variant	cgatcgtctccggtcg/c		ubiquitin carboxyl-terminal hydrolase

contig ID	position	gene ID	Change type	Nucleic acid variation	AA variation	Gene name
unitig_0	5905170	evm.model.unitig_0.1717	frameshift_variant	gc/g		ubiquitin carboxyl-terminal hydrolase
unitig_0	2100797	evm.model.unitig_0.625	missense_variant	Acc/Ccc	Thr59Pro	Isonitrile hydratase
unitig_0	2267694	evm.model.unitig_0.675	missense_variant	cTc/cCc	Leu140Pro	tRNA-specific adenosine deaminase subunit tad3
unitig_0	2343478	evm.model.unitig_0.703	missense_variant	tCc/tAc	Ser659Tyr	Mitochondrial escape protein 2
unitig_0	2797157	evm.model.unitig_0.807	frameshift_variant	ccc/Accc		cytochrome P450 monooxygenase
unitig_0	3330384	evm.model.unitig_0.983	frameshift_variant	tct/tctG		hypothetical protein
unitig_1	401272	evm.model.unitig_1.103	synonymous_variant	ggA/ggG	Gly492Gly	Amino acid transporters
unitig_1	3555983	evm.model.unitig_1.1041	disruptive_inframe_insertion	acc/aTCAcc		hypothetical protein
unitig_1	3781036	evm.model.unitig_1.1114	missense_variant	Tcc/Ccc	Ser109Pro	hypothetical protein
unitig_1	4076579	evm.model.unitig_1.1195	inframe_insertion	tct/ACTGGAGGCAGCAACGGCtct		hypothetical protein
unitig_1	4736044	evm.model.unitig_1.1385	intron_variant	C/G		C2H2 finger domain protein
unitig_1	4735854	evm.model.unitig_1.1385	intron_variant	C/T		C2H2 finger domain protein
unitig_1	524964	evm.model.unitig_1.140	frameshift_variant	ccc/cAcc		pre-mRNA-splicing factor syf1
unitig_1	2530872	evm.model.unitig_1.745	missense_variant	Agc/Cgc	Ser88Arg	CORD and CS domain protein
unitig_1	2988599	evm.model.unitig_1.873	synonymous_variant	ccA/ccC	Pro97Pro	PBSP domain protein
unitig_1	3290487	evm.model.unitig_1.969	frameshift_variant+start_lost	TGGCCGTTTGTTTGGCCGGCGCCTGCGACTCGAGGATTCGGAACATCGCAACTGGTAGCTCGTAATTTCATGGTCACCGCCGGCC		ER-Golgi vesicle-tethering protein p115

contig ID	position	gene ID	Change type	Nucleic acid variation	AA variation	Gene name
				G/TGGCCG		
unitig_2	4237654	evm.model.unitig_2.1270	missense_variant	caT/caG	His329Gln	succinate-semialdehyde dehydrogenase (NADP(+))
unitig_2	4403439	evm.model.unitig_2.1312	stop_gained+inframe_insertion	tac/tacTAA		Pantothenate transporter liz1
unitig_2	1317528	evm.model.unitig_2.405	missense_variant	Agc/Cgc	Ser68Arg	hypothetical protein
unitig_2	2229288	evm.model.unitig_2.697	missense_variant	aTa/aGa	Ile528Arg	Early growth response protein 1-B
unitig_2	2287481	evm.model.unitig_2.713	synonymous_variant	ccA/ccC	Pro50Pro	hypothetical protein
unitig_2	2287463	evm.model.unitig_2.713	synonymous_variant	ccT/ccC	Pro44Pro	hypothetical protein
unitig_2	3145846	evm.model.unitig_2.963	inframe_deletion	CCTCCTCCTCCTCCT CCTCCTCCTCCTCCT CCTCCTC/C		hypothetical protein
unitig_3	480299	evm.model.unitig_3.153	missense_variant	aTa/aGa	Ile527Arg	Heterokaryon incompatibility protein 6
unitig_3	1470785	evm.model.unitig_3.411	stop_lost+inframe_deletion	Gggagcattggatcatta ttctgcttcgagctag/G		isochorismatase family protein
unitig_3	1824862	evm.model.unitig_3.529	missense_variant	Ccc/Acc	Pro19Thr	sorting nexin-3
unitig_3	2173543	evm.model.unitig_3.624	missense_variant	Atc/Ctc	Ile325Leu	dimethylaniline monooxygenase
unitig_3	2173539	evm.model.unitig_3.624	missense_variant	gGa/gTa	Gly326Val	dimethylaniline monooxygenase
unitig_3	2484191	evm.model.unitig_3.722	missense_variant	aTg/aAg	Met101Lys	hypothetical protein
unitig_3	2731567	evm.model.unitig_3.784	missense_variant	gaT/gaG	Asp465Glu	trimethylguanosine synthase
unitig_4	3605300	evm.model.unitig_4.1030	inframe_deletion	Ggca/G	Ala1390del	Phospholipid-transporting ATPase
unitig_4	97713	evm.model.unitig_4.23	frameshift_variant	gtt/gttT		aconitase family protein
unitig_4	97714	evm.model.unitig_4.23	frameshift_variant	aac/aTac		aconitase family protein
unitig_4	2177645	evm.model.unitig_4.629	intron_variant	A/C		cell pattern formation-associated

contig ID	position	gene ID	Change type	Nucleic acid variation	AA variation	Gene name
						protein stuA
unitig_4	2172575	evm.model.unitig_4.629	intron_variant	atT/at		cell pattern formation-associated protein stuA
unitig_4	2177840	evm.model.unitig_4.629	intron_variant	A/T		cell pattern formation-associated protein stuA
unitig_4	270070	evm.model.unitig_4.77	splice_region_variant	c/Ct		Uncharacterized transcriptional regulatory protein
unitig_4	2808789	evm.model.unitig_4.809	missense_variant	Agc/Cgc	Ser151Arg	Flavin-containing monooxygenase
unitig_4	3229522	evm.model.unitig_4.928	intron_variant	T/A		Alpha-amylase A
unitig_5	496859	evm.model.unitig_5.165	frameshift_variant	ccc/ccAc		mRNA export protein mlo3
unitig_5	496856	evm.model.unitig_5.165	frameshift_variant	gcc/gcAc		mRNA export protein mlo3
unitig_5	496858	evm.model.unitig_5.165	frameshift_variant	ccc/cAcc		mRNA export protein mlo3
unitig_5	1068715	evm.model.unitig_5.347	missense_variant	tTg/tGg	Leu616Trp	hypothetical protein
unitig_5	1521187	evm.model.unitig_5.491	frameshift_variant	gcc/gccA		Cutinase transcription factor 1 alpha
unitig_5	1521189	evm.model.unitig_5.491	frameshift_variant	gcc/gAcc		Cutinase transcription factor 1 alpha
unitig_5	2095316	evm.model.unitig_5.644	frameshift_variant	ccg/ccAg		ubiquitin-conjugating enzyme E2-18 kDa
unitig_5	2095314	evm.model.unitig_5.644	frameshift_variant	ccg/Accg		ubiquitin-conjugating enzyme E2-18 kDa
unitig_5	2171271	evm.model.unitig_5.671	synonymous_variant	ccA/ccC	Pro321Pro	cytokinesis protein 3
unitig_6	1280151	evm.model.unitig_6.311	missense_variant	gAg/gGg	Glu358Gly	Histidine protein methyltransferase 1
unitig_6	2041466	evm.model.unitig_6.537	missense_variant	gAg/gGg	Glu184Gly	hypothetical protein
unitig_6	2828319	evm.model.unitig_6.787	frameshift_variant	cca/Acca		tfdA family oxidoreductase
unitig_6	2964360	evm.model.unitig_6.833	disruptive_inframe_deletion	caacaacag/cag		unnamed protein product

contig ID	position	gene ID	Change type	Nucleic acid variation	AA variation	Gene name
unitig_3	789515	evm.model.unitig_3.236	splice_region_variant	GTA/G		hypothetical protein
unitig_0	2658818	evm.model.unitig_0.772	splice_region_variant	GCATCATC/GCATC		riboflavin kinase
unitig_5	574639	evm.model.unitig_5.194	frameshift_variant	CAG/C		hypothetical protein
unitig_0	5905186	evm.model.unitig_0.1717	frameshift_variant	GATCGTCTCCGGTCT/G		ubiquitin carboxyl-terminal hydrolase
unitig_0	2269725	evm.model.unitig_0.676	inframe_deletion	TGCGGC/TGC		hypothetical protein

Supplementary Table S7. Gene variations in genomes of A. niger L2 and A. niger A1 to A. niger H915-1 in SV analysis

Gene ID of H915-1	Gene function	Variation type	nucleic acid change length(bp)	Variation position with gene
evm.model.unitig_2.1148	unnamed protein product	Tandom duplication	496	in frame
evm.model.unitig_1.848	DNA repair protein (Rad57)	Tandom duplication	5498	in frame
evm.model.unitig_6.117	GRAM domain protein	Tandom duplication	337	in frame
evm.model.unitig_4.408	two-component system protein A	Tandom duplication	811	promoter
evm.model.1.1050	plant senescence-associated protein	Inversion	49412	in frame
evm.model.unitig_2.196	lipin Smp2	Inversion	1007	in frame
evm.model.unitig_2.197	transcription initiation factor TFIID, 31kD subunit	Inversion	1007	promoter
evm.model.unitig_2.314	non-hemolytic phospholipase C precursor	Inversion	688	promoter
evm.model.unitig_2.1102	hypothetical protein	Inversion	104	promoter
evm.model.unitig_1.890	epoxide hydrolase	Inversion	783	in frame
evm.model.unitig_1.891	60S ribosomal protein L5	Inversion	783	in frame
evm.model.1.341	unnamed protein product	Insersion	211	promoter
evm.model.1.382	hypothetical protein	Insersion	220	in frame
evm.model.1.1046	hypothetical protein	Insersion	97	in frame
evm.model.1.1047	hypothetical protein	Insersion	195	in frame
evm.model.1.1048	hypothetical protein	Insersion	105	in frame
evm.model.1.1050	plant senescence-associated protein	Insersion	263	in frame
evm.model.unitig_2.667	hypothetical protein	Insersion	159	in frame
evm.model.unitig_2.743	pre-mRNA-splicing factor cwc22	Insersion	253	promoter
evm.model.unitig_1.73	hypothetical protein	Insersion	258	in frame
evm.model.unitig_1.141	Sec7 domain protein	Insersion	280	in frame
evm.model.unitig_1.788	unnamed protein product	Insersion	85	promoter
evm.model.unitig_1.789	unnamed protein product	Insersion	85	promoter

Gene ID of H915-1	Gene function	Variation type	nucleic acid change length(bp)	Variation position with gene
evm.model.unitig_1.1260	RNA-splicing protein MRS3	Insersion	274	in frame
evm.model.unitig_0.113	mRNA transport regulator (Mtr10)	Insersion	222	in frame
evm.model.unitig_0.311	unnamed protein product	Insersion	277	promoter
evm.model.unitig_0.312	hypothetical protein	Insersion	277	promoter
evm.model.unitig_0.635	ABC transporter (Adp1)	Insersion	201	in frame
evm.model.unitig_0.1140	phosphatidate cytidylyltransferase	Insersion	81	in frame
evm.model.unitig_6.686	calcofluor white hypersensitive protein precursor	Insersion	268	in frame
evm.model.unitig_5.900	hypothetical protein	Insersion	85	in frame
evm.model.unitig_4.405	hypothetical protein	Insersion	277	in frame
evm.model.unitig_4.679	DNA-directed RNA polymerase II subunit RPB9	Insersion	261	in frame
evm.model.unitig_1.969	unnamed protein product	Deletion	80	in frame
evm.model.unitig_5.207	hypothetical protein	Deletion	117	promoter

Supplementary Table S8. Genes involving micro-colony formation

gene ID of H915-1	gene ID of CBS513.88	gene function	GenBank ID	SwissProt ID
evm.model.1.1107	An03g02400	spore-wall fungal hydrophobin dewA	gi|145234977|ref|XP_001390137.1|	sp|P52750|DEWA_EMENI
evm.model.1.1139	An03g01820	polyketide synthase	gi|317028440|ref|XP_001390084.2|	sp|Q12397|STCA_EMENI
evm.model.1.139	An04g08500	hydrophobin	gi|145258896|ref|XP_001402208.1|	sp|P28346|RODL_EMENI
evm.model.1.17	An04g10030	polyketide synthase	gi|317038885|ref|XP_001402359.2|	sp|Q9Y8A5|LNKS_ASPTE
evm.model.1.61	An04g09530	Conidial yellow pigment biosynthesis polyketide synthase	gi|350631780|gb|EHA20150.1|	sp|Q03149|WA_EMENI
evm.model.1.820	An03g06380	polyketide synthase	gi|317028699|ref|XP_001390513.2|	sp|Q4WAZ9|NRP14_ASPFU
evm.model.1.887	An03g05440	Conidial yellow pigment biosynthesis polyketide synthase	gi|134058112|emb|CAK38306.1|	sp|Q03149|WA_EMENI
evm.model.1.909	An03g05140	polyketide synthase	gi|317028624|ref|XP_001390395.2|	sp|Q0C8M3|LNKS_ASPTN
evm.model.unitig_0.1081	An02g10140	Polyketide synthase PksJ	gi|350634928|gb|EHA23290.1|	sp|P40806|PKSJ_BACSU
evm.model.unitig_0.236	An07g03340	hydrophobin	gi|145237540|ref|XP_001391417.1|	sp|P28346|RODL_EMENI
evm.model.unitig_1.406	An08g08480	Granaticin polyketide synthase putative ketoacyl reductase 2	gi|358373461|dbj|GAA90059.1|	sp|P16543|DHK2_STRVN
evm.model.unitig_2.264	An01g11790	nonribosomal peptide synthetase TdiA	gi|145230650|ref|XP_001389589.1|	sp|P40806|PKSJ_BACSU
evm.model.unitig_2.964	An09g01290	Phthiocerol synthesis polyketide synthase type I PpsC	gi|134054733|emb|CAK43573.1|	sp|P96202|PPSC_MYCTU
evm.model.unitig_3.162	An12g02730	polyketide synthase	gi|145246206|ref|XP_001395352.1|	sp|Q4WAZ9|NRP14_ASPFU
evm.model.unitig_3.167	An12g02670	Phthiocerol synthesis polyketide synthase type I PpsC	gi|134080059|emb|CAK41106.1|	sp|P96202|PPSC_MYCTU
evm.model.unitig_3.209	An12g02050	polyketide synthase	gi|317033668|ref|XP_001395291.2|	sp|Q03149|WA_EMENI
evm.model.unitig_3.667	An11g07310	polyketide synthase	gi|145244871|ref|XP_001394705.1|	sp|Q03149|WA_EMENI

gene ID of H915-1	gene ID of CBS513.88	gene function	GenBank ID	SwissProt ID
evm.model.unitig_3.755	An11g05960	polyketide synthase	gi|317032924|ref|XP_001394581.2|	sp|Q9Y8A5|LNKS_ASPTE
evm.model.unitig_3.757	An11g05940	polyketide synthase	gi|145244150|ref|XP_001394579.1|	sp|Q0C8M3|LNKS_ASPTN
evm.model.unitig_3.780	An11g05570	polyketide synthase	gi|317032887|ref|XP_001394543.2|	sp|Q0C8M3|LNKS_ASPTN
evm.model.unitig_3.870	An11g04280	Catalytic activity: polyketide synthases	gi|134079105|emb|CAK40660.1|	sp|Q4WAZ9|NRP14_ASPFU
evm.model.unitig_4.214	An15g05090	polyketide synthase	gi|317035844|ref|XP_001397040.2|	sp|Q4WAZ9|NRP14_ASPFU
evm.model.unitig_4.407	An15g02130	polyketide synthase	gi|145250477|ref|XP_001396752.1|	sp|Q0C8M3|LNKS_ASPTN
evm.model.unitig_4.684	An12g03950	L-aminoadipate-semialdehyde dehydrogenase-phosphopantetheinyl transferase (npgA)	gi|145246440|ref|XP_001395469.1|	sp|Q9CQF6|ADPPT_MOUSE
evm.model.unitig_4.900	An12g07070	polyketide synthase	gi|317033991|ref|XP_001395761.2|	sp|Q9Y8A5|LNKS_ASPTE
evm.model.unitig_4.901	An12g07070	Similarity to polyketide synthase PKS1	gi|134080488|emb|CAK46336.1|	sp|Q4WAZ9|NRP14_ASPFU
evm.model.unitig_5.198	An14g04860	Catalytic activity: polyketide synthases	gi|134081790|emb|CAK42046.1|	sp|Q03131|ERYA1_SACER
evm.model.unitig_5.499	An14g00830	phosphopantetheinyl transferase PptB	gi|317034610|ref|XP_001400712.2|	-
evm.model.unitig_5.672	An09g05730	Conidial yellow pigment biosynthesis polyketide synthase	gi|134078436|emb|CAL00851.1|	sp|Q03149|WA_EMENI
evm.model.unitig_5.720	An09g05110	Polyketide synthase PksJ	gi|134078372|emb|CAK40363.1|	sp|P40806|PKSJ_BACSU
evm.model.unitig_5.950	An09g01860	Conidial yellow pigment biosynthesis polyketide synthase	gi|350639884|gb|EHA28237.1|	sp|Q03149|WA_EMENI

Supplementary Table S9. Regulation of genes involved main heteroacids formation

EC number	gene ID of H915-1	gene ID of CBS513.88	Gene function	FPKM-6h	FPKM-12h	FPKM-24h	FPKM-36h	FPKM-48h
EC 4.1.1.2	evm.model.1.1180	An03g01140	oxalate decarboxylase OxdC	10.0074	514.407	2999.5	2018.14	2832.48
EC 1.1.1.27	evm.model.1.153	An04g08220	L-lactate dehydrogenase	1.83775	0.789	0.192629	0	0.16791
EC 6.2.1.1	evm.model.1.340	An04g05620	acetyl-CoA synthetase	400.783	146.2	183.762	240.954	179.627
EC 1.1.99.1	evm.model.unitig_0.36	An07g00450	Glucose oxidase	792.581	7.59982	5.52318	9.00983	12.9734
EC 3.1.2.1	evm.model.unitig_6.429	An16g07110	acetyl-CoA hydrolase	2299.17	33.862	32.829	16.3067	13.3424
EC 3.7.1.1	evm.model.unitig_6.59	An10g00820	oxaloacetate acetylhydrolase	7968.16	33.4376	4.34472	5.28545	6.43708

Supplementary Table S10. Transcription levels of genes involving cell wall synthesis during citrate fermentation.

gene ID of H915-1	gene ID of CBS 513.88	gene function	abbreviation	FPKM-6h	FPKM-12h	FPKM-24h	FPKM-36h	FPKM-48h
**Gas-family**								
evm.model.unitig_3.549	An02g03070	1,3-beta-glucanosyltransferase	GelC	433.688	266.047	120.248	156.893	114.367
evm.model.unitig_5.1027	An09g00670	1,3-beta-glucanosyltransferase	GelD	347.75	1183.05	1546.35	1636.47	2032.39
evm.model.1.835	An03g06220	1,3-beta-glucanosyltransferase	GelE	7.98677	6.53206	7.28515	5.65486	7.23761
evm.model.unitig_6.497	An16g06120	1,3-beta-glucanosyltransferase	GelF	0	0.142663	0.424798	0.281753	0.251436
evm.model.unitig_6.32	An10g00400	1,3-beta-glucanosyltransferase	GelA	182.41	71.739	8.32015	47.2731	24.2603
evm.model.unitig_1.1003	An08g07350	1,3-beta-glucanosyltransferase	GelB	124.206	108.339	124.296	93.5029	76.1817
evm.model.unitig_0.1147	An02g09050	1,3-beta-glucanosyltransferase	GelG	3.37788	4.13736	4.94468	6.27805	7.90065
**Bgl-family**								
evm.model.unitig_1.737	An08g03580	Glucan 1,3-beta-glucosidase	BgtA	39.9038	53.5506	16.7324	190.818	99.3332
evm.model.1.898	An03g05290	Probable glucan endo-1,3-beta-glucosidase	eglC	67.2244	114.701	85.5947	143.378	88.2422
evm.model.unitig_0.323	An07g04650	Putative glucan endo-1,3-beta-glucosidase	BtgC	29.6338	19.1013	31.2696	14.117	17.8292
evm.model.unitig_1.1272	An06g01530	Probable family 17 glucosidase	Scw	3.04155	1.80463	0.659209	0.883687	0.776761
evm.model.unitig_6.431	An16g07040	Probable beta-glucosidase btgE	BtgE	69.5802	95.1163	104.077	67.5617	63.5846
evm.model.unitig_1.986	An08g07090	SUN domain protein (Adg3)		73.0387	67.3362	105.523	49.8541	74.4551
evm.model.unitig_0.188	An07g02730	SUN domain protein (Uth1)		187.914	322.05	321.125	232.741	228.9
**Crh1-family**								
evm.model.unitig_3.1068	An11g01540	Probable glycosidase	CrhA	36.3163	20.197	39.8707	21.4461	20.5791
evm.model.unitig_2.317	An01g11010	Probable glycosidase	CrhD	17.3047	88.3407	157.803	116.044	130.69
evm.model.unitig_6.721	An16g02850	Probable glycosidase	CrhF	100.793	97.9857	38.6559	44.7124	28.122
evm.model.unitig_4.200	An15g05350	Probable glycosidase	CrhG	11.2157	39.3065	39.1893	40.1699	34.7719

gene ID of H915-1	gene ID of CBS 513.88	gene function	abbreviation	FPKM-6h	FPKM-12h	FPKM-24h	FPKM-36h	FPKM-48h
evm.model.unitig_0.522	An07g07530	Probable glycosidase	CrhB	97.9781	144.602	194.156	150.878	127.798
evm.model.unitig_0.83	An07g01160	Probable glycosidase	CrhC	13.5355	120.163	237.258	116.55	180.128
evm.model.unitig_0.1055	An02g10490	probable glycosidase		40.9125	31.2089	48.6761	49.0684	61.722
evm.model.unitig_2.883	An01g03090	Putative endo-1,3(4)-beta-glucanase	EngA	31.8375	30.7227	33.1075	56.3619	119.447
evm.model.unitig_2.863	An01g03340	Probable endo-beta-1,4-glucanase A		584.567	502.49	115.875	112.818	112.276
evm.model.unitig_0.1757	An02g00850	Probable endo-1,3(4)-beta-glucanase		92.423	103.935	102.81	68.4599	46.2662
evm.model.unitig_5.348	An14g02760	endo-1,4-beta-glucanase precursor		7071.84	1027.03	99.331	169.691	43.2697
evm.model.unitig_4.225	An15g04900	Probable endo-beta-1,4-glucanase		17.0986	2.44082	2.24502	0.604792	0.363236
evm.model.unitig_1.260	An18g04100	Probable glucan 1,3-beta-glucosidase	ExgA	3.16418	1.7518	1.89166	5.14283	7.07359
evm.model.unitig_3.651	An11g07660	Probable glucan 1,3-beta-glucosidase	ExgB	39.2042	26.8141	35.7971	24.4634	24.3788
evm.model.unitig_1.552	An08g01100	Probable glucan 1,3-beta-glucosidase	ExgC	15.4429	36.4156	43.8723	34.9773	51.9825
evm.model.unitig_1.1420	An06g02060	Probable glucan 1,3-beta-glucosidase	ExgD	0	0.204065	0	0	0
evm.model.unitig_2.209	An01g12450	Glucan 1,3-beta-glucosidase	BxgA	344.399	551.739	519.35	526.324	403.968
evm.model.unitig_0.884	An02g13180	Glucan endo-1,3-beta-glucosidase	BgxB	5.10612	9.01723	5.68684	12.8277	28.7822
evm.model.unitig_2.1411	An19g00090	Glucan 1,3-beta-glucosidase	BgxC	0.685964	0.047614	0	0	0
**Ags-family**								
evm.model.1.29	An04g09890	Cell wall alpha-1,3-glucan synthase	AgsA	169.943	282.768	282.946	317.366	285.498

gene ID of H915-1	gene ID of CBS 513.88	gene function	abbreviation	FPKM-6h	FPKM-12h	FPKM-24h	FPKM-36h	FPKM-48h
evm.model.unitig_0.1588	An02g03260	Cell wall alpha-1,3-glucan synthase	AgsD	2.23031	8.82718	3.48992	64.9818	35.9973
evm.model.unitig_5.859	An09g03070	Cell wall alpha-1,3-glucan synthase	AgsE	47.7876	54.046	35.0288	63.1913	35.827
evm.model.unitig_3.182	An12g02450	Cell wall alpha-1,3-glucan synthase	AgsC	24.1429	144.188	172.476	133.579	108.774
evm.model.unitig_4.19	An15g07810	Cell wall alpha-1,3-glucan synthase	AgsB	47.6622	157.271	21.0596	146.674	59.1841
evm.model.unitig_6.619	An16g04200	GTP-binding protein rho2	RhoB	173.942	221.408	234.063	296.498	289.229
**Agt-family**								
evm.model.unitig_3.181	An12g02460	Acid alpha-amylase	agtB	94.2973	297.199	419.9	384.096	358.325
evm.model.unitig_4.20	An15g07800	Alpha-amylase A type-3	agtC	13.2914	44.1562	6.93325	44.1816	14.4295
evm.model.unitig_5.858	An09g03100	Alpha-amylase A type-3	agtA	122.561	70.9253	49.7084	24.9631	23.0356
**Agn-family**								
evm.model.unitig_0.592	An07g08640	Glucan endo-1,3-alpha-glucosidas	AgnB	5.903	7.82034	7.96088	8.96181	12.1774
evm.model.unitig_5.633	An09g06260	Glucan endo-1,3-alpha-glucosidase	AgnC	44.5241	31.5329	34.4453	52.983	41.7625
evm.model.unitig_1.1164	An08g09610	Glucan endo-1,3-alpha-glucosidase	AgnD	394.829	681.203	829.257	633.59	609.324
evm.model.unitig_4.237	An15g04760	Glucan endo-1,3-alpha-glucosidase	AgnE	0	0.381614	0.249933	3.3578	3.16017
evm.model.unitig_3.229	An12g01800	Glucan endo-1,3-alpha-glucosidase	AgnF	0.219113	0.143616	0	0.138753	0.305501
evm.model.unitig_1.1330	An06g00810	Glucan endo-1,3-alpha-glucosidase	AgnG	0.479026	0.266505	0.389786	0.450368	0.857455
evm.model.1.257	An04g06930	Alpha-amylase A type-3		49.5177	125.179	70.9508	114.751	105.761
evm.model.unitig_2.126	An01g13610	Glucan 1,4-alpha-maltohexaosidase		27.7415	32.3324	23.5033	33.0255	29.6689
evm.model.unitig_3.939	An11g03340	Acid alpha-amylase		9406.27	13401.3	9128.24	5542.22	9089.55
**Chitin synthases**								
evm.model.unitig_0.1646	An02g02360	Chitin synthase	CsmA	121.832	215.961	162.438	230.794	197.261
evm.model.unitig_0.1647	An02g02340	Chitin synthase	CsmB	186.867	351.722	269.736	365.92	309.08

gene ID of H915-1	gene ID of CBS 513.88	gene function	abbreviation	FPKM-6h	FPKM-12h	FPKM-24h	FPKM-36h	FPKM-48h
evm.model.unitig_5.922	An09g02290	Chitin synthase	ChsD	57.1857	89.9635	75.9485	64.6078	57.0536
evm.model.unitig_5.789	An09g04010	Chitin synthase	ChsB	280.053	335.388	272.588	292.975	242.53
evm.model.unitig_4.1127	An12g10380	Chitin synthase	ChsE	21.7103	124.166	120.939	170.282	120.051
evm.model.unitig_1.857	An08g05290	Chitin synthase	ChsG	1.16509	1.78908	3.47599	9.31298	14.9153
evm.model.1.822	An03g06360	Chitin synthase	ChsF	0	0	0	0.07609	0
evm.model.unitig_5.512	An14g00660	Chitin synthase	ChsC	55.6076	61.7083	101.074	30.7118	39.0433
evm.model.unitig_0.385	An07g05570	Chitin synthase	ChsA	19.7451	32.3549	40.2954	45.9547	36.6638
evm.model.unitig_1.801	An08g04350	Chitin synthase export chaperone		81.3704	72.3258	141.221	129.138	149.885
evm.model.unitig_5.927	An09g02240	Beta-hexosaminidase		98.8768	200.375	218.569	201.472	396.727
evm.model.unitig_3.394	An11g11050	Chitin synthase regulatory factor		34.1481	59.39	57.0364	75.1117	61.6274
evm.model.unitig_4.975	An12g07840	Glucosamine 6-phosphate N-acetyltransferase		150.507	196.763	146.381	206.745	169.775
evm.model.unitig_4.979	An12g07880	Cell fusion protein cfr1		23.8721	20.7203	28.3961	23.3557	18.4492
evm.model.unitig_4.951	An12g07540	Chitin synthase regulatory factor		46.6925	49.4799	49.9162	49.4965	40.939
evm.model.unitig_3.336	An12g00480	UDP-N-acetylglucosamine pyrophosphorylase		154.738	208.841	181.376	324.571	235.485
**Chitinases**								
evm.model.unitig_5.620	An09g06400	Endochitinase	CtcA	3.91932	2.93238	3.14127	5.3972	3.91676
evm.model.unitig_5.660	An09g05920	Chitinase	CtcB	4.71241	3.90778	4.69047	5.16489	7.70855
evm.model.unitig_0.1325	An02g07020	Endochitinase	CfcA	14.5408	18.3883	23.0106	44.9139	81.1113
evm.model.unitig_1.1124	An08g09030	Endochitinase	CfcB	1.44866	1.04379	0.514962	2.05976	5.11871
evm.model.1.403	An04g04670	Endochitinase	CfcC	42.213	128.615	62.0976	203.941	100.62
evm.model.unitig_2.728	An01g05360	Endochitinase	CfcD	15.4151	19.1235	18.3242	18.5392	25.1596
evm.model.unitig_2.1410	An19g00100	class V chitinase	CfcG	0.163024	0.052542	0.051115	0.050851	0.045011

gene ID of H915-1	gene ID of CBS 513.88	gene function	abbreviation	FPKM-6h	FPKM-12h	FPKM-24h	FPKM-36h	FPKM-48h
evm.model.unitig_0.841	An02g13580	Chitinase	CfcI	3.3609	2.12306	4.6016	4.92571	9.3432
evm.model.unitig_0.1536	An02g03980	Beta-glucan synthesis-associated protein	KslA	20.6661	46.6438	22.2827	36.0561	38.607
evm.model.unitig_4.612	An05g00130	Cell wall synthesis protein	KnlA	90.1949	95.653	97.6268	107.621	98.3554
evm.model.unitig_0.110	An07g01540	Protein rot1	RotA	77.4846	55.8693	104.477	62.237	75.1657
**Dfg-family**								
evm.model.1.1206	An03g00740	Mannan endo-1,6-alpha-mannosidase	DfgB	1.35522	1.95428	2.6843	3.57772	5.54232
evm.model.unitig_5.282	An14g03520	Mannan endo-1,6-alpha-mannosidase	DfgC	93.495	116.532	106.391	105.103	98.1553
evm.model.unitig_2.647	An01g06500	Mannan endo-1,6-alpha-mannosidase	DfgD	4.32931	2.45921	0.853608	1.55632	1.87156
evm.model.unitig_6.356	An16g08090	Mannan endo-1,6-alpha-mannosidase	DfgE	16.3897	35.1257	42.8621	60.157	55.5207
evm.model.unitig_1.1363	An06g00360	Mannan endo-1,6-alpha-mannosidase	DfgF	16.8543	14.191	19.4463	10.9905	14.8966
evm.model.unitig_0.1631	An02g02660	Putative mannan endo-1,6-alpha-mannosidase	DfgG	7.41031	5.3498	2.91568	4.15995	2.58706
evm.model.unitig_3.1087	An11g01240	Mannan endo-1,6-alpha-mannosidase	DfgH	74.8663	62.3018	59.9235	49.4836	26.1839
evm.model.unitig_4.836	An12g05930	Putative mannan endo-1,6-alpha-mannosidase	DfgI	0	0.52315	0.382449	0.628217	0.221378
evm.model.1.48	An04g09650	Putative mannan endo-1,6-alpha-mannosidase		0.94681	0.365253	0.534255	6.97958	16.3193

gene ID of H915-1	gene ID of CBS 513.88	gene function	abbreviation	FPKM-6h	FPKM-12h	FPKM-24h	FPKM-36h	FPKM-48h
evm.model.1.676	An04g01230	Protein ecm33	EcmA	929.988	1203.71	1399.09	1203.41	1268.35
**WSC-family**								
evm.model.unitig_2.422	An01g09460	Cell wall integrity and stress response component 2	WscA	235.559	273.829	290.222	268.634	311.975
evm.model.unitig_0.288	An07g04070	Cell wall integrity and stress response component 4	WscB	111.895	175.154	195.666	238.122	181.34
**Rho-GEFs**								
evm.model.unitig_0.356	An07g05090	Rho1 guanine nucleotide exchange factor	RefA	52.8923	68.5687	68.44	74.6655	69.3745
evm.model.unitig_4.350	An15g02900	Rho1 guanine nucleotide exchange factor	RefB	25.4491	33.3459	35.4138	39.4765	35.9026
evm.model.1.372	An04g05150	Rho guanine nucleotide exchange factor	CefA	26.2798	32.4087	39.2074	41.5341	38.1165
evm.model.unitig_1.1186	An08g10040	rho guanyl nucleotide exchange factor	CefB	0.497884	0.319301	0.84013	0.615691	0.962403
**Rho-GAPs**								
evm.model.unitig_6.40	An10g00490	Rho-GTPase-activating protein	RapA	18.5192	20.8484	26.765	26.3171	24.8437
evm.model.unitig_3.467	An11g10060	Probable Rho-GTPase-activating protein	RapB	36.7498	54.8389	44.0932	60.9663	50.8077
evm.model.unitig_4.343	An15g03040	Rho-type GTPase-activating protein	RapC	47.4675	66.5632	67.4757	70.7548	60.5539
evm.model.unitig_2.1202	An13g01020	Probable Rho-type GTPase-activating protein	RapD	26.01	35.5613	32.579	43.3829	30.0461
evm.model.unitig_2.1189	An13g00850	Rho-GTPase-activating protein	RapE	28.6628	42.3099	40.4563	65.8285	42.576

gene ID of H915-1	gene ID of CBS 513.88	gene function	abbreviation	FPKM-6h	FPKM-12h	FPKM-24h	FPKM-36h	FPKM-48h
evm.model.unitig_1.870	An08g05440	Probable Rho-type GTPase-activating protein	CapA	14.5782	24.1364	24.0061	31.7156	21.2868
evm.model.unitig_1.458	An18g06730	Probable Rho-type GTPase-activating protein	CapB	32.6111	46.9568	52.137	54.2704	45.0441
**Rho-related GTPases**								
evm.model.unitig_5.148	An14g05530	GTP-binding protein	RhoD	30.4013	25.9519	34.4187	18.4885	12.8308
evm.model.unitig_3.469	An11g10030	Ras-related C3 botulinum toxin substrate	RacA	38.7862	71.7136	55.0574	128.527	86.0568
evm.model.unitig_3.513	An11g09620	GTP-binding protein	RhoC	26.4742	33.7911	26.6553	46.7332	33.9395
evm.model.unitig_1.135	An18g02400	Protein kinase C with putative function in CWI signaling	PkcA	51.7412	70.6264	87.8224	82.0698	78.4548
**MAPKKKs**								
evm.model.unitig_0.1339	An02g06830	MAP kinase kinase kinase	BckA	11.3569	19.4852	21.0069	28.0421	21.2434
evm.model.unitig_6.186	An17g01280	MAP kinase kinase kinase	SteK	49.5722	70.2375	66.6273	79.3403	60.3522
evm.model.unitig_1.709	An08g03240	MAP kinase kinase kinase	SskB	50.9855	82.7021	75.2767	87.2121	81.6301
**MAPKKs**								
evm.model.unitig_1.229	An18g03740	MAP kinase kinase	MkkA	53.0267	108.27	108.346	163.971	109.647
evm.model.unitig_3.420	An11g10690	MAP kinase kinase	SteG	6.09445	7.6951	8.00354	9.9322	7.35432
evm.model.unitig_2.316	An01g11080	Protein kinase wis1	PbsB	32.8318	78.593	87.6352	118.403	117.221
**MAPKs**								
evm.model.unitig_2.418	An01g09520	Mitogen-activated protein kinase	MpkA	146.664	130.995	147.38	140.736	119.403
evm.model.unitig_1.1235	An08g10670	Mitogen-activated protein kinase	FusC	111.246	145.531	245.124	256.164	241.179
evm.model.unitig_0.281	An07g03980	Mitogen-activated protein kinase	HogA	8.12914	8.77733	12.3331	7.79599	7.51126

gene ID of H915-1	gene ID of CBS 513.88	gene function	abbreviation	FPKM-6h	FPKM-12h	FPKM-24h	FPKM-36h	FPKM-48h
evm.model.unitig_1.896	An08g05850	Mitogen-activated protein kinase	HogB	141.295	147.167	169.79	181.585	165.733
evm.model.unitig_1.348	An18g05270	Mitogen-activated protein kinase	HogC	1.58693	0.774868	3.92005	6.72042	20.3115
**MADS-BOX transcription factors**								
evm.model.unitig_0.945	An02g12210	Transcription factor RLM1	RlmA	7.26868	15.633	15.411	17.4989	13.7581
evm.model.unitig_4.1026	An12g08730	Transcription factor of morphogenesis	McmA	191.85	147.766	153.792	152.946	136.861
evm.model.1.139	An04g08500	hydrophobin	hypE	0.331532	1.60662	0.309212	0.320809	0.550592
evm.model.unitig_0.236	An07g03340	hydrophobin	hypF	24.6584	16.3423	25.2839	43.4444	55.1751
**spore wall maturation proteins**								
evm.model.1.899	An03g05260	fungal chitosanase		23.2343	19.2568	8.30921	13.1914	6.97961
**Others**								
evm.model.unitig_1.1270	An06g01550	1,3-beta-glucan synthase component	FksA	350.208	541.062	562.486	672.174	542.831
evm.model.unitig_1.409	An18g05980	GTP-binding protein	RhoA	525.453	604.953	574.374	782.461	649.664
evm.model.unitig_5.501	An14g00800	Signaling mucin MSB2	HkrA	38.4008	78.7296	84.9039	92.6592	74.5294
evm.model.unitig_6.244	An17g02120	Glucan synthesis regulatory protein	KnrA	86.9143	95.0952	97.6845	93.8772	75.3459
evm.model.unitig_3.1090	An11g01190	GPI-anchored cell wall protein Pst1		8.19626	33.0153	90.2154	96.7182	109.892
evm.model.unitig_2.210	An01g12440	chitin binding protein		634.108	135.492	118.697	205.541	121.575
evm.model.unitig_4.261	An15g04570	fungal cellulose binding domain protein		109.335	1.77699	1.51897	0.645444	0.189446
evm.model.1.863	An03g05740	Cellulose synthase catalytic subunit [UDP-forming]		3.52841	6.39177	3.83371	5.4007	4.64119

Supplementary Table S11. Citrate production and yield of H915-1 and H915(*⊿oah::hph*)

Strain	Titer (g L^{-1})	Yield (g g^{-1} %)
H915-1	129 ± 1.28	95.78 ± 1.85
H915(*⊿oah::hph*)	130 ± 2.82	97.46 ± 0.79

The results shown were mean ± sd (n=3). Statistical significance was examined by T-test.
