# Supplementary material for: Comparative genomics and transcriptome analysis of Aspergillus niger and metabolic engineering for citrate production
Source: Sci Rep. 2017 Jan 20;7:41040. doi: 10.1038/srep41040 (PMC5247736; doi:10.1038/srep41040)
Supplement: Supplementary Information [file srep41040-s2.pdf]

Comparative genomics and transcriptome analysis of *Aspergillus niger* and metabolic engineering for citrate production

Xian Yin[a, b], Hyun-dong Shin[c], Jianghua Li[a, b], Guocheng Du[a, b], Long Liu[a, b§], Jian Chen[a§§]

[a]Key Laboratory of Industrial Biotechnology, Ministry of Education, Jiangnan University, Wuxi 214122, China

[b]Key Laboratory of Carbohydrate Chemistry and Biotechnology, Ministry of Education, Jiangnan University, Wuxi 214122, China

[c]School of Chemical and Biomolecular Engineering, Georgia Institute of Technology, Atlanta, GA 30332, USA

Corresponding authors: [§]Long Liu, Tel.: +86-510-85918312, Fax: +86-510-85918309, E-mail: longliu@jiangnan.edu.cn; [§§]Jian Chen, Tel.: +86-510-85913661, Fax: +86-510-85910799, E-mail: jchen@jiangnan.edu.cn

Supplementary Table S2. Gene ID matching of H915-1 and CBS513.88

Gene ID of H915-1	Gene ID of 513.88
evm.model.1.1	An04g10300
evm.model.1.10	An04g10110
evm.model.1.100	An04g09030
evm.model.1.1000	An03g03820
evm.model.1.1001	An03g03810
evm.model.1.1002	An03g03800
evm.model.1.1003	An03g03800
evm.model.1.1004	An03g03780
evm.model.1.1005	An03g03770
evm.model.1.1006	An03g03760
evm.model.1.1007	An03g03750
evm.model.1.1008	An03g03740
evm.model.1.1009	An03g03730
evm.model.1.101	An04g09020
evm.model.1.1010	An03g03720
evm.model.1.1011	An03g03710
evm.model.1.1012	An03g03700
evm.model.1.1013	An03g03670
evm.model.1.1014	An03g03660
evm.model.1.1015	An03g03650
evm.model.1.1016	An03g03640
evm.model.1.1017	An03g03620
evm.model.1.1018	An03g03600
evm.model.1.1019	An03g03590
evm.model.1.102	An04g09010
evm.model.1.1020	An03g03580
evm.model.1.1021	An03g03570
evm.model.1.1022	An03g03560
evm.model.1.1023	An03g03550
evm.model.1.1024	An03g03540
evm.model.1.1025	An03g03530
evm.model.1.1026	An03g03520
evm.model.1.1027	An03g03510
evm.model.1.1028	An03g03500
evm.model.1.1029	An03g03490
evm.model.1.103	-
evm.model.1.1030	An03g03480
evm.model.1.1031	An03g03470
evm.model.1.1032	An03g03460
evm.model.1.1033	An03g03450
evm.model.1.1035	An03g03420
evm.model.1.1037	An03g03030

Gene ID of H915-1	Gene ID of 513.88
evm.model.1.1038	An03g03360
evm.model.1.1039	An03g03350
evm.model.1.104	An04g09000
evm.model.1.1040	An03g03330
evm.model.1.1041	An03g03300
evm.model.1.1042	An03g03290
evm.model.1.1043	An03g03270
evm.model.1.1049	-
evm.model.1.105	An04g08990
evm.model.1.1050	-
evm.model.1.1051	An03g03130
evm.model.1.1053	-
evm.model.1.1055	-
evm.model.1.1056	An03g03050
evm.model.1.1057	An03g03030
evm.model.1.1058	An03g03010
evm.model.1.1059	An03g03000
evm.model.1.106	An04g08970
evm.model.1.1060	An03g02990
evm.model.1.1061	An03g02980
evm.model.1.1062	An03g02970
evm.model.1.1063	An03g02960
evm.model.1.1064	An03g02950
evm.model.1.1065	An03g02940
evm.model.1.1066	An03g02930
evm.model.1.1067	An03g02920
evm.model.1.1068	An03g02910
evm.model.1.1069	An03g02890
evm.model.1.107	An15g05530
evm.model.1.1070	-
evm.model.1.1071	An03g02880
evm.model.1.1072	An03g02870
evm.model.1.1073	An03g02850
evm.model.1.1074	An03g02840
evm.model.1.1075	An03g02830
evm.model.1.1076	An03g02810
evm.model.1.1077	An03g02800
evm.model.1.1079	An03g02780
evm.model.1.108	An04g08940
evm.model.1.1080	An03g02770
evm.model.1.1081	An03g02750
evm.model.1.1082	An03g02720
evm.model.1.1083	An03g02700

Gene ID of H915-1	Gene ID of 513.88
evm.model.1.1084	An03g02690
evm.model.1.1085	An03g02680
evm.model.1.1086	An03g02670
evm.model.1.1087	An03g02660
evm.model.1.1088	An03g02650
evm.model.1.1089	An12g04690
evm.model.1.109	An04g08920
evm.model.1.1091	An03g02630
evm.model.1.1092	An03g02620
evm.model.1.1093	An03g02610
evm.model.1.1094	An03g02600
evm.model.1.1095	An03g02580
evm.model.1.1096	An03g02570
evm.model.1.1097	An03g02560
evm.model.1.1098	An03g02550
evm.model.1.1099	An03g02540
evm.model.1.110	An04g08900
evm.model.1.1100	An03g02540
evm.model.1.1101	An03g02530
evm.model.1.1102	An03g02510
evm.model.1.1103	An03g02500
evm.model.1.1104	An03g02490
evm.model.1.1105	An03g02480
evm.model.1.1106	An03g02430
evm.model.1.1107	An03g02400
evm.model.1.1108	An03g02380
evm.model.1.1109	An03g02330
evm.model.1.111	An04g08890
evm.model.1.1110	An03g02320
evm.model.1.1111	An03g02300
evm.model.1.1112	An03g02280
evm.model.1.1113	An03g02270
evm.model.1.1114	An03g02260
evm.model.1.1115	An03g02230
evm.model.1.1116	An03g02190
evm.model.1.1117	An03g02180
evm.model.1.1118	An03g02160
evm.model.1.1119	An03g02130
evm.model.1.112	An04g08860
evm.model.1.1120	An03g02120
evm.model.1.1121	An03g02100
evm.model.1.1122	An03g02090
evm.model.1.1123	An03g02080

Gene ID of H915-1	Gene ID of 513.88
evm.model.1.1124	An03g02070
evm.model.1.1125	An03g02060
evm.model.1.1126	An03g02040
evm.model.1.1127	An03g02000
evm.model.1.1128	An03g01950
evm.model.1.1129	An03g01940
evm.model.1.113	An04g08840
evm.model.1.1130	An03g01900
evm.model.1.1131	An03g01900
evm.model.1.1133	An03g01890
evm.model.1.1134	An03g01880
evm.model.1.1135	An03g01880
evm.model.1.1136	An03g01860
evm.model.1.1137	An03g01850
evm.model.1.1138	An03g01830
evm.model.1.1139	An03g01820
evm.model.1.114	An04g08830
evm.model.1.1140	An03g01810
evm.model.1.1141	An03g01790
evm.model.1.1142	An03g01750
evm.model.1.1143	An03g01710
evm.model.1.1144	An03g01700
evm.model.1.1145	An03g01690
evm.model.1.1146	An03g01660
evm.model.1.1147	An03g01650
evm.model.1.1148	An03g01640
evm.model.1.1149	An03g01620
evm.model.1.115	An04g08810
evm.model.1.1150	An03g01590
evm.model.1.1151	An03g01570
evm.model.1.1152	An03g01560
evm.model.1.1153	An03g01540
evm.model.1.1154	An03g01530
evm.model.1.1155	An03g01520
evm.model.1.1156	An14g01490
evm.model.1.1157	An03g01490
evm.model.1.1158	An03g01480
evm.model.1.1159	An03g01470
evm.model.1.116	An04g08800
evm.model.1.1160	An03g01460
evm.model.1.1161	An03g01450
evm.model.1.1162	An03g01430
evm.model.1.1163	An03g01420

Gene ID of H915-1	Gene ID of 513.88
evm.model.1.1164	An03g01390
evm.model.1.1165	An03g01370
evm.model.1.1166	An03g01360
evm.model.1.1167	An03g01320
evm.model.1.1168	An03g01310
evm.model.1.1169	An03g01300
evm.model.1.117	An04g08790
evm.model.1.1170	An03g01290
evm.model.1.1171	An03g01280
evm.model.1.1172	An03g01270
evm.model.1.1173	An03g01240
evm.model.1.1174	An03g01230
evm.model.1.1175	An03g01200
evm.model.1.1176	An03g01190
evm.model.1.1177	An03g01180
evm.model.1.1178	An03g01170
evm.model.1.1179	An03g01160
evm.model.1.118	An04g08760
evm.model.1.1180	An03g01140
evm.model.1.1181	An03g01130
evm.model.1.1182	An03g01120
evm.model.1.1183	An03g01110
evm.model.1.1184	An03g01090
evm.model.1.1185	An18g00060
evm.model.1.1186	An03g01060
evm.model.1.1187	An03g01050
evm.model.1.1188	An03g01030
evm.model.1.1189	An03g01010
evm.model.1.119	An04g08740
evm.model.1.1190	An03g01000
evm.model.1.1191	An03g00980
evm.model.1.1192	An03g00970
evm.model.1.1193	An03g00960
evm.model.1.1194	An03g00940
evm.model.1.1195	An03g00920
evm.model.1.1196	An03g00910
evm.model.1.1197	An03g00890
evm.model.1.1198	An03g00880
evm.model.1.1199	An03g00870
evm.model.1.12	An04g10090
evm.model.1.120	An04g08740
evm.model.1.1200	An03g00860
evm.model.1.1201	An03g00850

Gene ID of H915-1	Gene ID of 513.88
evm.model.1.1203	An03g00790
evm.model.1.1204	An03g00770
evm.model.1.1205	An03g00750
evm.model.1.1206	An03g00740
evm.model.1.1207	An03g00730
evm.model.1.1208	An03g00680
evm.model.1.1209	An03g00660
evm.model.1.121	An04g08730
evm.model.1.1210	An03g00650
evm.model.1.1211	An03g00640
evm.model.1.1212	An03g00600
evm.model.1.1213	An03g00590
evm.model.1.1214	An03g00580
evm.model.1.1215	An03g00560
evm.model.1.1216	An03g00500
evm.model.1.1217	An03g00490
evm.model.1.1218	An03g00480
evm.model.1.1219	An03g00470
evm.model.1.122	An04g08720
evm.model.1.1220	An03g00460
evm.model.1.1221	An03g00450
evm.model.1.1222	An03g00430
evm.model.1.1223	An03g00420
evm.model.1.1224	An03g00400
evm.model.1.1225	An03g00390
evm.model.1.1226	An03g00370
evm.model.1.1227	An03g00360
evm.model.1.1228	An03g00340
evm.model.1.1229	-
evm.model.1.123	An04g08710
evm.model.1.1230	An03g00320
evm.model.1.1231	An03g00310
evm.model.1.1232	An03g00280
evm.model.1.1234	An03g00250
evm.model.1.1235	An03g00240
evm.model.1.1236	An03g00230
evm.model.1.1238	An03g00200
evm.model.1.1239	An03g00190
evm.model.1.124	An04g08700
evm.model.1.1240	An03g00180
evm.model.1.1241	An03g00170
evm.model.1.1242	An03g00160
evm.model.1.1244	An03g00140

Gene ID of H915-1	Gene ID of 513.88
evm.model.1.1245	An03g00130
evm.model.1.1246	An03g00060
evm.model.1.1247	An03g00040
evm.model.1.1248	An03g00030
evm.model.1.125	An04g08690
evm.model.1.1250	An03g00010
evm.model.1.1251	An03g06480
evm.model.1.1252	An14g04420
evm.model.1.1253	An18g04550
evm.model.1.126	An04g08680
evm.model.1.127	An04g08660
evm.model.1.128	An04g08650
evm.model.1.129	An04g08640
evm.model.1.13	An04g10080
evm.model.1.130	An04g08630
evm.model.1.132	An04g08620
evm.model.1.133	An04g08610
evm.model.1.134	An04g08600
evm.model.1.135	An04g08590
evm.model.1.136	An04g08580
evm.model.1.137	An04g08560
evm.model.1.138	An04g08550
evm.model.1.139	An04g08500
evm.model.1.14	An04g10070
evm.model.1.140	An04g08490
evm.model.1.141	An04g08440
evm.model.1.142	An04g08420
evm.model.1.143	An04g08390
evm.model.1.144	An04g08370
evm.model.1.145	An04g08360
evm.model.1.146	An04g08340
evm.model.1.147	An04g08330
evm.model.1.148	An04g08320
evm.model.1.149	An04g08300
evm.model.1.15	An04g10060
evm.model.1.151	An04g08240
evm.model.1.153	An04g08220
evm.model.1.154	An04g08210
evm.model.1.155	An04g08190
evm.model.1.156	An04g08160
evm.model.1.157	An04g08150
evm.model.1.159	An04g08130
evm.model.1.16	An04g10040

Gene ID of H915-1	Gene ID of 513.88
evm.model.1.160	An04g08120
evm.model.1.161	An04g08110
evm.model.1.162	An04g08100
evm.model.1.163	An04g08090
evm.model.1.164	An04g08080
evm.model.1.165	An04g08070
evm.model.1.166	An04g08050
evm.model.1.167	An04g08040
evm.model.1.168	An04g08030
evm.model.1.169	An04g08020
evm.model.1.17	An04g10030
evm.model.1.171	An08g09960
evm.model.1.173	An12g06860
evm.model.1.174	An08g12110
evm.model.1.175	An08g12110
evm.model.1.176	An03g03030
evm.model.1.177	An08g12200
evm.model.1.178	An13g03520
evm.model.1.18	An04g10020
evm.model.1.180	An03g06600
evm.model.1.181	An08g11160
evm.model.1.183	An02g13500
evm.model.1.187	An04g07800
evm.model.1.189	An04g07720
evm.model.1.19	An04g10010
evm.model.1.190	An12g05680
evm.model.1.191	An04g07800
evm.model.1.192	An04g07680
evm.model.1.193	An09g02020
evm.model.1.198	An08g12250
evm.model.1.2	An04g10290
evm.model.1.20	An04g10000
evm.model.1.204	An04g07540
evm.model.1.205	An04g07530
evm.model.1.206	An04g07510
evm.model.1.207	An04g07500
evm.model.1.208	An04g07490
evm.model.1.209	An04g07480
evm.model.1.21	An04g09990
evm.model.1.210	An04g07470
evm.model.1.212	An04g07460
evm.model.1.213	An04g07440
evm.model.1.214	-

Gene ID of H915-1	Gene ID of 513.88
evm.model.1.215	An04g07430
evm.model.1.216	An04g07410
evm.model.1.217	An04g07400
evm.model.1.218	An04g07380
evm.model.1.219	An04g07370
evm.model.1.22	An01g12090
evm.model.1.220	An04g07340
evm.model.1.221	An04g07330
evm.model.1.222	An04g07320
evm.model.1.224	An04g07280
evm.model.1.225	An04g07270
evm.model.1.226	An04g07260
evm.model.1.227	An04g07250
evm.model.1.228	An04g07240
evm.model.1.229	An04g07230
evm.model.1.23	An01g12090
evm.model.1.230	An04g07220
evm.model.1.231	An04g07210
evm.model.1.232	An04g07200
evm.model.1.233	An04g07190
evm.model.1.234	An04g07180
evm.model.1.235	An04g07170
evm.model.1.236	An04g07160
evm.model.1.237	An04g07130
evm.model.1.238	An04g07120
evm.model.1.239	An04g07110
evm.model.1.24	An04g09960
evm.model.1.240	An04g07100
evm.model.1.241	An04g07090
evm.model.1.242	An04g07080
evm.model.1.243	An04g07070
evm.model.1.244	An04g07060
evm.model.1.245	An04g07050
evm.model.1.246	An04g07040
evm.model.1.247	An04g07030
evm.model.1.248	An04g07020
evm.model.1.249	An04g07010
evm.model.1.25	An04g09940
evm.model.1.250	An04g07000
evm.model.1.251	An04g06990
evm.model.1.252	An04g06980
evm.model.1.253	An04g06970
evm.model.1.254	An04g06960

Gene ID of H915-1	Gene ID of 513.88
evm.model.1.255	An04g06950
evm.model.1.256	An04g06940
evm.model.1.257	An04g06930
evm.model.1.258	An04g06920
evm.model.1.259	An04g06910
evm.model.1.26	An04g09920
evm.model.1.260	An04g06900
evm.model.1.261	An04g06890
evm.model.1.262	An04g06880
evm.model.1.263	An04g06870
evm.model.1.264	An04g06840
evm.model.1.265	An04g06730
evm.model.1.266	An04g06690
evm.model.1.267	An04g06680
evm.model.1.268	An04g06670
evm.model.1.269	An04g06660
evm.model.1.27	An04g09910
evm.model.1.270	An04g06650
evm.model.1.271	An04g06640
evm.model.1.273	An04g06620
evm.model.1.274	An04g06600
evm.model.1.275	An04g06590
evm.model.1.276	An04g06580
evm.model.1.277	An04g06570
evm.model.1.278	An04g06530
evm.model.1.279	An04g06510
evm.model.1.28	An04g09900
evm.model.1.280	An04g06500
evm.model.1.281	An04g06490
evm.model.1.282	An04g06430
evm.model.1.283	An04g06420
evm.model.1.284	An04g06400
evm.model.1.285	An04g06380
evm.model.1.286	An04g06370
evm.model.1.287	An04g06310
evm.model.1.288	An04g06300
evm.model.1.289	An04g06290
evm.model.1.29	An04g09890
evm.model.1.290	An04g06270
evm.model.1.291	An04g06260
evm.model.1.292	An04g06250
evm.model.1.293	An04g06210
evm.model.1.294	An04g06200

Gene ID of H915-1	Gene ID of 513.88
evm.model.1.295	An04g06180
evm.model.1.296	An04g06170
evm.model.1.297	An04g06160
evm.model.1.298	An04g06150
evm.model.1.299	An04g06140
evm.model.1.3	An04g10280
evm.model.1.30	An04g09870
evm.model.1.300	An04g06120
evm.model.1.301	An04g06100
evm.model.1.302	An04g06090
evm.model.1.303	An04g06080
evm.model.1.304	An04g06070
evm.model.1.305	An04g06060
evm.model.1.306	An04g06050
evm.model.1.307	An04g06040
evm.model.1.308	An04g06030
evm.model.1.309	An04g06020
evm.model.1.31	An04g09850
evm.model.1.310	An04g06010
evm.model.1.311	An04g06000
evm.model.1.312	An04g05990
evm.model.1.313	An04g05980
evm.model.1.314	An04g05970
evm.model.1.315	An04g05960
evm.model.1.316	An04g05950
evm.model.1.317	An04g05940
evm.model.1.318	An04g05930
evm.model.1.319	An04g05880
evm.model.1.32	An04g09830
evm.model.1.320	An04g05870
evm.model.1.321	An04g05860
evm.model.1.322	An04g05850
evm.model.1.323	An04g05820
evm.model.1.324	An04g05820
evm.model.1.325	An04g05810
evm.model.1.326	An04g05790
evm.model.1.327	-
evm.model.1.328	An04g05760
evm.model.1.329	An04g05740
evm.model.1.33	An04g09820
evm.model.1.330	An04g05730
evm.model.1.331	An04g05720
evm.model.1.332	An04g05710

Gene ID of H915-1	Gene ID of 513.88
evm.model.1.333	An04g05700
evm.model.1.334	An04g05680
evm.model.1.335	An04g05670
evm.model.1.336	An04g05660
evm.model.1.337	An04g05650
evm.model.1.338	An04g05640
evm.model.1.339	An04g05630
evm.model.1.34	An04g09810
evm.model.1.340	An04g05620
evm.model.1.341	An04g05590
evm.model.1.342	An12g00800
evm.model.1.343	An04g05570
evm.model.1.345	An04g05550
evm.model.1.346	An04g05540
evm.model.1.347	An04g05530
evm.model.1.348	An04g05510
evm.model.1.349	An04g05500
evm.model.1.35	An04g09790
evm.model.1.350	An04g05490
evm.model.1.351	An04g05480
evm.model.1.352	An04g05460
evm.model.1.353	An04g05450
evm.model.1.354	An04g05440
evm.model.1.355	An04g05430
evm.model.1.356	An04g05420
evm.model.1.357	An04g05380
evm.model.1.358	An04g05360
evm.model.1.359	An04g05330
evm.model.1.36	An04g09770
evm.model.1.360	An04g05320
evm.model.1.361	An04g05310
evm.model.1.362	An04g05300
evm.model.1.363	An04g05280
evm.model.1.364	An04g05270
evm.model.1.365	An04g05260
evm.model.1.366	An04g05250
evm.model.1.367	An04g05230
evm.model.1.368	An04g05220
evm.model.1.369	An04g05210
evm.model.1.37	An04g09760
evm.model.1.370	An04g05190
evm.model.1.371	An04g05180
evm.model.1.372	An04g05150

Gene ID of H915-1	Gene ID of 513.88
evm.model.1.373	An04g05130
evm.model.1.374	An04g05120
evm.model.1.375	An04g05110
evm.model.1.376	An04g05100
evm.model.1.377	An04g05060
evm.model.1.378	An04g05000
evm.model.1.379	An04g04990
evm.model.1.38	An04g09750
evm.model.1.380	An04g04980
evm.model.1.381	An04g04970
evm.model.1.382	An04g04950
evm.model.1.383	An04g04940
evm.model.1.384	An04g04890
evm.model.1.385	An04g04880
evm.model.1.386	An04g04870
evm.model.1.387	An04g04860
evm.model.1.389	An04g04840
evm.model.1.39	An04g09740
evm.model.1.391	An04g04820
evm.model.1.392	An04g04810
evm.model.1.393	An04g04790
evm.model.1.394	An04g04770
evm.model.1.395	An04g04750
evm.model.1.396	An04g04740
evm.model.1.397	An04g04730
evm.model.1.398	An04g04720
evm.model.1.399	An04g04710
evm.model.1.4	An04g10270
evm.model.1.40	An04g09730
evm.model.1.400	An04g04700
evm.model.1.401	An04g04690
evm.model.1.402	An04g04680
evm.model.1.403	An04g04670
evm.model.1.404	An04g04640
evm.model.1.405	An04g04640
evm.model.1.406	An04g04630
evm.model.1.407	An04g04620
evm.model.1.408	An04g04590
evm.model.1.409	An04g04580
evm.model.1.41	An04g09720
evm.model.1.410	An04g04540
evm.model.1.411	An04g04510
evm.model.1.412	An04g04480

Gene ID of H915-1	Gene ID of 513.88
evm.model.1.413	An04g04470
evm.model.1.414	An04g04440
evm.model.1.415	An04g04430
evm.model.1.416	An04g04400
evm.model.1.417	An04g04380
evm.model.1.418	An04g04370
evm.model.1.42	-
evm.model.1.420	An04g04340
evm.model.1.421	An04g04340
evm.model.1.422	An04g04330
evm.model.1.423	An04g04320
evm.model.1.424	An04g04280
evm.model.1.425	-
evm.model.1.426	An04g04270
evm.model.1.427	An04g04240
evm.model.1.428	An04g04230
evm.model.1.429	An04g04210
evm.model.1.43	An04g09700
evm.model.1.430	An04g04170
evm.model.1.431	An07g00360
evm.model.1.432	An04g04150
evm.model.1.433	An04g04130
evm.model.1.434	An04g04110
evm.model.1.435	An04g04100
evm.model.1.436	An04g04080
evm.model.1.437	An04g04070
evm.model.1.438	An04g04060
evm.model.1.439	An04g04050
evm.model.1.44	An04g09690
evm.model.1.440	An04g04040
evm.model.1.442	An04g03980
evm.model.1.443	An04g03970
evm.model.1.444	An04g03960
evm.model.1.445	An04g03950
evm.model.1.446	An04g03940
evm.model.1.447	An04g03930
evm.model.1.448	An04g03920
evm.model.1.449	An04g03910
evm.model.1.45	An04g09680
evm.model.1.450	An04g03900
evm.model.1.451	An04g03890
evm.model.1.452	An04g03880
evm.model.1.453	An04g03870

Gene ID of H915-1	Gene ID of 513.88
evm.model.1.454	An04g03860
evm.model.1.455	An04g03850
evm.model.1.456	An04g03840
evm.model.1.457	An04g03830
evm.model.1.458	An04g03820
evm.model.1.459	An04g03810
evm.model.1.46	An04g09670
evm.model.1.460	An04g03800
evm.model.1.461	An04g03790
evm.model.1.462	An04g03780
evm.model.1.463	An04g03770
evm.model.1.464	An04g03750
evm.model.1.466	An04g03720
evm.model.1.467	An04g03710
evm.model.1.468	An04g03700
evm.model.1.469	An04g03690
evm.model.1.47	An04g09660
evm.model.1.470	An04g03680
evm.model.1.471	An04g03650
evm.model.1.472	An04g03640
evm.model.1.473	An04g03620
evm.model.1.474	An04g03610
evm.model.1.475	An04g03600
evm.model.1.476	An04g03590
evm.model.1.477	An04g03580
evm.model.1.478	An04g03570
evm.model.1.479	An04g03530
evm.model.1.48	An04g09650
evm.model.1.480	An04g03500
evm.model.1.481	An04g03495
evm.model.1.482	An04g03490
evm.model.1.483	An04g03460
evm.model.1.484	An04g03440
evm.model.1.485	An04g03420
evm.model.1.486	An04g03400
evm.model.1.487	An04g03380
evm.model.1.488	An04g03360
evm.model.1.489	An04g03350
evm.model.1.49	An04g09640
evm.model.1.490	An04g03340
evm.model.1.491	An04g03330
evm.model.1.492	An04g03320
evm.model.1.493	An04g03310

Gene ID of H915-1	Gene ID of 513.88
evm.model.1.494	An04g03300
evm.model.1.495	An04g03290
evm.model.1.496	An04g03270
evm.model.1.497	An04g03260
evm.model.1.498	An04g03250
evm.model.1.499	An04g03250
evm.model.1.5	An16g02400
evm.model.1.50	An04g09630
evm.model.1.500	An04g03220
evm.model.1.501	An04g03210
evm.model.1.502	An04g03200
evm.model.1.503	An04g03190
evm.model.1.504	An04g03190
evm.model.1.505	An04g03180
evm.model.1.506	An04g03170
evm.model.1.507	An04g03160
evm.model.1.508	An04g03140
evm.model.1.509	An04g03130
evm.model.1.51	An04g09620
evm.model.1.510	An04g03120
evm.model.1.511	An04g03110
evm.model.1.512	An04g03100
evm.model.1.513	An04g03090
evm.model.1.514	An04g03080
evm.model.1.515	An04g03070
evm.model.1.516	An04g03060
evm.model.1.517	An04g03050
evm.model.1.519	An04g03010
evm.model.1.52	An04g09610
evm.model.1.520	An12g05680
evm.model.1.521	An12g02770
evm.model.1.522	An04g02990
evm.model.1.523	An04g02980
evm.model.1.524	An04g02970
evm.model.1.525	An04g02950
evm.model.1.526	An04g02930
evm.model.1.528	An04g02910
evm.model.1.529	An04g02900
evm.model.1.53	An04g09600
evm.model.1.530	An04g02890
evm.model.1.531	An04g02880
evm.model.1.532	-
evm.model.1.533	An04g02850

Gene ID of H915-1	Gene ID of 513.88
evm.model.1.534	An04g02840
evm.model.1.535	An04g02830
evm.model.1.536	An04g02810
evm.model.1.537	An04g02810
evm.model.1.538	An04g02800
evm.model.1.539	An04g02790
evm.model.1.54	-
evm.model.1.540	An04g02780
evm.model.1.541	An04g02770
evm.model.1.542	An04g02760
evm.model.1.543	An08g06050
evm.model.1.544	An04g02730
evm.model.1.545	An04g02720
evm.model.1.546	An04g02710
evm.model.1.547	An04g02700
evm.model.1.548	An04g02690
evm.model.1.549	An04g02670
evm.model.1.55	An04g09580
evm.model.1.550	An04g02660
evm.model.1.551	An04g02650
evm.model.1.552	An04g02640
evm.model.1.553	An04g02620
evm.model.1.554	An04g02610
evm.model.1.555	An04g02600
evm.model.1.556	-
evm.model.1.557	An04g02580
evm.model.1.558	An04g02570
evm.model.1.559	An04g02550
evm.model.1.56	An04g09570
evm.model.1.560	An04g02540
evm.model.1.561	An04g02530
evm.model.1.562	An04g02520
evm.model.1.563	An04g02510
evm.model.1.564	An04g02500
evm.model.1.565	An04g02490
evm.model.1.566	An04g02480
evm.model.1.567	An04g02460
evm.model.1.568	An04g02450
evm.model.1.569	An04g02420
evm.model.1.570	An04g02410
evm.model.1.571	An04g02400
evm.model.1.572	An04g02380
evm.model.1.573	An04g02370

Gene ID of H915-1	Gene ID of 513.88
evm.model.1.574	An04g02360
evm.model.1.576	An04g02340
evm.model.1.577	An04g02320
evm.model.1.578	An04g02320
evm.model.1.579	An04g02310
evm.model.1.58	An04g09560
evm.model.1.580	An04g02300
evm.model.1.581	An04g02280
evm.model.1.582	An04g02270
evm.model.1.583	An04g02240
evm.model.1.584	An04g02230
evm.model.1.585	An04g02220
evm.model.1.586	An04g02210
evm.model.1.587	An04g02200
evm.model.1.588	An04g02190
evm.model.1.589	An04g02170
evm.model.1.59	An04g09550
evm.model.1.590	An04g02160
evm.model.1.591	An04g02150
evm.model.1.592	An04g02130
evm.model.1.593	An04g02120
evm.model.1.594	An04g02110
evm.model.1.595	An04g02100
evm.model.1.596	An04g02090
evm.model.1.597	An04g02070
evm.model.1.598	An04g02060
evm.model.1.599	An04g02050
evm.model.1.6	An08g08410
evm.model.1.60	An04g09540
evm.model.1.600	An04g02040
evm.model.1.601	An04g02030
evm.model.1.602	An04g02020
evm.model.1.603	An04g02010
evm.model.1.604	An04g02000
evm.model.1.605	An04g01990
evm.model.1.606	An04g01980
evm.model.1.607	An04g01970
evm.model.1.608	An04g01960
evm.model.1.609	An04g01950
evm.model.1.61	An04g09530
evm.model.1.610	An04g01940
evm.model.1.611	An04g01930
evm.model.1.612	An04g01920

Gene ID of H915-1	Gene ID of 513.88
evm.model.1.613	An04g01910
evm.model.1.614	An04g01900
evm.model.1.615	An04g01890
evm.model.1.616	An04g01880
evm.model.1.617	An04g01870
evm.model.1.618	An04g01860
evm.model.1.619	An04g01850
evm.model.1.62	An04g09520
evm.model.1.620	An04g01840
evm.model.1.621	An04g01830
evm.model.1.622	-
evm.model.1.623	An04g01810
evm.model.1.624	An04g01800
evm.model.1.625	An04g01790
evm.model.1.626	An04g01780
evm.model.1.627	An04g01770
evm.model.1.628	An04g01760
evm.model.1.629	An04g01750
evm.model.1.63	An04g09510
evm.model.1.630	An04g01740
evm.model.1.631	An04g01730
evm.model.1.632	An04g01720
evm.model.1.633	An04g01710
evm.model.1.634	An04g01700
evm.model.1.635	An04g01690
evm.model.1.636	An04g01680
evm.model.1.637	An04g01670
evm.model.1.638	An04g01660
evm.model.1.639	An04g01650
evm.model.1.64	An12g10140
evm.model.1.640	An04g01640
evm.model.1.641	An04g01630
evm.model.1.642	An04g01620
evm.model.1.643	An04g01610
evm.model.1.644	An04g01600
evm.model.1.645	An04g01590
evm.model.1.646	An04g01580
evm.model.1.647	An04g01570
evm.model.1.648	An04g01550
evm.model.1.649	An04g01540
evm.model.1.65	-
evm.model.1.650	An04g01535
evm.model.1.651	An04g01530

Gene ID of H915-1	Gene ID of 513.88
evm.model.1.652	An04g01520
evm.model.1.653	An04g01500
evm.model.1.654	An04g01480
evm.model.1.655	An04g01470
evm.model.1.656	An04g01460
evm.model.1.657	An04g01450
evm.model.1.658	An04g01440
evm.model.1.659	An04g01430
evm.model.1.66	An04g09480
evm.model.1.660	An04g01420
evm.model.1.661	An04g01410
evm.model.1.663	An04g01390
evm.model.1.664	An04g01370
evm.model.1.665	An04g01360
evm.model.1.666	An04g01340
evm.model.1.668	An04g01330
evm.model.1.669	An04g01320
evm.model.1.67	An04g09440
evm.model.1.670	An04g01310
evm.model.1.671	An04g01300
evm.model.1.672	An04g01290
evm.model.1.673	An04g01280
evm.model.1.674	An04g01260
evm.model.1.675	An04g01250
evm.model.1.676	An04g01230
evm.model.1.677	An04g01210
evm.model.1.678	An04g01190
evm.model.1.679	An04g01170
evm.model.1.68	An16g04830
evm.model.1.680	An04g01160
evm.model.1.681	An04g01150
evm.model.1.682	An04g01140
evm.model.1.683	An04g01130
evm.model.1.684	An04g01120
evm.model.1.685	An04g01110
evm.model.1.686	An04g01100
evm.model.1.687	An04g01080
evm.model.1.689	An11g00670
evm.model.1.69	An04g09420
evm.model.1.690	An04g00960
evm.model.1.691	An04g00980
evm.model.1.692	An04g00990
evm.model.1.694	An04g00930

Gene ID of H915-1	Gene ID of 513.88
evm.model.1.695	An04g00910
evm.model.1.696	An04g00900
evm.model.1.697	An04g00880
evm.model.1.698	An04g00870
evm.model.1.699	An04g00860
evm.model.1.70	An04g09410
evm.model.1.700	An04g00850
evm.model.1.701	An04g00840
evm.model.1.702	An04g00830
evm.model.1.703	An04g00820
evm.model.1.704	An04g00800
evm.model.1.705	An04g00790
evm.model.1.706	An04g00780
evm.model.1.707	An04g00760
evm.model.1.708	An04g00750
evm.model.1.709	An04g00740
evm.model.1.71	An04g09400
evm.model.1.711	An04g00720
evm.model.1.713	An04g00690
evm.model.1.714	An04g00680
evm.model.1.715	An04g00670
evm.model.1.716	An04g00660
evm.model.1.717	An04g00640
evm.model.1.718	-
evm.model.1.719	An04g00630
evm.model.1.72	An04g09390
evm.model.1.720	An04g00610
evm.model.1.721	An04g00600
evm.model.1.722	An04g00590
evm.model.1.723	An04g00580
evm.model.1.724	An04g00570
evm.model.1.725	An04g00560
evm.model.1.726	An04g00530
evm.model.1.728	An04g00510
evm.model.1.729	An04g00500
evm.model.1.73	An04g09380
evm.model.1.730	An04g00490
evm.model.1.731	An04g00480
evm.model.1.732	An04g00430
evm.model.1.733	An04g00420
evm.model.1.734	An04g00410
evm.model.1.735	An04g00390
evm.model.1.736	An04g00370

Gene ID of H915-1	Gene ID of 513.88
evm.model.1.737	An04g00360
evm.model.1.738	An04g00350
evm.model.1.739	An04g00340
evm.model.1.74	An04g09360
evm.model.1.740	An04g00300
evm.model.1.741	An04g00270
evm.model.1.742	An04g00250
evm.model.1.743	An18g03170
evm.model.1.744	An04g00220
evm.model.1.745	An04g00210
evm.model.1.746	An04g00200
evm.model.1.747	An04g00180
evm.model.1.748	An04g00170
evm.model.1.749	An04g00160
evm.model.1.75	An04g09290
evm.model.1.750	An04g00150
evm.model.1.751	An04g00140
evm.model.1.752	An04g00130
evm.model.1.753	An04g00120
evm.model.1.754	An04g00110
evm.model.1.755	An04g00100
evm.model.1.756	An04g00080
evm.model.1.757	An04g00070
evm.model.1.758	An04g00060
evm.model.1.759	An04g00050
evm.model.1.76	An04g09270
evm.model.1.760	An04g00020
evm.model.1.761	An04g00010
evm.model.1.763	An03g06970
evm.model.1.764	An03g06960
evm.model.1.765	An03g06950
evm.model.1.766	An03g06940
evm.model.1.767	An03g06930
evm.model.1.768	An03g06920
evm.model.1.769	An03g06910
evm.model.1.77	An04g09260
evm.model.1.770	An03g06900
evm.model.1.772	An03g06880
evm.model.1.773	An03g06870
evm.model.1.774	An03g06860
evm.model.1.775	An03g06850
evm.model.1.776	An03g06840
evm.model.1.777	An03g06830

Gene ID of H915-1	Gene ID of 513.88
evm.model.1.778	An03g06820
evm.model.1.779	An03g06810
evm.model.1.78	An04g09250
evm.model.1.780	An03g06800
evm.model.1.781	An03g06790
evm.model.1.782	-
evm.model.1.784	An03g06760
evm.model.1.785	An03g06750
evm.model.1.786	An03g06730
evm.model.1.787	An03g06720
evm.model.1.788	An03g06710
evm.model.1.789	-
evm.model.1.79	An04g09230
evm.model.1.790	An03g06700
evm.model.1.791	An03g06690
evm.model.1.792	An03g06670
evm.model.1.793	An03g06660
evm.model.1.794	An03g06650
evm.model.1.795	An03g06630
evm.model.1.796	An03g06620
evm.model.1.798	An03g06600
evm.model.1.799	An03g06590
evm.model.1.8	An02g12900
evm.model.1.80	An04g09220
evm.model.1.800	An03g06580
evm.model.1.801	An03g06580
evm.model.1.802	An03g06570
evm.model.1.803	An03g06560
evm.model.1.804	An03g06550
evm.model.1.805	An03g06540
evm.model.1.806	An03g06530
evm.model.1.807	An03g06510
evm.model.1.808	An03g06500
evm.model.1.809	An03g06490
evm.model.1.81	An08g09960
evm.model.1.810	An03g06480
evm.model.1.811	An03g06470
evm.model.1.812	An03g06460
evm.model.1.813	An03g06450
evm.model.1.814	An03g06440
evm.model.1.815	An03g06430
evm.model.1.816	An03g06420
evm.model.1.817	An03g06410

Gene ID of H915-1	Gene ID of 513.88
evm.model.1.818	An03g06400
evm.model.1.819	An03g06390
evm.model.1.82	An04g09190
evm.model.1.820	An03g06380
evm.model.1.821	An03g06370
evm.model.1.822	An03g06360
evm.model.1.823	An03g06350
evm.model.1.824	An03g06340
evm.model.1.825	An03g06330
evm.model.1.826	An03g06320
evm.model.1.827	An03g06310
evm.model.1.828	An03g06300
evm.model.1.829	An03g06270
evm.model.1.83	An04g09170
evm.model.1.830	An03g06250
evm.model.1.831	An03g06250
evm.model.1.832	An03g06240
evm.model.1.833	An03g06230
evm.model.1.834	An13g01780
evm.model.1.835	An03g06220
evm.model.1.836	An03g06140
evm.model.1.837	An03g06120
evm.model.1.838	An03g06090
evm.model.1.839	An03g06080
evm.model.1.84	An04g09160
evm.model.1.840	An03g06020
evm.model.1.841	An03g06010
evm.model.1.842	An03g06000
evm.model.1.843	An03g05990
evm.model.1.844	An03g05980
evm.model.1.845	An03g05960
evm.model.1.846	An03g05940
evm.model.1.847	An03g05930
evm.model.1.848	An07g05640
evm.model.1.849	An03g05890
evm.model.1.85	An04g09150
evm.model.1.850	An03g05890
evm.model.1.851	An03g05880
evm.model.1.852	-
evm.model.1.853	An03g05870
evm.model.1.854	An03g05860
evm.model.1.855	An03g05850
evm.model.1.856	An03g05840

Gene ID of H915-1	Gene ID of 513.88
evm.model.1.857	An03g05830
evm.model.1.858	An03g05810
evm.model.1.859	An03g05790
evm.model.1.860	An03g05780
evm.model.1.861	An03g05770
evm.model.1.862	An03g05750
evm.model.1.863	An03g05740
evm.model.1.865	An03g05680
evm.model.1.866	An03g05670
evm.model.1.867	An03g05660
evm.model.1.868	An03g05650
evm.model.1.869	An08g11230
evm.model.1.87	An04g09130
evm.model.1.871	An03g05620
evm.model.1.872	An03g05610
evm.model.1.873	An03g05600
evm.model.1.874	An03g05590
evm.model.1.875	An03g05590
evm.model.1.876	An03g05560
evm.model.1.877	An03g05540
evm.model.1.878	An03g05530
evm.model.1.879	An08g10880
evm.model.1.88	An04g09120
evm.model.1.881	An03g05490
evm.model.1.882	An03g05480
evm.model.1.884	An03g05460
evm.model.1.885	An03g05450
evm.model.1.886	-
evm.model.1.887	An03g05440
evm.model.1.888	An03g05430
evm.model.1.889	An03g05420
evm.model.1.89	-
evm.model.1.890	An04g03890
evm.model.1.891	An03g05380
evm.model.1.892	An03g05360
evm.model.1.893	An03g05340
evm.model.1.894	An03g05330
evm.model.1.895	An03g05320
evm.model.1.896	An03g05310
evm.model.1.897	An03g05300
evm.model.1.898	An03g05290
evm.model.1.899	An03g05260
evm.model.1.9	An04g10130

Gene ID of H915-1	Gene ID of 513.88
evm.model.1.90	An04g09100
evm.model.1.900	An03g05250
evm.model.1.901	An03g05240
evm.model.1.902	An03g05220
evm.model.1.903	An03g05210
evm.model.1.904	An03g05200
evm.model.1.905	An03g05190
evm.model.1.906	An03g05170
evm.model.1.907	An03g05160
evm.model.1.908	An03g05150
evm.model.1.909	An03g05140
evm.model.1.91	An04g09090
evm.model.1.910	An03g05130
evm.model.1.911	An03g05120
evm.model.1.912	An03g05110
evm.model.1.913	An03g05100
evm.model.1.914	An03g05090
evm.model.1.915	An03g05080
evm.model.1.916	An03g05070
evm.model.1.917	An03g05060
evm.model.1.918	An03g05050
evm.model.1.919	An03g05030
evm.model.1.92	An04g09080
evm.model.1.920	An03g05020
evm.model.1.921	An03g05010
evm.model.1.922	An03g05000
evm.model.1.923	An03g04990
evm.model.1.924	An03g04970
evm.model.1.925	-
evm.model.1.926	An03g04960
evm.model.1.927	An03g04940
evm.model.1.928	An03g04930
evm.model.1.929	An03g04890
evm.model.1.93	An04g09070
evm.model.1.930	An03g04880
evm.model.1.931	An03g04880
evm.model.1.932	An03g04870
evm.model.1.933	An03g04860
evm.model.1.934	An03g04850
evm.model.1.935	An03g04840
evm.model.1.936	An03g04820
evm.model.1.937	An03g04810
evm.model.1.938	An03g04800

Gene ID of H915-1	Gene ID of 513.88
evm.model.1.939	An03g04790
evm.model.1.94	-
evm.model.1.940	An03g04770
evm.model.1.941	An03g04760
evm.model.1.942	An03g04750
evm.model.1.943	An03g04740
evm.model.1.944	An03g04730
evm.model.1.945	An03g04720
evm.model.1.946	An03g04710
evm.model.1.947	An03g04700
evm.model.1.948	An03g04690
evm.model.1.949	An03g04680
evm.model.1.95	-
evm.model.1.950	An03g04670
evm.model.1.951	An03g04660
evm.model.1.952	An03g04650
evm.model.1.953	An03g04610
evm.model.1.954	An03g04600
evm.model.1.955	An03g04590
evm.model.1.956	An03g04570
evm.model.1.957	An03g04570
evm.model.1.958	An03g04560
evm.model.1.959	An03g04550
evm.model.1.96	An04g09065
evm.model.1.960	An03g04530
evm.model.1.961	An03g04520
evm.model.1.962	An03g04510
evm.model.1.963	An03g04500
evm.model.1.964	An03g04430
evm.model.1.965	An03g04410
evm.model.1.966	An03g04400
evm.model.1.967	An03g04390
evm.model.1.968	-
evm.model.1.969	An03g04380
evm.model.1.97	An04g09062
evm.model.1.970	An03g04360
evm.model.1.971	An03g04350
evm.model.1.972	An03g04340
evm.model.1.973	An03g04330
evm.model.1.974	An03g04320
evm.model.1.975	An03g04310
evm.model.1.976	An03g04280
evm.model.1.977	An03g04250

Gene ID of H915-1	Gene ID of 513.88
evm.model.1.978	An03g04250
evm.model.1.979	An03g04230
evm.model.1.98	An04g09060
evm.model.1.980	An03g04210
evm.model.1.981	An03g04190
evm.model.1.982	An03g04180
evm.model.1.983	An03g04140
evm.model.1.984	An03g04130
evm.model.1.985	An03g04120
evm.model.1.986	An03g04110
evm.model.1.987	An03g04090
evm.model.1.988	An03g04080
evm.model.1.989	An03g04060
evm.model.1.99	An04g09050
evm.model.1.990	An03g03960
evm.model.1.991	An03g03940
evm.model.1.992	An03g03930
evm.model.1.993	An03g03900
evm.model.1.994	An03g03890
evm.model.1.995	An03g03870
evm.model.1.996	An03g03860
evm.model.1.997	An03g03850
evm.model.1.998	An03g03840
evm.model.1.999	An03g03830
evm.model.unitig_0.1	-
evm.model.unitig_0.10	An07g00060
evm.model.unitig_0.100	An07g01410
evm.model.unitig_0.1000	An02g11330
evm.model.unitig_0.1001	An02g11320
evm.model.unitig_0.1002	An02g11300
evm.model.unitig_0.1003	An02g11290
evm.model.unitig_0.1004	An02g11280
evm.model.unitig_0.1005	An02g11270
evm.model.unitig_0.1006	An02g11260
evm.model.unitig_0.1007	An02g11230
evm.model.unitig_0.1008	An02g11210
evm.model.unitig_0.1009	An02g11200
evm.model.unitig_0.101	An07g01420
evm.model.unitig_0.1010	An02g11190
evm.model.unitig_0.1011	An02g11180
evm.model.unitig_0.1012	An02g11180
evm.model.unitig_0.1013	An02g11170
evm.model.unitig_0.1014	An02g11160

Gene ID of H915-1	Gene ID of 513.88
evm.model.unitig_0.1015	An02g11150
evm.model.unitig_0.1016	An02g11140
evm.model.unitig_0.1017	An02g11120
evm.model.unitig_0.1018	An02g11090
evm.model.unitig_0.1019	An02g11080
evm.model.unitig_0.102	An07g01430
evm.model.unitig_0.1020	An02g11070
evm.model.unitig_0.1021	An02g11060
evm.model.unitig_0.1022	An02g11040
evm.model.unitig_0.1023	An02g11030
evm.model.unitig_0.1024	An02g11000
evm.model.unitig_0.1025	An02g10690
evm.model.unitig_0.1026	An02g10980
evm.model.unitig_0.1027	An02g10970
evm.model.unitig_0.1028	An02g10940
evm.model.unitig_0.1029	An02g10930
evm.model.unitig_0.103	An07g01440
evm.model.unitig_0.1030	An02g10920
evm.model.unitig_0.1031	An02g10900
evm.model.unitig_0.1032	An02g10890
evm.model.unitig_0.1033	An02g10870
evm.model.unitig_0.1034	An07g02880
evm.model.unitig_0.1035	An02g10840
evm.model.unitig_0.1036	An02g10830
evm.model.unitig_0.1037	An02g10810
evm.model.unitig_0.1038	An02g10790
evm.model.unitig_0.1039	An02g10780
evm.model.unitig_0.104	An07g01450
evm.model.unitig_0.1040	An02g10760
evm.model.unitig_0.1041	An02g10750
evm.model.unitig_0.1042	An02g10740
evm.model.unitig_0.1043	An02g10730
evm.model.unitig_0.1044	An02g10710
evm.model.unitig_0.1045	An02g10700
evm.model.unitig_0.1046	An02g10690
evm.model.unitig_0.1047	An02g10680
evm.model.unitig_0.1048	An02g10670
evm.model.unitig_0.1049	An02g10660
evm.model.unitig_0.105	An07g01470
evm.model.unitig_0.1050	An02g10620
evm.model.unitig_0.1051	An02g10610
evm.model.unitig_0.1052	An02g10610
evm.model.unitig_0.1053	An02g10580

Gene ID of H915-1	Gene ID of 513.88
evm.model.unitig_0.1054	An02g10550
evm.model.unitig_0.1055	An02g10490
evm.model.unitig_0.1056	An02g10480
evm.model.unitig_0.1057	An02g10470
evm.model.unitig_0.1058	An02g10460
evm.model.unitig_0.1059	An02g10450
evm.model.unitig_0.106	An07g01480
evm.model.unitig_0.1060	An02g10440
evm.model.unitig_0.1061	An02g10400
evm.model.unitig_0.1062	An02g10390
evm.model.unitig_0.1063	An02g10370
evm.model.unitig_0.1064	An02g10360
evm.model.unitig_0.1065	An02g10350
evm.model.unitig_0.1066	An02g10340
evm.model.unitig_0.1067	An02g10330
evm.model.unitig_0.1068	An02g10320
evm.model.unitig_0.1069	An02g10310
evm.model.unitig_0.107	An07g01510
evm.model.unitig_0.1070	An02g10270
evm.model.unitig_0.1071	An02g10260
evm.model.unitig_0.1072	An02g10230
evm.model.unitig_0.1073	An02g10220
evm.model.unitig_0.1074	An02g10210
evm.model.unitig_0.1075	An02g10200
evm.model.unitig_0.1076	An02g10190
evm.model.unitig_0.1077	An02g10180
evm.model.unitig_0.1078	An02g10170
evm.model.unitig_0.1079	An02g10160
evm.model.unitig_0.108	An07g01520
evm.model.unitig_0.1080	An02g10150
evm.model.unitig_0.1081	An02g10140
evm.model.unitig_0.1082	An02g10120
evm.model.unitig_0.1084	An02g10110
evm.model.unitig_0.1085	An02g10100
evm.model.unitig_0.1086	An02g10090
evm.model.unitig_0.1087	An02g10080
evm.model.unitig_0.1088	An02g10050
evm.model.unitig_0.1089	An02g10040
evm.model.unitig_0.109	An07g01530
evm.model.unitig_0.1090	An02g10030
evm.model.unitig_0.1092	An18g03810
evm.model.unitig_0.1093	An02g10000
evm.model.unitig_0.1094	An02g09990

Gene ID of H915-1	Gene ID of 513.88
evm.model.unitig_0.1095	An02g09970
evm.model.unitig_0.1096	An02g09960
evm.model.unitig_0.1097	An02g09950
evm.model.unitig_0.1098	An02g09940
evm.model.unitig_0.1099	An02g09930
evm.model.unitig_0.11	An07g00080
evm.model.unitig_0.110	An07g01540
evm.model.unitig_0.1100	An02g09910
evm.model.unitig_0.1101	An02g09890
evm.model.unitig_0.1102	An02g09830
evm.model.unitig_0.1103	An02g09810
evm.model.unitig_0.1104	An02g09790
evm.model.unitig_0.1105	An02g09780
evm.model.unitig_0.1106	An02g09740
evm.model.unitig_0.1107	An02g09730
evm.model.unitig_0.1108	An02g09720
evm.model.unitig_0.1109	An02g09710
evm.model.unitig_0.111	An07g01550
evm.model.unitig_0.1110	An02g09690
evm.model.unitig_0.1111	An02g09610
evm.model.unitig_0.1112	An02g09560
evm.model.unitig_0.1113	An02g09550
evm.model.unitig_0.1114	An02g09540
evm.model.unitig_0.1115	An02g09470
evm.model.unitig_0.1116	An02g09460
evm.model.unitig_0.1117	An02g09440
evm.model.unitig_0.1118	An02g09430
evm.model.unitig_0.1119	An02g09420
evm.model.unitig_0.112	An07g01560
evm.model.unitig_0.1120	An02g09390
evm.model.unitig_0.1121	An02g09370
evm.model.unitig_0.1122	An02g09350
evm.model.unitig_0.1123	An02g09340
evm.model.unitig_0.1124	An02g09330
evm.model.unitig_0.1125	An02g09310
evm.model.unitig_0.1126	An02g09300
evm.model.unitig_0.1127	An02g09290
evm.model.unitig_0.1128	An02g09280
evm.model.unitig_0.1129	-
evm.model.unitig_0.113	An07g01580
evm.model.unitig_0.1130	An02g09270
evm.model.unitig_0.1131	An02g09260
evm.model.unitig_0.1132	An02g09250

Gene ID of H915-1	Gene ID of 513.88
evm.model.unitig_0.1133	An02g09240
evm.model.unitig_0.1134	An02g09220
evm.model.unitig_0.1135	An02g09210
evm.model.unitig_0.1136	An02g09200
evm.model.unitig_0.1137	An02g09190
evm.model.unitig_0.1138	An02g09180
evm.model.unitig_0.1139	An02g09170
evm.model.unitig_0.114	An07g01620
evm.model.unitig_0.1140	An02g09160
evm.model.unitig_0.1141	An02g09150
evm.model.unitig_0.1142	An02g09100
evm.model.unitig_0.1143	An02g09090
evm.model.unitig_0.1144	An02g09080
evm.model.unitig_0.1145	An02g09070
evm.model.unitig_0.1146	An02g09060
evm.model.unitig_0.1147	An02g09050
evm.model.unitig_0.1148	An02g09040
evm.model.unitig_0.1149	An02g09030
evm.model.unitig_0.115	An07g01640
evm.model.unitig_0.1150	An02g09020
evm.model.unitig_0.1151	An02g09010
evm.model.unitig_0.1152	An02g09000
evm.model.unitig_0.1153	An02g08990
evm.model.unitig_0.1154	An02g08980
evm.model.unitig_0.1155	An02g08970
evm.model.unitig_0.1156	An02g08950
evm.model.unitig_0.1157	An02g08940
evm.model.unitig_0.1158	An02g08930
evm.model.unitig_0.1159	An02g08920
evm.model.unitig_0.116	An07g01650
evm.model.unitig_0.1160	-
evm.model.unitig_0.1161	An02g08910
evm.model.unitig_0.1162	An02g08900
evm.model.unitig_0.1163	An02g08890
evm.model.unitig_0.1164	-
evm.model.unitig_0.1165	An02g08870
evm.model.unitig_0.1166	An02g08860
evm.model.unitig_0.1167	An02g08850
evm.model.unitig_0.1168	An02g08845
evm.model.unitig_0.1169	An02g08840
evm.model.unitig_0.117	An07g01670
evm.model.unitig_0.1170	An02g08830
evm.model.unitig_0.1171	An02g08820

Gene ID of H915-1	Gene ID of 513.88
evm.model.unitig_0.1172	An02g08800
evm.model.unitig_0.1173	An02g08790
evm.model.unitig_0.1174	An02g08780
evm.model.unitig_0.1175	An02g08770
evm.model.unitig_0.1176	An02g08760
evm.model.unitig_0.1177	An02g08750
evm.model.unitig_0.1178	An02g08740
evm.model.unitig_0.1179	An02g08730
evm.model.unitig_0.118	An07g01680
evm.model.unitig_0.1180	An02g08720
evm.model.unitig_0.1181	An02g08700
evm.model.unitig_0.1182	An02g08690
evm.model.unitig_0.1183	An02g08680
evm.model.unitig_0.1184	An02g08670
evm.model.unitig_0.1185	An02g08660
evm.model.unitig_0.1186	An02g08640
evm.model.unitig_0.1187	An02g08630
evm.model.unitig_0.1188	An02g08620
evm.model.unitig_0.1189	An02g08610
evm.model.unitig_0.119	An07g01690
evm.model.unitig_0.1190	An02g08600
evm.model.unitig_0.1191	An02g08590
evm.model.unitig_0.1193	An02g08570
evm.model.unitig_0.1194	An02g08560
evm.model.unitig_0.1195	An04g04570
evm.model.unitig_0.1196	An02g08530
evm.model.unitig_0.1197	An02g08520
evm.model.unitig_0.1198	An02g08510
evm.model.unitig_0.1199	An02g08490
evm.model.unitig_0.12	An07g00090
evm.model.unitig_0.120	An07g01700
evm.model.unitig_0.1200	An02g08470
evm.model.unitig_0.1202	An02g08460
evm.model.unitig_0.1203	An02g08450
evm.model.unitig_0.1204	An02g08440
evm.model.unitig_0.1205	An02g08430
evm.model.unitig_0.1206	An02g08420
evm.model.unitig_0.1207	An02g08410
evm.model.unitig_0.1208	An02g08400
evm.model.unitig_0.1209	An02g08390
evm.model.unitig_0.121	An07g01710
evm.model.unitig_0.1210	An02g08380
evm.model.unitig_0.1211	An02g08370

Gene ID of H915-1	Gene ID of 513.88
evm.model.unitig_0.1212	An02g08350
evm.model.unitig_0.1213	An02g08340
evm.model.unitig_0.1214	An02g08330
evm.model.unitig_0.1215	An02g08320
evm.model.unitig_0.1216	An02g08310
evm.model.unitig_0.1217	An02g08290
evm.model.unitig_0.1218	An02g08000
evm.model.unitig_0.1219	An02g08010
evm.model.unitig_0.122	An03g06510
evm.model.unitig_0.1220	An02g08020
evm.model.unitig_0.1222	An02g08040
evm.model.unitig_0.1223	An02g08050
evm.model.unitig_0.1224	An02g08060
evm.model.unitig_0.1225	An02g08070
evm.model.unitig_0.1226	An02g08080
evm.model.unitig_0.1227	An02g08090
evm.model.unitig_0.1228	An02g08100
evm.model.unitig_0.1229	An02g08110
evm.model.unitig_0.123	An07g01730
evm.model.unitig_0.1230	An02g08120
evm.model.unitig_0.1231	An02g08130
evm.model.unitig_0.1232	An02g08140
evm.model.unitig_0.1233	An02g08160
evm.model.unitig_0.1234	An02g08170
evm.model.unitig_0.1235	An02g08180
evm.model.unitig_0.1236	An02g08200
evm.model.unitig_0.1237	An02g08210
evm.model.unitig_0.1238	An02g08220
evm.model.unitig_0.1239	An02g08250
evm.model.unitig_0.124	An07g01780
evm.model.unitig_0.1240	An02g08260
evm.model.unitig_0.1241	An02g08270
evm.model.unitig_0.1242	An02g07980
evm.model.unitig_0.1243	An02g07960
evm.model.unitig_0.1244	An02g07950
evm.model.unitig_0.1245	An02g07940
evm.model.unitig_0.1246	An02g07930
evm.model.unitig_0.1248	An02g07910
evm.model.unitig_0.1249	An02g07900
evm.model.unitig_0.1250	An02g07890
evm.model.unitig_0.1251	An02g07880
evm.model.unitig_0.1252	An02g07870
evm.model.unitig_0.1253	An02g07860

Gene ID of H915-1	Gene ID of 513.88
evm.model.unitig_0.1254	An02g07850
evm.model.unitig_0.1255	An02g07840
evm.model.unitig_0.1256	An02g07830
evm.model.unitig_0.1257	-
evm.model.unitig_0.1258	An02g07820
evm.model.unitig_0.1259	An02g07800
evm.model.unitig_0.1260	An02g07780
evm.model.unitig_0.1261	An02g07770
evm.model.unitig_0.1262	-
evm.model.unitig_0.1263	An02g07760
evm.model.unitig_0.1264	An02g07750
evm.model.unitig_0.1265	An02g07740
evm.model.unitig_0.1266	An02g07730
evm.model.unitig_0.1267	-
evm.model.unitig_0.1268	An02g07720
evm.model.unitig_0.1269	An02g07710
evm.model.unitig_0.127	An18g06220
evm.model.unitig_0.1270	An02g07700
evm.model.unitig_0.1271	An02g07690
evm.model.unitig_0.1272	An02g07660
evm.model.unitig_0.1273	An02g07650
evm.model.unitig_0.1274	-
evm.model.unitig_0.1275	An02g07640
evm.model.unitig_0.1276	An04g00180
evm.model.unitig_0.1277	An02g07610
evm.model.unitig_0.1278	An02g07590
evm.model.unitig_0.1279	An02g07580
evm.model.unitig_0.1280	An02g07570
evm.model.unitig_0.1281	An02g07560
evm.model.unitig_0.1282	An02g07550
evm.model.unitig_0.1283	An02g07530
evm.model.unitig_0.1284	An02g07520
evm.model.unitig_0.1285	An02g07510
evm.model.unitig_0.1286	An02g07500
evm.model.unitig_0.1287	An02g07490
evm.model.unitig_0.1288	An02g07480
evm.model.unitig_0.1289	An02g07470
evm.model.unitig_0.129	An07g01810
evm.model.unitig_0.1290	An02g07460
evm.model.unitig_0.1291	An02g07440
evm.model.unitig_0.1292	An02g07430
evm.model.unitig_0.1293	An02g07420
evm.model.unitig_0.1294	An02g07410

Gene ID of H915-1	Gene ID of 513.88
evm.model.unitig_0.1295	An02g07400
evm.model.unitig_0.1296	An02g07390
evm.model.unitig_0.1297	An02g07380
evm.model.unitig_0.1298	An02g07370
evm.model.unitig_0.1299	An02g07360
evm.model.unitig_0.13	An07g00100
evm.model.unitig_0.130	-
evm.model.unitig_0.1300	An02g07350
evm.model.unitig_0.1301	An02g07340
evm.model.unitig_0.1302	An02g07330
evm.model.unitig_0.1303	An02g07320
evm.model.unitig_0.1304	An02g07310
evm.model.unitig_0.1305	An02g07300
evm.model.unitig_0.1306	An02g07290
evm.model.unitig_0.1307	An02g07260
evm.model.unitig_0.1308	An02g07250
evm.model.unitig_0.1309	An02g07240
evm.model.unitig_0.131	An07g01830
evm.model.unitig_0.1310	An02g07230
evm.model.unitig_0.1311	An02g07210
evm.model.unitig_0.1312	An02g07190
evm.model.unitig_0.1313	An02g07170
evm.model.unitig_0.1314	An02g07160
evm.model.unitig_0.1315	An02g07140
evm.model.unitig_0.1316	An02g07130
evm.model.unitig_0.1317	An02g07120
evm.model.unitig_0.1318	An02g07090
evm.model.unitig_0.1319	An02g07080
evm.model.unitig_0.132	An07g01850
evm.model.unitig_0.1320	An02g07070
evm.model.unitig_0.1321	An02g07060
evm.model.unitig_0.1322	An02g07050
evm.model.unitig_0.1323	An02g07040
evm.model.unitig_0.1324	An02g07030
evm.model.unitig_0.1325	An02g07020
evm.model.unitig_0.1326	An02g07010
evm.model.unitig_0.1327	An02g07000
evm.model.unitig_0.1328	An02g06980
evm.model.unitig_0.1329	An02g06970
evm.model.unitig_0.133	An07g01870
evm.model.unitig_0.1330	An02g06960
evm.model.unitig_0.1331	An02g06940
evm.model.unitig_0.1332	An02g06910

Gene ID of H915-1	Gene ID of 513.88
evm.model.unitig_0.1333	An02g06900
evm.model.unitig_0.1334	An02g06890
evm.model.unitig_0.1335	An02g06870
evm.model.unitig_0.1336	An02g06860
evm.model.unitig_0.1337	An02g06850
evm.model.unitig_0.1338	An02g06840
evm.model.unitig_0.1339	An02g06830
evm.model.unitig_0.134	An07g01940
evm.model.unitig_0.1340	An02g06820
evm.model.unitig_0.1341	An02g06810
evm.model.unitig_0.1342	An02g06780
evm.model.unitig_0.1343	An02g06770
evm.model.unitig_0.1344	An02g06760
evm.model.unitig_0.1345	An02g06750
evm.model.unitig_0.1346	An02g06740
evm.model.unitig_0.1347	An02g06720
evm.model.unitig_0.1348	An02g06710
evm.model.unitig_0.1349	An02g06700
evm.model.unitig_0.135	An07g01950
evm.model.unitig_0.1350	An02g06680
evm.model.unitig_0.1351	An02g06670
evm.model.unitig_0.1352	An02g06630
evm.model.unitig_0.1353	An02g06610
evm.model.unitig_0.1354	An02g06600
evm.model.unitig_0.1355	An02g06590
evm.model.unitig_0.1356	An02g06580
evm.model.unitig_0.1357	An02g06570
evm.model.unitig_0.1358	An02g06560
evm.model.unitig_0.1359	An02g06550
evm.model.unitig_0.136	An07g01960
evm.model.unitig_0.1360	An02g06530
evm.model.unitig_0.1361	An02g06520
evm.model.unitig_0.1363	An02g06470
evm.model.unitig_0.1364	An02g06460
evm.model.unitig_0.1365	An02g06450
evm.model.unitig_0.1366	An02g06440
evm.model.unitig_0.1367	An02g06430
evm.model.unitig_0.1368	An02g06420
evm.model.unitig_0.1369	An02g06400
evm.model.unitig_0.137	An07g01970
evm.model.unitig_0.1370	An02g06390
evm.model.unitig_0.1371	An02g06380
evm.model.unitig_0.1372	An02g06370

Gene ID of H915-1	Gene ID of 513.88
evm.model.unitig_0.1373	An02g06360
evm.model.unitig_0.1374	An02g06350
evm.model.unitig_0.1375	An02g06340
evm.model.unitig_0.1376	An02g06330
evm.model.unitig_0.1377	An02g06320
evm.model.unitig_0.1378	An02g06300
evm.model.unitig_0.1379	An02g06280
evm.model.unitig_0.138	An07g01990
evm.model.unitig_0.1380	An02g06240
evm.model.unitig_0.1381	An02g06230
evm.model.unitig_0.1382	An02g06220
evm.model.unitig_0.1383	An02g06180
evm.model.unitig_0.1384	An02g06150
evm.model.unitig_0.1385	An02g06140
evm.model.unitig_0.1386	An02g06130
evm.model.unitig_0.1387	An02g06120
evm.model.unitig_0.1388	An02g06090
evm.model.unitig_0.1389	An02g06080
evm.model.unitig_0.139	An07g02000
evm.model.unitig_0.1390	An02g06070
evm.model.unitig_0.1391	An02g06060
evm.model.unitig_0.1392	An02g06050
evm.model.unitig_0.1393	An02g06040
evm.model.unitig_0.1394	An02g06030
evm.model.unitig_0.1395	An02g06020
evm.model.unitig_0.1396	An02g06010
evm.model.unitig_0.1397	An02g06000
evm.model.unitig_0.1398	An02g05930
evm.model.unitig_0.1399	An02g05920
evm.model.unitig_0.140	An07g02010
evm.model.unitig_0.1400	An02g05890
evm.model.unitig_0.1401	An02g05880
evm.model.unitig_0.1402	An02g05870
evm.model.unitig_0.1403	An02g05860
evm.model.unitig_0.1404	An02g05850
evm.model.unitig_0.1405	An02g05840
evm.model.unitig_0.1406	An02g05830
evm.model.unitig_0.1407	An02g05790
evm.model.unitig_0.1408	An02g05760
evm.model.unitig_0.1409	An02g05750
evm.model.unitig_0.141	An07g02020
evm.model.unitig_0.1410	An02g05740
evm.model.unitig_0.1411	An02g05730

Gene ID of H915-1	Gene ID of 513.88
evm.model.unitig_0.1412	An02g05700
evm.model.unitig_0.1413	An02g05690
evm.model.unitig_0.1414	An02g05680
evm.model.unitig_0.1415	An02g05670
evm.model.unitig_0.1416	An02g05660
evm.model.unitig_0.1417	An02g05650
evm.model.unitig_0.1418	An02g05640
evm.model.unitig_0.1419	An02g05630
evm.model.unitig_0.142	An07g02040
evm.model.unitig_0.1420	An02g05610
evm.model.unitig_0.1421	An02g05600
evm.model.unitig_0.1422	An02g05590
evm.model.unitig_0.1424	An17g00640
evm.model.unitig_0.1425	An02g05560
evm.model.unitig_0.1426	An02g05540
evm.model.unitig_0.1427	An02g05530
evm.model.unitig_0.1428	An02g05490
evm.model.unitig_0.1429	An02g05480
evm.model.unitig_0.143	An07g02050
evm.model.unitig_0.1430	An02g05470
evm.model.unitig_0.1431	An02g05460
evm.model.unitig_0.1432	An02g05450
evm.model.unitig_0.1433	An02g05440
evm.model.unitig_0.1434	An02g05420
evm.model.unitig_0.1435	An02g05410
evm.model.unitig_0.1436	An02g05400
evm.model.unitig_0.1437	An02g05390
evm.model.unitig_0.1438	An02g05380
evm.model.unitig_0.1439	An02g05360
evm.model.unitig_0.144	An07g02060
evm.model.unitig_0.1440	An02g05340
evm.model.unitig_0.1441	An02g05320
evm.model.unitig_0.1442	An02g05260
evm.model.unitig_0.1443	An02g05240
evm.model.unitig_0.1444	An02g05230
evm.model.unitig_0.1445	An02g05220
evm.model.unitig_0.1446	An02g05210
evm.model.unitig_0.1447	An02g05190
evm.model.unitig_0.1448	An02g05170
evm.model.unitig_0.1449	An02g05150
evm.model.unitig_0.145	An07g02080
evm.model.unitig_0.1450	An02g05140
evm.model.unitig_0.1451	An02g05120

Gene ID of H915-1	Gene ID of 513.88
evm.model.unitig_0.1452	An02g05100
evm.model.unitig_0.1453	An11g01150
evm.model.unitig_0.1454	An02g05080
evm.model.unitig_0.1455	An02g05070
evm.model.unitig_0.1456	An02g05060
evm.model.unitig_0.1457	An02g05010
evm.model.unitig_0.1458	An02g05000
evm.model.unitig_0.1459	An02g04990
evm.model.unitig_0.146	An07g02090
evm.model.unitig_0.1460	An02g04980
evm.model.unitig_0.1461	An02g04970
evm.model.unitig_0.1462	An02g04960
evm.model.unitig_0.1463	An02g04920
evm.model.unitig_0.1464	An02g04910
evm.model.unitig_0.1465	An02g04900
evm.model.unitig_0.1466	An02g04880
evm.model.unitig_0.1467	An02g04870
evm.model.unitig_0.1468	An02g04860
evm.model.unitig_0.1469	An02g04850
evm.model.unitig_0.147	An07g02100
evm.model.unitig_0.1470	An02g04840
evm.model.unitig_0.1471	An02g04830
evm.model.unitig_0.1472	An02g04820
evm.model.unitig_0.1473	An02g04800
evm.model.unitig_0.1474	An02g04790
evm.model.unitig_0.1475	An02g04750
evm.model.unitig_0.1476	An02g04730
evm.model.unitig_0.1477	An02g04690
evm.model.unitig_0.1478	An02g04680
evm.model.unitig_0.1479	An02g04660
evm.model.unitig_0.148	An07g02110
evm.model.unitig_0.1480	An04g08640
evm.model.unitig_0.1481	An02g04640
evm.model.unitig_0.1482	An02g04630
evm.model.unitig_0.1483	An02g04620
evm.model.unitig_0.1485	An02g04590
evm.model.unitig_0.1486	An02g04580
evm.model.unitig_0.1487	An02g04555
evm.model.unitig_0.1488	An02g04540
evm.model.unitig_0.1489	An02g04530
evm.model.unitig_0.149	An07g02130
evm.model.unitig_0.1490	An02g04520
evm.model.unitig_0.1491	An02g04510

Gene ID of H915-1	Gene ID of 513.88
evm.model.unitig_0.1492	An02g04500
evm.model.unitig_0.1493	An02g04480
evm.model.unitig_0.1494	An02g04460
evm.model.unitig_0.1495	An02g04440
evm.model.unitig_0.1496	An02g04430
evm.model.unitig_0.1497	An02g04420
evm.model.unitig_0.1498	An02g04410
evm.model.unitig_0.1499	An02g04400
evm.model.unitig_0.15	An07g00130
evm.model.unitig_0.1500	An02g04370
evm.model.unitig_0.1501	An02g04360
evm.model.unitig_0.1502	An02g04350
evm.model.unitig_0.1503	An02g04330
evm.model.unitig_0.1504	An02g04320
evm.model.unitig_0.1505	An02g04310
evm.model.unitig_0.1506	An02g04300
evm.model.unitig_0.1507	An02g04290
evm.model.unitig_0.1508	An02g04280
evm.model.unitig_0.1509	An02g04270
evm.model.unitig_0.151	An07g02150
evm.model.unitig_0.1510	An02g04260
evm.model.unitig_0.1511	An02g04250
evm.model.unitig_0.1512	An02g04240
evm.model.unitig_0.1513	An02g04230
evm.model.unitig_0.1514	An02g04220
evm.model.unitig_0.1515	An02g04210
evm.model.unitig_0.1516	An02g04200
evm.model.unitig_0.1517	An02g04190
evm.model.unitig_0.1518	An02g04180
evm.model.unitig_0.1519	An02g04170
evm.model.unitig_0.152	An07g02160
evm.model.unitig_0.1520	An02g04160
evm.model.unitig_0.1521	An02g04150
evm.model.unitig_0.1522	An02g04130
evm.model.unitig_0.1523	An02g04120
evm.model.unitig_0.1524	An02g04110
evm.model.unitig_0.1525	An02g04100
evm.model.unitig_0.1526	An02g04080
evm.model.unitig_0.1527	An02g04070
evm.model.unitig_0.1528	An02g04060
evm.model.unitig_0.1529	An02g04050
evm.model.unitig_0.153	An07g02180
evm.model.unitig_0.1530	An02g04040

Gene ID of H915-1	Gene ID of 513.88
evm.model.unitig_0.1531	An02g04030
evm.model.unitig_0.1532	An02g04020
evm.model.unitig_0.1533	An02g04010
evm.model.unitig_0.1534	An02g04000
evm.model.unitig_0.1535	An02g03990
evm.model.unitig_0.1536	An02g03980
evm.model.unitig_0.1538	An02g03960
evm.model.unitig_0.1539	An02g03950
evm.model.unitig_0.154	An07g02190
evm.model.unitig_0.1540	An02g03940
evm.model.unitig_0.1541	-
evm.model.unitig_0.1542	An02g03860
evm.model.unitig_0.1543	An02g03850
evm.model.unitig_0.1544	An02g03840
evm.model.unitig_0.1545	An02g03830
evm.model.unitig_0.1546	An02g03790
evm.model.unitig_0.1547	An02g03780
evm.model.unitig_0.1548	An02g03770
evm.model.unitig_0.1549	An02g03760
evm.model.unitig_0.155	An07g02200
evm.model.unitig_0.1550	An02g03740
evm.model.unitig_0.1551	An02g03730
evm.model.unitig_0.1552	An02g03728
evm.model.unitig_0.1553	An01g07410
evm.model.unitig_0.1556	An02g03720
evm.model.unitig_0.1557	An02g03710
evm.model.unitig_0.1558	An02g03700
evm.model.unitig_0.1559	An02g03670
evm.model.unitig_0.156	An07g02210
evm.model.unitig_0.1560	An02g03650
evm.model.unitig_0.1561	An02g03630
evm.model.unitig_0.1562	An02g03620
evm.model.unitig_0.1563	An02g03600
evm.model.unitig_0.1564	An02g03590
evm.model.unitig_0.1565	An02g03580
evm.model.unitig_0.1566	An02g03570
evm.model.unitig_0.1567	An02g03540
evm.model.unitig_0.1568	An02g03520
evm.model.unitig_0.1569	An02g03510
evm.model.unitig_0.157	An07g02240
evm.model.unitig_0.1570	An02g03490
evm.model.unitig_0.1571	An02g03480
evm.model.unitig_0.1572	An02g03460

Gene ID of H915-1	Gene ID of 513.88
evm.model.unitig_0.1573	An02g03420
evm.model.unitig_0.1574	An02g03410
evm.model.unitig_0.1575	An02g03400
evm.model.unitig_0.1576	An02g03390
evm.model.unitig_0.1577	An02g03380
evm.model.unitig_0.1578	An02g03360
evm.model.unitig_0.1579	An02g03350
evm.model.unitig_0.158	An07g02250
evm.model.unitig_0.1580	An02g03340
evm.model.unitig_0.1581	An02g03330
evm.model.unitig_0.1582	An02g03320
evm.model.unitig_0.1583	An02g03310
evm.model.unitig_0.1584	An02g03300
evm.model.unitig_0.1585	An02g03290
evm.model.unitig_0.1586	An02g03280
evm.model.unitig_0.1587	An02g03270
evm.model.unitig_0.1588	An02g03260
evm.model.unitig_0.1589	An02g03250
evm.model.unitig_0.159	An07g02270
evm.model.unitig_0.1590	An02g03240
evm.model.unitig_0.1591	An02g03230
evm.model.unitig_0.1592	An02g03220
evm.model.unitig_0.1593	An02g03210
evm.model.unitig_0.1594	An02g03200
evm.model.unitig_0.1595	An02g03190
evm.model.unitig_0.1596	An02g03180
evm.model.unitig_0.1597	An02g03160
evm.model.unitig_0.1598	An02g03140
evm.model.unitig_0.1599	An02g03120
evm.model.unitig_0.16	An07g00150
evm.model.unitig_0.160	An07g02270
evm.model.unitig_0.1600	An02g03110
evm.model.unitig_0.1601	An02g03090
evm.model.unitig_0.1602	An02g03100
evm.model.unitig_0.1603	An02g03010
evm.model.unitig_0.1604	An02g03000
evm.model.unitig_0.1605	An02g02990
evm.model.unitig_0.1606	An02g02980
evm.model.unitig_0.1607	An02g02970
evm.model.unitig_0.1608	An02g02960
evm.model.unitig_0.1609	An02g02950
evm.model.unitig_0.161	An07g02280
evm.model.unitig_0.1610	An02g02940

Gene ID of H915-1	Gene ID of 513.88
evm.model.unitig_0.1611	An02g02930
evm.model.unitig_0.1612	An02g02920
evm.model.unitig_0.1613	An02g02910
evm.model.unitig_0.1614	An02g02890
evm.model.unitig_0.1615	An02g02880
evm.model.unitig_0.1616	An02g02870
evm.model.unitig_0.1617	An02g02860
evm.model.unitig_0.1618	An02g02840
evm.model.unitig_0.1619	An02g02830
evm.model.unitig_0.162	An07g02290
evm.model.unitig_0.1620	An02g02820
evm.model.unitig_0.1621	An02g02810
evm.model.unitig_0.1622	An02g02790
evm.model.unitig_0.1623	An02g02780
evm.model.unitig_0.1624	An02g02750
evm.model.unitig_0.1625	An02g02740
evm.model.unitig_0.1626	An02g02730
evm.model.unitig_0.1627	An02g02720
evm.model.unitig_0.1628	An02g02710
evm.model.unitig_0.1629	An02g02690
evm.model.unitig_0.163	An07g02300
evm.model.unitig_0.1630	An02g02680
evm.model.unitig_0.1631	An02g02660
evm.model.unitig_0.1632	An02g02650
evm.model.unitig_0.1633	An02g02640
evm.model.unitig_0.1634	An02g02620
evm.model.unitig_0.1635	An02g02540
evm.model.unitig_0.1636	An02g02530
evm.model.unitig_0.1637	An02g02520
evm.model.unitig_0.1638	An02g02510
evm.model.unitig_0.1639	An02g02500
evm.model.unitig_0.164	An07g02310
evm.model.unitig_0.1640	An02g02480
evm.model.unitig_0.1641	An02g02460
evm.model.unitig_0.1642	An02g02410
evm.model.unitig_0.1643	An02g02390
evm.model.unitig_0.1644	An02g02380
evm.model.unitig_0.1645	An02g02370
evm.model.unitig_0.1646	An02g02360
evm.model.unitig_0.1647	An02g02340
evm.model.unitig_0.1648	An02g02330
evm.model.unitig_0.1649	An02g02320
evm.model.unitig_0.165	An07g02350

Gene ID of H915-1	Gene ID of 513.88
evm.model.unitig_0.1650	An12g02840
evm.model.unitig_0.1651	An02g02290
evm.model.unitig_0.1652	An02g02280
evm.model.unitig_0.1653	An02g02270
evm.model.unitig_0.1654	An02g02260
evm.model.unitig_0.1655	An02g02250
evm.model.unitig_0.1656	An02g02240
evm.model.unitig_0.1657	An02g02230
evm.model.unitig_0.1658	An02g02210
evm.model.unitig_0.1659	An02g02200
evm.model.unitig_0.166	An07g02360
evm.model.unitig_0.1660	-
evm.model.unitig_0.1661	-
evm.model.unitig_0.1662	An02g02190
evm.model.unitig_0.1663	An02g02180
evm.model.unitig_0.1664	An02g02170
evm.model.unitig_0.1665	An02g02150
evm.model.unitig_0.1666	An02g02070
evm.model.unitig_0.1667	An02g02060
evm.model.unitig_0.1668	An02g02040
evm.model.unitig_0.1669	An02g02030
evm.model.unitig_0.167	An07g02370
evm.model.unitig_0.1670	An02g02020
evm.model.unitig_0.1671	An02g02010
evm.model.unitig_0.1672	An02g02000
evm.model.unitig_0.1673	An02g01990
evm.model.unitig_0.1674	An02g01980
evm.model.unitig_0.1675	An02g01970
evm.model.unitig_0.1676	An02g01950
evm.model.unitig_0.1677	An02g01890
evm.model.unitig_0.1678	An02g01880
evm.model.unitig_0.1679	An02g01870
evm.model.unitig_0.168	An07g02380
evm.model.unitig_0.1680	An02g01860
evm.model.unitig_0.1681	An02g01830
evm.model.unitig_0.1682	An02g01810
evm.model.unitig_0.1683	An02g01800
evm.model.unitig_0.1684	An02g01790
evm.model.unitig_0.1685	An02g01770
evm.model.unitig_0.1686	An02g01760
evm.model.unitig_0.1687	An02g01750
evm.model.unitig_0.1688	An02g01740
evm.model.unitig_0.1689	An02g01730

Gene ID of H915-1	Gene ID of 513.88
evm.model.unitig_0.169	An07g02390
evm.model.unitig_0.1690	An02g01720
evm.model.unitig_0.1691	An02g01700
evm.model.unitig_0.1692	An02g01690
evm.model.unitig_0.1693	An02g01680
evm.model.unitig_0.1694	-
evm.model.unitig_0.1695	An02g01650
evm.model.unitig_0.1696	An02g01640
evm.model.unitig_0.1697	An02g01630
evm.model.unitig_0.1698	An02g01620
evm.model.unitig_0.1699	An02g01610
evm.model.unitig_0.17	An07g00200
evm.model.unitig_0.170	An07g02400
evm.model.unitig_0.1700	An02g01600
evm.model.unitig_0.1701	An02g01590
evm.model.unitig_0.1702	An02g01580
evm.model.unitig_0.1703	-
evm.model.unitig_0.1704	An02g01570
evm.model.unitig_0.1705	An02g01560
evm.model.unitig_0.1706	An02g01550
evm.model.unitig_0.1707	An02g01540
evm.model.unitig_0.1708	An02g01530
evm.model.unitig_0.1709	An02g01520
evm.model.unitig_0.171	-
evm.model.unitig_0.1710	An02g01510
evm.model.unitig_0.1711	An02g01500
evm.model.unitig_0.1712	An02g01490
evm.model.unitig_0.1713	An02g01480
evm.model.unitig_0.1714	An02g01470
evm.model.unitig_0.1716	An02g01430
evm.model.unitig_0.1717	An02g01420
evm.model.unitig_0.1718	An02g01400
evm.model.unitig_0.1719	An02g01390
evm.model.unitig_0.172	-
evm.model.unitig_0.1721	An02g01370
evm.model.unitig_0.1722	An02g01360
evm.model.unitig_0.1723	An02g01350
evm.model.unitig_0.1724	An02g01330
evm.model.unitig_0.1725	An02g01320
evm.model.unitig_0.1726	An02g01290
evm.model.unitig_0.1727	An02g01270
evm.model.unitig_0.1728	-
evm.model.unitig_0.1729	An02g01260

Gene ID of H915-1	Gene ID of 513.88
evm.model.unitig_0.1730	An02g01255
evm.model.unitig_0.1731	An02g01250
evm.model.unitig_0.1732	An02g01240
evm.model.unitig_0.1733	An02g01220
evm.model.unitig_0.1734	An02g01210
evm.model.unitig_0.1735	An02g01200
evm.model.unitig_0.1736	An02g01180
evm.model.unitig_0.1737	An02g01160
evm.model.unitig_0.1738	An02g01140
evm.model.unitig_0.1739	An02g01110
evm.model.unitig_0.174	An07g02440
evm.model.unitig_0.1740	An02g01100
evm.model.unitig_0.1741	An02g01090
evm.model.unitig_0.1742	An02g01080
evm.model.unitig_0.1743	An02g01070
evm.model.unitig_0.1744	An02g01050
evm.model.unitig_0.1745	An02g01040
evm.model.unitig_0.1746	An02g01040
evm.model.unitig_0.1747	An02g01000
evm.model.unitig_0.1748	An02g00990
evm.model.unitig_0.1749	An02g00980
evm.model.unitig_0.175	An07g02450
evm.model.unitig_0.1750	An02g00970
evm.model.unitig_0.1751	An02g00960
evm.model.unitig_0.1753	An02g00910
evm.model.unitig_0.1754	An02g00890
evm.model.unitig_0.1755	An02g00880
evm.model.unitig_0.1756	An02g00870
evm.model.unitig_0.1757	An02g00850
evm.model.unitig_0.1758	An02g00840
evm.model.unitig_0.1759	An02g00810
evm.model.unitig_0.176	An07g02510
evm.model.unitig_0.1760	An02g00780
evm.model.unitig_0.1761	An02g00760
evm.model.unitig_0.1762	An02g00750
evm.model.unitig_0.1763	An02g00740
evm.model.unitig_0.1765	An02g00700
evm.model.unitig_0.1767	An02g00670
evm.model.unitig_0.1768	An02g00660
evm.model.unitig_0.1769	An02g00610
evm.model.unitig_0.177	An07g02530
evm.model.unitig_0.1770	An02g00590
evm.model.unitig_0.1771	An02g00580

Gene ID of H915-1	Gene ID of 513.88
evm.model.unitig_0.1772	An02g00560
evm.model.unitig_0.1773	An02g00540
evm.model.unitig_0.1774	An02g00500
evm.model.unitig_0.1775	An02g00490
evm.model.unitig_0.1776	An02g00470
evm.model.unitig_0.1777	An02g00470
evm.model.unitig_0.1779	An02g00460
evm.model.unitig_0.178	An07g02540
evm.model.unitig_0.1780	An03g05140
evm.model.unitig_0.1781	An02g00420
evm.model.unitig_0.1782	An02g00400
evm.model.unitig_0.1783	An02g00390
evm.model.unitig_0.1784	An06g00670
evm.model.unitig_0.1785	An02g00350
evm.model.unitig_0.1786	An02g00340
evm.model.unitig_0.1787	An02g00320
evm.model.unitig_0.1788	An02g00310
evm.model.unitig_0.1789	An02g00290
evm.model.unitig_0.179	An07g02560
evm.model.unitig_0.1790	An02g00260
evm.model.unitig_0.1791	An02g00260
evm.model.unitig_0.1792	An02g00250
evm.model.unitig_0.1793	An02g00240
evm.model.unitig_0.1794	An02g00230
evm.model.unitig_0.1795	An02g00220
evm.model.unitig_0.1796	An02g00210
evm.model.unitig_0.1797	An02g00200
evm.model.unitig_0.1798	An02g00190
evm.model.unitig_0.1799	An02g00180
evm.model.unitig_0.18	An07g00230
evm.model.unitig_0.180	An07g02570
evm.model.unitig_0.1800	An02g00170
evm.model.unitig_0.1801	An02g00160
evm.model.unitig_0.1802	An02g00150
evm.model.unitig_0.1803	An02g00140
evm.model.unitig_0.1805	An02g00120
evm.model.unitig_0.1806	An02g00110
evm.model.unitig_0.1807	An02g00090
evm.model.unitig_0.1808	An02g00080
evm.model.unitig_0.1809	An02g00070
evm.model.unitig_0.1810	An02g00060
evm.model.unitig_0.1811	-
evm.model.unitig_0.1812	An02g00050

Gene ID of H915-1	Gene ID of 513.88
evm.model.unitig_0.1813	An02g00050
evm.model.unitig_0.1814	An02g00040
evm.model.unitig_0.1815	An02g00030
evm.model.unitig_0.1816	An12g05070
evm.model.unitig_0.1817	An02g06350
evm.model.unitig_0.1820	An03g00140
evm.model.unitig_0.1821	An14g07360
evm.model.unitig_0.1823	An11g10970
evm.model.unitig_0.1824	An16g00740
evm.model.unitig_0.1825	An11g04030
evm.model.unitig_0.1826	An07g04020
evm.model.unitig_0.1827	An18g00230
evm.model.unitig_0.1829	An04g04320
evm.model.unitig_0.184	An07g02650
evm.model.unitig_0.185	An07g02680
evm.model.unitig_0.186	An07g02690
evm.model.unitig_0.187	An07g02700
evm.model.unitig_0.188	An07g02730
evm.model.unitig_0.189	An07g02760
evm.model.unitig_0.19	An07g00240
evm.model.unitig_0.190	An07g02760
evm.model.unitig_0.191	An07g02770
evm.model.unitig_0.192	An07g02780
evm.model.unitig_0.194	An07g02800
evm.model.unitig_0.195	An07g02810
evm.model.unitig_0.196	An07g02820
evm.model.unitig_0.197	An07g02840
evm.model.unitig_0.198	An07g02860
evm.model.unitig_0.199	An07g02870
evm.model.unitig_0.2	An12g08270
evm.model.unitig_0.20	An07g00250
evm.model.unitig_0.200	An07g02880
evm.model.unitig_0.201	An07g02890
evm.model.unitig_0.202	An07g02900
evm.model.unitig_0.203	An07g02930
evm.model.unitig_0.204	An07g02960
evm.model.unitig_0.205	An07g02970
evm.model.unitig_0.206	An07g02990
evm.model.unitig_0.207	An07g03000
evm.model.unitig_0.208	An07g03005
evm.model.unitig_0.209	An07g03020
evm.model.unitig_0.21	An07g00260
evm.model.unitig_0.210	An07g03040

Gene ID of H915-1	Gene ID of 513.88
evm.model.unitig_0.211	An07g03050
evm.model.unitig_0.212	An07g03070
evm.model.unitig_0.213	An07g03080
evm.model.unitig_0.214	An07g03090
evm.model.unitig_0.215	An07g03100
evm.model.unitig_0.216	An07g03110
evm.model.unitig_0.217	An07g03120
evm.model.unitig_0.218	An07g03130
evm.model.unitig_0.219	An07g03140
evm.model.unitig_0.22	An07g00280
evm.model.unitig_0.220	An07g03150
evm.model.unitig_0.221	An07g03160
evm.model.unitig_0.222	An07g03170
evm.model.unitig_0.223	An07g03190
evm.model.unitig_0.224	An07g03200
evm.model.unitig_0.225	An07g03210
evm.model.unitig_0.226	An07g03220
evm.model.unitig_0.227	An07g03250
evm.model.unitig_0.228	An07g03260
evm.model.unitig_0.229	An07g03270
evm.model.unitig_0.23	An07g00290
evm.model.unitig_0.230	An07g03280
evm.model.unitig_0.231	An07g03290
evm.model.unitig_0.232	An07g03300
evm.model.unitig_0.233	An07g03320
evm.model.unitig_0.234	An07g03330
evm.model.unitig_0.235	An01g05470
evm.model.unitig_0.236	An07g03340
evm.model.unitig_0.237	An07g03360
evm.model.unitig_0.238	An07g03370
evm.model.unitig_0.239	An07g03380
evm.model.unitig_0.24	An07g00300
evm.model.unitig_0.241	An07g03390
evm.model.unitig_0.242	An07g03410
evm.model.unitig_0.243	An07g03420
evm.model.unitig_0.244	An07g03430
evm.model.unitig_0.245	An07g03440
evm.model.unitig_0.246	An07g03450
evm.model.unitig_0.247	An07g03460
evm.model.unitig_0.248	An07g03470
evm.model.unitig_0.249	An07g03520
evm.model.unitig_0.25	An07g00310
evm.model.unitig_0.250	An12g04180

Gene ID of H915-1	Gene ID of 513.88
evm.model.unitig_0.251	An11g09540
evm.model.unitig_0.252	An02g00500
evm.model.unitig_0.253	An07g03570
evm.model.unitig_0.254	An07g03590
evm.model.unitig_0.255	An07g03600
evm.model.unitig_0.256	An07g03610
evm.model.unitig_0.257	An07g03620
evm.model.unitig_0.258	An07g03630
evm.model.unitig_0.259	-
evm.model.unitig_0.26	An07g00320
evm.model.unitig_0.260	An07g03660
evm.model.unitig_0.261	An07g03670
evm.model.unitig_0.262	An07g03680
evm.model.unitig_0.263	An07g03690
evm.model.unitig_0.264	An07g03710
evm.model.unitig_0.265	An07g03720
evm.model.unitig_0.266	An07g03730
evm.model.unitig_0.267	An07g03740
evm.model.unitig_0.268	An07g03750
evm.model.unitig_0.269	An07g03760
evm.model.unitig_0.27	An07g00330
evm.model.unitig_0.270	An07g03770
evm.model.unitig_0.271	An07g03850
evm.model.unitig_0.272	An07g03860
evm.model.unitig_0.273	An07g03880
evm.model.unitig_0.274	An07g03890
evm.model.unitig_0.275	An07g03920
evm.model.unitig_0.276	An07g03930
evm.model.unitig_0.277	An07g03940
evm.model.unitig_0.278	An07g03950
evm.model.unitig_0.279	An07g03960
evm.model.unitig_0.280	An07g03970
evm.model.unitig_0.281	An07g03980
evm.model.unitig_0.282	An07g03990
evm.model.unitig_0.283	An07g04000
evm.model.unitig_0.284	An07g04010
evm.model.unitig_0.285	An07g04020
evm.model.unitig_0.286	An07g04030
evm.model.unitig_0.287	An07g04040
evm.model.unitig_0.288	An07g04070
evm.model.unitig_0.289	An07g04120
evm.model.unitig_0.29	An07g00350
evm.model.unitig_0.290	An07g04130

Gene ID of H915-1	Gene ID of 513.88
evm.model.unitig_0.291	An07g04160
evm.model.unitig_0.292	An07g04170
evm.model.unitig_0.293	An07g04180
evm.model.unitig_0.294	An07g04190
evm.model.unitig_0.295	An07g04200
evm.model.unitig_0.296	An07g04240
evm.model.unitig_0.297	An07g04250
evm.model.unitig_0.298	An07g04260
evm.model.unitig_0.299	An07g04270
evm.model.unitig_0.30	An07g00360
evm.model.unitig_0.300	An07g04280
evm.model.unitig_0.301	An07g04290
evm.model.unitig_0.302	An07g04300
evm.model.unitig_0.303	An07g04320
evm.model.unitig_0.304	An07g04330
evm.model.unitig_0.305	An07g04350
evm.model.unitig_0.306	An07g04390
evm.model.unitig_0.307	An16g01460
evm.model.unitig_0.308	An07g04420
evm.model.unitig_0.309	An07g04430
evm.model.unitig_0.31	An07g00370
evm.model.unitig_0.310	An07g04470
evm.model.unitig_0.311	An07g04480
evm.model.unitig_0.312	An07g04490
evm.model.unitig_0.313	An07g04500
evm.model.unitig_0.314	An07g04510
evm.model.unitig_0.315	An07g04520
evm.model.unitig_0.316	An07g04540
evm.model.unitig_0.317	An07g04560
evm.model.unitig_0.318	An07g04580
evm.model.unitig_0.319	An07g04590
evm.model.unitig_0.32	An07g00380
evm.model.unitig_0.320	An07g04610
evm.model.unitig_0.321	An07g04620
evm.model.unitig_0.322	An07g04640
evm.model.unitig_0.323	An07g04650
evm.model.unitig_0.324	An07g04660
evm.model.unitig_0.325	An07g04700
evm.model.unitig_0.326	An07g04730
evm.model.unitig_0.328	An07g04770
evm.model.unitig_0.329	An07g04780
evm.model.unitig_0.33	An07g00400
evm.model.unitig_0.330	An07g04800

Gene ID of H915-1	Gene ID of 513.88
evm.model.unitig_0.331	An07g04810
evm.model.unitig_0.332	An07g04820
evm.model.unitig_0.333	An07g04830
evm.model.unitig_0.334	An07g04840
evm.model.unitig_0.335	An07g04850
evm.model.unitig_0.337	An07g04860
evm.model.unitig_0.338	An11g05110
evm.model.unitig_0.339	An07g04880
evm.model.unitig_0.34	-
evm.model.unitig_0.340	An07g04910
evm.model.unitig_0.341	An07g04920
evm.model.unitig_0.342	An07g04930
evm.model.unitig_0.343	An07g04940
evm.model.unitig_0.344	An07g04960
evm.model.unitig_0.345	An11g09010
evm.model.unitig_0.346	An07g04980
evm.model.unitig_0.347	An07g04990
evm.model.unitig_0.348	An07g05000
evm.model.unitig_0.349	An07g05010
evm.model.unitig_0.35	An07g00440
evm.model.unitig_0.350	An07g05020
evm.model.unitig_0.351	An07g05030
evm.model.unitig_0.352	An07g05040
evm.model.unitig_0.353	An07g05050
evm.model.unitig_0.354	An07g05060
evm.model.unitig_0.355	An07g05080
evm.model.unitig_0.356	An07g05090
evm.model.unitig_0.357	An07g05100
evm.model.unitig_0.358	An07g05110
evm.model.unitig_0.359	An07g05130
evm.model.unitig_0.36	An07g00450
evm.model.unitig_0.360	An07g05140
evm.model.unitig_0.361	An07g05150
evm.model.unitig_0.362	An07g05160
evm.model.unitig_0.363	An07g05170
evm.model.unitig_0.364	An07g05180
evm.model.unitig_0.365	An07g05210
evm.model.unitig_0.366	An07g05230
evm.model.unitig_0.368	An07g05260
evm.model.unitig_0.369	An07g05280
evm.model.unitig_0.37	An07g00470
evm.model.unitig_0.370	An07g05290
evm.model.unitig_0.371	An07g05350

Gene ID of H915-1	Gene ID of 513.88
evm.model.unitig_0.372	-
evm.model.unitig_0.373	An07g05370
evm.model.unitig_0.374	An07g05380
evm.model.unitig_0.375	An07g05410
evm.model.unitig_0.376	An07g05430
evm.model.unitig_0.377	An07g05440
evm.model.unitig_0.379	An07g05460
evm.model.unitig_0.38	An07g00480
evm.model.unitig_0.380	An03g01570
evm.model.unitig_0.381	An07g05500
evm.model.unitig_0.382	An07g05510
evm.model.unitig_0.383	An07g05520
evm.model.unitig_0.384	An07g05550
evm.model.unitig_0.385	An07g05570
evm.model.unitig_0.386	An07g05600
evm.model.unitig_0.387	An07g05610
evm.model.unitig_0.388	An07g05620
evm.model.unitig_0.389	An07g05630
evm.model.unitig_0.39	An07g00510
evm.model.unitig_0.390	An07g05640
evm.model.unitig_0.391	An07g05650
evm.model.unitig_0.392	An07g05660
evm.model.unitig_0.393	An07g05670
evm.model.unitig_0.394	An07g05690
evm.model.unitig_0.395	An07g05710
evm.model.unitig_0.396	An07g05730
evm.model.unitig_0.397	An07g05740
evm.model.unitig_0.398	An07g05750
evm.model.unitig_0.399	An07g05760
evm.model.unitig_0.4	An03g06700
evm.model.unitig_0.40	An07g00520
evm.model.unitig_0.400	An07g05780
evm.model.unitig_0.401	An07g05790
evm.model.unitig_0.402	An07g05800
evm.model.unitig_0.403	An07g05810
evm.model.unitig_0.404	An07g05820
evm.model.unitig_0.405	An07g05830
evm.model.unitig_0.406	An07g05880
evm.model.unitig_0.407	An07g05900
evm.model.unitig_0.408	An07g05920
evm.model.unitig_0.409	An07g05930
evm.model.unitig_0.41	An07g00530
evm.model.unitig_0.410	An07g05940

Gene ID of H915-1	Gene ID of 513.88
evm.model.unitig_0.411	An07g05960
evm.model.unitig_0.412	-
evm.model.unitig_0.413	An07g05970
evm.model.unitig_0.414	An07g05970
evm.model.unitig_0.415	An07g05980
evm.model.unitig_0.416	-
evm.model.unitig_0.417	An07g05990
evm.model.unitig_0.418	An07g06000
evm.model.unitig_0.419	An07g06010
evm.model.unitig_0.42	An07g00540
evm.model.unitig_0.420	An07g06020
evm.model.unitig_0.421	An07g06030
evm.model.unitig_0.422	An07g06040
evm.model.unitig_0.423	An07g06050
evm.model.unitig_0.424	An07g06080
evm.model.unitig_0.425	An07g06100
evm.model.unitig_0.426	An07g06130
evm.model.unitig_0.427	An07g06140
evm.model.unitig_0.428	An07g06150
evm.model.unitig_0.429	An07g06160
evm.model.unitig_0.43	An07g00550
evm.model.unitig_0.430	An07g06190
evm.model.unitig_0.431	An07g06200
evm.model.unitig_0.434	An07g06240
evm.model.unitig_0.435	An07g06270
evm.model.unitig_0.436	An07g06280
evm.model.unitig_0.437	An07g06300
evm.model.unitig_0.438	An07g06310
evm.model.unitig_0.439	An07g06330
evm.model.unitig_0.44	An07g00570
evm.model.unitig_0.440	An07g06340
evm.model.unitig_0.441	An07g06350
evm.model.unitig_0.442	An07g06360
evm.model.unitig_0.443	An07g06370
evm.model.unitig_0.444	An07g06380
evm.model.unitig_0.445	An07g06390
evm.model.unitig_0.446	An07g06400
evm.model.unitig_0.447	An07g06420
evm.model.unitig_0.448	An07g06430
evm.model.unitig_0.449	An02g03670
evm.model.unitig_0.45	An07g00580
evm.model.unitig_0.450	An07g06460
evm.model.unitig_0.451	An07g06480

Gene ID of H915-1	Gene ID of 513.88
evm.model.unitig_0.452	An07g06490
evm.model.unitig_0.453	An07g06500
evm.model.unitig_0.454	An07g06510
evm.model.unitig_0.455	An07g06520
evm.model.unitig_0.456	An07g06530
evm.model.unitig_0.457	An07g06540
evm.model.unitig_0.458	An07g06550
evm.model.unitig_0.459	An07g06570
evm.model.unitig_0.46	An07g00590
evm.model.unitig_0.460	An07g06580
evm.model.unitig_0.461	An07g06590
evm.model.unitig_0.462	An07g06600
evm.model.unitig_0.463	An07g06610
evm.model.unitig_0.464	An07g06630
evm.model.unitig_0.465	An07g06640
evm.model.unitig_0.466	An07g01830
evm.model.unitig_0.467	An07g06700
evm.model.unitig_0.468	An04g09660
evm.model.unitig_0.469	An07g06730
evm.model.unitig_0.47	An07g00620
evm.model.unitig_0.470	An07g06740
evm.model.unitig_0.471	An07g06750
evm.model.unitig_0.472	An07g06760
evm.model.unitig_0.473	An07g06770
evm.model.unitig_0.474	An07g06780
evm.model.unitig_0.475	An04g06250
evm.model.unitig_0.476	An07g06820
evm.model.unitig_0.477	An07g06840
evm.model.unitig_0.478	An07g06880
evm.model.unitig_0.479	An07g06910
evm.model.unitig_0.48	An07g00630
evm.model.unitig_0.480	An07g06960
evm.model.unitig_0.481	An07g06980
evm.model.unitig_0.482	An07g06990
evm.model.unitig_0.483	An07g07000
evm.model.unitig_0.484	An07g07010
evm.model.unitig_0.485	An07g07020
evm.model.unitig_0.486	An07g07050
evm.model.unitig_0.487	An07g07060
evm.model.unitig_0.488	An07g07090
evm.model.unitig_0.489	An07g07100
evm.model.unitig_0.49	An07g00640
evm.model.unitig_0.490	An07g07110

Gene ID of H915-1	Gene ID of 513.88
evm.model.unitig_0.491	An07g07140
evm.model.unitig_0.492	An07g07150
evm.model.unitig_0.493	An07g07160
evm.model.unitig_0.494	An07g07170
evm.model.unitig_0.495	An07g07180
evm.model.unitig_0.496	An07g07190
evm.model.unitig_0.497	An07g07210
evm.model.unitig_0.498	An07g07240
evm.model.unitig_0.499	An07g07250
evm.model.unitig_0.5	An11g06460
evm.model.unitig_0.50	An07g00670
evm.model.unitig_0.500	An07g07260
evm.model.unitig_0.501	An07g07280
evm.model.unitig_0.502	-
evm.model.unitig_0.503	An07g07290
evm.model.unitig_0.504	An07g07300
evm.model.unitig_0.505	An07g07310
evm.model.unitig_0.506	An07g07320
evm.model.unitig_0.507	An07g07340
evm.model.unitig_0.509	An07g07370
evm.model.unitig_0.51	An07g00680
evm.model.unitig_0.510	An07g07380
evm.model.unitig_0.511	An07g07390
evm.model.unitig_0.512	An07g07400
evm.model.unitig_0.513	An07g07420
evm.model.unitig_0.514	An07g07430
evm.model.unitig_0.515	An07g07450
evm.model.unitig_0.516	An07g07460
evm.model.unitig_0.517	An07g07470
evm.model.unitig_0.518	An07g07480
evm.model.unitig_0.519	An07g07500
evm.model.unitig_0.52	An07g00700
evm.model.unitig_0.520	An07g07510
evm.model.unitig_0.521	An07g07520
evm.model.unitig_0.522	An07g07530
evm.model.unitig_0.523	An07g07550
evm.model.unitig_0.524	An07g07560
evm.model.unitig_0.525	An07g07570
evm.model.unitig_0.526	An07g07580
evm.model.unitig_0.527	An07g07590
evm.model.unitig_0.528	An07g07610
evm.model.unitig_0.529	An07g00850
evm.model.unitig_0.53	An07g00730

Gene ID of H915-1	Gene ID of 513.88
evm.model.unitig_0.530	An07g07630
evm.model.unitig_0.531	An07g07650
evm.model.unitig_0.532	An07g07670
evm.model.unitig_0.533	An07g07680
evm.model.unitig_0.534	An07g07690
evm.model.unitig_0.535	An07g07700
evm.model.unitig_0.536	An07g07740
evm.model.unitig_0.537	An07g07760
evm.model.unitig_0.538	An07g07780
evm.model.unitig_0.539	An07g07790
evm.model.unitig_0.54	An07g00750
evm.model.unitig_0.540	An07g07800
evm.model.unitig_0.541	An07g07810
evm.model.unitig_0.542	An07g07820
evm.model.unitig_0.543	An07g07830
evm.model.unitig_0.544	An07g07840
evm.model.unitig_0.545	An07g07850
evm.model.unitig_0.546	An07g07860
evm.model.unitig_0.547	An07g07870
evm.model.unitig_0.548	An07g07880
evm.model.unitig_0.549	An07g07910
evm.model.unitig_0.55	An07g00760
evm.model.unitig_0.550	An07g07930
evm.model.unitig_0.551	An07g07940
evm.model.unitig_0.552	An07g07970
evm.model.unitig_0.553	An07g07980
evm.model.unitig_0.554	An07g08010
evm.model.unitig_0.555	An07g08030
evm.model.unitig_0.556	An16g01690
evm.model.unitig_0.557	An07g08100
evm.model.unitig_0.558	An07g08140
evm.model.unitig_0.56	An07g00790
evm.model.unitig_0.560	An07g08160
evm.model.unitig_0.561	An07g08170
evm.model.unitig_0.562	An07g08190
evm.model.unitig_0.563	An07g08200
evm.model.unitig_0.564	An07g08210
evm.model.unitig_0.565	An07g08220
evm.model.unitig_0.566	An07g08240
evm.model.unitig_0.567	An07g08250
evm.model.unitig_0.568	An07g08280
evm.model.unitig_0.569	An07g08290
evm.model.unitig_0.570	An07g08300

Gene ID of H915-1	Gene ID of 513.88
evm.model.unitig_0.571	An07g08310
evm.model.unitig_0.572	An07g08320
evm.model.unitig_0.573	An07g08330
evm.model.unitig_0.574	An07g08340
evm.model.unitig_0.575	An07g08350
evm.model.unitig_0.576	An07g08360
evm.model.unitig_0.577	An07g08370
evm.model.unitig_0.578	An07g08380
evm.model.unitig_0.579	An07g08390
evm.model.unitig_0.58	An07g00810
evm.model.unitig_0.580	An07g08400
evm.model.unitig_0.581	An11g06480
evm.model.unitig_0.582	An07g08430
evm.model.unitig_0.583	An07g08470
evm.model.unitig_0.584	An07g08490
evm.model.unitig_0.585	An07g08520
evm.model.unitig_0.587	An07g08590
evm.model.unitig_0.588	An07g08600
evm.model.unitig_0.589	An07g08610
evm.model.unitig_0.59	An04g03400
evm.model.unitig_0.590	An07g08620
evm.model.unitig_0.591	An07g08630
evm.model.unitig_0.592	An07g08640
evm.model.unitig_0.593	An07g08650
evm.model.unitig_0.594	An07g08690
evm.model.unitig_0.595	An07g08710
evm.model.unitig_0.596	An07g08720
evm.model.unitig_0.597	An07g08730
evm.model.unitig_0.598	An07g08740
evm.model.unitig_0.599	An07g08750
evm.model.unitig_0.6	An07g00010
evm.model.unitig_0.60	An16g07700
evm.model.unitig_0.600	An07g08760
evm.model.unitig_0.601	An07g08770
evm.model.unitig_0.602	An07g08810
evm.model.unitig_0.603	An07g08820
evm.model.unitig_0.604	An07g08830
evm.model.unitig_0.605	An07g08850
evm.model.unitig_0.606	An07g08860
evm.model.unitig_0.607	An07g08870
evm.model.unitig_0.608	An07g08880
evm.model.unitig_0.609	An07g08890
evm.model.unitig_0.61	An12g10110

Gene ID of H915-1	Gene ID of 513.88
evm.model.unitig_0.610	An07g08900
evm.model.unitig_0.611	An07g08910
evm.model.unitig_0.612	An03g03030
evm.model.unitig_0.613	An07g08920
evm.model.unitig_0.614	An07g08930
evm.model.unitig_0.615	An07g08940
evm.model.unitig_0.616	An07g08950
evm.model.unitig_0.617	An07g08960
evm.model.unitig_0.618	An07g08970
evm.model.unitig_0.619	An07g08980
evm.model.unitig_0.62	An15g04620
evm.model.unitig_0.620	An07g08990
evm.model.unitig_0.621	An07g09005
evm.model.unitig_0.622	An07g09010
evm.model.unitig_0.623	An07g09020
evm.model.unitig_0.624	An07g09040
evm.model.unitig_0.625	An07g09050
evm.model.unitig_0.626	An07g09060
evm.model.unitig_0.627	An07g09070
evm.model.unitig_0.628	An07g09090
evm.model.unitig_0.629	An07g09110
evm.model.unitig_0.63	An12g10140
evm.model.unitig_0.630	An07g09120
evm.model.unitig_0.631	An07g09130
evm.model.unitig_0.632	An07g09140
evm.model.unitig_0.633	An07g09150
evm.model.unitig_0.634	An07g09160
evm.model.unitig_0.635	An07g09170
evm.model.unitig_0.636	An07g09180
evm.model.unitig_0.637	An07g09190
evm.model.unitig_0.638	An07g09200
evm.model.unitig_0.639	An07g09220
evm.model.unitig_0.64	An03g01460
evm.model.unitig_0.640	An07g09230
evm.model.unitig_0.641	An07g09240
evm.model.unitig_0.642	An07g09250
evm.model.unitig_0.643	An07g09260
evm.model.unitig_0.644	An07g09270
evm.model.unitig_0.645	-
evm.model.unitig_0.646	An07g09280
evm.model.unitig_0.647	An07g09290
evm.model.unitig_0.648	An07g09320
evm.model.unitig_0.649	An07g09330

Gene ID of H915-1	Gene ID of 513.88
evm.model.unitig_0.65	An11g04940
evm.model.unitig_0.650	An07g09340
evm.model.unitig_0.651	An07g09350
evm.model.unitig_0.652	An07g09360
evm.model.unitig_0.653	An07g09370
evm.model.unitig_0.654	An07g09380
evm.model.unitig_0.655	An07g09390
evm.model.unitig_0.656	An07g09400
evm.model.unitig_0.658	An07g00320
evm.model.unitig_0.66	An07g00820
evm.model.unitig_0.660	An04g07430
evm.model.unitig_0.661	An04g07400
evm.model.unitig_0.663	An15g06750
evm.model.unitig_0.664	An07g09410
evm.model.unitig_0.665	An07g09430
evm.model.unitig_0.666	An07g09470
evm.model.unitig_0.667	An07g09530
evm.model.unitig_0.668	An07g09540
evm.model.unitig_0.669	An07g09550
evm.model.unitig_0.67	An07g00860
evm.model.unitig_0.670	An07g09560
evm.model.unitig_0.671	An07g09570
evm.model.unitig_0.672	An07g09580
evm.model.unitig_0.673	An07g09610
evm.model.unitig_0.674	An07g09630
evm.model.unitig_0.676	An07g09710
evm.model.unitig_0.677	An07g09720
evm.model.unitig_0.678	An07g09650
evm.model.unitig_0.679	An07g09680
evm.model.unitig_0.68	An07g00905
evm.model.unitig_0.680	An07g09690
evm.model.unitig_0.681	An07g09700
evm.model.unitig_0.682	An07g09730
evm.model.unitig_0.683	An07g09740
evm.model.unitig_0.684	An07g09750
evm.model.unitig_0.685	An07g09760
evm.model.unitig_0.686	An07g09770
evm.model.unitig_0.687	An07g09780
evm.model.unitig_0.688	An07g09780
evm.model.unitig_0.689	An11g07910
evm.model.unitig_0.69	An07g00930
evm.model.unitig_0.690	An04g08560
evm.model.unitig_0.692	An07g02100

Gene ID of H915-1	Gene ID of 513.88
evm.model.unitig_0.693	An18g00810
evm.model.unitig_0.694	An07g09790
evm.model.unitig_0.695	An07g09800
evm.model.unitig_0.696	An07g09810
evm.model.unitig_0.697	An07g09830
evm.model.unitig_0.698	An07g09840
evm.model.unitig_0.699	An07g09850
evm.model.unitig_0.7	An07g00020
evm.model.unitig_0.70	An07g00940
evm.model.unitig_0.700	An07g09860
evm.model.unitig_0.701	An07g09870
evm.model.unitig_0.702	An07g09880
evm.model.unitig_0.703	An07g09890
evm.model.unitig_0.704	An07g09900
evm.model.unitig_0.705	An07g09920
evm.model.unitig_0.706	An07g09950
evm.model.unitig_0.707	An07g09960
evm.model.unitig_0.708	An07g09970
evm.model.unitig_0.709	An07g09990
evm.model.unitig_0.71	An07g00950
evm.model.unitig_0.710	An07g10010
evm.model.unitig_0.711	An07g10020
evm.model.unitig_0.712	An07g10040
evm.model.unitig_0.713	An07g10050
evm.model.unitig_0.714	An07g10080
evm.model.unitig_0.715	An07g10090
evm.model.unitig_0.716	An07g10100
evm.model.unitig_0.717	An07g10110
evm.model.unitig_0.718	An07g10190
evm.model.unitig_0.719	An07g10200
evm.model.unitig_0.72	An07g00970
evm.model.unitig_0.720	An07g10210
evm.model.unitig_0.721	An07g10220
evm.model.unitig_0.722	An07g10230
evm.model.unitig_0.723	An07g10240
evm.model.unitig_0.724	An07g10250
evm.model.unitig_0.725	An07g10270
evm.model.unitig_0.726	An07g10280
evm.model.unitig_0.727	An07g10290
evm.model.unitig_0.728	An07g10310
evm.model.unitig_0.729	An07g10320
evm.model.unitig_0.73	An07g00980
evm.model.unitig_0.730	An07g10340

Gene ID of H915-1	Gene ID of 513.88
evm.model.unitig_0.731	An07g10350
evm.model.unitig_0.732	An07g10370
evm.model.unitig_0.733	An07g10400
evm.model.unitig_0.734	An07g10410
evm.model.unitig_0.735	An07g10420
evm.model.unitig_0.736	An07g10430
evm.model.unitig_0.737	An02g14990
evm.model.unitig_0.738	An02g14980
evm.model.unitig_0.739	An02g14970
evm.model.unitig_0.74	An07g01000
evm.model.unitig_0.740	An02g14950
evm.model.unitig_0.741	An02g14930
evm.model.unitig_0.742	An02g14920
evm.model.unitig_0.743	An02g14900
evm.model.unitig_0.744	An02g14890
evm.model.unitig_0.745	An02g14870
evm.model.unitig_0.746	An02g14860
evm.model.unitig_0.747	An02g14840
evm.model.unitig_0.748	An02g14830
evm.model.unitig_0.749	An02g14820
evm.model.unitig_0.75	An07g01010
evm.model.unitig_0.750	An02g14810
evm.model.unitig_0.751	An02g14800
evm.model.unitig_0.752	An02g14790
evm.model.unitig_0.753	An02g14770
evm.model.unitig_0.754	An02g14760
evm.model.unitig_0.755	An02g14750
evm.model.unitig_0.756	An02g14740
evm.model.unitig_0.757	An02g14730
evm.model.unitig_0.758	An02g14720
evm.model.unitig_0.759	An02g14710
evm.model.unitig_0.76	An07g01030
evm.model.unitig_0.760	An02g14690
evm.model.unitig_0.761	An02g14670
evm.model.unitig_0.762	An02g14660
evm.model.unitig_0.763	An02g14650
evm.model.unitig_0.764	An02g14630
evm.model.unitig_0.765	An02g14620
evm.model.unitig_0.766	An02g14610
evm.model.unitig_0.767	An02g14600
evm.model.unitig_0.768	An02g14590
evm.model.unitig_0.769	An02g14560
evm.model.unitig_0.77	An12g02670

Gene ID of H915-1	Gene ID of 513.88
evm.model.unitig_0.770	An02g14550
evm.model.unitig_0.771	An02g14540
evm.model.unitig_0.772	An02g14530
evm.model.unitig_0.773	An02g14520
evm.model.unitig_0.774	An02g14500
evm.model.unitig_0.775	An02g14490
evm.model.unitig_0.776	An02g14470
evm.model.unitig_0.777	An02g14460
evm.model.unitig_0.778	An02g14450
evm.model.unitig_0.779	An02g14410
evm.model.unitig_0.78	An07g01050
evm.model.unitig_0.780	An02g14400
evm.model.unitig_0.781	An02g14390
evm.model.unitig_0.782	An02g14380
evm.model.unitig_0.783	An02g14360
evm.model.unitig_0.784	An02g14350
evm.model.unitig_0.785	An02g14340
evm.model.unitig_0.786	An02g14330
evm.model.unitig_0.787	An02g14310
evm.model.unitig_0.788	An02g14300
evm.model.unitig_0.789	An02g14290
evm.model.unitig_0.790	An02g14280
evm.model.unitig_0.791	An02g14270
evm.model.unitig_0.792	An02g14240
evm.model.unitig_0.794	An02g14230
evm.model.unitig_0.795	An02g14220
evm.model.unitig_0.796	An02g14210
evm.model.unitig_0.797	An02g14190
evm.model.unitig_0.798	An02g14170
evm.model.unitig_0.799	An02g14160
evm.model.unitig_0.8	An07g00030
evm.model.unitig_0.80	An07g01090
evm.model.unitig_0.800	-
evm.model.unitig_0.801	-
evm.model.unitig_0.802	An02g14110
evm.model.unitig_0.803	An02g14100
evm.model.unitig_0.804	An02g14080
evm.model.unitig_0.805	An02g14070
evm.model.unitig_0.806	An02g14010
evm.model.unitig_0.807	An02g13980
evm.model.unitig_0.808	An02g13970
evm.model.unitig_0.809	An02g13940
evm.model.unitig_0.81	An07g01110

Gene ID of H915-1	Gene ID of 513.88
evm.model.unitig_0.811	An02g13920
evm.model.unitig_0.812	An02g13910
evm.model.unitig_0.813	-
evm.model.unitig_0.814	An02g13900
evm.model.unitig_0.815	An02g13890
evm.model.unitig_0.816	An02g13880
evm.model.unitig_0.817	An02g13870
evm.model.unitig_0.818	An02g13860
evm.model.unitig_0.819	An02g13850
evm.model.unitig_0.820	An02g13840
evm.model.unitig_0.821	An02g13830
evm.model.unitig_0.822	An02g13820
evm.model.unitig_0.823	An02g13810
evm.model.unitig_0.824	An02g13800
evm.model.unitig_0.825	An02g13790
evm.model.unitig_0.826	-
evm.model.unitig_0.827	An02g13770
evm.model.unitig_0.828	An02g13750
evm.model.unitig_0.829	An02g13740
evm.model.unitig_0.83	An07g01160
evm.model.unitig_0.830	An02g13710
evm.model.unitig_0.831	An02g13700
evm.model.unitig_0.832	An02g13680
evm.model.unitig_0.833	An02g13670
evm.model.unitig_0.835	An02g13650
evm.model.unitig_0.836	An02g13640
evm.model.unitig_0.837	An02g13630
evm.model.unitig_0.838	An02g13620
evm.model.unitig_0.839	An02g13600
evm.model.unitig_0.840	An02g13590
evm.model.unitig_0.841	An02g13580
evm.model.unitig_0.842	An02g13570
evm.model.unitig_0.843	An02g13560
evm.model.unitig_0.844	An02g13550
evm.model.unitig_0.845	An02g13540
evm.model.unitig_0.846	An02g13530
evm.model.unitig_0.847	An02g13520
evm.model.unitig_0.848	An02g13510
evm.model.unitig_0.849	An02g13500
evm.model.unitig_0.85	An07g01200
evm.model.unitig_0.850	An02g13490
evm.model.unitig_0.851	An02g13480
evm.model.unitig_0.852	An02g13470

Gene ID of H915-1	Gene ID of 513.88
evm.model.unitig_0.853	An02g13460
evm.model.unitig_0.855	An02g13450
evm.model.unitig_0.856	An02g13440
evm.model.unitig_0.857	An02g13430
evm.model.unitig_0.858	An02g13420
evm.model.unitig_0.859	An02g13410
evm.model.unitig_0.86	An07g01220
evm.model.unitig_0.860	An02g13400
evm.model.unitig_0.861	An02g13390
evm.model.unitig_0.862	An02g13380
evm.model.unitig_0.863	An02g13370
evm.model.unitig_0.864	An02g13360
evm.model.unitig_0.865	An02g13350
evm.model.unitig_0.866	An02g13340
evm.model.unitig_0.867	An02g13330
evm.model.unitig_0.868	An02g13320
evm.model.unitig_0.869	An02g13310
evm.model.unitig_0.87	An17g01560
evm.model.unitig_0.870	An02g13310
evm.model.unitig_0.871	An02g13300
evm.model.unitig_0.872	An02g13290
evm.model.unitig_0.873	An02g13280
evm.model.unitig_0.874	An02g13270
evm.model.unitig_0.875	An02g13260
evm.model.unitig_0.876	An02g13250
evm.model.unitig_0.877	An02g13240
evm.model.unitig_0.879	An02g13220
evm.model.unitig_0.88	An07g01250
evm.model.unitig_0.880	-
evm.model.unitig_0.881	An02g13210
evm.model.unitig_0.882	-
evm.model.unitig_0.883	An02g13190
evm.model.unitig_0.884	An02g13180
evm.model.unitig_0.885	An02g13160
evm.model.unitig_0.886	An02g13150
evm.model.unitig_0.887	An02g13140
evm.model.unitig_0.888	An02g13130
evm.model.unitig_0.889	An02g13120
evm.model.unitig_0.89	An07g01260
evm.model.unitig_0.890	An02g13100
evm.model.unitig_0.891	An02g13090
evm.model.unitig_0.892	An02g13050
evm.model.unitig_0.893	An02g13040

Gene ID of H915-1	Gene ID of 513.88
evm.model.unitig_0.897	An02g12980
evm.model.unitig_0.898	An02g12970
evm.model.unitig_0.899	An11g08370
evm.model.unitig_0.9	An07g00040
evm.model.unitig_0.90	An07g01270
evm.model.unitig_0.900	An02g12940
evm.model.unitig_0.901	An02g12930
evm.model.unitig_0.902	An02g12920
evm.model.unitig_0.903	An02g12900
evm.model.unitig_0.904	An02g12850
evm.model.unitig_0.906	An02g12830
evm.model.unitig_0.907	An02g12820
evm.model.unitig_0.908	An02g12810
evm.model.unitig_0.909	An02g12800
evm.model.unitig_0.91	An07g01280
evm.model.unitig_0.910	An02g12790
evm.model.unitig_0.912	An02g12770
evm.model.unitig_0.913	An02g12760
evm.model.unitig_0.914	An02g12750
evm.model.unitig_0.915	An02g12680
evm.model.unitig_0.916	An02g12670
evm.model.unitig_0.917	An02g12650
evm.model.unitig_0.918	An02g12640
evm.model.unitig_0.919	An02g12630
evm.model.unitig_0.92	An07g01290
evm.model.unitig_0.920	An02g12610
evm.model.unitig_0.921	An02g12600
evm.model.unitig_0.922	An02g12510
evm.model.unitig_0.923	An02g12505
evm.model.unitig_0.924	-
evm.model.unitig_0.925	An02g12503
evm.model.unitig_0.927	An02g12500
evm.model.unitig_0.928	An02g12490
evm.model.unitig_0.929	An02g12480
evm.model.unitig_0.93	An07g01310
evm.model.unitig_0.930	An02g12470
evm.model.unitig_0.931	An02g12460
evm.model.unitig_0.932	An02g12450
evm.model.unitig_0.934	-
evm.model.unitig_0.935	An02g12430
evm.model.unitig_0.936	An02g12420
evm.model.unitig_0.937	An02g12390
evm.model.unitig_0.938	An02g12370

Gene ID of H915-1	Gene ID of 513.88
evm.model.unitig_0.939	An02g12360
evm.model.unitig_0.94	An07g01340
evm.model.unitig_0.940	An02g12350
evm.model.unitig_0.942	An02g12320
evm.model.unitig_0.943	An02g12310
evm.model.unitig_0.944	An02g12260
evm.model.unitig_0.945	An02g12210
evm.model.unitig_0.946	An02g12160
evm.model.unitig_0.947	An02g12140
evm.model.unitig_0.95	An07g01350
evm.model.unitig_0.950	An02g12080
evm.model.unitig_0.951	An02g12070
evm.model.unitig_0.952	An02g12050
evm.model.unitig_0.953	An02g12040
evm.model.unitig_0.954	An02g12030
evm.model.unitig_0.955	An02g12000
evm.model.unitig_0.956	An02g11990
evm.model.unitig_0.957	An02g11980
evm.model.unitig_0.958	-
evm.model.unitig_0.959	An02g11950
evm.model.unitig_0.96	An07g01360
evm.model.unitig_0.960	An02g11940
evm.model.unitig_0.961	An02g11920
evm.model.unitig_0.962	An02g11910
evm.model.unitig_0.963	-
evm.model.unitig_0.964	An02g11900
evm.model.unitig_0.965	An02g11890
evm.model.unitig_0.966	An02g11860
evm.model.unitig_0.967	An12g06430
evm.model.unitig_0.968	An02g11830
evm.model.unitig_0.969	An02g11810
evm.model.unitig_0.97	An07g01380
evm.model.unitig_0.970	An02g11800
evm.model.unitig_0.971	An02g11790
evm.model.unitig_0.972	An02g11760
evm.model.unitig_0.973	An02g11750
evm.model.unitig_0.974	An02g11720
evm.model.unitig_0.976	An02g11700
evm.model.unitig_0.977	An02g11690
evm.model.unitig_0.978	An02g11680
evm.model.unitig_0.979	An02g11670
evm.model.unitig_0.98	An07g01390
evm.model.unitig_0.980	An02g11660

Gene ID of H915-1	Gene ID of 513.88
evm.model.unitig_0.981	An02g11650
evm.model.unitig_0.982	An02g11630
evm.model.unitig_0.983	An02g11625
evm.model.unitig_0.984	An02g11590
evm.model.unitig_0.985	-
evm.model.unitig_0.986	An02g11560
evm.model.unitig_0.987	An02g11550
evm.model.unitig_0.988	An02g11530
evm.model.unitig_0.989	An02g11500
evm.model.unitig_0.99	An07g01400
evm.model.unitig_0.990	An02g11480
evm.model.unitig_0.991	An02g11470
evm.model.unitig_0.992	An02g11460
evm.model.unitig_0.993	An02g11450
evm.model.unitig_0.994	An02g11440
evm.model.unitig_0.996	An02g11420
evm.model.unitig_0.997	An02g11400
evm.model.unitig_0.998	An02g11390
evm.model.unitig_0.999	An02g11360
evm.model.unitig_1.1	An15g05100
evm.model.unitig_1.10	An18g00100
evm.model.unitig_1.100	An18g01890
evm.model.unitig_1.1000	An08g07320
evm.model.unitig_1.1001	An08g07330
evm.model.unitig_1.1002	An08g07340
evm.model.unitig_1.1003	An08g07350
evm.model.unitig_1.1004	An08g07360
evm.model.unitig_1.1005	An08g07370
evm.model.unitig_1.1006	An08g07380
evm.model.unitig_1.1007	An08g07400
evm.model.unitig_1.1008	An08g07420
evm.model.unitig_1.1009	An08g07430
evm.model.unitig_1.101	An12g05650
evm.model.unitig_1.1010	An08g07440
evm.model.unitig_1.1011	An08g07460
evm.model.unitig_1.1012	An08g07465
evm.model.unitig_1.1013	An08g07480
evm.model.unitig_1.1014	An08g07490
evm.model.unitig_1.1015	An08g07510
evm.model.unitig_1.1016	An08g07520
evm.model.unitig_1.1017	An08g07530
evm.model.unitig_1.1018	An08g07540
evm.model.unitig_1.1019	An08g07550

Gene ID of H915-1	Gene ID of 513.88
evm.model.unitig_1.102	An18g01930
evm.model.unitig_1.1020	An08g07560
evm.model.unitig_1.1021	An08g07570
evm.model.unitig_1.1022	An08g07580
evm.model.unitig_1.1023	An08g07590
evm.model.unitig_1.1024	An08g07600
evm.model.unitig_1.1025	An08g07610
evm.model.unitig_1.1026	An06g02010
evm.model.unitig_1.1027	An08g07630
evm.model.unitig_1.1028	An08g07710
evm.model.unitig_1.1029	An08g07740
evm.model.unitig_1.103	An18g01950
evm.model.unitig_1.1030	An08g07770
evm.model.unitig_1.1031	An08g07780
evm.model.unitig_1.1032	An08g07790
evm.model.unitig_1.1033	An08g07800
evm.model.unitig_1.1034	An08g07810
evm.model.unitig_1.1035	An08g07820
evm.model.unitig_1.1036	An08g07820
evm.model.unitig_1.1037	An08g07830
evm.model.unitig_1.1038	An08g07840
evm.model.unitig_1.1039	An08g07870
evm.model.unitig_1.104	An18g01960
evm.model.unitig_1.1040	An08g07890
evm.model.unitig_1.1041	An08g07900
evm.model.unitig_1.1042	An08g07920
evm.model.unitig_1.1043	An08g07930
evm.model.unitig_1.1044	An08g07940
evm.model.unitig_1.1045	An08g07950
evm.model.unitig_1.1046	An08g07960
evm.model.unitig_1.1047	An08g08000
evm.model.unitig_1.1048	An08g08010
evm.model.unitig_1.1049	An08g08020
evm.model.unitig_1.105	An18g01970
evm.model.unitig_1.1050	An08g08030
evm.model.unitig_1.1051	An08g08040
evm.model.unitig_1.1052	An08g08060
evm.model.unitig_1.1053	An08g08070
evm.model.unitig_1.1054	An08g08080
evm.model.unitig_1.1055	An08g08100
evm.model.unitig_1.1057	An08g08120
evm.model.unitig_1.1058	An08g08130
evm.model.unitig_1.1059	An08g08140

Gene ID of H915-1	Gene ID of 513.88
evm.model.unitig_1.106	An18g01980
evm.model.unitig_1.1060	An08g08150
evm.model.unitig_1.1061	An08g08220
evm.model.unitig_1.1062	An08g08230
evm.model.unitig_1.1063	An08g08240
evm.model.unitig_1.1064	An08g08250
evm.model.unitig_1.1065	An08g08260
evm.model.unitig_1.1066	An08g08280
evm.model.unitig_1.1067	An08g08290
evm.model.unitig_1.1068	An08g08300
evm.model.unitig_1.1069	An08g08310
evm.model.unitig_1.107	An18g01990
evm.model.unitig_1.1070	An08g08320
evm.model.unitig_1.1071	An08g08330
evm.model.unitig_1.1072	An08g08340
evm.model.unitig_1.1073	An01g11250
evm.model.unitig_1.1075	An08g08370
evm.model.unitig_1.1076	An08g08380
evm.model.unitig_1.1077	An02g10090
evm.model.unitig_1.1078	An08g08400
evm.model.unitig_1.1079	An08g08410
evm.model.unitig_1.108	An18g02000
evm.model.unitig_1.1080	An08g08430
evm.model.unitig_1.1081	An08g08450
evm.model.unitig_1.1083	An08g08470
evm.model.unitig_1.1084	An08g08480
evm.model.unitig_1.1085	An08g08480
evm.model.unitig_1.1086	An08g08490
evm.model.unitig_1.1087	An08g08500
evm.model.unitig_1.1088	An08g08510
evm.model.unitig_1.1089	An08g08530
evm.model.unitig_1.109	An18g02010
evm.model.unitig_1.1090	An08g08540
evm.model.unitig_1.1091	An08g08550
evm.model.unitig_1.1092	An08g08560
evm.model.unitig_1.1093	An08g08570
evm.model.unitig_1.1094	An08g08590
evm.model.unitig_1.1095	An08g08600
evm.model.unitig_1.1096	An08g08620
evm.model.unitig_1.1097	An08g08660
evm.model.unitig_1.1098	An08g08670
evm.model.unitig_1.11	An18g00110
evm.model.unitig_1.110	An18g02020

Gene ID of H915-1	Gene ID of 513.88
evm.model.unitig_1.1100	An08g08690
evm.model.unitig_1.1102	An08g08710
evm.model.unitig_1.1103	An08g08720
evm.model.unitig_1.1104	An08g08730
evm.model.unitig_1.1105	An08g08740
evm.model.unitig_1.1106	An08g08750
evm.model.unitig_1.1107	An08g08760
evm.model.unitig_1.1108	An08g08770
evm.model.unitig_1.1109	An08g08820
evm.model.unitig_1.111	An18g02050
evm.model.unitig_1.1110	An08g08840
evm.model.unitig_1.1111	An08g08850
evm.model.unitig_1.1112	An08g08860
evm.model.unitig_1.1113	An08g08870
evm.model.unitig_1.1114	An08g08890
evm.model.unitig_1.1115	An08g08910
evm.model.unitig_1.1116	An08g08920
evm.model.unitig_1.1117	An08g08930
evm.model.unitig_1.1118	An08g08940
evm.model.unitig_1.1119	An08g08950
evm.model.unitig_1.112	An18g02060
evm.model.unitig_1.1120	An08g08980
evm.model.unitig_1.1121	An08g09000
evm.model.unitig_1.1122	An08g09010
evm.model.unitig_1.1123	An08g09020
evm.model.unitig_1.1124	An08g09030
evm.model.unitig_1.1125	An08g09040
evm.model.unitig_1.1127	An08g09120
evm.model.unitig_1.1128	An08g09140
evm.model.unitig_1.1129	An08g09150
evm.model.unitig_1.113	An18g02090
evm.model.unitig_1.1130	An08g09160
evm.model.unitig_1.1131	An08g09170
evm.model.unitig_1.1132	An08g09180
evm.model.unitig_1.1133	An08g09190
evm.model.unitig_1.1134	An09g00520
evm.model.unitig_1.1135	An08g09230
evm.model.unitig_1.1136	An08g09240
evm.model.unitig_1.1137	An08g09250
evm.model.unitig_1.1138	An08g09260
evm.model.unitig_1.114	An18g02110
evm.model.unitig_1.1140	An08g09280
evm.model.unitig_1.1141	An15g00280

Gene ID of H915-1	Gene ID of 513.88
evm.model.unitig_1.1142	An08g09310
evm.model.unitig_1.1143	An08g09310
evm.model.unitig_1.1144	An08g09320
evm.model.unitig_1.1145	An08g09330
evm.model.unitig_1.1146	An08g09340
evm.model.unitig_1.1147	An08g09350
evm.model.unitig_1.1148	An08g09360
evm.model.unitig_1.1149	An08g09370
evm.model.unitig_1.115	An18g02130
evm.model.unitig_1.1150	An08g09390
evm.model.unitig_1.1151	An01g00700
evm.model.unitig_1.1152	An08g09420
evm.model.unitig_1.1153	An16g03130
evm.model.unitig_1.1154	An08g09460
evm.model.unitig_1.1155	An08g09470
evm.model.unitig_1.1156	An08g09490
evm.model.unitig_1.1157	An03g03030
evm.model.unitig_1.1159	An08g11190
evm.model.unitig_1.116	An18g02140
evm.model.unitig_1.1161	An08g09550
evm.model.unitig_1.1163	An08g09580
evm.model.unitig_1.1164	An08g09610
evm.model.unitig_1.1165	An08g09630
evm.model.unitig_1.1166	An08g09690
evm.model.unitig_1.1167	An08g09700
evm.model.unitig_1.1169	An08g09750
evm.model.unitig_1.117	An18g02150
evm.model.unitig_1.1170	An08g09760
evm.model.unitig_1.1171	An08g09780
evm.model.unitig_1.1172	An08g09800
evm.model.unitig_1.1173	An08g09810
evm.model.unitig_1.1174	An08g09850
evm.model.unitig_1.1175	An09g01430
evm.model.unitig_1.1176	An08g09870
evm.model.unitig_1.1178	An08g09940
evm.model.unitig_1.1179	An08g09970
evm.model.unitig_1.118	An18g02170
evm.model.unitig_1.1180	An08g09980
evm.model.unitig_1.1181	An12g00100
evm.model.unitig_1.1182	An08g10000
evm.model.unitig_1.1183	An08g10010
evm.model.unitig_1.1184	An08g10020
evm.model.unitig_1.1185	An08g10030

Gene ID of H915-1	Gene ID of 513.88
evm.model.unitig_1.1186	An08g10040
evm.model.unitig_1.1187	An08g10050
evm.model.unitig_1.1188	An08g10060
evm.model.unitig_1.1189	An08g10070
evm.model.unitig_1.119	An18g02190
evm.model.unitig_1.1190	An08g10100
evm.model.unitig_1.1191	An08g10110
evm.model.unitig_1.1192	An08g10130
evm.model.unitig_1.1193	An08g10140
evm.model.unitig_1.1194	An08g10150
evm.model.unitig_1.1197	An08g10170
evm.model.unitig_1.1198	An08g10180
evm.model.unitig_1.1199	An08g10190
evm.model.unitig_1.12	An18g00120
evm.model.unitig_1.120	An18g02200
evm.model.unitig_1.1200	An08g10210
evm.model.unitig_1.1201	An08g10260
evm.model.unitig_1.1202	An08g10270
evm.model.unitig_1.1203	An08g10280
evm.model.unitig_1.1204	An08g10300
evm.model.unitig_1.1205	An08g10310
evm.model.unitig_1.1206	An08g10320
evm.model.unitig_1.1207	An08g10330
evm.model.unitig_1.1208	An08g10340
evm.model.unitig_1.1209	An08g10350
evm.model.unitig_1.121	An18g02210
evm.model.unitig_1.1210	An08g10360
evm.model.unitig_1.1211	-
evm.model.unitig_1.1212	An08g10370
evm.model.unitig_1.1213	An08g10380
evm.model.unitig_1.1214	An08g10390
evm.model.unitig_1.1215	An08g10390
evm.model.unitig_1.1216	An08g10400
evm.model.unitig_1.1217	An08g10410
evm.model.unitig_1.1218	An08g10440
evm.model.unitig_1.1219	An08g10450
evm.model.unitig_1.122	An18g02220
evm.model.unitig_1.1220	An08g10460
evm.model.unitig_1.1221	An08g10480
evm.model.unitig_1.1222	An08g10490
evm.model.unitig_1.1223	An08g10500
evm.model.unitig_1.1224	An08g10510
evm.model.unitig_1.1225	An08g10520

Gene ID of H915-1	Gene ID of 513.88
evm.model.unitig_1.1226	An08g10530
evm.model.unitig_1.1227	An08g10550
evm.model.unitig_1.1228	An08g10560
evm.model.unitig_1.1229	An08g10570
evm.model.unitig_1.123	An18g02230
evm.model.unitig_1.1230	An08g10600
evm.model.unitig_1.1231	An08g10610
evm.model.unitig_1.1232	-
evm.model.unitig_1.1233	An08g10640
evm.model.unitig_1.1234	An08g10650
evm.model.unitig_1.1235	An08g10670
evm.model.unitig_1.1236	An08g10680
evm.model.unitig_1.1237	An08g10690
evm.model.unitig_1.1238	An08g10700
evm.model.unitig_1.1239	An08g10710
evm.model.unitig_1.124	An18g02240
evm.model.unitig_1.1240	An08g10720
evm.model.unitig_1.1241	An08g10740
evm.model.unitig_1.1242	An08g10750
evm.model.unitig_1.1243	An06g01920
evm.model.unitig_1.1244	An06g01910
evm.model.unitig_1.1245	An06g01900
evm.model.unitig_1.1246	An06g01890
evm.model.unitig_1.1247	An06g01880
evm.model.unitig_1.1248	An06g01870
evm.model.unitig_1.1249	An06g01860
evm.model.unitig_1.125	An18g02250
evm.model.unitig_1.1250	An06g01850
evm.model.unitig_1.1251	An06g01840
evm.model.unitig_1.1252	An06g01830
evm.model.unitig_1.1253	An06g01820
evm.model.unitig_1.1254	An06g01810
evm.model.unitig_1.1255	An06g01780
evm.model.unitig_1.1256	An06g01770
evm.model.unitig_1.1257	An06g01760
evm.model.unitig_1.1258	An06g01750
evm.model.unitig_1.1259	An06g01740
evm.model.unitig_1.126	An18g02270
evm.model.unitig_1.1260	An06g01730
evm.model.unitig_1.1261	An06g01710
evm.model.unitig_1.1262	An06g01660
evm.model.unitig_1.1263	An06g01650
evm.model.unitig_1.1264	An06g01640

Gene ID of H915-1	Gene ID of 513.88
evm.model.unitig_1.1265	An06g01630
evm.model.unitig_1.1266	An06g01620
evm.model.unitig_1.1267	An06g01600
evm.model.unitig_1.1268	An06g01590
evm.model.unitig_1.1269	An06g01580
evm.model.unitig_1.127	An18g02280
evm.model.unitig_1.1270	An06g01550
evm.model.unitig_1.1271	An06g01540
evm.model.unitig_1.1272	An06g01530
evm.model.unitig_1.1273	An06g01520
evm.model.unitig_1.1274	An06g01510
evm.model.unitig_1.1275	An06g01500
evm.model.unitig_1.1276	An06g01490
evm.model.unitig_1.1277	An06g01480
evm.model.unitig_1.1278	An06g01470
evm.model.unitig_1.1279	An06g01460
evm.model.unitig_1.128	An18g02305
evm.model.unitig_1.1280	An06g01440
evm.model.unitig_1.1281	An06g01430
evm.model.unitig_1.1282	An06g01400
evm.model.unitig_1.1283	An06g01390
evm.model.unitig_1.1284	An06g01380
evm.model.unitig_1.1285	An06g01370
evm.model.unitig_1.1286	-
evm.model.unitig_1.1287	An06g01360
evm.model.unitig_1.1288	An06g01350
evm.model.unitig_1.1289	An06g01320
evm.model.unitig_1.129	An18g02330
evm.model.unitig_1.1290	An06g01300
evm.model.unitig_1.1291	An06g01290
evm.model.unitig_1.1292	An06g01270
evm.model.unitig_1.1293	An06g01260
evm.model.unitig_1.1294	An06g01220
evm.model.unitig_1.1295	An06g01210
evm.model.unitig_1.1296	An06g01200
evm.model.unitig_1.1297	An06g01190
evm.model.unitig_1.1298	An06g01180
evm.model.unitig_1.1299	An06g01170
evm.model.unitig_1.13	An18g00170
evm.model.unitig_1.130	An18g02350
evm.model.unitig_1.1300	An06g01160
evm.model.unitig_1.1301	An06g01140
evm.model.unitig_1.1302	An06g01130

Gene ID of H915-1	Gene ID of 513.88
evm.model.unitig_1.1303	An06g01120
evm.model.unitig_1.1304	An06g01110
evm.model.unitig_1.1305	An06g01100
evm.model.unitig_1.1306	An06g01080
evm.model.unitig_1.1307	An06g01070
evm.model.unitig_1.1308	An06g01060
evm.model.unitig_1.1309	An06g01050
evm.model.unitig_1.131	-
evm.model.unitig_1.1310	An06g01040
evm.model.unitig_1.1311	An06g01030
evm.model.unitig_1.1312	An06g01020
evm.model.unitig_1.1313	-
evm.model.unitig_1.1314	An06g01000
evm.model.unitig_1.1315	An06g00990
evm.model.unitig_1.1316	An06g00970
evm.model.unitig_1.1317	An01g12480
evm.model.unitig_1.1318	An06g00950
evm.model.unitig_1.1319	An06g00940
evm.model.unitig_1.132	An18g02360
evm.model.unitig_1.1320	An06g00930
evm.model.unitig_1.1321	An06g00920
evm.model.unitig_1.1322	An06g00900
evm.model.unitig_1.1323	-
evm.model.unitig_1.1324	An06g00890
evm.model.unitig_1.1325	An06g00860
evm.model.unitig_1.1326	An06g00850
evm.model.unitig_1.1327	An06g00840
evm.model.unitig_1.1328	An06g00830
evm.model.unitig_1.1329	An06g00820
evm.model.unitig_1.133	An18g02370
evm.model.unitig_1.1330	An06g00810
evm.model.unitig_1.1332	An08g07440
evm.model.unitig_1.1333	An06g00770
evm.model.unitig_1.1334	An06g00740
evm.model.unitig_1.1335	An04g02690
evm.model.unitig_1.1336	An06g00750
evm.model.unitig_1.1337	An12g08620
evm.model.unitig_1.1338	An06g00760
evm.model.unitig_1.1339	An14g07040
evm.model.unitig_1.134	An18g02380
evm.model.unitig_1.1340	An06g00720
evm.model.unitig_1.1341	-
evm.model.unitig_1.1342	An05g00630

Gene ID of H915-1	Gene ID of 513.88
evm.model.unitig_1.1343	An06g00670
evm.model.unitig_1.1344	An06g00660
evm.model.unitig_1.1345	An06g00640
evm.model.unitig_1.1346	An06g00630
evm.model.unitig_1.1347	An06g00620
evm.model.unitig_1.1348	An06g00610
evm.model.unitig_1.1349	An06g00590
evm.model.unitig_1.135	An18g02400
evm.model.unitig_1.1350	An06g00580
evm.model.unitig_1.1351	An08g12110
evm.model.unitig_1.1352	An06g00560
evm.model.unitig_1.1355	An06g00490
evm.model.unitig_1.1356	An08g06440
evm.model.unitig_1.1357	An06g00460
evm.model.unitig_1.1358	An06g00430
evm.model.unitig_1.1359	An04g04750
evm.model.unitig_1.136	An18g02410
evm.model.unitig_1.1360	An06g00380
evm.model.unitig_1.1362	An06g00370
evm.model.unitig_1.1363	An06g00360
evm.model.unitig_1.1364	An06g00350
evm.model.unitig_1.1366	An06g00340
evm.model.unitig_1.1367	An06g00330
evm.model.unitig_1.1368	An06g00310
evm.model.unitig_1.1369	An06g00300
evm.model.unitig_1.137	An18g02440
evm.model.unitig_1.1370	An06g00290
evm.model.unitig_1.1371	An06g00280
evm.model.unitig_1.1372	An04g09900
evm.model.unitig_1.1373	An06g00270
evm.model.unitig_1.1374	An06g00260
evm.model.unitig_1.1375	An06g00210
evm.model.unitig_1.1376	An06g00190
evm.model.unitig_1.1377	An06g00170
evm.model.unitig_1.1378	An06g00160
evm.model.unitig_1.1379	An06g00150
evm.model.unitig_1.138	An18g02460
evm.model.unitig_1.1380	An06g00150
evm.model.unitig_1.1381	An06g00120
evm.model.unitig_1.1384	An17g00380
evm.model.unitig_1.1385	An08g11160
evm.model.unitig_1.1386	An08g11120
evm.model.unitig_1.1387	An08g11070

Gene ID of H915-1	Gene ID of 513.88
evm.model.unitig_1.1388	An08g11060
evm.model.unitig_1.1389	An08g11040
evm.model.unitig_1.139	An18g02470
evm.model.unitig_1.1390	An08g11030
evm.model.unitig_1.1391	An08g11010
evm.model.unitig_1.1392	An08g10990
evm.model.unitig_1.1393	An08g10980
evm.model.unitig_1.1394	An08g10970
evm.model.unitig_1.1395	An08g10950
evm.model.unitig_1.1396	An08g10930
evm.model.unitig_1.1397	An08g10920
evm.model.unitig_1.1399	An08g10880
evm.model.unitig_1.14	An18g00230
evm.model.unitig_1.140	An18g02480
evm.model.unitig_1.1400	An08g10870
evm.model.unitig_1.1401	An08g10860
evm.model.unitig_1.1402	An08g10830
evm.model.unitig_1.1403	An08g10820
evm.model.unitig_1.1404	An08g10810
evm.model.unitig_1.1405	An08g10800
evm.model.unitig_1.1406	An08g10790
evm.model.unitig_1.1407	An08g10780
evm.model.unitig_1.1409	An08g10760
evm.model.unitig_1.141	An18g02490
evm.model.unitig_1.1410	An08g10750
evm.model.unitig_1.1411	An06g01970
evm.model.unitig_1.1412	An06g01980
evm.model.unitig_1.1413	An06g01990
evm.model.unitig_1.1414	An06g02000
evm.model.unitig_1.1415	An06g02010
evm.model.unitig_1.1416	An06g02020
evm.model.unitig_1.1417	An06g02030
evm.model.unitig_1.1418	An06g02040
evm.model.unitig_1.1419	An06g02050
evm.model.unitig_1.142	An18g02510
evm.model.unitig_1.1420	An06g02060
evm.model.unitig_1.1421	An07g06270
evm.model.unitig_1.1422	An06g02070
evm.model.unitig_1.1423	An06g02080
evm.model.unitig_1.1424	An06g02090
evm.model.unitig_1.1425	An11g05660
evm.model.unitig_1.1426	An06g02120
evm.model.unitig_1.1427	An01g11330

Gene ID of H915-1	Gene ID of 513.88
evm.model.unitig_1.1428	An06g02150
evm.model.unitig_1.143	An18g02520
evm.model.unitig_1.1430	An06g02160
evm.model.unitig_1.1431	An06g02170
evm.model.unitig_1.1432	An06g02180
evm.model.unitig_1.1434	An06g02210
evm.model.unitig_1.1435	An06g02270
evm.model.unitig_1.1436	An06g02430
evm.model.unitig_1.1438	An02g00470
evm.model.unitig_1.1439	An06g02340
evm.model.unitig_1.144	An18g02550
evm.model.unitig_1.1440	An19g00290
evm.model.unitig_1.1441	An06g02380
evm.model.unitig_1.1442	An06g02400
evm.model.unitig_1.1443	An06g02420
evm.model.unitig_1.1444	An06g02430
evm.model.unitig_1.1445	An06g02450
evm.model.unitig_1.1446	An06g02460
evm.model.unitig_1.1447	An06g02470
evm.model.unitig_1.1448	An06g02480
evm.model.unitig_1.1449	An06g02500
evm.model.unitig_1.145	An18g02560
evm.model.unitig_1.1450	An06g02510
evm.model.unitig_1.1451	An02g13500
evm.model.unitig_1.1452	An06g02530
evm.model.unitig_1.1453	An06g02540
evm.model.unitig_1.1454	An06g02550
evm.model.unitig_1.1455	An08g03900
evm.model.unitig_1.1456	An08g03900
evm.model.unitig_1.1457	An06g02640
evm.model.unitig_1.1458	An06g02650
evm.model.unitig_1.1459	An06g02660
evm.model.unitig_1.146	An18g02600
evm.model.unitig_1.1460	An06g02670
evm.model.unitig_1.1461	An06g02680
evm.model.unitig_1.1462	An06g02680
evm.model.unitig_1.1463	An06g02700
evm.model.unitig_1.1464	An06g02710
evm.model.unitig_1.1465	An11g03420
evm.model.unitig_1.147	An18g02650
evm.model.unitig_1.148	An18g02680
evm.model.unitig_1.149	An18g02690
evm.model.unitig_1.15	An18g00240

Gene ID of H915-1	Gene ID of 513.88
evm.model.unitig_1.150	An18g02700
evm.model.unitig_1.151	An18g02710
evm.model.unitig_1.152	An18g02720
evm.model.unitig_1.153	An18g02730
evm.model.unitig_1.154	An12g01360
evm.model.unitig_1.155	An18g02750
evm.model.unitig_1.156	An18g02752
evm.model.unitig_1.157	An18g02760
evm.model.unitig_1.158	An18g02770
evm.model.unitig_1.159	An12g03140
evm.model.unitig_1.16	An18g00260
evm.model.unitig_1.161	An18g02800
evm.model.unitig_1.162	An18g02820
evm.model.unitig_1.163	An18g02830
evm.model.unitig_1.164	An18g02840
evm.model.unitig_1.165	An18g02860
evm.model.unitig_1.166	An18g02890
evm.model.unitig_1.167	An18g02900
evm.model.unitig_1.168	An18g02940
evm.model.unitig_1.169	An18g02950
evm.model.unitig_1.17	-
evm.model.unitig_1.170	An18g02950
evm.model.unitig_1.171	An18g02960
evm.model.unitig_1.172	An18g02970
evm.model.unitig_1.173	An18g02980
evm.model.unitig_1.174	An18g02990
evm.model.unitig_1.175	An18g03000
evm.model.unitig_1.176	An18g03010
evm.model.unitig_1.177	An18g03020
evm.model.unitig_1.178	An18g03030
evm.model.unitig_1.179	An18g03040
evm.model.unitig_1.18	An18g00280
evm.model.unitig_1.180	An18g03060
evm.model.unitig_1.181	An18g03070
evm.model.unitig_1.182	An18g03080
evm.model.unitig_1.183	An18g03090
evm.model.unitig_1.184	An18g03100
evm.model.unitig_1.185	An18g03110
evm.model.unitig_1.186	An18g03120
evm.model.unitig_1.187	An18g03130
evm.model.unitig_1.188	An18g03150
evm.model.unitig_1.189	An18g03170
evm.model.unitig_1.19	An18g00290

Gene ID of H915-1	Gene ID of 513.88
evm.model.unitig_1.190	An18g03180
evm.model.unitig_1.191	An18g03190
evm.model.unitig_1.192	An18g03200
evm.model.unitig_1.193	An18g03210
evm.model.unitig_1.194	An18g03220
evm.model.unitig_1.195	An18g03230
evm.model.unitig_1.196	An18g03240
evm.model.unitig_1.197	An18g03250
evm.model.unitig_1.198	An18g03270
evm.model.unitig_1.199	An18g03290
evm.model.unitig_1.2	An16g06440
evm.model.unitig_1.20	An18g00300
evm.model.unitig_1.200	An18g03300
evm.model.unitig_1.201	An18g03310
evm.model.unitig_1.202	An18g03320
evm.model.unitig_1.203	An18g03330
evm.model.unitig_1.204	An18g03335
evm.model.unitig_1.205	An18g03340
evm.model.unitig_1.206	An18g03350
evm.model.unitig_1.207	An18g03380
evm.model.unitig_1.208	An18g03430
evm.model.unitig_1.209	An18g03450
evm.model.unitig_1.21	An18g00320
evm.model.unitig_1.210	An18g03470
evm.model.unitig_1.211	An18g03480
evm.model.unitig_1.212	An18g03490
evm.model.unitig_1.213	An18g03530
evm.model.unitig_1.214	-
evm.model.unitig_1.215	An18g03550
evm.model.unitig_1.216	An18g03570
evm.model.unitig_1.217	An18g03580
evm.model.unitig_1.218	An18g03590
evm.model.unitig_1.219	An18g03600
evm.model.unitig_1.22	An18g00330
evm.model.unitig_1.220	An18g03610
evm.model.unitig_1.221	An18g03630
evm.model.unitig_1.222	An18g03660
evm.model.unitig_1.223	An18g03670
evm.model.unitig_1.224	An18g03690
evm.model.unitig_1.225	An18g03700
evm.model.unitig_1.226	An18g03710
evm.model.unitig_1.227	An18g03710
evm.model.unitig_1.228	An18g03730

Gene ID of H915-1	Gene ID of 513.88
evm.model.unitig_1.229	An18g03740
evm.model.unitig_1.230	An18g03760
evm.model.unitig_1.231	-
evm.model.unitig_1.232	An18g03770
evm.model.unitig_1.233	An18g03780
evm.model.unitig_1.234	An18g03790
evm.model.unitig_1.235	An18g03800
evm.model.unitig_1.236	An18g03810
evm.model.unitig_1.237	An18g03820
evm.model.unitig_1.238	An18g03830
evm.model.unitig_1.239	An18g03870
evm.model.unitig_1.24	An18g00380
evm.model.unitig_1.240	An18g03880
evm.model.unitig_1.241	An18g03900
evm.model.unitig_1.242	An18g03910
evm.model.unitig_1.243	An18g03920
evm.model.unitig_1.244	An18g03930
evm.model.unitig_1.245	An18g03930
evm.model.unitig_1.246	An18g03950
evm.model.unitig_1.247	An18g03960
evm.model.unitig_1.248	An18g03970
evm.model.unitig_1.249	An18g03980
evm.model.unitig_1.25	An18g00390
evm.model.unitig_1.250	An18g03990
evm.model.unitig_1.251	An18g04010
evm.model.unitig_1.252	An18g04020
evm.model.unitig_1.253	An18g04030
evm.model.unitig_1.254	An18g04040
evm.model.unitig_1.255	An18g04060
evm.model.unitig_1.257	An18g04070
evm.model.unitig_1.258	An18g04080
evm.model.unitig_1.259	An18g04090
evm.model.unitig_1.26	An18g00400
evm.model.unitig_1.260	An18g04100
evm.model.unitig_1.261	An18g04110
evm.model.unitig_1.262	An18g04120
evm.model.unitig_1.263	An18g04130
evm.model.unitig_1.264	An18g04140
evm.model.unitig_1.265	An18g04150
evm.model.unitig_1.266	An18g04160
evm.model.unitig_1.268	An18g04170
evm.model.unitig_1.269	An18g04180
evm.model.unitig_1.27	An18g00410

Gene ID of H915-1	Gene ID of 513.88
evm.model.unitig_1.270	An18g04190
evm.model.unitig_1.271	An18g04200
evm.model.unitig_1.272	An18g04210
evm.model.unitig_1.274	An18g04220
evm.model.unitig_1.275	An18g04230
evm.model.unitig_1.276	An18g04240
evm.model.unitig_1.277	An18g04250
evm.model.unitig_1.278	An18g04260
evm.model.unitig_1.279	An18g04270
evm.model.unitig_1.28	An18g00420
evm.model.unitig_1.280	An18g04280
evm.model.unitig_1.281	An18g04290
evm.model.unitig_1.282	An18g04300
evm.model.unitig_1.283	An18g04310
evm.model.unitig_1.284	An18g04320
evm.model.unitig_1.285	An18g04330
evm.model.unitig_1.286	An18g04340
evm.model.unitig_1.287	An18g04350
evm.model.unitig_1.288	An18g04360
evm.model.unitig_1.289	An18g04400
evm.model.unitig_1.29	An18g00430
evm.model.unitig_1.290	An18g04410
evm.model.unitig_1.291	An18g04420
evm.model.unitig_1.292	An18g04430
evm.model.unitig_1.293	An18g04440
evm.model.unitig_1.294	An18g04460
evm.model.unitig_1.295	An18g04470
evm.model.unitig_1.296	An18g04480
evm.model.unitig_1.297	An18g04490
evm.model.unitig_1.298	An18g04520
evm.model.unitig_1.299	An18g04530
evm.model.unitig_1.3	An18g00010
evm.model.unitig_1.30	An18g00440
evm.model.unitig_1.300	An18g04540
evm.model.unitig_1.301	An18g04550
evm.model.unitig_1.302	An18g04560
evm.model.unitig_1.303	An18g04570
evm.model.unitig_1.304	An18g04580
evm.model.unitig_1.305	An18g04590
evm.model.unitig_1.306	An18g04600
evm.model.unitig_1.307	An18g04610
evm.model.unitig_1.308	An18g04620
evm.model.unitig_1.309	An18g04640

Gene ID of H915-1	Gene ID of 513.88
evm.model.unitig_1.31	An18g00460
evm.model.unitig_1.310	An18g04650
evm.model.unitig_1.311	An18g04660
evm.model.unitig_1.312	An18g04670
evm.model.unitig_1.313	An18g04690
evm.model.unitig_1.314	An18g04730
evm.model.unitig_1.315	An18g04750
evm.model.unitig_1.316	An18g04780
evm.model.unitig_1.317	An18g04790
evm.model.unitig_1.318	-
evm.model.unitig_1.319	An18g04800
evm.model.unitig_1.32	An18g00470
evm.model.unitig_1.320	An18g04810
evm.model.unitig_1.322	An18g04840
evm.model.unitig_1.323	An18g04850
evm.model.unitig_1.324	An18g04870
evm.model.unitig_1.325	An18g04880
evm.model.unitig_1.327	An18g05000
evm.model.unitig_1.328	An18g05020
evm.model.unitig_1.329	An18g05040
evm.model.unitig_1.33	An18g00490
evm.model.unitig_1.330	An18g05050
evm.model.unitig_1.331	An18g05060
evm.model.unitig_1.332	An18g05070
evm.model.unitig_1.333	An18g05080
evm.model.unitig_1.334	An18g05090
evm.model.unitig_1.335	An18g05100
evm.model.unitig_1.336	An18g05110
evm.model.unitig_1.337	An18g05120
evm.model.unitig_1.338	An18g05130
evm.model.unitig_1.339	An18g05140
evm.model.unitig_1.34	An18g00500
evm.model.unitig_1.340	An18g05150
evm.model.unitig_1.341	An18g05170
evm.model.unitig_1.342	An18g05180
evm.model.unitig_1.343	An18g05190
evm.model.unitig_1.344	An18g05210
evm.model.unitig_1.345	An18g05220
evm.model.unitig_1.346	An18g05230
evm.model.unitig_1.348	An18g05270
evm.model.unitig_1.349	An18g05280
evm.model.unitig_1.35	An18g00510
evm.model.unitig_1.350	An18g05340

Gene ID of H915-1	Gene ID of 513.88
evm.model.unitig_1.351	An18g05380
evm.model.unitig_1.352	An18g05420
evm.model.unitig_1.353	An18g05430
evm.model.unitig_1.354	An18g05450
evm.model.unitig_1.355	An18g05470
evm.model.unitig_1.356	An18g05480
evm.model.unitig_1.357	An18g05490
evm.model.unitig_1.358	An18g05500
evm.model.unitig_1.359	An18g05520
evm.model.unitig_1.36	An18g00520
evm.model.unitig_1.360	An18g05540
evm.model.unitig_1.361	An18g05550
evm.model.unitig_1.362	An18g05560
evm.model.unitig_1.363	An18g05570
evm.model.unitig_1.364	An18g05590
evm.model.unitig_1.365	An18g05600
evm.model.unitig_1.366	An18g05610
evm.model.unitig_1.367	An18g05620
evm.model.unitig_1.368	An18g05630
evm.model.unitig_1.369	An18g05640
evm.model.unitig_1.37	An18g00540
evm.model.unitig_1.370	An18g05650
evm.model.unitig_1.371	An18g05660
evm.model.unitig_1.372	An18g05670
evm.model.unitig_1.373	An18g05680
evm.model.unitig_1.374	An18g05690
evm.model.unitig_1.375	-
evm.model.unitig_1.376	An18g05700
evm.model.unitig_1.377	An18g05710
evm.model.unitig_1.378	An18g05720
evm.model.unitig_1.379	An18g05730
evm.model.unitig_1.38	An18g00540
evm.model.unitig_1.380	An18g05740
evm.model.unitig_1.381	An18g05750
evm.model.unitig_1.382	An18g05760
evm.model.unitig_1.383	An18g05770
evm.model.unitig_1.384	An18g05780
evm.model.unitig_1.385	An18g05800
evm.model.unitig_1.386	An18g05810
evm.model.unitig_1.387	An18g05820
evm.model.unitig_1.388	An18g05830
evm.model.unitig_1.389	An18g05840
evm.model.unitig_1.39	An18g00550

Gene ID of H915-1	Gene ID of 513.88
evm.model.unitig_1.390	An18g05850
evm.model.unitig_1.391	An18g05860
evm.model.unitig_1.392	An18g05870
evm.model.unitig_1.393	An18g05880
evm.model.unitig_1.394	An18g05890
evm.model.unitig_1.395	An18g05900
evm.model.unitig_1.396	An18g05910
evm.model.unitig_1.397	An18g05920
evm.model.unitig_1.398	An18g05940
evm.model.unitig_1.399	An18g05960
evm.model.unitig_1.4	An18g00030
evm.model.unitig_1.406	An08g08480
evm.model.unitig_1.409	An18g05980
evm.model.unitig_1.41	An18g00620
evm.model.unitig_1.410	An18g06010
evm.model.unitig_1.411	An18g06020
evm.model.unitig_1.412	An18g06050
evm.model.unitig_1.413	An18g06110
evm.model.unitig_1.414	An18g06120
evm.model.unitig_1.415	An18g06130
evm.model.unitig_1.416	An18g06160
evm.model.unitig_1.417	An18g06170
evm.model.unitig_1.418	An18g06190
evm.model.unitig_1.419	An18g06210
evm.model.unitig_1.42	An18g00630
evm.model.unitig_1.420	An18g06220
evm.model.unitig_1.421	An18g06230
evm.model.unitig_1.422	An18g06250
evm.model.unitig_1.423	An18g06260
evm.model.unitig_1.424	An18g06270
evm.model.unitig_1.425	An18g06290
evm.model.unitig_1.426	An18g06310
evm.model.unitig_1.427	An18g06320
evm.model.unitig_1.428	An18g06340
evm.model.unitig_1.429	An18g06350
evm.model.unitig_1.43	An18g00730
evm.model.unitig_1.430	An18g06360
evm.model.unitig_1.431	An18g06370
evm.model.unitig_1.432	An18g06380
evm.model.unitig_1.433	An18g06390
evm.model.unitig_1.434	An18g06400
evm.model.unitig_1.435	An18g06410
evm.model.unitig_1.436	An18g06430

Gene ID of H915-1	Gene ID of 513.88
evm.model.unitig_1.437	An18g06440
evm.model.unitig_1.438	An18g06470
evm.model.unitig_1.439	-
evm.model.unitig_1.44	An18g00740
evm.model.unitig_1.440	An18g06490
evm.model.unitig_1.441	An18g06500
evm.model.unitig_1.442	An18g06510
evm.model.unitig_1.443	An18g06520
evm.model.unitig_1.444	An18g06540
evm.model.unitig_1.445	An18g06570
evm.model.unitig_1.446	An18g06580
evm.model.unitig_1.447	An18g06590
evm.model.unitig_1.448	An18g06600
evm.model.unitig_1.449	An18g06610
evm.model.unitig_1.45	An18g00740
evm.model.unitig_1.451	An18g06650
evm.model.unitig_1.452	An18g06670
evm.model.unitig_1.453	An18g06680
evm.model.unitig_1.454	An18g06690
evm.model.unitig_1.455	An18g06700
evm.model.unitig_1.456	An18g06710
evm.model.unitig_1.457	An18g06720
evm.model.unitig_1.458	An18g06730
evm.model.unitig_1.459	An18g06740
evm.model.unitig_1.46	An18g00810
evm.model.unitig_1.460	An18g06750
evm.model.unitig_1.461	An18g06760
evm.model.unitig_1.463	An18g06780
evm.model.unitig_1.464	An18g06800
evm.model.unitig_1.465	An18g06810
evm.model.unitig_1.467	An18g06820
evm.model.unitig_1.468	An18g06840
evm.model.unitig_1.47	An18g00820
evm.model.unitig_1.471	An18g04650
evm.model.unitig_1.472	An08g00010
evm.model.unitig_1.473	An08g00030
evm.model.unitig_1.474	An08g00070
evm.model.unitig_1.475	An08g00090
evm.model.unitig_1.476	An08g00100
evm.model.unitig_1.477	An08g00180
evm.model.unitig_1.478	An08g00210
evm.model.unitig_1.479	An15g05540
evm.model.unitig_1.48	An18g00840

Gene ID of H915-1	Gene ID of 513.88
evm.model.unitig_1.480	An08g00280
evm.model.unitig_1.481	An08g00290
evm.model.unitig_1.482	An08g00300
evm.model.unitig_1.483	An08g00310
evm.model.unitig_1.484	An08g00340
evm.model.unitig_1.485	An08g00340
evm.model.unitig_1.486	An08g00350
evm.model.unitig_1.487	An08g00360
evm.model.unitig_1.488	An08g00380
evm.model.unitig_1.489	An08g00400
evm.model.unitig_1.49	An18g00850
evm.model.unitig_1.490	An08g00410
evm.model.unitig_1.491	An08g00420
evm.model.unitig_1.492	An08g00430
evm.model.unitig_1.493	An08g00450
evm.model.unitig_1.494	An08g00470
evm.model.unitig_1.495	An08g00480
evm.model.unitig_1.496	An08g00490
evm.model.unitig_1.497	An08g00500
evm.model.unitig_1.498	An08g00510
evm.model.unitig_1.499	An08g00520
evm.model.unitig_1.5	An18g00040
evm.model.unitig_1.50	An18g00870
evm.model.unitig_1.500	An08g00540
evm.model.unitig_1.501	An08g00550
evm.model.unitig_1.502	An08g00560
evm.model.unitig_1.503	An08g00570
evm.model.unitig_1.504	An08g00580
evm.model.unitig_1.505	An08g00590
evm.model.unitig_1.506	An08g00600
evm.model.unitig_1.507	An08g00620
evm.model.unitig_1.508	An08g00630
evm.model.unitig_1.509	An08g00640
evm.model.unitig_1.510	An08g00660
evm.model.unitig_1.511	An08g00670
evm.model.unitig_1.512	An08g00680
evm.model.unitig_1.513	An08g00690
evm.model.unitig_1.514	An08g00700
evm.model.unitig_1.515	An08g00710
evm.model.unitig_1.516	An08g00720
evm.model.unitig_1.517	An08g00730
evm.model.unitig_1.518	An08g00740
evm.model.unitig_1.519	An08g00750

Gene ID of H915-1	Gene ID of 513.88
evm.model.unitig_1.52	An18g00910
evm.model.unitig_1.520	An08g00760
evm.model.unitig_1.521	An08g00770
evm.model.unitig_1.522	An08g00790
evm.model.unitig_1.523	An08g00800
evm.model.unitig_1.524	An08g00810
evm.model.unitig_1.525	An08g00830
evm.model.unitig_1.526	An08g00830
evm.model.unitig_1.527	An08g00840
evm.model.unitig_1.528	An08g00850
evm.model.unitig_1.529	An08g00860
evm.model.unitig_1.53	An18g00940
evm.model.unitig_1.530	An08g00870
evm.model.unitig_1.531	An08g00880
evm.model.unitig_1.532	An08g00890
evm.model.unitig_1.533	An08g00910
evm.model.unitig_1.534	An08g00920
evm.model.unitig_1.535	An08g00930
evm.model.unitig_1.536	An08g00940
evm.model.unitig_1.537	An08g00950
evm.model.unitig_1.538	-
evm.model.unitig_1.539	An08g00960
evm.model.unitig_1.54	An18g00950
evm.model.unitig_1.540	An08g00970
evm.model.unitig_1.541	An08g00980
evm.model.unitig_1.542	An08g00990
evm.model.unitig_1.543	An08g01000
evm.model.unitig_1.544	An08g01020
evm.model.unitig_1.545	An08g01030
evm.model.unitig_1.546	An08g01040
evm.model.unitig_1.547	An08g01060
evm.model.unitig_1.548	-
evm.model.unitig_1.549	An08g01070
evm.model.unitig_1.55	An18g00960
evm.model.unitig_1.550	An08g01080
evm.model.unitig_1.551	An08g01090
evm.model.unitig_1.552	An08g01100
evm.model.unitig_1.553	An08g01110
evm.model.unitig_1.554	An08g01120
evm.model.unitig_1.555	An08g01130
evm.model.unitig_1.556	An08g01140
evm.model.unitig_1.557	An08g01150
evm.model.unitig_1.558	An08g01160

Gene ID of H915-1	Gene ID of 513.88
evm.model.unitig_1.559	An08g01170
evm.model.unitig_1.56	An18g01000
evm.model.unitig_1.560	An08g01180
evm.model.unitig_1.561	An08g01190
evm.model.unitig_1.562	An08g01200
evm.model.unitig_1.563	An08g01210
evm.model.unitig_1.564	An08g01230
evm.model.unitig_1.565	An08g01240
evm.model.unitig_1.566	An08g01250
evm.model.unitig_1.567	An08g01260
evm.model.unitig_1.568	An08g01270
evm.model.unitig_1.569	An08g01280
evm.model.unitig_1.570	An08g01290
evm.model.unitig_1.571	An08g01300
evm.model.unitig_1.572	An08g01310
evm.model.unitig_1.573	An08g01320
evm.model.unitig_1.574	An08g01330
evm.model.unitig_1.575	An08g01340
evm.model.unitig_1.576	An08g01350
evm.model.unitig_1.577	An08g01360
evm.model.unitig_1.578	An08g01370
evm.model.unitig_1.579	An08g01380
evm.model.unitig_1.58	An18g01050
evm.model.unitig_1.581	An08g01420
evm.model.unitig_1.582	An08g01430
evm.model.unitig_1.583	An08g01440
evm.model.unitig_1.584	An08g01460
evm.model.unitig_1.586	An08g01480
evm.model.unitig_1.587	An08g01490
evm.model.unitig_1.588	An08g01500
evm.model.unitig_1.589	An08g01510
evm.model.unitig_1.59	An18g01120
evm.model.unitig_1.590	An08g01520
evm.model.unitig_1.591	An08g01540
evm.model.unitig_1.592	An08g01560
evm.model.unitig_1.593	An08g01580
evm.model.unitig_1.594	An08g01590
evm.model.unitig_1.595	An08g01600
evm.model.unitig_1.596	An08g01610
evm.model.unitig_1.597	An08g01630
evm.model.unitig_1.598	An08g01640
evm.model.unitig_1.599	An08g01650
evm.model.unitig_1.6	An18g00050

Gene ID of H915-1	Gene ID of 513.88
evm.model.unitig_1.60	An18g01130
evm.model.unitig_1.600	An08g01660
evm.model.unitig_1.601	An08g01680
evm.model.unitig_1.602	An08g01690
evm.model.unitig_1.603	An08g01710
evm.model.unitig_1.604	An08g01720
evm.model.unitig_1.605	An08g01730
evm.model.unitig_1.606	An08g01740
evm.model.unitig_1.607	An08g01750
evm.model.unitig_1.608	An08g01760
evm.model.unitig_1.609	An08g01770
evm.model.unitig_1.61	An18g01140
evm.model.unitig_1.610	An08g01780
evm.model.unitig_1.611	An08g01790
evm.model.unitig_1.612	An08g01800
evm.model.unitig_1.613	An08g01810
evm.model.unitig_1.614	An08g01840
evm.model.unitig_1.615	An08g01850
evm.model.unitig_1.616	-
evm.model.unitig_1.617	An08g01860
evm.model.unitig_1.618	An08g01870
evm.model.unitig_1.619	An08g01890
evm.model.unitig_1.62	An18g01150
evm.model.unitig_1.620	An08g01900
evm.model.unitig_1.621	An08g01910
evm.model.unitig_1.622	An08g01920
evm.model.unitig_1.623	An08g01940
evm.model.unitig_1.624	An08g01950
evm.model.unitig_1.625	An08g01960
evm.model.unitig_1.626	An08g01970
evm.model.unitig_1.627	An08g01980
evm.model.unitig_1.628	An08g01990
evm.model.unitig_1.629	An08g02000
evm.model.unitig_1.63	An18g01150
evm.model.unitig_1.630	An08g02020
evm.model.unitig_1.631	An08g02030
evm.model.unitig_1.632	An08g02060
evm.model.unitig_1.633	An08g02080
evm.model.unitig_1.634	An08g02090
evm.model.unitig_1.635	An08g02110
evm.model.unitig_1.636	An08g02170
evm.model.unitig_1.637	An08g02180
evm.model.unitig_1.638	An08g02190

Gene ID of H915-1	Gene ID of 513.88
evm.model.unitig_1.639	An08g02200
evm.model.unitig_1.64	An18g01170
evm.model.unitig_1.640	An08g02210
evm.model.unitig_1.641	An08g02220
evm.model.unitig_1.642	An08g02230
evm.model.unitig_1.643	An08g02260
evm.model.unitig_1.644	An08g02280
evm.model.unitig_1.645	An08g02290
evm.model.unitig_1.646	An08g02310
evm.model.unitig_1.647	An08g02330
evm.model.unitig_1.648	An08g02340
evm.model.unitig_1.649	An08g02350
evm.model.unitig_1.65	An18g01180
evm.model.unitig_1.650	An08g02360
evm.model.unitig_1.651	An08g02390
evm.model.unitig_1.652	An08g02400
evm.model.unitig_1.653	-
evm.model.unitig_1.654	An08g02410
evm.model.unitig_1.655	An08g02420
evm.model.unitig_1.656	An08g02440
evm.model.unitig_1.657	An08g02450
evm.model.unitig_1.658	An08g02460
evm.model.unitig_1.659	An08g02470
evm.model.unitig_1.66	An18g01180
evm.model.unitig_1.660	An08g02480
evm.model.unitig_1.661	An08g02500
evm.model.unitig_1.662	An08g02510
evm.model.unitig_1.663	An08g02520
evm.model.unitig_1.664	An08g02530
evm.model.unitig_1.665	An08g02580
evm.model.unitig_1.666	An08g02590
evm.model.unitig_1.667	An08g02600
evm.model.unitig_1.668	An08g02600
evm.model.unitig_1.669	An08g02670
evm.model.unitig_1.67	An18g01220
evm.model.unitig_1.670	An08g02680
evm.model.unitig_1.671	An08g02700
evm.model.unitig_1.672	An08g02710
evm.model.unitig_1.673	An08g02720
evm.model.unitig_1.674	An08g02730
evm.model.unitig_1.675	An08g02730
evm.model.unitig_1.676	An08g02770
evm.model.unitig_1.677	An08g02780

Gene ID of H915-1	Gene ID of 513.88
evm.model.unitig_1.678	An08g02790
evm.model.unitig_1.679	An08g02800
evm.model.unitig_1.680	An08g02810
evm.model.unitig_1.681	An08g02820
evm.model.unitig_1.682	An08g02830
evm.model.unitig_1.683	An08g02850
evm.model.unitig_1.684	An08g02860
evm.model.unitig_1.685	An08g02870
evm.model.unitig_1.686	An08g02900
evm.model.unitig_1.687	An08g02910
evm.model.unitig_1.688	An08g02920
evm.model.unitig_1.689	An08g02930
evm.model.unitig_1.69	An18g01250
evm.model.unitig_1.690	An08g02960
evm.model.unitig_1.691	An08g02970
evm.model.unitig_1.692	An08g02990
evm.model.unitig_1.693	An08g03000
evm.model.unitig_1.694	An08g03010
evm.model.unitig_1.695	An08g03030
evm.model.unitig_1.696	An08g03040
evm.model.unitig_1.697	An08g03060
evm.model.unitig_1.698	An08g03070
evm.model.unitig_1.699	An08g03080
evm.model.unitig_1.7	An18g00060
evm.model.unitig_1.70	An18g01290
evm.model.unitig_1.700	An08g03090
evm.model.unitig_1.701	An08g03120
evm.model.unitig_1.703	An08g03150
evm.model.unitig_1.704	An08g03170
evm.model.unitig_1.705	An08g03180
evm.model.unitig_1.706	An08g03190
evm.model.unitig_1.707	An08g03200
evm.model.unitig_1.709	An08g03240
evm.model.unitig_1.71	An18g01320
evm.model.unitig_1.710	An08g03250
evm.model.unitig_1.711	An08g03260
evm.model.unitig_1.712	An08g03270
evm.model.unitig_1.713	An08g03280
evm.model.unitig_1.714	An08g03290
evm.model.unitig_1.715	An08g03300
evm.model.unitig_1.716	An08g03320
evm.model.unitig_1.717	An08g03340
evm.model.unitig_1.718	An08g03360

Gene ID of H915-1	Gene ID of 513.88
evm.model.unitig_1.719	An08g03380
evm.model.unitig_1.72	An18g01350
evm.model.unitig_1.720	An08g03390
evm.model.unitig_1.721	An08g03400
evm.model.unitig_1.722	An08g03420
evm.model.unitig_1.723	An08g03430
evm.model.unitig_1.724	An08g03440
evm.model.unitig_1.725	An08g03450
evm.model.unitig_1.726	An08g03460
evm.model.unitig_1.727	An08g03470
evm.model.unitig_1.728	An08g03480
evm.model.unitig_1.729	An08g03490
evm.model.unitig_1.73	An18g01360
evm.model.unitig_1.730	An08g03493
evm.model.unitig_1.731	An08g03510
evm.model.unitig_1.732	An08g03520
evm.model.unitig_1.733	An08g03530
evm.model.unitig_1.734	An08g03550
evm.model.unitig_1.735	An08g03560
evm.model.unitig_1.736	An08g03570
evm.model.unitig_1.737	An08g03580
evm.model.unitig_1.738	An08g03590
evm.model.unitig_1.739	An08g03600
evm.model.unitig_1.74	An18g01380
evm.model.unitig_1.740	An08g03610
evm.model.unitig_1.741	An08g03620
evm.model.unitig_1.742	An08g03630
evm.model.unitig_1.743	An08g03640
evm.model.unitig_1.744	An08g03650
evm.model.unitig_1.745	An08g03660
evm.model.unitig_1.746	An08g03670
evm.model.unitig_1.747	An08g03680
evm.model.unitig_1.748	An08g03690
evm.model.unitig_1.749	An08g03700
evm.model.unitig_1.75	An18g01390
evm.model.unitig_1.750	An08g03710
evm.model.unitig_1.751	An08g03720
evm.model.unitig_1.752	An08g03730
evm.model.unitig_1.753	An08g03740
evm.model.unitig_1.754	An08g03750
evm.model.unitig_1.755	An08g03760
evm.model.unitig_1.756	An08g03770
evm.model.unitig_1.757	An08g03780

Gene ID of H915-1	Gene ID of 513.88
evm.model.unitig_1.758	An08g03790
evm.model.unitig_1.759	An08g03810
evm.model.unitig_1.76	An18g01400
evm.model.unitig_1.760	An08g03820
evm.model.unitig_1.761	An08g03830
evm.model.unitig_1.762	An08g03840
evm.model.unitig_1.763	An08g03850
evm.model.unitig_1.764	-
evm.model.unitig_1.765	An08g03870
evm.model.unitig_1.766	An08g03880
evm.model.unitig_1.767	An08g03890
evm.model.unitig_1.768	An08g03900
evm.model.unitig_1.769	An08g03910
evm.model.unitig_1.77	An18g01410
evm.model.unitig_1.770	An08g03920
evm.model.unitig_1.771	An08g03930
evm.model.unitig_1.772	An08g03950
evm.model.unitig_1.773	An08g03960
evm.model.unitig_1.774	An08g03970
evm.model.unitig_1.775	An08g03980
evm.model.unitig_1.776	An08g04000
evm.model.unitig_1.777	An08g04015
evm.model.unitig_1.778	An08g04030
evm.model.unitig_1.779	An08g04040
evm.model.unitig_1.78	An18g01470
evm.model.unitig_1.781	An08g04060
evm.model.unitig_1.782	An08g04070
evm.model.unitig_1.783	An08g04080
evm.model.unitig_1.784	An08g04090
evm.model.unitig_1.785	An08g04100
evm.model.unitig_1.786	An08g04110
evm.model.unitig_1.787	An08g04120
evm.model.unitig_1.788	An08g04130
evm.model.unitig_1.789	An08g04140
evm.model.unitig_1.79	An18g01480
evm.model.unitig_1.790	An08g04150
evm.model.unitig_1.791	An08g04160
evm.model.unitig_1.793	An08g04240
evm.model.unitig_1.794	An08g04250
evm.model.unitig_1.795	An08g04260
evm.model.unitig_1.796	An08g04280
evm.model.unitig_1.797	An08g04290
evm.model.unitig_1.798	An08g04300

Gene ID of H915-1	Gene ID of 513.88
evm.model.unitig_1.799	An08g04310
evm.model.unitig_1.8	An18g00070
evm.model.unitig_1.80	An18g01490
evm.model.unitig_1.800	An08g04330
evm.model.unitig_1.801	An08g04350
evm.model.unitig_1.802	An08g04360
evm.model.unitig_1.803	An08g04370
evm.model.unitig_1.804	An08g04390
evm.model.unitig_1.805	An08g04400
evm.model.unitig_1.806	An08g04420
evm.model.unitig_1.807	An08g04430
evm.model.unitig_1.808	An08g04440
evm.model.unitig_1.809	An08g04450
evm.model.unitig_1.810	An08g04460
evm.model.unitig_1.811	An08g04470
evm.model.unitig_1.812	An08g04480
evm.model.unitig_1.813	An08g04500
evm.model.unitig_1.814	An08g04540
evm.model.unitig_1.815	An08g04550
evm.model.unitig_1.816	An08g04560
evm.model.unitig_1.817	An08g04570
evm.model.unitig_1.818	An08g04580
evm.model.unitig_1.819	An08g04590
evm.model.unitig_1.82	An18g01520
evm.model.unitig_1.820	An08g04630
evm.model.unitig_1.821	An08g04640
evm.model.unitig_1.822	An08g04640
evm.model.unitig_1.824	An08g04710
evm.model.unitig_1.825	An08g04740
evm.model.unitig_1.826	An08g04750
evm.model.unitig_1.827	An08g04780
evm.model.unitig_1.828	An08g04810
evm.model.unitig_1.829	An08g04820
evm.model.unitig_1.83	An18g01530
evm.model.unitig_1.830	An08g04830
evm.model.unitig_1.831	An08g04830
evm.model.unitig_1.832	An08g04860
evm.model.unitig_1.833	An15g05630
evm.model.unitig_1.834	An08g04870
evm.model.unitig_1.835	An08g04880
evm.model.unitig_1.836	An08g04890
evm.model.unitig_1.837	An08g04910
evm.model.unitig_1.838	An08g04920

Gene ID of H915-1	Gene ID of 513.88
evm.model.unitig_1.839	An08g04930
evm.model.unitig_1.84	An18g01540
evm.model.unitig_1.840	An08g04960
evm.model.unitig_1.841	An08g04990
evm.model.unitig_1.842	An08g05030
evm.model.unitig_1.843	An08g05050
evm.model.unitig_1.844	An08g05060
evm.model.unitig_1.845	An08g05070
evm.model.unitig_1.846	An08g05080
evm.model.unitig_1.847	An08g05100
evm.model.unitig_1.848	An08g05150
evm.model.unitig_1.849	An08g05160
evm.model.unitig_1.85	An18g01570
evm.model.unitig_1.850	An08g05190
evm.model.unitig_1.851	An08g05200
evm.model.unitig_1.852	An08g05230
evm.model.unitig_1.854	An08g05250
evm.model.unitig_1.855	An08g05260
evm.model.unitig_1.856	An08g05278
evm.model.unitig_1.857	An08g05290
evm.model.unitig_1.858	An08g05300
evm.model.unitig_1.859	An08g05310
evm.model.unitig_1.86	An18g01580
evm.model.unitig_1.860	An08g05320
evm.model.unitig_1.861	An08g05330
evm.model.unitig_1.862	An08g05340
evm.model.unitig_1.863	An08g05340
evm.model.unitig_1.864	An08g05360
evm.model.unitig_1.865	An08g05380
evm.model.unitig_1.866	An08g05390
evm.model.unitig_1.867	An08g05400
evm.model.unitig_1.868	An08g05410
evm.model.unitig_1.869	An08g05420
evm.model.unitig_1.87	An18g01590
evm.model.unitig_1.870	An08g05440
evm.model.unitig_1.871	An08g05450
evm.model.unitig_1.872	An08g05470
evm.model.unitig_1.873	An08g05490
evm.model.unitig_1.874	An08g05500
evm.model.unitig_1.875	An08g05510
evm.model.unitig_1.876	An08g05520
evm.model.unitig_1.877	An08g05530
evm.model.unitig_1.878	An08g05540

Gene ID of H915-1	Gene ID of 513.88
evm.model.unitig_1.879	An08g05560
evm.model.unitig_1.88	An18g01610
evm.model.unitig_1.880	An08g05570
evm.model.unitig_1.881	An08g05580
evm.model.unitig_1.882	An08g05590
evm.model.unitig_1.883	An08g05610
evm.model.unitig_1.884	An08g05640
evm.model.unitig_1.885	An08g05670
evm.model.unitig_1.887	An08g05680
evm.model.unitig_1.888	An08g05690
evm.model.unitig_1.889	An08g05700
evm.model.unitig_1.89	-
evm.model.unitig_1.890	An08g05720
evm.model.unitig_1.891	An08g05730
evm.model.unitig_1.892	An08g05760
evm.model.unitig_1.893	An08g05780
evm.model.unitig_1.894	An08g05790
evm.model.unitig_1.895	An08g05820
evm.model.unitig_1.896	An08g05850
evm.model.unitig_1.897	An08g05860
evm.model.unitig_1.898	An08g05870
evm.model.unitig_1.899	-
evm.model.unitig_1.9	An18g00090
evm.model.unitig_1.90	An18g01620
evm.model.unitig_1.900	An08g05890
evm.model.unitig_1.901	An08g05900
evm.model.unitig_1.902	An08g05910
evm.model.unitig_1.903	An08g05920
evm.model.unitig_1.904	An12g01470
evm.model.unitig_1.905	An08g05950
evm.model.unitig_1.907	An08g05970
evm.model.unitig_1.908	An08g06030
evm.model.unitig_1.909	An08g06050
evm.model.unitig_1.91	An18g01630
evm.model.unitig_1.910	An08g06060
evm.model.unitig_1.911	An08g06070
evm.model.unitig_1.912	An08g06080
evm.model.unitig_1.913	An08g06090
evm.model.unitig_1.914	An08g06100
evm.model.unitig_1.915	An08g06120
evm.model.unitig_1.916	-
evm.model.unitig_1.917	An08g06130
evm.model.unitig_1.918	An08g06150

Gene ID of H915-1	Gene ID of 513.88
evm.model.unitig_1.919	An08g06160
evm.model.unitig_1.92	An18g01640
evm.model.unitig_1.920	An08g06170
evm.model.unitig_1.921	An08g06180
evm.model.unitig_1.922	An08g06190
evm.model.unitig_1.923	An08g06200
evm.model.unitig_1.924	An08g06210
evm.model.unitig_1.925	An08g06230
evm.model.unitig_1.926	An08g06240
evm.model.unitig_1.927	An08g06250
evm.model.unitig_1.928	An08g06260
evm.model.unitig_1.929	An08g06270
evm.model.unitig_1.93	An18g01670
evm.model.unitig_1.930	An08g06320
evm.model.unitig_1.931	An08g06330
evm.model.unitig_1.932	An08g06340
evm.model.unitig_1.933	An08g06350
evm.model.unitig_1.934	An08g06360
evm.model.unitig_1.935	An08g06380
evm.model.unitig_1.936	An08g06390
evm.model.unitig_1.937	An08g06400
evm.model.unitig_1.938	An08g06420
evm.model.unitig_1.939	An08g06430
evm.model.unitig_1.94	An18g01680
evm.model.unitig_1.940	An08g06440
evm.model.unitig_1.941	An08g06450
evm.model.unitig_1.942	An08g06460
evm.model.unitig_1.943	An08g06490
evm.model.unitig_1.944	An08g06500
evm.model.unitig_1.945	An08g06520
evm.model.unitig_1.946	An08g06530
evm.model.unitig_1.947	An08g06540
evm.model.unitig_1.948	An08g06560
evm.model.unitig_1.949	An08g06570
evm.model.unitig_1.95	An18g01690
evm.model.unitig_1.950	An08g06580
evm.model.unitig_1.951	An08g06590
evm.model.unitig_1.952	An08g06600
evm.model.unitig_1.953	An08g06610
evm.model.unitig_1.954	An08g06620
evm.model.unitig_1.955	An08g06630
evm.model.unitig_1.956	-
evm.model.unitig_1.957	An08g06650

Gene ID of H915-1	Gene ID of 513.88
evm.model.unitig_1.958	An08g06660
evm.model.unitig_1.959	An08g06670
evm.model.unitig_1.96	An18g01700
evm.model.unitig_1.960	An08g06680
evm.model.unitig_1.961	An08g06690
evm.model.unitig_1.962	An08g06700
evm.model.unitig_1.963	An08g06710
evm.model.unitig_1.964	An08g06720
evm.model.unitig_1.965	An08g06730
evm.model.unitig_1.966	An08g06750
evm.model.unitig_1.967	An08g06760
evm.model.unitig_1.968	An08g06770
evm.model.unitig_1.969	An08g06780
evm.model.unitig_1.97	An18g01720
evm.model.unitig_1.970	An08g06790
evm.model.unitig_1.971	An08g06810
evm.model.unitig_1.972	An08g06850
evm.model.unitig_1.973	An08g06890
evm.model.unitig_1.974	An08g06940
evm.model.unitig_1.975	An08g06960
evm.model.unitig_1.976	An08g06980
evm.model.unitig_1.977	An08g06990
evm.model.unitig_1.979	An08g07020
evm.model.unitig_1.98	An18g01840
evm.model.unitig_1.980	An08g07030
evm.model.unitig_1.981	An08g07040
evm.model.unitig_1.982	An08g07050
evm.model.unitig_1.983	An08g07060
evm.model.unitig_1.984	An08g07070
evm.model.unitig_1.985	An08g07080
evm.model.unitig_1.986	An08g07090
evm.model.unitig_1.987	An08g07100
evm.model.unitig_1.988	An08g07120
evm.model.unitig_1.989	An08g07150
evm.model.unitig_1.99	An18g01860
evm.model.unitig_1.991	An08g07200
evm.model.unitig_1.992	An08g07210
evm.model.unitig_1.993	An08g07220
evm.model.unitig_1.994	An08g07230
evm.model.unitig_1.995	An08g07240
evm.model.unitig_1.996	An08g07250
evm.model.unitig_1.997	An08g07270
evm.model.unitig_1.998	An08g07280

Gene ID of H915-1	Gene ID of 513.88
evm.model.unitig_1.999	An08g07290
evm.model.unitig_13.2	-
evm.model.unitig_16.2	-
evm.model.unitig_19.2	-
evm.model.unitig_2.1	An01g15200
evm.model.unitig_2.10	An01g15120
evm.model.unitig_2.100	An01g13940
evm.model.unitig_2.1000	An01g01490
evm.model.unitig_2.1001	An01g01470
evm.model.unitig_2.1002	An01g01450
evm.model.unitig_2.1003	-
evm.model.unitig_2.1004	An01g01430
evm.model.unitig_2.1005	An12g05390
evm.model.unitig_2.1006	An02g00470
evm.model.unitig_2.1007	An01g01400
evm.model.unitig_2.1008	An01g01390
evm.model.unitig_2.1009	An01g01380
evm.model.unitig_2.101	An01g13930
evm.model.unitig_2.1010	An01g01370
evm.model.unitig_2.1011	An01g01360
evm.model.unitig_2.1012	An01g01350
evm.model.unitig_2.1013	An01g01340
evm.model.unitig_2.1014	An01g01330
evm.model.unitig_2.1015	An01g01320
evm.model.unitig_2.1016	An01g01290
evm.model.unitig_2.1017	An01g01280
evm.model.unitig_2.1018	An01g01260
evm.model.unitig_2.1019	-
evm.model.unitig_2.102	An01g13920
evm.model.unitig_2.1020	An01g01230
evm.model.unitig_2.1021	An01g01220
evm.model.unitig_2.1022	An04g02850
evm.model.unitig_2.1023	An01g01190
evm.model.unitig_2.1024	An01g01180
evm.model.unitig_2.1025	An01g01170
evm.model.unitig_2.1026	An01g01160
evm.model.unitig_2.1027	An01g01120
evm.model.unitig_2.1029	An01g01110
evm.model.unitig_2.103	An01g13910
evm.model.unitig_2.1030	An01g01090
evm.model.unitig_2.1031	An01g01080
evm.model.unitig_2.1032	An01g01070
evm.model.unitig_2.1033	An01g01060

Gene ID of H915-1	Gene ID of 513.88
evm.model.unitig_2.1034	An01g01050
evm.model.unitig_2.1035	An08g11190
evm.model.unitig_2.1036	An01g01030
evm.model.unitig_2.1037	An01g01020
evm.model.unitig_2.1038	An01g01010
evm.model.unitig_2.1039	An01g01000
evm.model.unitig_2.104	An01g13900
evm.model.unitig_2.1040	An01g00960
evm.model.unitig_2.1041	An14g03300
evm.model.unitig_2.1042	An01g00950
evm.model.unitig_2.1043	An01g00930
evm.model.unitig_2.1044	An04g03170
evm.model.unitig_2.1046	An18g01740
evm.model.unitig_2.1047	An09g04810
evm.model.unitig_2.1048	An11g02870
evm.model.unitig_2.1049	An01g00890
evm.model.unitig_2.105	An01g13890
evm.model.unitig_2.1050	An01g00880
evm.model.unitig_2.1051	An01g00870
evm.model.unitig_2.1052	An01g00860
evm.model.unitig_2.1053	An01g00850
evm.model.unitig_2.1054	An01g00820
evm.model.unitig_2.1056	An01g00800
evm.model.unitig_2.1057	An01g00780
evm.model.unitig_2.1058	An01g00770
evm.model.unitig_2.1059	An01g00750
evm.model.unitig_2.106	An01g13880
evm.model.unitig_2.1060	An01g00730
evm.model.unitig_2.1061	An01g00720
evm.model.unitig_2.1062	An01g00710
evm.model.unitig_2.1063	An01g00700
evm.model.unitig_2.1064	An01g00690
evm.model.unitig_2.1065	An01g00680
evm.model.unitig_2.1066	An01g00660
evm.model.unitig_2.1067	An01g00640
evm.model.unitig_2.1068	An01g00630
evm.model.unitig_2.1069	An01g00620
evm.model.unitig_2.107	An01g13830
evm.model.unitig_2.1070	An01g00610
evm.model.unitig_2.1071	An01g00600
evm.model.unitig_2.1072	-
evm.model.unitig_2.1073	An01g00570
evm.model.unitig_2.1074	An01g00560

Gene ID of H915-1	Gene ID of 513.88
evm.model.unitig_2.1075	An01g00550
evm.model.unitig_2.1076	An01g00530
evm.model.unitig_2.1077	An01g00520
evm.model.unitig_2.1078	-
evm.model.unitig_2.1079	An01g00510
evm.model.unitig_2.108	An01g13820
evm.model.unitig_2.1080	An01g00490
evm.model.unitig_2.1081	An01g00480
evm.model.unitig_2.1083	An01g00460
evm.model.unitig_2.1084	An01g00450
evm.model.unitig_2.1086	An01g01580
evm.model.unitig_2.1087	An01g00400
evm.model.unitig_2.1088	An01g00390
evm.model.unitig_2.1089	An01g00380
evm.model.unitig_2.109	An01g13810
evm.model.unitig_2.1090	An11g08190
evm.model.unitig_2.1091	An01g00370
evm.model.unitig_2.1093	An01g00340
evm.model.unitig_2.1094	An01g00330
evm.model.unitig_2.1095	An01g00290
evm.model.unitig_2.1096	An01g00280
evm.model.unitig_2.1097	An01g00270
evm.model.unitig_2.1098	An01g00260
evm.model.unitig_2.1099	An01g00250
evm.model.unitig_2.11	An01g15110
evm.model.unitig_2.110	An01g13790
evm.model.unitig_2.1100	An01g00240
evm.model.unitig_2.1101	An01g00220
evm.model.unitig_2.1102	An01g00210
evm.model.unitig_2.1103	An01g00200
evm.model.unitig_2.1104	An01g00190
evm.model.unitig_2.1105	An01g00170
evm.model.unitig_2.1106	An01g00160
evm.model.unitig_2.1107	An01g00150
evm.model.unitig_2.1108	An01g00140
evm.model.unitig_2.1109	An01g00130
evm.model.unitig_2.111	An01g13780
evm.model.unitig_2.1110	An01g00120
evm.model.unitig_2.1111	An01g00110
evm.model.unitig_2.1112	An01g00100
evm.model.unitig_2.1113	An01g00090
evm.model.unitig_2.1114	An01g00070
evm.model.unitig_2.1115	An01g00070

Gene ID of H915-1	Gene ID of 513.88
evm.model.unitig_2.1116	An01g00060
evm.model.unitig_2.1117	An01g00050
evm.model.unitig_2.1118	An01g00040
evm.model.unitig_2.1119	An01g00030
evm.model.unitig_2.112	An01g13770
evm.model.unitig_2.1120	An03g03700
evm.model.unitig_2.1121	An02g08060
evm.model.unitig_2.1122	An14g04980
evm.model.unitig_2.1124	An14g00540
evm.model.unitig_2.1126	An13g00010
evm.model.unitig_2.1127	An13g00020
evm.model.unitig_2.1128	An13g00030
evm.model.unitig_2.1129	An13g00040
evm.model.unitig_2.113	An01g13750
evm.model.unitig_2.1130	An13g00050
evm.model.unitig_2.1131	An13g00050
evm.model.unitig_2.1132	An13g00080
evm.model.unitig_2.1133	An13g00090
evm.model.unitig_2.1134	An13g00100
evm.model.unitig_2.1135	An13g00110
evm.model.unitig_2.1136	An13g00130
evm.model.unitig_2.1137	An13g00140
evm.model.unitig_2.1138	An13g00210
evm.model.unitig_2.1139	An13g00220
evm.model.unitig_2.114	An01g13740
evm.model.unitig_2.1140	An13g00240
evm.model.unitig_2.1141	An13g00250
evm.model.unitig_2.1142	An13g00270
evm.model.unitig_2.1143	An13g00280
evm.model.unitig_2.1144	An13g00290
evm.model.unitig_2.1145	An13g00300
evm.model.unitig_2.1146	An13g00310
evm.model.unitig_2.1147	An13g00320
evm.model.unitig_2.1148	An13g00370
evm.model.unitig_2.1149	An13g00380
evm.model.unitig_2.1150	An13g00390
evm.model.unitig_2.1151	An13g00400
evm.model.unitig_2.1152	An13g00410
evm.model.unitig_2.1153	An13g00420
evm.model.unitig_2.1154	An16g07920
evm.model.unitig_2.1155	An13g00430
evm.model.unitig_2.1156	An13g00440
evm.model.unitig_2.1157	An13g00450

Gene ID of H915-1	Gene ID of 513.88
evm.model.unitig_2.1158	An13g00460
evm.model.unitig_2.1159	An13g00480
evm.model.unitig_2.1160	An13g00510
evm.model.unitig_2.1161	An13g00520
evm.model.unitig_2.1162	An13g00540
evm.model.unitig_2.1163	An13g00550
evm.model.unitig_2.1164	An13g00560
evm.model.unitig_2.1166	An13g00590
evm.model.unitig_2.1167	An13g00600
evm.model.unitig_2.1168	An13g00610
evm.model.unitig_2.1169	An13g00620
evm.model.unitig_2.117	An01g13720
evm.model.unitig_2.1170	An13g00640
evm.model.unitig_2.1171	An13g00650
evm.model.unitig_2.1172	An13g00660
evm.model.unitig_2.1173	An13g00670
evm.model.unitig_2.1174	An13g00680
evm.model.unitig_2.1175	An13g00690
evm.model.unitig_2.1176	An13g00710
evm.model.unitig_2.1177	An13g00720
evm.model.unitig_2.1178	An13g00730
evm.model.unitig_2.1179	An13g00740
evm.model.unitig_2.118	An01g13690
evm.model.unitig_2.1180	An13g00750
evm.model.unitig_2.1181	An13g00760
evm.model.unitig_2.1182	An13g00770
evm.model.unitig_2.1183	An13g00780
evm.model.unitig_2.1184	An13g00790
evm.model.unitig_2.1185	An13g00793
evm.model.unitig_2.1186	An13g00800
evm.model.unitig_2.1187	An13g00810
evm.model.unitig_2.1188	An13g00840
evm.model.unitig_2.1189	An13g00850
evm.model.unitig_2.119	An01g13680
evm.model.unitig_2.1190	An13g00860
evm.model.unitig_2.1191	An13g00870
evm.model.unitig_2.1192	An13g00890
evm.model.unitig_2.1193	An13g00910
evm.model.unitig_2.1194	An13g00920
evm.model.unitig_2.1195	An13g00930
evm.model.unitig_2.1196	An13g00940
evm.model.unitig_2.1197	An13g00950
evm.model.unitig_2.1198	An13g00970

Gene ID of H915-1	Gene ID of 513.88
evm.model.unitig_2.1199	An13g00980
evm.model.unitig_2.12	An01g15100
evm.model.unitig_2.120	An01g13670
evm.model.unitig_2.1200	An13g01000
evm.model.unitig_2.1201	An13g01010
evm.model.unitig_2.1202	An13g01020
evm.model.unitig_2.1203	An13g01040
evm.model.unitig_2.1204	An13g01050
evm.model.unitig_2.1205	An13g01060
evm.model.unitig_2.1206	An13g01080
evm.model.unitig_2.1207	An13g01090
evm.model.unitig_2.1208	An13g01120
evm.model.unitig_2.1209	An13g01130
evm.model.unitig_2.121	An01g13660
evm.model.unitig_2.1210	An17g01925
evm.model.unitig_2.1211	An13g01150
evm.model.unitig_2.1212	An13g01150
evm.model.unitig_2.1213	An13g01180
evm.model.unitig_2.1214	An13g01190
evm.model.unitig_2.1215	An13g01200
evm.model.unitig_2.1218	An13g01210
evm.model.unitig_2.1219	An13g01220
evm.model.unitig_2.122	An01g13650
evm.model.unitig_2.1220	-
evm.model.unitig_2.1221	An13g01230
evm.model.unitig_2.1222	An13g01240
evm.model.unitig_2.1223	An12g05510
evm.model.unitig_2.1224	An13g01260
evm.model.unitig_2.1225	An13g01270
evm.model.unitig_2.1226	An13g01280
evm.model.unitig_2.1227	An13g01290
evm.model.unitig_2.1228	An13g01300
evm.model.unitig_2.1229	An13g01310
evm.model.unitig_2.123	An01g13640
evm.model.unitig_2.1230	-
evm.model.unitig_2.1231	An13g01320
evm.model.unitig_2.1232	An13g01340
evm.model.unitig_2.1233	An13g01350
evm.model.unitig_2.1234	An13g01370
evm.model.unitig_2.1235	An13g01420
evm.model.unitig_2.1236	-
evm.model.unitig_2.1237	An13g01440
evm.model.unitig_2.1238	An13g01450

Gene ID of H915-1	Gene ID of 513.88
evm.model.unitig_2.1239	An13g01460
evm.model.unitig_2.124	An01g13630
evm.model.unitig_2.1240	An13g01480
evm.model.unitig_2.1241	An13g01490
evm.model.unitig_2.1244	An13g01520
evm.model.unitig_2.1245	An13g01530
evm.model.unitig_2.1246	An13g01540
evm.model.unitig_2.1247	An13g01560
evm.model.unitig_2.1248	An13g01590
evm.model.unitig_2.1249	An13g01600
evm.model.unitig_2.125	An01g13620
evm.model.unitig_2.1250	An13g01610
evm.model.unitig_2.1251	An13g01620
evm.model.unitig_2.1252	An13g01640
evm.model.unitig_2.1253	An13g01680
evm.model.unitig_2.1254	An13g01690
evm.model.unitig_2.1255	An13g01700
evm.model.unitig_2.1256	An13g01730
evm.model.unitig_2.1257	An13g01750
evm.model.unitig_2.1258	An13g01760
evm.model.unitig_2.1259	An13g01780
evm.model.unitig_2.126	An01g13610
evm.model.unitig_2.1260	An13g01790
evm.model.unitig_2.1261	An13g01800
evm.model.unitig_2.1262	An13g01810
evm.model.unitig_2.1264	An13g01840
evm.model.unitig_2.1265	An13g01860
evm.model.unitig_2.1266	An13g01870
evm.model.unitig_2.1267	An13g01880
evm.model.unitig_2.1268	An13g01890
evm.model.unitig_2.1269	An13g01920
evm.model.unitig_2.127	An01g13600
evm.model.unitig_2.1270	An13g01930
evm.model.unitig_2.1271	An13g01940
evm.model.unitig_2.1272	An13g01960
evm.model.unitig_2.1273	An13g01980
evm.model.unitig_2.1275	An08g03080
evm.model.unitig_2.1276	An13g02000
evm.model.unitig_2.1277	An13g02020
evm.model.unitig_2.1278	An13g02030
evm.model.unitig_2.1279	An13g02050
evm.model.unitig_2.1280	An13g02060
evm.model.unitig_2.1281	An13g02070

Gene ID of H915-1	Gene ID of 513.88
evm.model.unitig_2.1282	An13g02080
evm.model.unitig_2.1283	An13g02090
evm.model.unitig_2.1284	An13g02100
evm.model.unitig_2.1285	An13g02130
evm.model.unitig_2.1286	An12g01700
evm.model.unitig_2.1287	An13g02160
evm.model.unitig_2.1288	An13g02180
evm.model.unitig_2.1289	An13g02220
evm.model.unitig_2.129	An01g13590
evm.model.unitig_2.1290	An13g02240
evm.model.unitig_2.1291	An13g02250
evm.model.unitig_2.1292	An13g02260
evm.model.unitig_2.1293	An13g02310
evm.model.unitig_2.1294	An14g03290
evm.model.unitig_2.1295	An13g02350
evm.model.unitig_2.1296	An13g02370
evm.model.unitig_2.1297	An13g02380
evm.model.unitig_2.1299	An13g02390
evm.model.unitig_2.13	An01g15070
evm.model.unitig_2.130	An01g13580
evm.model.unitig_2.1300	An13g02400
evm.model.unitig_2.1301	An13g02410
evm.model.unitig_2.1302	An13g02420
evm.model.unitig_2.1303	An13g02430
evm.model.unitig_2.1304	An13g02450
evm.model.unitig_2.1305	An13g02480
evm.model.unitig_2.1308	An13g02600
evm.model.unitig_2.1309	An13g02610
evm.model.unitig_2.131	An01g13560
evm.model.unitig_2.1310	An13g02620
evm.model.unitig_2.1311	An13g02640
evm.model.unitig_2.1312	An13g02680
evm.model.unitig_2.1313	An13g02690
evm.model.unitig_2.1314	An13g02780
evm.model.unitig_2.1315	An13g02790
evm.model.unitig_2.1316	An13g02790
evm.model.unitig_2.1317	An13g02820
evm.model.unitig_2.1318	An13g02880
evm.model.unitig_2.132	An01g13550
evm.model.unitig_2.1320	An08g03790
evm.model.unitig_2.1321	An13g02990
evm.model.unitig_2.1322	An13g03000
evm.model.unitig_2.1323	An13g03030

Gene ID of H915-1	Gene ID of 513.88
evm.model.unitig_2.1324	An13g03050
evm.model.unitig_2.1325	An13g03130
evm.model.unitig_2.1326	An13g03140
evm.model.unitig_2.1327	An13g03190
evm.model.unitig_2.1328	An13g03220
evm.model.unitig_2.1329	An13g03240
evm.model.unitig_2.133	An01g13540
evm.model.unitig_2.1330	An13g03250
evm.model.unitig_2.1331	An13g03260
evm.model.unitig_2.1333	An13g03290
evm.model.unitig_2.1334	An13g03300
evm.model.unitig_2.1335	An13g03310
evm.model.unitig_2.1336	An13g03360
evm.model.unitig_2.1337	An13g03370
evm.model.unitig_2.1338	An13g03380
evm.model.unitig_2.134	An01g13530
evm.model.unitig_2.1340	An13g03430
evm.model.unitig_2.1342	An13g03470
evm.model.unitig_2.1344	An13g03490
evm.model.unitig_2.1345	An13g03500
evm.model.unitig_2.1346	An08g12200
evm.model.unitig_2.1347	An13g03560
evm.model.unitig_2.1348	An13g03570
evm.model.unitig_2.1349	An13g03590
evm.model.unitig_2.135	An01g13510
evm.model.unitig_2.1351	An13g03600
evm.model.unitig_2.1352	An13g03610
evm.model.unitig_2.1353	An13g03640
evm.model.unitig_2.1354	An06g00160
evm.model.unitig_2.1355	An13g03680
evm.model.unitig_2.1356	An13g03690
evm.model.unitig_2.1357	An13g03710
evm.model.unitig_2.1358	-
evm.model.unitig_2.1359	An13g03800
evm.model.unitig_2.136	An01g13500
evm.model.unitig_2.1360	An13g03810
evm.model.unitig_2.1361	An13g03820
evm.model.unitig_2.1362	An13g03860
evm.model.unitig_2.1363	An13g03870
evm.model.unitig_2.1364	An13g03890
evm.model.unitig_2.1365	An13g03910
evm.model.unitig_2.1366	An13g03920
evm.model.unitig_2.1367	An13g03930

Gene ID of H915-1	Gene ID of 513.88
evm.model.unitig_2.1368	An13g03940
evm.model.unitig_2.1369	An13g03950
evm.model.unitig_2.137	An01g13490
evm.model.unitig_2.1370	An13g03970
evm.model.unitig_2.1371	An13g03980
evm.model.unitig_2.1372	An13g03990
evm.model.unitig_2.1373	An13g04000
evm.model.unitig_2.1374	An13g04000
evm.model.unitig_2.1376	An13g04030
evm.model.unitig_2.1377	An13g04060
evm.model.unitig_2.1378	An13g04070
evm.model.unitig_2.1379	An13g04080
evm.model.unitig_2.138	An01g13480
evm.model.unitig_2.1380	An09g01340
evm.model.unitig_2.1381	An19g00420
evm.model.unitig_2.1382	An19g00410
evm.model.unitig_2.1383	An19g00400
evm.model.unitig_2.1384	An19g00390
evm.model.unitig_2.1385	An19g00380
evm.model.unitig_2.1386	An19g00370
evm.model.unitig_2.1387	An02g01120
evm.model.unitig_2.1388	An19g00360
evm.model.unitig_2.1389	An19g00350
evm.model.unitig_2.139	An01g13470
evm.model.unitig_2.1390	An19g00340
evm.model.unitig_2.1391	An19g00330
evm.model.unitig_2.1392	An19g00320
evm.model.unitig_2.1393	An19g00300
evm.model.unitig_2.1394	An19g00290
evm.model.unitig_2.1395	An19g00280
evm.model.unitig_2.1396	An19g00270
evm.model.unitig_2.1397	An19g00240
evm.model.unitig_2.1398	An19g00230
evm.model.unitig_2.1399	An19g00230
evm.model.unitig_2.14	An01g15020
evm.model.unitig_2.140	An16g05270
evm.model.unitig_2.1400	An19g00210
evm.model.unitig_2.1401	-
evm.model.unitig_2.1402	An19g00180
evm.model.unitig_2.1403	An19g00170
evm.model.unitig_2.1404	An19g00160
evm.model.unitig_2.1405	-
evm.model.unitig_2.1406	An19g00140

Gene ID of H915-1	Gene ID of 513.88
evm.model.unitig_2.1407	An15g05440
evm.model.unitig_2.1408	An19g00120
evm.model.unitig_2.1409	An19g00110
evm.model.unitig_2.141	An01g13460
evm.model.unitig_2.1410	An19g00100
evm.model.unitig_2.1411	An19g00090
evm.model.unitig_2.1412	An19g00080
evm.model.unitig_2.1414	An19g00060
evm.model.unitig_2.1415	An19g00030
evm.model.unitig_2.1416	An19g00010
evm.model.unitig_2.1417	An11g09520
evm.model.unitig_2.1418	An15g04550
evm.model.unitig_2.1419	An03g00940
evm.model.unitig_2.142	An01g13450
evm.model.unitig_2.1420	An07g01380
evm.model.unitig_2.143	An01g13410
evm.model.unitig_2.144	An01g13400
evm.model.unitig_2.145	An01g13390
evm.model.unitig_2.146	An01g13370
evm.model.unitig_2.147	An01g13350
evm.model.unitig_2.148	An01g13340
evm.model.unitig_2.149	An01g13320
evm.model.unitig_2.15	An01g15010
evm.model.unitig_2.150	An01g13290
evm.model.unitig_2.151	An01g13280
evm.model.unitig_2.152	An01g13270
evm.model.unitig_2.153	An01g13260
evm.model.unitig_2.154	An01g13250
evm.model.unitig_2.155	An01g13240
evm.model.unitig_2.156	An01g13230
evm.model.unitig_2.157	An01g13220
evm.model.unitig_2.158	An01g13210
evm.model.unitig_2.159	An01g13200
evm.model.unitig_2.16	An01g15000
evm.model.unitig_2.160	An01g13190
evm.model.unitig_2.161	An01g13170
evm.model.unitig_2.162	An01g13160
evm.model.unitig_2.163	An01g13150
evm.model.unitig_2.164	An01g13130
evm.model.unitig_2.165	An01g13120
evm.model.unitig_2.166	An01g13100
evm.model.unitig_2.167	An01g13080
evm.model.unitig_2.168	An01g13070

Gene ID of H915-1	Gene ID of 513.88
evm.model.unitig_2.169	An01g13060
evm.model.unitig_2.17	An01g14990
evm.model.unitig_2.170	An01g13040
evm.model.unitig_2.171	An01g13030
evm.model.unitig_2.172	An01g13010
evm.model.unitig_2.173	An01g13000
evm.model.unitig_2.174	An01g12990
evm.model.unitig_2.175	An01g12970
evm.model.unitig_2.176	An01g12960
evm.model.unitig_2.177	An01g12940
evm.model.unitig_2.178	An01g12910
evm.model.unitig_2.18	An01g14980
evm.model.unitig_2.180	An01g12820
evm.model.unitig_2.181	An01g12810
evm.model.unitig_2.182	An01g12800
evm.model.unitig_2.183	An01g12780
evm.model.unitig_2.184	An01g12770
evm.model.unitig_2.185	An01g12760
evm.model.unitig_2.186	An01g12750
evm.model.unitig_2.187	An01g12740
evm.model.unitig_2.188	An01g12730
evm.model.unitig_2.189	An01g12720
evm.model.unitig_2.19	-
evm.model.unitig_2.190	An01g12710
evm.model.unitig_2.191	An01g12700
evm.model.unitig_2.192	An01g12690
evm.model.unitig_2.193	An01g12640
evm.model.unitig_2.194	An01g12630
evm.model.unitig_2.195	An01g12620
evm.model.unitig_2.196	An01g12610
evm.model.unitig_2.197	An01g12600
evm.model.unitig_2.198	An01g12590
evm.model.unitig_2.199	An01g12580
evm.model.unitig_2.2	An01g15190
evm.model.unitig_2.20	An01g14970
evm.model.unitig_2.200	An01g12570
evm.model.unitig_2.201	An01g12550
evm.model.unitig_2.202	An01g12530
evm.model.unitig_2.203	An01g12520
evm.model.unitig_2.204	An01g12500
evm.model.unitig_2.205	An01g12480
evm.model.unitig_2.206	An01g12480
evm.model.unitig_2.207	An01g12470

Gene ID of H915-1	Gene ID of 513.88
evm.model.unitig_2.208	An01g12460
evm.model.unitig_2.209	An01g12450
evm.model.unitig_2.21	An01g14960
evm.model.unitig_2.210	An01g12440
evm.model.unitig_2.211	An01g12430
evm.model.unitig_2.212	An01g12410
evm.model.unitig_2.213	An01g12400
evm.model.unitig_2.214	An01g12390
evm.model.unitig_2.215	An01g12380
evm.model.unitig_2.216	An01g12370
evm.model.unitig_2.217	An01g12360
evm.model.unitig_2.218	An01g12350
evm.model.unitig_2.219	An01g12330
evm.model.unitig_2.22	An01g14950
evm.model.unitig_2.220	An01g12320
evm.model.unitig_2.221	An01g12310
evm.model.unitig_2.222	An01g12300
evm.model.unitig_2.223	An01g12290
evm.model.unitig_2.224	An01g12270
evm.model.unitig_2.225	An01g12240
evm.model.unitig_2.226	An01g12230
evm.model.unitig_2.227	An01g12220
evm.model.unitig_2.228	An01g12210
evm.model.unitig_2.229	An01g12200
evm.model.unitig_2.23	An01g14940
evm.model.unitig_2.230	An01g12190
evm.model.unitig_2.231	An01g12180
evm.model.unitig_2.232	An01g12170
evm.model.unitig_2.233	An01g12150
evm.model.unitig_2.234	An01g12130
evm.model.unitig_2.235	An01g12120
evm.model.unitig_2.236	An01g12110
evm.model.unitig_2.237	An01g12100
evm.model.unitig_2.238	An01g12090
evm.model.unitig_2.239	An01g12090
evm.model.unitig_2.24	An01g14930
evm.model.unitig_2.240	An09g00640
evm.model.unitig_2.241	An01g12050
evm.model.unitig_2.242	An01g01130
evm.model.unitig_2.243	An15g02130
evm.model.unitig_2.244	An01g12020
evm.model.unitig_2.245	An01g12010
evm.model.unitig_2.246	An01g12000

Gene ID of H915-1	Gene ID of 513.88
evm.model.unitig_2.248	An01g11970
evm.model.unitig_2.249	An01g11960
evm.model.unitig_2.25	An01g14920
evm.model.unitig_2.250	An01g11950
evm.model.unitig_2.251	An01g11940
evm.model.unitig_2.252	An01g11930
evm.model.unitig_2.253	An01g11920
evm.model.unitig_2.254	An01g11910
evm.model.unitig_2.255	An01g11900
evm.model.unitig_2.256	-
evm.model.unitig_2.257	An01g11880
evm.model.unitig_2.258	An01g11880
evm.model.unitig_2.259	An01g11860
evm.model.unitig_2.26	An01g14910
evm.model.unitig_2.260	-
evm.model.unitig_2.261	An01g11820
evm.model.unitig_2.262	An01g11810
evm.model.unitig_2.263	An01g11800
evm.model.unitig_2.264	An01g11790
evm.model.unitig_2.265	An01g11780
evm.model.unitig_2.266	An05g01060
evm.model.unitig_2.267	An01g11760
evm.model.unitig_2.268	An01g11750
evm.model.unitig_2.269	An01g11740
evm.model.unitig_2.27	An01g14900
evm.model.unitig_2.270	An01g11720
evm.model.unitig_2.272	An01g11690
evm.model.unitig_2.273	An01g11680
evm.model.unitig_2.274	An01g11670
evm.model.unitig_2.275	An01g11660
evm.model.unitig_2.276	An01g11650
evm.model.unitig_2.277	An01g11640
evm.model.unitig_2.278	-
evm.model.unitig_2.279	An01g11620
evm.model.unitig_2.28	An01g14890
evm.model.unitig_2.280	An01g11610
evm.model.unitig_2.281	An01g11600
evm.model.unitig_2.282	An01g11580
evm.model.unitig_2.283	An01g11560
evm.model.unitig_2.284	An01g11560
evm.model.unitig_2.285	An01g11550
evm.model.unitig_2.286	An01g11540
evm.model.unitig_2.287	An01g11530

Gene ID of H915-1	Gene ID of 513.88
evm.model.unitig_2.288	An01g11520
evm.model.unitig_2.289	An01g11510
evm.model.unitig_2.29	An01g14880
evm.model.unitig_2.291	An16g04050
evm.model.unitig_2.292	An01g11480
evm.model.unitig_2.293	An01g11470
evm.model.unitig_2.294	An01g11450
evm.model.unitig_2.295	An01g11440
evm.model.unitig_2.296	An01g11430
evm.model.unitig_2.297	An01g11420
evm.model.unitig_2.298	An01g11410
evm.model.unitig_2.299	An01g11400
evm.model.unitig_2.3	An08g06440
evm.model.unitig_2.30	An01g14870
evm.model.unitig_2.300	An01g11390
evm.model.unitig_2.301	An01g11380
evm.model.unitig_2.302	An01g11360
evm.model.unitig_2.303	An01g11330
evm.model.unitig_2.304	An01g11310
evm.model.unitig_2.305	An01g11290
evm.model.unitig_2.306	An01g11270
evm.model.unitig_2.307	An01g11260
evm.model.unitig_2.308	An01g11250
evm.model.unitig_2.309	-
evm.model.unitig_2.31	An01g14860
evm.model.unitig_2.310	An01g11200
evm.model.unitig_2.311	An01g11190
evm.model.unitig_2.312	An01g11150
evm.model.unitig_2.313	An01g11140
evm.model.unitig_2.314	An01g11130
evm.model.unitig_2.315	An01g11100
evm.model.unitig_2.316	An01g11080
evm.model.unitig_2.317	An01g11010
evm.model.unitig_2.318	An01g11000
evm.model.unitig_2.319	An01g10990
evm.model.unitig_2.32	An01g14840
evm.model.unitig_2.320	An01g10980
evm.model.unitig_2.321	An01g10970
evm.model.unitig_2.322	An01g10960
evm.model.unitig_2.323	An01g10950
evm.model.unitig_2.324	An01g10930
evm.model.unitig_2.325	An01g10920
evm.model.unitig_2.326	An01g10910

Gene ID of H915-1	Gene ID of 513.88
evm.model.unitig_2.327	An01g10900
evm.model.unitig_2.328	An01g10880
evm.model.unitig_2.329	An01g10870
evm.model.unitig_2.33	An01g14820
evm.model.unitig_2.330	An01g10860
evm.model.unitig_2.331	An01g10850
evm.model.unitig_2.332	An01g10840
evm.model.unitig_2.333	An01g10820
evm.model.unitig_2.334	An01g10810
evm.model.unitig_2.335	An01g10800
evm.model.unitig_2.336	An01g10790
evm.model.unitig_2.337	An01g10760
evm.model.unitig_2.338	An01g10750
evm.model.unitig_2.339	An01g10740
evm.model.unitig_2.340	An01g10730
evm.model.unitig_2.341	-
evm.model.unitig_2.342	An01g10700
evm.model.unitig_2.343	An01g10690
evm.model.unitig_2.344	An01g10680
evm.model.unitig_2.345	An01g10660
evm.model.unitig_2.346	An01g10650
evm.model.unitig_2.347	An01g10640
evm.model.unitig_2.348	An01g10620
evm.model.unitig_2.349	An01g10610
evm.model.unitig_2.35	An01g14800
evm.model.unitig_2.350	An01g10600
evm.model.unitig_2.351	An01g10580
evm.model.unitig_2.352	An01g10570
evm.model.unitig_2.353	An01g10540
evm.model.unitig_2.354	An01g10490
evm.model.unitig_2.355	An01g10460
evm.model.unitig_2.357	An01g10380
evm.model.unitig_2.358	An01g10360
evm.model.unitig_2.359	An01g10350
evm.model.unitig_2.360	An01g10340
evm.model.unitig_2.361	An01g10320
evm.model.unitig_2.362	An01g10310
evm.model.unitig_2.363	An01g10290
evm.model.unitig_2.364	An07g00050
evm.model.unitig_2.365	An01g10270
evm.model.unitig_2.367	An01g10240
evm.model.unitig_2.368	An01g10200
evm.model.unitig_2.369	An01g10190

Gene ID of H915-1	Gene ID of 513.88
evm.model.unitig_2.37	An01g14790
evm.model.unitig_2.370	An01g10170
evm.model.unitig_2.371	An01g10150
evm.model.unitig_2.372	An01g10140
evm.model.unitig_2.374	An01g10100
evm.model.unitig_2.375	An01g10060
evm.model.unitig_2.376	An01g10050
evm.model.unitig_2.377	An01g10030
evm.model.unitig_2.379	An01g10010
evm.model.unitig_2.38	An01g14780
evm.model.unitig_2.380	An01g10000
evm.model.unitig_2.381	An01g09980
evm.model.unitig_2.382	An01g09960
evm.model.unitig_2.383	An01g09950
evm.model.unitig_2.384	An01g09940
evm.model.unitig_2.385	An01g09930
evm.model.unitig_2.386	An01g09920
evm.model.unitig_2.387	An01g09910
evm.model.unitig_2.388	An01g09900
evm.model.unitig_2.389	An01g09890
evm.model.unitig_2.39	An01g14770
evm.model.unitig_2.390	An01g09880
evm.model.unitig_2.391	An01g09870
evm.model.unitig_2.392	An01g09860
evm.model.unitig_2.393	An01g09850
evm.model.unitig_2.394	An01g09830
evm.model.unitig_2.395	An01g09820
evm.model.unitig_2.396	An01g09810
evm.model.unitig_2.397	An01g09800
evm.model.unitig_2.398	An01g09780
evm.model.unitig_2.399	An01g09770
evm.model.unitig_2.4	An18g04130
evm.model.unitig_2.40	An01g14760
evm.model.unitig_2.400	An01g09760
evm.model.unitig_2.401	An01g09750
evm.model.unitig_2.402	An01g09740
evm.model.unitig_2.403	An01g09730
evm.model.unitig_2.404	An01g09650
evm.model.unitig_2.406	An01g09640
evm.model.unitig_2.407	An01g09630
evm.model.unitig_2.408	An01g09620
evm.model.unitig_2.409	An01g09610
evm.model.unitig_2.41	An01g14740

Gene ID of H915-1	Gene ID of 513.88
evm.model.unitig_2.411	An01g09590
evm.model.unitig_2.412	An01g09580
evm.model.unitig_2.413	An01g09570
evm.model.unitig_2.414	An01g09560
evm.model.unitig_2.415	An01g09550
evm.model.unitig_2.416	An01g09540
evm.model.unitig_2.417	An01g09530
evm.model.unitig_2.418	An01g09520
evm.model.unitig_2.419	An01g09510
evm.model.unitig_2.42	An01g14730
evm.model.unitig_2.420	An01g09500
evm.model.unitig_2.421	An01g09480
evm.model.unitig_2.422	An01g09460
evm.model.unitig_2.423	An01g09450
evm.model.unitig_2.424	An01g09410
evm.model.unitig_2.425	An01g09400
evm.model.unitig_2.426	An01g09390
evm.model.unitig_2.427	An01g09380
evm.model.unitig_2.428	An01g09350
evm.model.unitig_2.429	An01g09330
evm.model.unitig_2.43	An01g14720
evm.model.unitig_2.430	An01g09320
evm.model.unitig_2.431	An01g09310
evm.model.unitig_2.432	An01g09300
evm.model.unitig_2.433	An01g09290
evm.model.unitig_2.434	An01g09270
evm.model.unitig_2.435	An01g09260
evm.model.unitig_2.436	An01g09250
evm.model.unitig_2.437	An01g09240
evm.model.unitig_2.438	An01g09220
evm.model.unitig_2.439	An01g09210
evm.model.unitig_2.44	An01g14710
evm.model.unitig_2.440	An01g09190
evm.model.unitig_2.441	An01g09180
evm.model.unitig_2.442	An01g09170
evm.model.unitig_2.443	An01g09160
evm.model.unitig_2.444	An01g09160
evm.model.unitig_2.445	An01g09130
evm.model.unitig_2.446	An01g09130
evm.model.unitig_2.447	An01g09120
evm.model.unitig_2.448	An01g09110
evm.model.unitig_2.449	An01g09100
evm.model.unitig_2.45	An01g14690

Gene ID of H915-1	Gene ID of 513.88
evm.model.unitig_2.450	An01g09090
evm.model.unitig_2.451	An07g06910
evm.model.unitig_2.452	An01g09050
evm.model.unitig_2.453	An01g09040
evm.model.unitig_2.454	An01g09030
evm.model.unitig_2.455	An01g09020
evm.model.unitig_2.456	An01g09010
evm.model.unitig_2.457	An01g09000
evm.model.unitig_2.458	An01g08990
evm.model.unitig_2.459	An01g08980
evm.model.unitig_2.46	An01g14680
evm.model.unitig_2.460	An01g08970
evm.model.unitig_2.461	An01g08960
evm.model.unitig_2.462	An01g08950
evm.model.unitig_2.463	An01g08950
evm.model.unitig_2.464	An01g08930
evm.model.unitig_2.465	An01g08920
evm.model.unitig_2.466	An01g08910
evm.model.unitig_2.467	An01g08900
evm.model.unitig_2.468	An01g08890
evm.model.unitig_2.469	An01g08880
evm.model.unitig_2.47	An01g14670
evm.model.unitig_2.470	An01g08870
evm.model.unitig_2.471	An01g08860
evm.model.unitig_2.472	An01g08850
evm.model.unitig_2.473	An01g08840
evm.model.unitig_2.474	An01g08830
evm.model.unitig_2.475	-
evm.model.unitig_2.476	-
evm.model.unitig_2.477	An01g08790
evm.model.unitig_2.478	An01g08780
evm.model.unitig_2.479	-
evm.model.unitig_2.48	An01g14660
evm.model.unitig_2.480	An01g08730
evm.model.unitig_2.481	An01g08720
evm.model.unitig_2.482	An01g08700
evm.model.unitig_2.483	An01g08690
evm.model.unitig_2.484	An01g08670
evm.model.unitig_2.485	An01g08660
evm.model.unitig_2.486	An01g08650
evm.model.unitig_2.487	An01g08630
evm.model.unitig_2.488	An01g08620
evm.model.unitig_2.489	An01g08610

Gene ID of H915-1	Gene ID of 513.88
evm.model.unitig_2.49	An01g14650
evm.model.unitig_2.490	An01g08600
evm.model.unitig_2.491	An01g08590
evm.model.unitig_2.492	An01g08580
evm.model.unitig_2.493	An01g08570
evm.model.unitig_2.494	An01g08560
evm.model.unitig_2.495	An01g08550
evm.model.unitig_2.496	An01g08540
evm.model.unitig_2.497	An01g08530
evm.model.unitig_2.498	An01g08520
evm.model.unitig_2.499	An01g08500
evm.model.unitig_2.5	An12g02770
evm.model.unitig_2.50	An01g14640
evm.model.unitig_2.500	An01g08490
evm.model.unitig_2.501	An01g08470
evm.model.unitig_2.502	An01g08460
evm.model.unitig_2.503	An01g08450
evm.model.unitig_2.504	An01g08440
evm.model.unitig_2.505	An01g08430
evm.model.unitig_2.506	An01g08420
evm.model.unitig_2.507	An01g08410
evm.model.unitig_2.508	An01g08400
evm.model.unitig_2.509	An01g08390
evm.model.unitig_2.51	An01g14620
evm.model.unitig_2.510	An01g08380
evm.model.unitig_2.511	An01g08310
evm.model.unitig_2.512	An01g08280
evm.model.unitig_2.513	An01g08240
evm.model.unitig_2.514	An01g08230
evm.model.unitig_2.515	An01g08220
evm.model.unitig_2.516	An01g08210
evm.model.unitig_2.517	An01g08180
evm.model.unitig_2.518	An01g08170
evm.model.unitig_2.519	An01g08160
evm.model.unitig_2.52	An01g14600
evm.model.unitig_2.520	An01g08150
evm.model.unitig_2.521	An01g08130
evm.model.unitig_2.522	An01g08120
evm.model.unitig_2.523	An01g08110
evm.model.unitig_2.524	An01g08100
evm.model.unitig_2.525	An01g08090
evm.model.unitig_2.526	An01g08080
evm.model.unitig_2.527	An01g08060

Gene ID of H915-1	Gene ID of 513.88
evm.model.unitig_2.528	An01g08050
evm.model.unitig_2.529	An01g08040
evm.model.unitig_2.53	An01g14590
evm.model.unitig_2.530	An01g08030
evm.model.unitig_2.531	An01g08020
evm.model.unitig_2.532	An01g08010
evm.model.unitig_2.533	An01g08000
evm.model.unitig_2.534	An01g07990
evm.model.unitig_2.535	An01g07980
evm.model.unitig_2.536	An06g02150
evm.model.unitig_2.537	An01g11330
evm.model.unitig_2.538	An11g07050
evm.model.unitig_2.539	An01g07950
evm.model.unitig_2.54	An01g14560
evm.model.unitig_2.540	An01g07920
evm.model.unitig_2.541	An01g07910
evm.model.unitig_2.543	An01g07870
evm.model.unitig_2.544	An01g07860
evm.model.unitig_2.545	An01g07850
evm.model.unitig_2.546	An01g07840
evm.model.unitig_2.547	An01g07830
evm.model.unitig_2.548	An01g07820
evm.model.unitig_2.549	An01g07810
evm.model.unitig_2.550	An01g07790
evm.model.unitig_2.551	An01g07770
evm.model.unitig_2.552	An01g07760
evm.model.unitig_2.553	An01g07750
evm.model.unitig_2.554	An09g02340
evm.model.unitig_2.555	An01g07720
evm.model.unitig_2.556	An01g07700
evm.model.unitig_2.557	An01g07680
evm.model.unitig_2.558	An01g07660
evm.model.unitig_2.559	An01g07650
evm.model.unitig_2.56	An01g14550
evm.model.unitig_2.560	An01g07640
evm.model.unitig_2.561	An01g07630
evm.model.unitig_2.562	An01g07600
evm.model.unitig_2.563	An01g07590
evm.model.unitig_2.564	An01g07580
evm.model.unitig_2.565	An01g07570
evm.model.unitig_2.566	An01g07550
evm.model.unitig_2.567	An01g07530
evm.model.unitig_2.568	An01g07520

Gene ID of H915-1	Gene ID of 513.88
evm.model.unitig_2.569	An01g07510
evm.model.unitig_2.57	An01g14540
evm.model.unitig_2.570	An01g07500
evm.model.unitig_2.571	An01g07480
evm.model.unitig_2.572	An01g07470
evm.model.unitig_2.573	An01g07460
evm.model.unitig_2.574	An12g02210
evm.model.unitig_2.575	An01g07430
evm.model.unitig_2.576	An01g07420
evm.model.unitig_2.577	An01g07400
evm.model.unitig_2.578	An01g07390
evm.model.unitig_2.579	An01g07380
evm.model.unitig_2.58	An01g14540
evm.model.unitig_2.580	An01g07370
evm.model.unitig_2.581	An01g07360
evm.model.unitig_2.582	An01g07350
evm.model.unitig_2.583	An01g07330
evm.model.unitig_2.584	An01g07320
evm.model.unitig_2.585	An01g07300
evm.model.unitig_2.586	An01g07290
evm.model.unitig_2.587	An01g07280
evm.model.unitig_2.588	An01g07270
evm.model.unitig_2.589	An01g07260
evm.model.unitig_2.59	An01g14520
evm.model.unitig_2.590	An01g07240
evm.model.unitig_2.591	An01g07230
evm.model.unitig_2.592	An01g07220
evm.model.unitig_2.593	An01g07200
evm.model.unitig_2.594	An01g07190
evm.model.unitig_2.595	An01g07180
evm.model.unitig_2.596	An01g07170
evm.model.unitig_2.597	An01g07160
evm.model.unitig_2.598	An01g07150
evm.model.unitig_2.599	An01g07140
evm.model.unitig_2.60	An01g14510
evm.model.unitig_2.600	An01g07110
evm.model.unitig_2.601	An01g07090
evm.model.unitig_2.602	An01g07080
evm.model.unitig_2.603	-
evm.model.unitig_2.604	An01g07060
evm.model.unitig_2.605	An01g07050
evm.model.unitig_2.606	An01g07040
evm.model.unitig_2.607	An01g07030

Gene ID of H915-1	Gene ID of 513.88
evm.model.unitig_2.608	An01g07025
evm.model.unitig_2.609	An01g07010
evm.model.unitig_2.610	An01g07000
evm.model.unitig_2.611	An01g06990
evm.model.unitig_2.612	An01g06970
evm.model.unitig_2.613	An01g06960
evm.model.unitig_2.614	An01g06930
evm.model.unitig_2.615	An01g06930
evm.model.unitig_2.616	An01g06920
evm.model.unitig_2.617	An01g06910
evm.model.unitig_2.618	An01g06880
evm.model.unitig_2.619	An01g06870
evm.model.unitig_2.62	An01g14480
evm.model.unitig_2.620	An01g06860
evm.model.unitig_2.621	An01g06850
evm.model.unitig_2.622	An01g06830
evm.model.unitig_2.623	An01g06820
evm.model.unitig_2.624	An01g06810
evm.model.unitig_2.625	An01g06800
evm.model.unitig_2.627	An01g06790
evm.model.unitig_2.628	An01g06780
evm.model.unitig_2.629	An01g06770
evm.model.unitig_2.63	An01g14450
evm.model.unitig_2.630	An08g03810
evm.model.unitig_2.631	An01g06750
evm.model.unitig_2.633	An01g06710
evm.model.unitig_2.634	An01g06690
evm.model.unitig_2.635	An01g06670
evm.model.unitig_2.636	An01g06660
evm.model.unitig_2.637	An01g06650
evm.model.unitig_2.638	An01g06640
evm.model.unitig_2.639	An01g06630
evm.model.unitig_2.64	An01g14430
evm.model.unitig_2.640	An01g06620
evm.model.unitig_2.641	An01g06610
evm.model.unitig_2.642	An01g06570
evm.model.unitig_2.643	An01g06560
evm.model.unitig_2.644	An01g06550
evm.model.unitig_2.645	An01g06530
evm.model.unitig_2.646	An01g06520
evm.model.unitig_2.647	An01g06500
evm.model.unitig_2.648	An01g06480
evm.model.unitig_2.649	An01g06470

Gene ID of H915-1	Gene ID of 513.88
evm.model.unitig_2.65	An01g14420
evm.model.unitig_2.650	An01g06460
evm.model.unitig_2.651	An01g06440
evm.model.unitig_2.652	An01g06420
evm.model.unitig_2.653	An01g06410
evm.model.unitig_2.654	An01g06400
evm.model.unitig_2.655	An01g06390
evm.model.unitig_2.656	An01g06380
evm.model.unitig_2.657	An01g06370
evm.model.unitig_2.658	An01g06360
evm.model.unitig_2.659	An01g06350
evm.model.unitig_2.66	An01g14410
evm.model.unitig_2.660	An01g06340
evm.model.unitig_2.661	An01g06330
evm.model.unitig_2.662	An01g06310
evm.model.unitig_2.663	An01g06290
evm.model.unitig_2.664	An01g06280
evm.model.unitig_2.665	An01g06270
evm.model.unitig_2.666	An01g06260
evm.model.unitig_2.667	An01g06250
evm.model.unitig_2.668	An01g06240
evm.model.unitig_2.669	An01g06230
evm.model.unitig_2.67	An01g14400
evm.model.unitig_2.670	An08g05010
evm.model.unitig_2.671	An01g06210
evm.model.unitig_2.672	An01g06190
evm.model.unitig_2.673	An01g06180
evm.model.unitig_2.674	An01g06170
evm.model.unitig_2.675	An01g06150
evm.model.unitig_2.676	An01g06120
evm.model.unitig_2.677	An01g06120
evm.model.unitig_2.678	An01g06110
evm.model.unitig_2.679	An01g06080
evm.model.unitig_2.68	An01g14390
evm.model.unitig_2.680	An01g06080
evm.model.unitig_2.681	An01g06070
evm.model.unitig_2.682	An01g06060
evm.model.unitig_2.683	An01g06050
evm.model.unitig_2.684	An01g06040
evm.model.unitig_2.685	An01g06030
evm.model.unitig_2.686	An01g06020
evm.model.unitig_2.687	An01g06010
evm.model.unitig_2.688	An01g05990

Gene ID of H915-1	Gene ID of 513.88
evm.model.unitig_2.689	An01g05970
evm.model.unitig_2.69	An01g14380
evm.model.unitig_2.690	An01g05960
evm.model.unitig_2.691	An01g05920
evm.model.unitig_2.692	An01g05900
evm.model.unitig_2.693	An01g05890
evm.model.unitig_2.694	An01g05860
evm.model.unitig_2.695	An01g05850
evm.model.unitig_2.696	An01g05840
evm.model.unitig_2.697	An03g04730
evm.model.unitig_2.698	An01g05830
evm.model.unitig_2.699	An01g05820
evm.model.unitig_2.7	An01g15140
evm.model.unitig_2.70	An01g14380
evm.model.unitig_2.700	An01g05810
evm.model.unitig_2.701	An01g05800
evm.model.unitig_2.702	An01g05790
evm.model.unitig_2.703	An01g05780
evm.model.unitig_2.704	An01g05770
evm.model.unitig_2.705	An01g05760
evm.model.unitig_2.706	An01g05750
evm.model.unitig_2.707	An01g05740
evm.model.unitig_2.708	An01g05700
evm.model.unitig_2.709	An01g05680
evm.model.unitig_2.71	An01g14360
evm.model.unitig_2.710	An01g05670
evm.model.unitig_2.711	An01g05660
evm.model.unitig_2.712	An01g05650
evm.model.unitig_2.713	An01g05640
evm.model.unitig_2.714	An01g05630
evm.model.unitig_2.715	An01g05620
evm.model.unitig_2.716	An01g05610
evm.model.unitig_2.717	An01g05600
evm.model.unitig_2.718	An01g05580
evm.model.unitig_2.719	An01g05570
evm.model.unitig_2.72	An01g14330
evm.model.unitig_2.720	An01g05540
evm.model.unitig_2.721	-
evm.model.unitig_2.722	An01g05510
evm.model.unitig_2.723	An01g05500
evm.model.unitig_2.724	An01g05470
evm.model.unitig_2.725	An01g05410
evm.model.unitig_2.726	An01g05390

Gene ID of H915-1	Gene ID of 513.88
evm.model.unitig_2.727	An01g05370
evm.model.unitig_2.728	An01g05360
evm.model.unitig_2.73	An01g14310
evm.model.unitig_2.730	An01g05350
evm.model.unitig_2.731	An01g05340
evm.model.unitig_2.732	An01g05330
evm.model.unitig_2.733	An01g05320
evm.model.unitig_2.734	An01g05310
evm.model.unitig_2.735	An01g05300
evm.model.unitig_2.736	-
evm.model.unitig_2.737	An01g05290
evm.model.unitig_2.738	An01g05280
evm.model.unitig_2.739	An01g05270
evm.model.unitig_2.74	An01g14290
evm.model.unitig_2.740	An01g05260
evm.model.unitig_2.741	An01g05230
evm.model.unitig_2.742	An01g05220
evm.model.unitig_2.743	An01g05190
evm.model.unitig_2.744	-
evm.model.unitig_2.745	An01g05180
evm.model.unitig_2.746	An01g05160
evm.model.unitig_2.747	An01g05150
evm.model.unitig_2.748	An01g05100
evm.model.unitig_2.749	An01g05090
evm.model.unitig_2.75	An01g14280
evm.model.unitig_2.750	An01g05080
evm.model.unitig_2.751	An01g05070
evm.model.unitig_2.752	An01g05060
evm.model.unitig_2.753	An01g05050
evm.model.unitig_2.754	An01g05040
evm.model.unitig_2.755	An01g05030
evm.model.unitig_2.756	An01g04990
evm.model.unitig_2.757	An01g04980
evm.model.unitig_2.758	An01g04970
evm.model.unitig_2.759	An01g04950
evm.model.unitig_2.76	An01g14250
evm.model.unitig_2.761	An01g04930
evm.model.unitig_2.762	An01g04910
evm.model.unitig_2.763	An01g04900
evm.model.unitig_2.764	An01g04880
evm.model.unitig_2.765	An01g04830
evm.model.unitig_2.768	An01g04760
evm.model.unitig_2.769	An01g04740

Gene ID of H915-1	Gene ID of 513.88
evm.model.unitig_2.77	An01g14230
evm.model.unitig_2.770	An01g04730
evm.model.unitig_2.771	An01g04720
evm.model.unitig_2.772	An01g04710
evm.model.unitig_2.773	An01g04700
evm.model.unitig_2.774	An01g04690
evm.model.unitig_2.775	An01g04680
evm.model.unitig_2.776	An01g04670
evm.model.unitig_2.777	An01g04660
evm.model.unitig_2.778	An01g04650
evm.model.unitig_2.779	An01g04640
evm.model.unitig_2.78	An01g14210
evm.model.unitig_2.780	An01g04630
evm.model.unitig_2.781	An01g04620
evm.model.unitig_2.782	An01g04610
evm.model.unitig_2.783	An01g04600
evm.model.unitig_2.784	An01g04590
evm.model.unitig_2.785	An01g04570
evm.model.unitig_2.786	An01g04560
evm.model.unitig_2.787	An01g04550
evm.model.unitig_2.788	An01g04530
evm.model.unitig_2.789	An01g04520
evm.model.unitig_2.79	An01g14200
evm.model.unitig_2.790	An01g04470
evm.model.unitig_2.792	An01g04450
evm.model.unitig_2.793	An01g04440
evm.model.unitig_2.794	An01g04430
evm.model.unitig_2.795	An01g04420
evm.model.unitig_2.796	An01g04410
evm.model.unitig_2.797	An01g04400
evm.model.unitig_2.798	An01g04380
evm.model.unitig_2.799	An01g04370
evm.model.unitig_2.8	An01g15130
evm.model.unitig_2.80	An01g14180
evm.model.unitig_2.800	An01g04360
evm.model.unitig_2.801	An01g04330
evm.model.unitig_2.802	An01g04320
evm.model.unitig_2.803	An01g04310
evm.model.unitig_2.804	An01g04300
evm.model.unitig_2.805	An01g04280
evm.model.unitig_2.806	An01g04260
evm.model.unitig_2.807	An01g04250
evm.model.unitig_2.808	An01g04240

Gene ID of H915-1	Gene ID of 513.88
evm.model.unitig_2.809	An01g04230
evm.model.unitig_2.81	An01g14160
evm.model.unitig_2.810	An15g04600
evm.model.unitig_2.811	An01g04100
evm.model.unitig_2.812	An01g04090
evm.model.unitig_2.813	An01g04080
evm.model.unitig_2.814	An01g04060
evm.model.unitig_2.815	An01g04180
evm.model.unitig_2.816	An01g04150
evm.model.unitig_2.817	An12g02980
evm.model.unitig_2.818	An01g04040
evm.model.unitig_2.819	An01g04030
evm.model.unitig_2.82	An01g14140
evm.model.unitig_2.820	An01g03970
evm.model.unitig_2.821	An01g03950
evm.model.unitig_2.822	An15g04620
evm.model.unitig_2.823	An01g03950
evm.model.unitig_2.824	An01g03900
evm.model.unitig_2.825	An01g03870
evm.model.unitig_2.826	An01g03820
evm.model.unitig_2.827	An01g03790
evm.model.unitig_2.828	An01g03780
evm.model.unitig_2.829	An01g03760
evm.model.unitig_2.83	An01g14130
evm.model.unitig_2.830	An01g03750
evm.model.unitig_2.831	An01g03740
evm.model.unitig_2.832	An01g03720
evm.model.unitig_2.833	An01g03700
evm.model.unitig_2.834	An01g03680
evm.model.unitig_2.835	An01g03670
evm.model.unitig_2.836	An01g03660
evm.model.unitig_2.837	An01g03650
evm.model.unitig_2.838	An01g03630
evm.model.unitig_2.839	An01g03610
evm.model.unitig_2.84	An01g14120
evm.model.unitig_2.840	An01g03600
evm.model.unitig_2.841	An01g03590
evm.model.unitig_2.842	An01g03580
evm.model.unitig_2.843	An01g03570
evm.model.unitig_2.844	An01g03560
evm.model.unitig_2.845	An01g03550
evm.model.unitig_2.846	An01g03540
evm.model.unitig_2.847	An01g03530

Gene ID of H915-1	Gene ID of 513.88
evm.model.unitig_2.848	An01g03520
evm.model.unitig_2.849	An01g03510
evm.model.unitig_2.85	An01g14110
evm.model.unitig_2.850	An01g03500
evm.model.unitig_2.851	An01g03490
evm.model.unitig_2.852	An01g03480
evm.model.unitig_2.853	An01g03470
evm.model.unitig_2.854	An01g03460
evm.model.unitig_2.855	An01g03450
evm.model.unitig_2.856	An01g03410
evm.model.unitig_2.857	An12g05490
evm.model.unitig_2.858	An01g03390
evm.model.unitig_2.859	An01g03370
evm.model.unitig_2.86	An01g14100
evm.model.unitig_2.860	-
evm.model.unitig_2.861	An01g03360
evm.model.unitig_2.862	An01g03350
evm.model.unitig_2.863	An01g03340
evm.model.unitig_2.864	An01g03330
evm.model.unitig_2.865	An01g03310
evm.model.unitig_2.866	An01g03300
evm.model.unitig_2.867	-
evm.model.unitig_2.868	An01g03270
evm.model.unitig_2.869	An01g03250
evm.model.unitig_2.87	An01g14090
evm.model.unitig_2.870	An01g03240
evm.model.unitig_2.871	An01g03230
evm.model.unitig_2.872	An01g03220
evm.model.unitig_2.873	An01g03210
evm.model.unitig_2.874	An01g03190
evm.model.unitig_2.875	An01g03180
evm.model.unitig_2.876	An01g03170
evm.model.unitig_2.877	An01g03160
evm.model.unitig_2.878	An01g03150
evm.model.unitig_2.879	An01g03140
evm.model.unitig_2.88	An01g14080
evm.model.unitig_2.880	An01g03130
evm.model.unitig_2.881	An01g03120
evm.model.unitig_2.882	An01g03100
evm.model.unitig_2.883	An01g03090
evm.model.unitig_2.884	An01g03080
evm.model.unitig_2.885	An01g03070
evm.model.unitig_2.886	An01g03060

Gene ID of H915-1	Gene ID of 513.88
evm.model.unitig_2.887	An01g03050
evm.model.unitig_2.888	An01g03040
evm.model.unitig_2.889	An01g03030
evm.model.unitig_2.89	An01g14070
evm.model.unitig_2.890	An01g03000
evm.model.unitig_2.891	An01g02990
evm.model.unitig_2.892	An04g00410
evm.model.unitig_2.893	An01g02970
evm.model.unitig_2.894	An01g02960
evm.model.unitig_2.895	An01g02950
evm.model.unitig_2.896	An01g02940
evm.model.unitig_2.897	An01g02930
evm.model.unitig_2.898	An01g02910
evm.model.unitig_2.899	An01g02900
evm.model.unitig_2.9	-
evm.model.unitig_2.90	An01g14050
evm.model.unitig_2.900	An01g02890
evm.model.unitig_2.901	An01g02870
evm.model.unitig_2.902	An01g02860
evm.model.unitig_2.903	An01g02850
evm.model.unitig_2.904	An01g02840
evm.model.unitig_2.905	An01g02830
evm.model.unitig_2.906	An01g02810
evm.model.unitig_2.907	An01g02800
evm.model.unitig_2.908	An01g02790
evm.model.unitig_2.909	An01g02760
evm.model.unitig_2.91	An01g14040
evm.model.unitig_2.910	An15g03540
evm.model.unitig_2.911	An01g02740
evm.model.unitig_2.912	An01g02730
evm.model.unitig_2.913	An01g02720
evm.model.unitig_2.914	An01g02700
evm.model.unitig_2.915	An01g02690
evm.model.unitig_2.916	An01g02680
evm.model.unitig_2.917	An01g02670
evm.model.unitig_2.918	An01g02660
evm.model.unitig_2.919	An03g05810
evm.model.unitig_2.92	An01g14030
evm.model.unitig_2.920	An01g02640
evm.model.unitig_2.921	An01g02630
evm.model.unitig_2.922	An01g02620
evm.model.unitig_2.923	An01g02600
evm.model.unitig_2.924	An01g02510

Gene ID of H915-1	Gene ID of 513.88
evm.model.unitig_2.925	An01g02500
evm.model.unitig_2.926	An01g02460
evm.model.unitig_2.927	An01g02440
evm.model.unitig_2.928	An01g02430
evm.model.unitig_2.929	An01g02420
evm.model.unitig_2.93	An01g14020
evm.model.unitig_2.930	An01g02410
evm.model.unitig_2.931	An01g02370
evm.model.unitig_2.932	An01g02360
evm.model.unitig_2.933	An01g02340
evm.model.unitig_2.934	An01g02330
evm.model.unitig_2.935	An01g02320
evm.model.unitig_2.936	An01g02310
evm.model.unitig_2.937	An01g02280
evm.model.unitig_2.939	An01g02260
evm.model.unitig_2.94	An01g14010
evm.model.unitig_2.940	An01g02250
evm.model.unitig_2.941	An01g02240
evm.model.unitig_2.942	An01g02230
evm.model.unitig_2.943	An01g02220
evm.model.unitig_2.944	An01g02210
evm.model.unitig_2.945	An01g02200
evm.model.unitig_2.946	An01g02190
evm.model.unitig_2.947	An01g02180
evm.model.unitig_2.948	An01g02170
evm.model.unitig_2.949	An01g02160
evm.model.unitig_2.95	An18g01460
evm.model.unitig_2.950	An01g02150
evm.model.unitig_2.951	An01g02140
evm.model.unitig_2.952	An01g02140
evm.model.unitig_2.953	An02g06470
evm.model.unitig_2.954	An01g02120
evm.model.unitig_2.955	An01g02110
evm.model.unitig_2.956	An01g02100
evm.model.unitig_2.957	An01g02090
evm.model.unitig_2.958	An01g02080
evm.model.unitig_2.959	An01g02070
evm.model.unitig_2.96	An01g14000
evm.model.unitig_2.960	An01g02060
evm.model.unitig_2.961	An01g02050
evm.model.unitig_2.964	An09g01290
evm.model.unitig_2.965	An09g05340
evm.model.unitig_2.967	An01g02000

Gene ID of H915-1	Gene ID of 513.88
evm.model.unitig_2.968	An01g01980
evm.model.unitig_2.970	An01g01960
evm.model.unitig_2.971	An01g01950
evm.model.unitig_2.972	An01g01940
evm.model.unitig_2.974	An01g01920
evm.model.unitig_2.975	An01g01900
evm.model.unitig_2.976	An01g01880
evm.model.unitig_2.977	An01g01870
evm.model.unitig_2.978	-
evm.model.unitig_2.979	An01g01850
evm.model.unitig_2.98	An01g13960
evm.model.unitig_2.980	An01g01840
evm.model.unitig_2.981	An01g01830
evm.model.unitig_2.982	An01g01820
evm.model.unitig_2.983	An01g01810
evm.model.unitig_2.984	An01g01800
evm.model.unitig_2.985	An01g01790
evm.model.unitig_2.986	An01g01780
evm.model.unitig_2.987	An01g01750
evm.model.unitig_2.988	An01g01720
evm.model.unitig_2.989	An01g01670
evm.model.unitig_2.99	An01g13950
evm.model.unitig_2.990	An01g01640
evm.model.unitig_2.991	An01g01620
evm.model.unitig_2.992	An01g01600
evm.model.unitig_2.993	An01g01580
evm.model.unitig_2.994	An01g01560
evm.model.unitig_2.995	An01g01550
evm.model.unitig_2.996	An01g01540
evm.model.unitig_2.997	An01g01530
evm.model.unitig_2.998	An01g01520
evm.model.unitig_2.999	An01g01500
evm.model.unitig_3.1	An05g02540
evm.model.unitig_3.10	An05g02450
evm.model.unitig_3.100	An05g01160
evm.model.unitig_3.1000	An11g02440
evm.model.unitig_3.1001	An11g02430
evm.model.unitig_3.1002	An11g02400
evm.model.unitig_3.1003	An11g02390
evm.model.unitig_3.1004	An11g02380
evm.model.unitig_3.1005	-
evm.model.unitig_3.1006	-
evm.model.unitig_3.1007	-

Gene ID of H915-1	Gene ID of 513.88
evm.model.unitig_3.1008	-
evm.model.unitig_3.1009	An11g02370
evm.model.unitig_3.101	An05g01170
evm.model.unitig_3.1010	An11g02370
evm.model.unitig_3.1012	-
evm.model.unitig_3.1013	An11g02360
evm.model.unitig_3.1014	-
evm.model.unitig_3.1015	An11g02270
evm.model.unitig_3.1016	An11g02230
evm.model.unitig_3.1017	An11g02220
evm.model.unitig_3.1018	An11g02200
evm.model.unitig_3.1019	An11g02190
evm.model.unitig_3.102	An05g01200
evm.model.unitig_3.1020	An11g02180
evm.model.unitig_3.1021	An11g02170
evm.model.unitig_3.1022	An11g02160
evm.model.unitig_3.1023	An11g02150
evm.model.unitig_3.1024	An11g02140
evm.model.unitig_3.1025	An11g02120
evm.model.unitig_3.1026	An11g02110
evm.model.unitig_3.1027	An11g02100
evm.model.unitig_3.1028	-
evm.model.unitig_3.1029	An11g02090
evm.model.unitig_3.103	An05g01210
evm.model.unitig_3.1030	An11g02070
evm.model.unitig_3.1031	An11g02050
evm.model.unitig_3.1032	An11g02040
evm.model.unitig_3.1033	An11g02030
evm.model.unitig_3.1034	An11g02020
evm.model.unitig_3.1035	An11g02010
evm.model.unitig_3.1036	An11g02000
evm.model.unitig_3.1037	An11g01990
evm.model.unitig_3.1038	An11g01980
evm.model.unitig_3.1039	An11g01970
evm.model.unitig_3.104	An06g00680
evm.model.unitig_3.1040	An11g01960
evm.model.unitig_3.1041	An11g01950
evm.model.unitig_3.1042	An11g01940
evm.model.unitig_3.1043	An11g05660
evm.model.unitig_3.1044	An11g01890
evm.model.unitig_3.1045	An11g01880
evm.model.unitig_3.1046	An11g01870
evm.model.unitig_3.1047	An11g01860

Gene ID of H915-1	Gene ID of 513.88
evm.model.unitig_3.1048	An11g01850
evm.model.unitig_3.1049	An11g01840
evm.model.unitig_3.1050	An11g01830
evm.model.unitig_3.1051	An11g01810
evm.model.unitig_3.1052	An11g01790
evm.model.unitig_3.1053	An11g01770
evm.model.unitig_3.1054	An11g01760
evm.model.unitig_3.1055	An11g01750
evm.model.unitig_3.1056	An11g01740
evm.model.unitig_3.1057	An11g01720
evm.model.unitig_3.1058	An11g01700
evm.model.unitig_3.1059	An11g01660
evm.model.unitig_3.1060	An11g01650
evm.model.unitig_3.1061	An11g01640
evm.model.unitig_3.1062	An11g01630
evm.model.unitig_3.1063	An11g01610
evm.model.unitig_3.1064	An11g01600
evm.model.unitig_3.1065	An11g01580
evm.model.unitig_3.1066	An11g01570
evm.model.unitig_3.1067	An11g01550
evm.model.unitig_3.1068	An11g01540
evm.model.unitig_3.1069	An11g01520
evm.model.unitig_3.1070	An11g01510
evm.model.unitig_3.1071	An12g00830
evm.model.unitig_3.1072	An11g01430
evm.model.unitig_3.1073	An11g01420
evm.model.unitig_3.1074	An11g01410
evm.model.unitig_3.1075	An11g01400
evm.model.unitig_3.1076	An11g01390
evm.model.unitig_3.1077	An11g01380
evm.model.unitig_3.1078	An11g01350
evm.model.unitig_3.1079	An11g01340
evm.model.unitig_3.1080	An11g01330
evm.model.unitig_3.1081	An11g01310
evm.model.unitig_3.1082	An11g01300
evm.model.unitig_3.1083	An11g01270
evm.model.unitig_3.1084	An11g01260
evm.model.unitig_3.1085	An13g02410
evm.model.unitig_3.1086	An11g01250
evm.model.unitig_3.1087	An11g01240
evm.model.unitig_3.1088	An11g01220
evm.model.unitig_3.1089	An11g01200
evm.model.unitig_3.109	An05g01270

Gene ID of H915-1	Gene ID of 513.88
evm.model.unitig_3.1090	An11g01190
evm.model.unitig_3.1091	An11g01180
evm.model.unitig_3.1092	An08g09030
evm.model.unitig_3.1093	An11g01150
evm.model.unitig_3.1094	An11g01140
evm.model.unitig_3.1095	An11g01140
evm.model.unitig_3.1096	An11g01120
evm.model.unitig_3.1097	An11g01110
evm.model.unitig_3.1098	An11g01100
evm.model.unitig_3.1099	An11g01080
evm.model.unitig_3.11	An05g02440
evm.model.unitig_3.110	An05g01290
evm.model.unitig_3.1100	An11g01070
evm.model.unitig_3.1101	An11g01060
evm.model.unitig_3.1102	An11g01040
evm.model.unitig_3.1103	An11g01000
evm.model.unitig_3.1104	An11g00990
evm.model.unitig_3.1105	An11g00970
evm.model.unitig_3.1106	An11g00960
evm.model.unitig_3.1107	An15g05650
evm.model.unitig_3.1108	An11g00940
evm.model.unitig_3.1109	An11g00930
evm.model.unitig_3.111	An05g01320
evm.model.unitig_3.1110	An11g00920
evm.model.unitig_3.1111	An11g00910
evm.model.unitig_3.1112	An11g00890
evm.model.unitig_3.1113	An11g00870
evm.model.unitig_3.1114	An11g00860
evm.model.unitig_3.1115	-
evm.model.unitig_3.1116	An11g00840
evm.model.unitig_3.1117	An11g00830
evm.model.unitig_3.1118	An11g00810
evm.model.unitig_3.1119	An11g00800
evm.model.unitig_3.112	An05g01330
evm.model.unitig_3.1120	An11g00790
evm.model.unitig_3.1121	An11g00780
evm.model.unitig_3.1122	An11g00770
evm.model.unitig_3.1123	An11g00760
evm.model.unitig_3.1124	-
evm.model.unitig_3.1125	An11g00750
evm.model.unitig_3.1126	An11g00740
evm.model.unitig_3.1127	-
evm.model.unitig_3.1128	-

Gene ID of H915-1	Gene ID of 513.88
evm.model.unitig_3.1129	An11g00690
evm.model.unitig_3.113	An05g01340
evm.model.unitig_3.1130	An11g00680
evm.model.unitig_3.1131	An11g00670
evm.model.unitig_3.1132	An11g00660
evm.model.unitig_3.1133	An11g00650
evm.model.unitig_3.1134	An11g00640
evm.model.unitig_3.1135	An11g00630
evm.model.unitig_3.1136	An11g00620
evm.model.unitig_3.1137	An11g00610
evm.model.unitig_3.1138	An11g00600
evm.model.unitig_3.1139	An11g00590
evm.model.unitig_3.114	An05g01350
evm.model.unitig_3.1140	An11g00580
evm.model.unitig_3.1141	An11g00570
evm.model.unitig_3.1142	-
evm.model.unitig_3.1143	-
evm.model.unitig_3.1144	An11g00530
evm.model.unitig_3.1145	An11g00510
evm.model.unitig_3.1146	An11g00500
evm.model.unitig_3.1147	An11g00490
evm.model.unitig_3.1148	An11g00480
evm.model.unitig_3.1149	An11g00470
evm.model.unitig_3.115	An12g03460
evm.model.unitig_3.1150	An11g00460
evm.model.unitig_3.1151	An11g00450
evm.model.unitig_3.1152	An11g00440
evm.model.unitig_3.1153	An11g00430
evm.model.unitig_3.1154	An11g00420
evm.model.unitig_3.1155	An11g00410
evm.model.unitig_3.1156	An11g00400
evm.model.unitig_3.1157	An11g00390
evm.model.unitig_3.1158	An11g00380
evm.model.unitig_3.1159	An11g00370
evm.model.unitig_3.116	An12g03440
evm.model.unitig_3.1160	An11g00350
evm.model.unitig_3.1161	An11g00310
evm.model.unitig_3.1162	An11g00300
evm.model.unitig_3.1163	An11g00300
evm.model.unitig_3.1164	An11g00280
evm.model.unitig_3.1165	An11g00260
evm.model.unitig_3.1166	An11g00220
evm.model.unitig_3.1167	An11g00210

Gene ID of H915-1	Gene ID of 513.88
evm.model.unitig_3.1168	An11g00200
evm.model.unitig_3.1169	An12g05820
evm.model.unitig_3.1170	An11g00130
evm.model.unitig_3.1171	An11g00120
evm.model.unitig_3.1172	An11g00110
evm.model.unitig_3.1173	An11g00100
evm.model.unitig_3.1176	An11g00070
evm.model.unitig_3.1177	An11g00070
evm.model.unitig_3.1178	An11g00060
evm.model.unitig_3.1179	An11g00050
evm.model.unitig_3.1180	An11g00040
evm.model.unitig_3.1182	An04g02910
evm.model.unitig_3.1183	An04g02930
evm.model.unitig_3.1184	An12g01730
evm.model.unitig_3.1185	An09g01210
evm.model.unitig_3.1186	An01g07570
evm.model.unitig_3.1188	An14g03500
evm.model.unitig_3.119	An12g03370
evm.model.unitig_3.1190	An04g02850
evm.model.unitig_3.1191	An16g06580
evm.model.unitig_3.1192	An07g05640
evm.model.unitig_3.1193	An11g09490
evm.model.unitig_3.1195	An15g00840
evm.model.unitig_3.1196	An09g03770
evm.model.unitig_3.12	An05g02420
evm.model.unitig_3.121	An12g03310
evm.model.unitig_3.122	An12g03300
evm.model.unitig_3.123	An12g03290
evm.model.unitig_3.124	An12g03280
evm.model.unitig_3.125	An12g03270
evm.model.unitig_3.126	An12g03260
evm.model.unitig_3.127	An12g03250
evm.model.unitig_3.128	An12g03240
evm.model.unitig_3.129	An12g03230
evm.model.unitig_3.13	An05g02410
evm.model.unitig_3.130	An12g03220
evm.model.unitig_3.131	An12g03210
evm.model.unitig_3.132	-
evm.model.unitig_3.133	An12g03200
evm.model.unitig_3.134	An12g03190
evm.model.unitig_3.135	An12g03180
evm.model.unitig_3.136	An12g03170
evm.model.unitig_3.137	An12g03160

Gene ID of H915-1	Gene ID of 513.88
evm.model.unitig_3.138	An12g03150
evm.model.unitig_3.139	An12g03140
evm.model.unitig_3.14	An05g02400
evm.model.unitig_3.140	An12g03130
evm.model.unitig_3.141	An12g03120
evm.model.unitig_3.142	An12g03110
evm.model.unitig_3.143	An07g01680
evm.model.unitig_3.144	An12g03070
evm.model.unitig_3.145	An12g03040
evm.model.unitig_3.146	An12g02990
evm.model.unitig_3.147	An12g02970
evm.model.unitig_3.148	An12g02960
evm.model.unitig_3.149	An12g02950
evm.model.unitig_3.15	An05g02390
evm.model.unitig_3.151	An12g02930
evm.model.unitig_3.152	An12g02920
evm.model.unitig_3.153	An12g02900
evm.model.unitig_3.154	An12g02880
evm.model.unitig_3.155	An12g02860
evm.model.unitig_3.156	An12g02850
evm.model.unitig_3.157	An12g02840
evm.model.unitig_3.158	An12g02830
evm.model.unitig_3.159	An12g02810
evm.model.unitig_3.16	An05g02380
evm.model.unitig_3.160	An12g02800
evm.model.unitig_3.161	An12g02790
evm.model.unitig_3.162	An12g02730
evm.model.unitig_3.163	An12g02720
evm.model.unitig_3.164	An12g02700
evm.model.unitig_3.165	An12g02680
evm.model.unitig_3.166	An12g02670
evm.model.unitig_3.167	An12g02670
evm.model.unitig_3.169	An12g02650
evm.model.unitig_3.17	An05g02370
evm.model.unitig_3.170	An12g02640
evm.model.unitig_3.171	An12g02630
evm.model.unitig_3.172	An11g09210
evm.model.unitig_3.173	An16g04840
evm.model.unitig_3.174	An12g02590
evm.model.unitig_3.175	An12g02520
evm.model.unitig_3.176	An12g02510
evm.model.unitig_3.177	An12g02500
evm.model.unitig_3.178	An12g02490

Gene ID of H915-1	Gene ID of 513.88
evm.model.unitig_3.179	An12g02480
evm.model.unitig_3.18	An05g02340
evm.model.unitig_3.180	An12g02470
evm.model.unitig_3.181	An12g02460
evm.model.unitig_3.182	An12g02450
evm.model.unitig_3.183	An12g02430
evm.model.unitig_3.184	An12g02420
evm.model.unitig_3.185	An12g02410
evm.model.unitig_3.186	An12g02400
evm.model.unitig_3.187	An09g04980
evm.model.unitig_3.188	An12g02380
evm.model.unitig_3.189	An12g02370
evm.model.unitig_3.19	An05g02330
evm.model.unitig_3.190	An12g02360
evm.model.unitig_3.191	An15g00810
evm.model.unitig_3.192	An12g02340
evm.model.unitig_3.193	An12g02330
evm.model.unitig_3.194	An12g02320
evm.model.unitig_3.195	An12g02300
evm.model.unitig_3.196	An12g02280
evm.model.unitig_3.197	An12g02240
evm.model.unitig_3.198	An12g02220
evm.model.unitig_3.199	An12g02210
evm.model.unitig_3.2	An01g00640
evm.model.unitig_3.20	An05g02320
evm.model.unitig_3.200	An12g02190
evm.model.unitig_3.201	An02g09120
evm.model.unitig_3.202	An15g03830
evm.model.unitig_3.203	An12g02120
evm.model.unitig_3.204	An12g02110
evm.model.unitig_3.205	An12g02100
evm.model.unitig_3.206	An12g02090
evm.model.unitig_3.207	An12g02080
evm.model.unitig_3.208	An12g02060
evm.model.unitig_3.209	An12g02050
evm.model.unitig_3.21	An05g02310
evm.model.unitig_3.210	An12g02040
evm.model.unitig_3.211	An12g02020
evm.model.unitig_3.212	An12g02020
evm.model.unitig_3.213	An12g01990
evm.model.unitig_3.214	An12g01980
evm.model.unitig_3.215	An02g09120
evm.model.unitig_3.216	An12g01960

Gene ID of H915-1	Gene ID of 513.88
evm.model.unitig_3.217	An12g01950
evm.model.unitig_3.218	An12g01940
evm.model.unitig_3.219	An12g01930
evm.model.unitig_3.22	An05g02280
evm.model.unitig_3.220	An12g01920
evm.model.unitig_3.221	An12g01910
evm.model.unitig_3.222	An12g01900
evm.model.unitig_3.223	An12g01890
evm.model.unitig_3.224	An12g01880
evm.model.unitig_3.225	An12g01870
evm.model.unitig_3.226	An12g01850
evm.model.unitig_3.227	An16g05190
evm.model.unitig_3.228	An12g01820
evm.model.unitig_3.229	An12g01800
evm.model.unitig_3.23	An05g02270
evm.model.unitig_3.230	An12g01750
evm.model.unitig_3.231	An12g01730
evm.model.unitig_3.232	An12g01710
evm.model.unitig_3.233	An12g01700
evm.model.unitig_3.234	An12g01680
evm.model.unitig_3.235	An12g01670
evm.model.unitig_3.236	An02g10560
evm.model.unitig_3.237	An07g05970
evm.model.unitig_3.24	An05g02260
evm.model.unitig_3.240	An12g01640
evm.model.unitig_3.241	An12g01630
evm.model.unitig_3.242	An12g01620
evm.model.unitig_3.243	An12g01610
evm.model.unitig_3.244	An12g01600
evm.model.unitig_3.245	An12g01590
evm.model.unitig_3.246	An12g01580
evm.model.unitig_3.247	An12g01570
evm.model.unitig_3.248	An12g01560
evm.model.unitig_3.249	An12g01550
evm.model.unitig_3.25	An05g02250
evm.model.unitig_3.250	An12g01540
evm.model.unitig_3.251	An12g01520
evm.model.unitig_3.252	An12g01510
evm.model.unitig_3.253	An12g01500
evm.model.unitig_3.254	An12g01490
evm.model.unitig_3.255	An12g01480
evm.model.unitig_3.256	An12g01470
evm.model.unitig_3.257	An12g01460

Gene ID of H915-1	Gene ID of 513.88
evm.model.unitig_3.259	An12g01380
evm.model.unitig_3.26	An11g08510
evm.model.unitig_3.260	-
evm.model.unitig_3.261	An12g01370
evm.model.unitig_3.262	An12g01360
evm.model.unitig_3.263	An12g01350
evm.model.unitig_3.264	An12g01340
evm.model.unitig_3.265	An12g01330
evm.model.unitig_3.266	An12g01320
evm.model.unitig_3.267	An12g01310
evm.model.unitig_3.268	An18g03620
evm.model.unitig_3.269	An01g02240
evm.model.unitig_3.27	An05g02230
evm.model.unitig_3.270	An12g01280
evm.model.unitig_3.271	An12g01280
evm.model.unitig_3.272	An12g01260
evm.model.unitig_3.273	An12g01250
evm.model.unitig_3.274	An12g01210
evm.model.unitig_3.275	An12g01190
evm.model.unitig_3.276	An12g01180
evm.model.unitig_3.277	An12g01170
evm.model.unitig_3.278	An12g01150
evm.model.unitig_3.279	An12g01140
evm.model.unitig_3.28	An05g02220
evm.model.unitig_3.280	An12g01130
evm.model.unitig_3.281	An12g01110
evm.model.unitig_3.282	An12g01100
evm.model.unitig_3.283	An12g01080
evm.model.unitig_3.284	An12g01070
evm.model.unitig_3.285	An12g01060
evm.model.unitig_3.286	An14g07190
evm.model.unitig_3.287	An12g01040
evm.model.unitig_3.288	An12g01030
evm.model.unitig_3.289	An12g01020
evm.model.unitig_3.29	An05g02210
evm.model.unitig_3.290	An12g01010
evm.model.unitig_3.291	An12g01000
evm.model.unitig_3.292	An12g00980
evm.model.unitig_3.293	An12g00960
evm.model.unitig_3.294	An12g00950
evm.model.unitig_3.295	An12g00940
evm.model.unitig_3.296	An12g00930
evm.model.unitig_3.297	An12g00920

Gene ID of H915-1	Gene ID of 513.88
evm.model.unitig_3.298	An12g00910
evm.model.unitig_3.299	An12g00900
evm.model.unitig_3.30	An05g02200
evm.model.unitig_3.300	An12g00890
evm.model.unitig_3.301	An12g00880
evm.model.unitig_3.302	An12g00870
evm.model.unitig_3.304	An12g00840
evm.model.unitig_3.305	An12g00830
evm.model.unitig_3.306	An12g00800
evm.model.unitig_3.307	An12g00790
evm.model.unitig_3.308	An12g00780
evm.model.unitig_3.309	An12g00770
evm.model.unitig_3.31	An05g02190
evm.model.unitig_3.310	An12g00760
evm.model.unitig_3.311	An12g00750
evm.model.unitig_3.312	An12g00730
evm.model.unitig_3.313	An12g00720
evm.model.unitig_3.314	An12g00710
evm.model.unitig_3.315	An12g00700
evm.model.unitig_3.316	An12g00690
evm.model.unitig_3.317	-
evm.model.unitig_3.318	An12g00680
evm.model.unitig_3.319	An09g03270
evm.model.unitig_3.32	An05g02170
evm.model.unitig_3.320	An12g00670
evm.model.unitig_3.321	An12g00660
evm.model.unitig_3.322	An12g00640
evm.model.unitig_3.323	An12g00630
evm.model.unitig_3.324	An12g00620
evm.model.unitig_3.325	An12g00610
evm.model.unitig_3.326	An12g00600
evm.model.unitig_3.327	An12g00590
evm.model.unitig_3.328	An12g00580
evm.model.unitig_3.329	An12g00570
evm.model.unitig_3.33	An16g01190
evm.model.unitig_3.330	An12g00550
evm.model.unitig_3.331	An12g00530
evm.model.unitig_3.332	An12g00520
evm.model.unitig_3.333	An12g00510
evm.model.unitig_3.334	An12g00500
evm.model.unitig_3.335	An12g00490
evm.model.unitig_3.336	An12g00480
evm.model.unitig_3.337	An12g00470

Gene ID of H915-1	Gene ID of 513.88
evm.model.unitig_3.338	An12g00460
evm.model.unitig_3.339	An12g00450
evm.model.unitig_3.34	An03g06670
evm.model.unitig_3.340	An12g00440
evm.model.unitig_3.341	An12g00410
evm.model.unitig_3.342	An12g00400
evm.model.unitig_3.343	An12g00390
evm.model.unitig_3.344	An12g00380
evm.model.unitig_3.345	An12g00370
evm.model.unitig_3.346	An12g00350
evm.model.unitig_3.347	An12g00340
evm.model.unitig_3.348	An12g00330
evm.model.unitig_3.349	An12g00320
evm.model.unitig_3.35	An03g01670
evm.model.unitig_3.350	An12g00260
evm.model.unitig_3.351	An12g00240
evm.model.unitig_3.352	An12g00230
evm.model.unitig_3.353	An12g00220
evm.model.unitig_3.354	An12g00210
evm.model.unitig_3.355	An12g00180
evm.model.unitig_3.356	An12g00170
evm.model.unitig_3.357	An12g00160
evm.model.unitig_3.358	-
evm.model.unitig_3.359	An12g00130
evm.model.unitig_3.36	An05g02080
evm.model.unitig_3.360	An12g00120
evm.model.unitig_3.361	An12g00060
evm.model.unitig_3.362	An12g00050
evm.model.unitig_3.363	An12g00030
evm.model.unitig_3.364	An12g00020
evm.model.unitig_3.368	An18g03700
evm.model.unitig_3.37	An05g02070
evm.model.unitig_3.370	An11g11320
evm.model.unitig_3.371	An11g11310
evm.model.unitig_3.372	An11g11300
evm.model.unitig_3.373	An11g11280
evm.model.unitig_3.375	An11g11260
evm.model.unitig_3.376	An11g11250
evm.model.unitig_3.377	An11g11240
evm.model.unitig_3.378	An11g11230
evm.model.unitig_3.379	An11g11210
evm.model.unitig_3.38	An05g02060
evm.model.unitig_3.380	An11g11190

Gene ID of H915-1	Gene ID of 513.88
evm.model.unitig_3.381	An11g11180
evm.model.unitig_3.382	An11g11170
evm.model.unitig_3.383	An11g11160
evm.model.unitig_3.384	An11g11150
evm.model.unitig_3.385	An11g11140
evm.model.unitig_3.386	An11g11130
evm.model.unitig_3.387	An11g11120
evm.model.unitig_3.388	An11g11110
evm.model.unitig_3.389	An11g11100
evm.model.unitig_3.39	An05g02050
evm.model.unitig_3.390	An11g11090
evm.model.unitig_3.391	An11g11080
evm.model.unitig_3.392	An11g11070
evm.model.unitig_3.393	An11g11070
evm.model.unitig_3.394	An11g11050
evm.model.unitig_3.395	An11g11040
evm.model.unitig_3.396	An11g11020
evm.model.unitig_3.397	An11g11010
evm.model.unitig_3.398	An11g11000
evm.model.unitig_3.399	An11g10990
evm.model.unitig_3.4	An05g02510
evm.model.unitig_3.40	An05g02030
evm.model.unitig_3.400	An11g10970
evm.model.unitig_3.401	An11g10960
evm.model.unitig_3.402	An11g10950
evm.model.unitig_3.403	An11g10930
evm.model.unitig_3.404	An11g10920
evm.model.unitig_3.405	An11g10910
evm.model.unitig_3.406	An11g10890
evm.model.unitig_3.407	An11g10870
evm.model.unitig_3.408	An11g10840
evm.model.unitig_3.409	An11g10820
evm.model.unitig_3.41	An05g02010
evm.model.unitig_3.410	An11g10810
evm.model.unitig_3.411	An11g10800
evm.model.unitig_3.412	An11g10790
evm.model.unitig_3.413	An11g10780
evm.model.unitig_3.414	An11g10760
evm.model.unitig_3.415	An11g10750
evm.model.unitig_3.416	An11g10740
evm.model.unitig_3.417	An11g10730
evm.model.unitig_3.418	An11g10720
evm.model.unitig_3.419	An11g10710

Gene ID of H915-1	Gene ID of 513.88
evm.model.unitig_3.42	An05g02000
evm.model.unitig_3.420	An11g10690
evm.model.unitig_3.421	An11g10680
evm.model.unitig_3.422	An11g10670
evm.model.unitig_3.423	An11g10660
evm.model.unitig_3.424	An11g10650
evm.model.unitig_3.425	An11g10630
evm.model.unitig_3.426	An11g10620
evm.model.unitig_3.427	An11g10610
evm.model.unitig_3.428	An11g10600
evm.model.unitig_3.429	An11g10580
evm.model.unitig_3.43	An05g01960
evm.model.unitig_3.430	An11g10560
evm.model.unitig_3.431	An11g10540
evm.model.unitig_3.432	An11g10520
evm.model.unitig_3.433	An15g05650
evm.model.unitig_3.434	An11g10490
evm.model.unitig_3.435	An11g10470
evm.model.unitig_3.436	An11g10460
evm.model.unitig_3.437	An11g10450
evm.model.unitig_3.438	An11g10430
evm.model.unitig_3.439	An11g10410
evm.model.unitig_3.440	An11g10400
evm.model.unitig_3.441	An11g10390
evm.model.unitig_3.442	An11g10380
evm.model.unitig_3.443	An11g10350
evm.model.unitig_3.444	An11g10340
evm.model.unitig_3.445	An11g10330
evm.model.unitig_3.446	An11g10320
evm.model.unitig_3.447	An11g10310
evm.model.unitig_3.448	An11g10300
evm.model.unitig_3.449	An11g10290
evm.model.unitig_3.450	An08g04870
evm.model.unitig_3.451	An11g09240
evm.model.unitig_3.452	An11g10260
evm.model.unitig_3.453	An11g00640
evm.model.unitig_3.454	An11g00640
evm.model.unitig_3.455	An11g10240
evm.model.unitig_3.456	An11g10210
evm.model.unitig_3.457	An11g10190
evm.model.unitig_3.458	An11g10180
evm.model.unitig_3.459	An11g10160
evm.model.unitig_3.46	An05g01890

Gene ID of H915-1	Gene ID of 513.88
evm.model.unitig_3.460	An11g10150
evm.model.unitig_3.461	An11g10140
evm.model.unitig_3.462	An11g10110
evm.model.unitig_3.463	An11g10100
evm.model.unitig_3.464	An11g10090
evm.model.unitig_3.465	An11g10080
evm.model.unitig_3.466	An11g10070
evm.model.unitig_3.467	An11g10060
evm.model.unitig_3.468	An11g10050
evm.model.unitig_3.469	An11g10030
evm.model.unitig_3.47	An05g01870
evm.model.unitig_3.470	An11g10020
evm.model.unitig_3.471	An11g10000
evm.model.unitig_3.472	An11g09990
evm.model.unitig_3.473	An11g09985
evm.model.unitig_3.474	An11g09980
evm.model.unitig_3.475	An11g09970
evm.model.unitig_3.476	An11g09960
evm.model.unitig_3.477	An11g09950
evm.model.unitig_3.478	An11g09940
evm.model.unitig_3.479	An11g09930
evm.model.unitig_3.48	An05g01860
evm.model.unitig_3.480	An11g09920
evm.model.unitig_3.481	An11g09910
evm.model.unitig_3.482	An11g09900
evm.model.unitig_3.483	An11g09890
evm.model.unitig_3.484	An11g09880
evm.model.unitig_3.485	An11g09870
evm.model.unitig_3.486	An11g09860
evm.model.unitig_3.487	An11g09850
evm.model.unitig_3.488	An11g09840
evm.model.unitig_3.489	An11g09830
evm.model.unitig_3.49	An05g01840
evm.model.unitig_3.490	An11g09820
evm.model.unitig_3.491	An11g09800
evm.model.unitig_3.492	An11g09790
evm.model.unitig_3.493	An11g09790
evm.model.unitig_3.494	An11g09780
evm.model.unitig_3.495	An11g09770
evm.model.unitig_3.496	An11g09760
evm.model.unitig_3.497	An11g09750
evm.model.unitig_3.498	An11g09740
evm.model.unitig_3.499	An11g09730

Gene ID of H915-1	Gene ID of 513.88
evm.model.unitig_3.5	An05g02500
evm.model.unitig_3.50	An05g01830
evm.model.unitig_3.500	-
evm.model.unitig_3.501	-
evm.model.unitig_3.502	An04g04340
evm.model.unitig_3.503	An11g09710
evm.model.unitig_3.504	An11g09700
evm.model.unitig_3.505	An11g09690
evm.model.unitig_3.506	An11g09680
evm.model.unitig_3.507	An11g09670
evm.model.unitig_3.508	An11g09660
evm.model.unitig_3.509	An11g09650
evm.model.unitig_3.51	An05g01820
evm.model.unitig_3.511	An11g09640
evm.model.unitig_3.512	An11g09630
evm.model.unitig_3.513	An11g09620
evm.model.unitig_3.514	An11g09610
evm.model.unitig_3.516	An11g09600
evm.model.unitig_3.517	An11g09590
evm.model.unitig_3.518	An11g09580
evm.model.unitig_3.519	An11g09570
evm.model.unitig_3.52	An05g01810
evm.model.unitig_3.520	An11g09560
evm.model.unitig_3.521	An11g09550
evm.model.unitig_3.522	An11g09540
evm.model.unitig_3.523	An11g09520
evm.model.unitig_3.524	An11g09510
evm.model.unitig_3.525	An11g09500
evm.model.unitig_3.526	An11g09490
evm.model.unitig_3.527	An11g09480
evm.model.unitig_3.528	An11g09470
evm.model.unitig_3.529	An11g09460
evm.model.unitig_3.53	An05g01800
evm.model.unitig_3.530	An11g09450
evm.model.unitig_3.531	An11g09440
evm.model.unitig_3.533	An11g09420
evm.model.unitig_3.534	An11g09390
evm.model.unitig_3.535	An11g09380
evm.model.unitig_3.536	An11g09370
evm.model.unitig_3.537	An11g09360
evm.model.unitig_3.538	An11g09350
evm.model.unitig_3.539	An11g09340
evm.model.unitig_3.54	An05g01780

Gene ID of H915-1	Gene ID of 513.88
evm.model.unitig_3.540	An11g09330
evm.model.unitig_3.541	An11g09320
evm.model.unitig_3.542	An11g09310
evm.model.unitig_3.546	-
evm.model.unitig_3.547	An11g00810
evm.model.unitig_3.548	An02g07580
evm.model.unitig_3.549	An02g03070
evm.model.unitig_3.55	An05g01770
evm.model.unitig_3.550	An01g03100
evm.model.unitig_3.551	-
evm.model.unitig_3.552	An11g09270
evm.model.unitig_3.553	An11g09260
evm.model.unitig_3.554	An11g09240
evm.model.unitig_3.555	An11g09230
evm.model.unitig_3.556	An11g09210
evm.model.unitig_3.557	An11g09200
evm.model.unitig_3.558	An11g09190
evm.model.unitig_3.559	An06g01420
evm.model.unitig_3.56	An05g01760
evm.model.unitig_3.560	An11g09170
evm.model.unitig_3.562	An11g09150
evm.model.unitig_3.563	An11g09140
evm.model.unitig_3.564	An11g09130
evm.model.unitig_3.565	An11g09120
evm.model.unitig_3.566	An11g09110
evm.model.unitig_3.567	An11g09010
evm.model.unitig_3.568	An11g09000
evm.model.unitig_3.569	An11g08990
evm.model.unitig_3.57	An05g01750
evm.model.unitig_3.570	An11g08950
evm.model.unitig_3.571	An11g08940
evm.model.unitig_3.572	An11g08930
evm.model.unitig_3.573	An11g08920
evm.model.unitig_3.575	An11g08900
evm.model.unitig_3.576	An11g08890
evm.model.unitig_3.577	An11g08880
evm.model.unitig_3.578	An11g08870
evm.model.unitig_3.579	An11g08860
evm.model.unitig_3.58	An05g01740
evm.model.unitig_3.580	An11g08840
evm.model.unitig_3.581	An11g08830
evm.model.unitig_3.582	An11g08820
evm.model.unitig_3.583	An11g08810

Gene ID of H915-1	Gene ID of 513.88
evm.model.unitig_3.584	An11g08790
evm.model.unitig_3.585	An11g08770
evm.model.unitig_3.586	An11g08750
evm.model.unitig_3.587	An11g06320
evm.model.unitig_3.588	An12g00950
evm.model.unitig_3.589	An11g08650
evm.model.unitig_3.59	An05g01720
evm.model.unitig_3.590	An09g01180
evm.model.unitig_3.591	An11g08620
evm.model.unitig_3.592	An11g08610
evm.model.unitig_3.593	An11g08550
evm.model.unitig_3.594	-
evm.model.unitig_3.595	An11g08530
evm.model.unitig_3.596	An11g08510
evm.model.unitig_3.597	An11g08510
evm.model.unitig_3.598	An11g08470
evm.model.unitig_3.599	An11g08470
evm.model.unitig_3.6	An05g02490
evm.model.unitig_3.60	An05g01710
evm.model.unitig_3.600	An11g08460
evm.model.unitig_3.601	An11g08440
evm.model.unitig_3.603	An11g08410
evm.model.unitig_3.604	An11g08400
evm.model.unitig_3.606	An11g08370
evm.model.unitig_3.609	An11g08320
evm.model.unitig_3.61	An05g01700
evm.model.unitig_3.610	An11g08280
evm.model.unitig_3.611	An11g08260
evm.model.unitig_3.612	An11g08250
evm.model.unitig_3.614	An11g08200
evm.model.unitig_3.615	An11g08190
evm.model.unitig_3.616	An11g08160
evm.model.unitig_3.617	An11g08150
evm.model.unitig_3.618	An11g08140
evm.model.unitig_3.619	An11g08130
evm.model.unitig_3.62	An05g01700
evm.model.unitig_3.620	An11g08100
evm.model.unitig_3.621	An11g08080
evm.model.unitig_3.622	An11g08070
evm.model.unitig_3.623	An11g08060
evm.model.unitig_3.624	An11g08030
evm.model.unitig_3.625	An11g08020
evm.model.unitig_3.626	An11g08010

Gene ID of H915-1	Gene ID of 513.88
evm.model.unitig_3.627	An11g07980
evm.model.unitig_3.628	An11g07960
evm.model.unitig_3.629	An11g07950
evm.model.unitig_3.63	An05g01700
evm.model.unitig_3.630	An11g07930
evm.model.unitig_3.631	An11g07910
evm.model.unitig_3.633	An11g07875
evm.model.unitig_3.634	An11g07840
evm.model.unitig_3.635	An11g07830
evm.model.unitig_3.636	An11g07820
evm.model.unitig_3.637	-
evm.model.unitig_3.638	An18g02610
evm.model.unitig_3.639	An14g02830
evm.model.unitig_3.64	-
evm.model.unitig_3.640	An11g07780
evm.model.unitig_3.641	An11g07760
evm.model.unitig_3.642	An11g07750
evm.model.unitig_3.643	An11g07740
evm.model.unitig_3.646	An11g07700
evm.model.unitig_3.647	An11g07690
evm.model.unitig_3.648	An11g07680
evm.model.unitig_3.649	-
evm.model.unitig_3.65	An05g01680
evm.model.unitig_3.650	An11g07670
evm.model.unitig_3.651	An11g07660
evm.model.unitig_3.652	An11g07650
evm.model.unitig_3.653	An11g07640
evm.model.unitig_3.654	An08g09040
evm.model.unitig_3.655	An11g07600
evm.model.unitig_3.656	An11g07590
evm.model.unitig_3.657	An11g07570
evm.model.unitig_3.658	An11g07550
evm.model.unitig_3.659	An09g00640
evm.model.unitig_3.660	An11g07510
evm.model.unitig_3.661	An11g07490
evm.model.unitig_3.662	An11g07450
evm.model.unitig_3.663	An11g07380
evm.model.unitig_3.664	An11g07350
evm.model.unitig_3.665	An11g07330
evm.model.unitig_3.666	An11g07320
evm.model.unitig_3.667	An11g07310
evm.model.unitig_3.668	An11g07300
evm.model.unitig_3.669	An11g07290

Gene ID of H915-1	Gene ID of 513.88
evm.model.unitig_3.67	An05g01660
evm.model.unitig_3.670	An11g07270
evm.model.unitig_3.671	-
evm.model.unitig_3.672	An11g07220
evm.model.unitig_3.673	An11g07210
evm.model.unitig_3.674	An11g07190
evm.model.unitig_3.675	An11g07160
evm.model.unitig_3.676	An11g07150
evm.model.unitig_3.677	An11g07120
evm.model.unitig_3.678	An11g07080
evm.model.unitig_3.679	An11g07070
evm.model.unitig_3.68	An05g01650
evm.model.unitig_3.680	An11g07060
evm.model.unitig_3.681	An11g07030
evm.model.unitig_3.682	An11g07020
evm.model.unitig_3.683	An11g07010
evm.model.unitig_3.684	An11g07000
evm.model.unitig_3.685	An11g06990
evm.model.unitig_3.686	An11g06980
evm.model.unitig_3.687	An11g06970
evm.model.unitig_3.688	An11g06960
evm.model.unitig_3.689	An11g06950
evm.model.unitig_3.69	An05g01640
evm.model.unitig_3.690	An11g06930
evm.model.unitig_3.691	An11g06920
evm.model.unitig_3.692	An11g06900
evm.model.unitig_3.693	An11g06890
evm.model.unitig_3.694	An11g06880
evm.model.unitig_3.695	An11g06860
evm.model.unitig_3.696	An11g06850
evm.model.unitig_3.697	An11g06830
evm.model.unitig_3.698	An11g06820
evm.model.unitig_3.699	An11g06810
evm.model.unitig_3.7	An05g02470
evm.model.unitig_3.70	-
evm.model.unitig_3.700	An11g06800
evm.model.unitig_3.701	An11g06790
evm.model.unitig_3.702	An11g06780
evm.model.unitig_3.703	An11g06770
evm.model.unitig_3.704	An11g06760
evm.model.unitig_3.705	An11g06750
evm.model.unitig_3.706	An11g06740
evm.model.unitig_3.707	An11g06730

Gene ID of H915-1	Gene ID of 513.88
evm.model.unitig_3.708	An11g06720
evm.model.unitig_3.709	An11g06690
evm.model.unitig_3.71	An05g01620
evm.model.unitig_3.710	An11g06680
evm.model.unitig_3.711	An11g06670
evm.model.unitig_3.712	An11g06660
evm.model.unitig_3.713	An11g06650
evm.model.unitig_3.714	An11g06640
evm.model.unitig_3.715	An11g06630
evm.model.unitig_3.716	An11g06610
evm.model.unitig_3.717	An11g06600
evm.model.unitig_3.718	An11g06590
evm.model.unitig_3.719	An11g06570
evm.model.unitig_3.72	An05g01610
evm.model.unitig_3.720	An11g06570
evm.model.unitig_3.721	An11g06540
evm.model.unitig_3.722	An11g06520
evm.model.unitig_3.723	An11g06510
evm.model.unitig_3.724	An11g06490
evm.model.unitig_3.725	An11g06460
evm.model.unitig_3.726	An11g06440
evm.model.unitig_3.727	An11g06430
evm.model.unitig_3.728	An11g06420
evm.model.unitig_3.729	An05g01870
evm.model.unitig_3.730	An11g06330
evm.model.unitig_3.731	An11g06320
evm.model.unitig_3.732	An11g06290
evm.model.unitig_3.733	An11g06280
evm.model.unitig_3.734	An11g06270
evm.model.unitig_3.735	An11g06260
evm.model.unitig_3.737	An11g06230
evm.model.unitig_3.738	-
evm.model.unitig_3.739	An11g06200
evm.model.unitig_3.74	An05g01560
evm.model.unitig_3.740	An11g06190
evm.model.unitig_3.741	An11g06180
evm.model.unitig_3.742	An11g06170
evm.model.unitig_3.743	An11g06160
evm.model.unitig_3.744	An11g06150
evm.model.unitig_3.745	An11g06140
evm.model.unitig_3.746	An11g06130
evm.model.unitig_3.747	An11g06120
evm.model.unitig_3.748	An11g06110

Gene ID of H915-1	Gene ID of 513.88
evm.model.unitig_3.749	An11g06080
evm.model.unitig_3.75	An05g01500
evm.model.unitig_3.750	An11g06060
evm.model.unitig_3.751	An11g06040
evm.model.unitig_3.752	An19g00080
evm.model.unitig_3.753	An11g06000
evm.model.unitig_3.755	An11g05960
evm.model.unitig_3.756	An11g05950
evm.model.unitig_3.757	An11g05940
evm.model.unitig_3.758	An11g05930
evm.model.unitig_3.759	An11g05910
evm.model.unitig_3.76	An05g01490
evm.model.unitig_3.761	An11g05850
evm.model.unitig_3.762	An11g05820
evm.model.unitig_3.763	An11g05810
evm.model.unitig_3.764	An11g05800
evm.model.unitig_3.766	An11g05780
evm.model.unitig_3.767	An11g05770
evm.model.unitig_3.768	An11g05750
evm.model.unitig_3.769	An11g05730
evm.model.unitig_3.77	An05g01480
evm.model.unitig_3.771	An11g05710
evm.model.unitig_3.772	An11g05700
evm.model.unitig_3.773	-
evm.model.unitig_3.774	An11g05680
evm.model.unitig_3.775	An11g05660
evm.model.unitig_3.776	An11g05640
evm.model.unitig_3.777	An11g05630
evm.model.unitig_3.778	An11g05600
evm.model.unitig_3.779	An11g05580
evm.model.unitig_3.78	An05g01450
evm.model.unitig_3.780	An11g05570
evm.model.unitig_3.781	An11g05560
evm.model.unitig_3.782	An11g05550
evm.model.unitig_3.783	-
evm.model.unitig_3.784	An11g05530
evm.model.unitig_3.785	An11g05520
evm.model.unitig_3.786	An11g05510
evm.model.unitig_3.787	An11g05500
evm.model.unitig_3.788	An11g05490
evm.model.unitig_3.789	An11g05480
evm.model.unitig_3.79	An05g01440
evm.model.unitig_3.790	An11g05470

Gene ID of H915-1	Gene ID of 513.88
evm.model.unitig_3.791	-
evm.model.unitig_3.792	An11g05460
evm.model.unitig_3.793	An11g05450
evm.model.unitig_3.794	An11g05440
evm.model.unitig_3.795	An11g05430
evm.model.unitig_3.796	An11g05430
evm.model.unitig_3.797	An11g05420
evm.model.unitig_3.798	-
evm.model.unitig_3.799	An11g05380
evm.model.unitig_3.8	An05g02470
evm.model.unitig_3.800	An11g05350
evm.model.unitig_3.801	An11g05340
evm.model.unitig_3.802	An11g05330
evm.model.unitig_3.803	An11g05320
evm.model.unitig_3.804	An11g05310
evm.model.unitig_3.805	An11g05280
evm.model.unitig_3.806	An11g05260
evm.model.unitig_3.807	An11g05240
evm.model.unitig_3.808	An11g05230
evm.model.unitig_3.809	An11g05190
evm.model.unitig_3.810	An11g05180
evm.model.unitig_3.811	An11g05130
evm.model.unitig_3.812	An11g05120
evm.model.unitig_3.813	An11g05110
evm.model.unitig_3.815	An11g05100
evm.model.unitig_3.816	An02g07610
evm.model.unitig_3.817	An11g05060
evm.model.unitig_3.818	An11g05010
evm.model.unitig_3.819	An11g05000
evm.model.unitig_3.82	An05g01410
evm.model.unitig_3.820	An11g04990
evm.model.unitig_3.821	An11g04970
evm.model.unitig_3.822	An11g04940
evm.model.unitig_3.823	An11g04930
evm.model.unitig_3.824	An11g04880
evm.model.unitig_3.825	An11g04880
evm.model.unitig_3.826	An11g04870
evm.model.unitig_3.827	An11g04860
evm.model.unitig_3.829	An11g04840
evm.model.unitig_3.83	An05g01400
evm.model.unitig_3.830	An11g04830
evm.model.unitig_3.831	An11g04820
evm.model.unitig_3.832	An11g04810

Gene ID of H915-1	Gene ID of 513.88
evm.model.unitig_3.833	An11g04780
evm.model.unitig_3.834	An11g04770
evm.model.unitig_3.835	An11g04750
evm.model.unitig_3.836	An11g04740
evm.model.unitig_3.837	An11g04730
evm.model.unitig_3.838	An11g04720
evm.model.unitig_3.839	An01g14020
evm.model.unitig_3.84	An05g01380
evm.model.unitig_3.840	An11g04700
evm.model.unitig_3.841	An11g04690
evm.model.unitig_3.842	An11g04670
evm.model.unitig_3.843	An11g04660
evm.model.unitig_3.844	An11g04650
evm.model.unitig_3.845	An11g04640
evm.model.unitig_3.846	An11g04630
evm.model.unitig_3.847	An11g04620
evm.model.unitig_3.848	An11g04610
evm.model.unitig_3.849	An11g04600
evm.model.unitig_3.85	An05g01370
evm.model.unitig_3.850	An11g04600
evm.model.unitig_3.851	An11g04550
evm.model.unitig_3.852	An11g04540
evm.model.unitig_3.853	An11g04530
evm.model.unitig_3.854	An11g04520
evm.model.unitig_3.855	An11g04510
evm.model.unitig_3.856	An11g04500
evm.model.unitig_3.857	An11g04480
evm.model.unitig_3.858	An11g04470
evm.model.unitig_3.859	An11g04460
evm.model.unitig_3.86	An05g00980
evm.model.unitig_3.860	An11g04420
evm.model.unitig_3.861	An11g04410
evm.model.unitig_3.862	An11g04400
evm.model.unitig_3.863	An11g04380
evm.model.unitig_3.864	An11g04370
evm.model.unitig_3.865	An11g04360
evm.model.unitig_3.866	An11g04340
evm.model.unitig_3.867	An11g04320
evm.model.unitig_3.868	An11g04310
evm.model.unitig_3.869	An11g04290
evm.model.unitig_3.87	An05g00990
evm.model.unitig_3.870	An11g04280
evm.model.unitig_3.871	An11g04270

Gene ID of H915-1	Gene ID of 513.88
evm.model.unitig_3.872	An11g04260
evm.model.unitig_3.873	An11g04250
evm.model.unitig_3.874	An11g04240
evm.model.unitig_3.875	An03g03030
evm.model.unitig_3.876	An11g04220
evm.model.unitig_3.877	An11g04210
evm.model.unitig_3.878	An11g04200
evm.model.unitig_3.879	An11g04180
evm.model.unitig_3.88	An05g01000
evm.model.unitig_3.880	An11g04150
evm.model.unitig_3.881	An11g04120
evm.model.unitig_3.882	An11g04110
evm.model.unitig_3.883	An11g04100
evm.model.unitig_3.884	An11g04090
evm.model.unitig_3.886	An11g04060
evm.model.unitig_3.887	An12g01700
evm.model.unitig_3.888	An11g04040
evm.model.unitig_3.889	An11g04030
evm.model.unitig_3.89	An05g01010
evm.model.unitig_3.890	An11g04020
evm.model.unitig_3.891	An11g04010
evm.model.unitig_3.892	An11g04000
evm.model.unitig_3.893	An11g03970
evm.model.unitig_3.894	An11g03960
evm.model.unitig_3.897	An08g08410
evm.model.unitig_3.898	An16g02400
evm.model.unitig_3.899	An11g03900
evm.model.unitig_3.9	An05g02460
evm.model.unitig_3.90	An05g01030
evm.model.unitig_3.900	An11g03890
evm.model.unitig_3.901	An11g03880
evm.model.unitig_3.902	An11g03870
evm.model.unitig_3.903	An11g03860
evm.model.unitig_3.904	An11g03830
evm.model.unitig_3.905	An11g03820
evm.model.unitig_3.906	An11g03810
evm.model.unitig_3.907	An11g03800
evm.model.unitig_3.908	An11g03790
evm.model.unitig_3.909	An11g03780
evm.model.unitig_3.91	An05g01040
evm.model.unitig_3.910	An11g03770
evm.model.unitig_3.911	An11g03760
evm.model.unitig_3.913	An11g03740

Gene ID of H915-1	Gene ID of 513.88
evm.model.unitig_3.914	An11g03730
evm.model.unitig_3.915	An11g03700
evm.model.unitig_3.916	An11g03690
evm.model.unitig_3.917	An11g03680
evm.model.unitig_3.918	An11g03660
evm.model.unitig_3.919	An11g03640
evm.model.unitig_3.92	An05g01060
evm.model.unitig_3.920	An11g03630
evm.model.unitig_3.921	An11g03620
evm.model.unitig_3.922	An11g03610
evm.model.unitig_3.923	An11g03600
evm.model.unitig_3.924	An11g03590
evm.model.unitig_3.925	An11g03580
evm.model.unitig_3.926	An11g03540
evm.model.unitig_3.927	An11g03530
evm.model.unitig_3.928	An11g03510
evm.model.unitig_3.929	An11g03500
evm.model.unitig_3.93	An05g01070
evm.model.unitig_3.930	An11g03480
evm.model.unitig_3.931	An11g03470
evm.model.unitig_3.932	An11g03460
evm.model.unitig_3.933	An11g03430
evm.model.unitig_3.934	An11g03420
evm.model.unitig_3.935	An11g03400
evm.model.unitig_3.936	An11g03390
evm.model.unitig_3.937	An11g03380
evm.model.unitig_3.938	An11g03350
evm.model.unitig_3.939	An11g03340
evm.model.unitig_3.94	An09g00380
evm.model.unitig_3.940	An11g03330
evm.model.unitig_3.941	An11g03310
evm.model.unitig_3.942	An11g03290
evm.model.unitig_3.943	An11g03270
evm.model.unitig_3.944	An11g03260
evm.model.unitig_3.945	An11g03240
evm.model.unitig_3.946	An11g03230
evm.model.unitig_3.947	An11g03220
evm.model.unitig_3.948	An11g03200
evm.model.unitig_3.949	An11g03190
evm.model.unitig_3.95	-
evm.model.unitig_3.950	An11g03180
evm.model.unitig_3.951	An11g03170
evm.model.unitig_3.952	An11g03160

Gene ID of H915-1	Gene ID of 513.88
evm.model.unitig_3.953	An11g03130
evm.model.unitig_3.954	An11g03120
evm.model.unitig_3.955	An11g03110
evm.model.unitig_3.956	An11g03090
evm.model.unitig_3.958	An11g03060
evm.model.unitig_3.959	An11g03150
evm.model.unitig_3.96	An05g01100
evm.model.unitig_3.960	An11g03040
evm.model.unitig_3.961	An11g03000
evm.model.unitig_3.962	An11g02990
evm.model.unitig_3.963	An11g02980
evm.model.unitig_3.964	An11g02970
evm.model.unitig_3.965	An11g02960
evm.model.unitig_3.966	An11g02950
evm.model.unitig_3.967	An11g02900
evm.model.unitig_3.968	An11g02870
evm.model.unitig_3.969	An11g02860
evm.model.unitig_3.97	An05g01110
evm.model.unitig_3.970	An11g02850
evm.model.unitig_3.971	An11g02840
evm.model.unitig_3.972	An11g02830
evm.model.unitig_3.973	An11g02780
evm.model.unitig_3.974	An11g02770
evm.model.unitig_3.975	An11g02760
evm.model.unitig_3.976	An11g02750
evm.model.unitig_3.977	An11g02730
evm.model.unitig_3.978	An11g02700
evm.model.unitig_3.979	An11g02690
evm.model.unitig_3.98	An05g01120
evm.model.unitig_3.980	An11g02670
evm.model.unitig_3.981	An11g02660
evm.model.unitig_3.982	An11g02650
evm.model.unitig_3.983	An11g02640
evm.model.unitig_3.984	An11g02630
evm.model.unitig_3.985	An11g02620
evm.model.unitig_3.986	An11g02610
evm.model.unitig_3.987	An11g02600
evm.model.unitig_3.988	An11g02590
evm.model.unitig_3.989	An11g02580
evm.model.unitig_3.99	An05g01140
evm.model.unitig_3.990	An11g02550
evm.model.unitig_3.991	An10g00660
evm.model.unitig_3.992	An11g02540

Gene ID of H915-1	Gene ID of 513.88
evm.model.unitig_3.993	An11g02520
evm.model.unitig_3.994	An11g02510
evm.model.unitig_3.995	An11g02500
evm.model.unitig_3.996	An11g02480
evm.model.unitig_3.997	An11g02470
evm.model.unitig_3.998	An11g02460
evm.model.unitig_3.999	An11g02450
evm.model.unitig_4.1	An03g06500
evm.model.unitig_4.10	An15g07910
evm.model.unitig_4.100	An15g06700
evm.model.unitig_4.1000	An12g08320
evm.model.unitig_4.1001	An12g08340
evm.model.unitig_4.1002	An12g08350
evm.model.unitig_4.1003	An12g08360
evm.model.unitig_4.1004	An12g08370
evm.model.unitig_4.1005	An12g08380
evm.model.unitig_4.1006	An12g08390
evm.model.unitig_4.1007	An12g08400
evm.model.unitig_4.1008	An12g08510
evm.model.unitig_4.1009	An12g08530
evm.model.unitig_4.101	An15g06690
evm.model.unitig_4.1010	An12g08550
evm.model.unitig_4.1011	An12g08560
evm.model.unitig_4.1012	An12g08570
evm.model.unitig_4.1013	An12g08580
evm.model.unitig_4.1014	An12g08590
evm.model.unitig_4.1015	An12g08610
evm.model.unitig_4.1016	An12g08620
evm.model.unitig_4.1017	An12g08630
evm.model.unitig_4.1018	An12g08640
evm.model.unitig_4.1019	An12g08650
evm.model.unitig_4.102	An15g06680
evm.model.unitig_4.1020	An12g08660
evm.model.unitig_4.1021	An12g08670
evm.model.unitig_4.1022	An12g08680
evm.model.unitig_4.1023	An12g08690
evm.model.unitig_4.1025	An12g08720
evm.model.unitig_4.1026	An12g08730
evm.model.unitig_4.1027	An12g08750
evm.model.unitig_4.1028	An12g08760
evm.model.unitig_4.1029	An12g08770
evm.model.unitig_4.103	An15g06670
evm.model.unitig_4.1030	An12g08790

Gene ID of H915-1	Gene ID of 513.88
evm.model.unitig_4.1031	An12g08800
evm.model.unitig_4.1032	An12g08820
evm.model.unitig_4.1033	An12g08830
evm.model.unitig_4.1034	An12g08890
evm.model.unitig_4.1035	An12g08900
evm.model.unitig_4.1036	An12g08930
evm.model.unitig_4.1037	An12g08940
evm.model.unitig_4.1038	An12g08950
evm.model.unitig_4.104	An15g06660
evm.model.unitig_4.1040	An12g08960
evm.model.unitig_4.1041	An12g09020
evm.model.unitig_4.1042	-
evm.model.unitig_4.1043	An12g09120
evm.model.unitig_4.1044	An12g09130
evm.model.unitig_4.1045	An12g09140
evm.model.unitig_4.1046	An12g06490
evm.model.unitig_4.1048	An12g09270
evm.model.unitig_4.1049	An12g09280
evm.model.unitig_4.105	An15g06650
evm.model.unitig_4.1050	An12g09310
evm.model.unitig_4.1051	An12g09330
evm.model.unitig_4.1052	An12g09340
evm.model.unitig_4.1053	An12g09350
evm.model.unitig_4.1054	An12g09370
evm.model.unitig_4.1056	An12g09440
evm.model.unitig_4.1057	-
evm.model.unitig_4.1058	An12g09460
evm.model.unitig_4.1059	An12g09470
evm.model.unitig_4.106	An15g06600
evm.model.unitig_4.1060	An12g09480
evm.model.unitig_4.1061	An12g09500
evm.model.unitig_4.1062	An12g09530
evm.model.unitig_4.1064	An12g09580
evm.model.unitig_4.1065	An12g09610
evm.model.unitig_4.1066	-
evm.model.unitig_4.1067	An12g09630
evm.model.unitig_4.1068	An12g09640
evm.model.unitig_4.1069	An12g09650
evm.model.unitig_4.107	An15g06580
evm.model.unitig_4.1070	An12g09660
evm.model.unitig_4.1071	An12g09670
evm.model.unitig_4.1072	An12g09680
evm.model.unitig_4.1073	An12g09700

Gene ID of H915-1	Gene ID of 513.88
evm.model.unitig_4.1074	An12g09720
evm.model.unitig_4.1075	An12g09750
evm.model.unitig_4.1076	An12g09760
evm.model.unitig_4.1077	An12g09780
evm.model.unitig_4.1078	An12g09790
evm.model.unitig_4.1079	An12g09810
evm.model.unitig_4.1080	An12g09820
evm.model.unitig_4.1081	An12g09830
evm.model.unitig_4.1082	An01g11010
evm.model.unitig_4.1083	An12g09860
evm.model.unitig_4.1084	An12g09880
evm.model.unitig_4.1085	An12g09900
evm.model.unitig_4.1086	An12g09910
evm.model.unitig_4.1089	An12g09940
evm.model.unitig_4.109	An15g06560
evm.model.unitig_4.1090	An12g09950
evm.model.unitig_4.1091	An12g09960
evm.model.unitig_4.1092	An12g09970
evm.model.unitig_4.1093	An12g09980
evm.model.unitig_4.1094	An12g09990
evm.model.unitig_4.1095	An12g10000
evm.model.unitig_4.1096	An12g10020
evm.model.unitig_4.1097	An12g10030
evm.model.unitig_4.1098	An12g10050
evm.model.unitig_4.1099	An12g10060
evm.model.unitig_4.11	An15g07900
evm.model.unitig_4.110	An15g06550
evm.model.unitig_4.1101	An12g10090
evm.model.unitig_4.1102	An12g10100
evm.model.unitig_4.1103	An12g10110
evm.model.unitig_4.1104	An12g10120
evm.model.unitig_4.1105	An12g10130
evm.model.unitig_4.1106	An12g10140
evm.model.unitig_4.1107	An12g10150
evm.model.unitig_4.1108	An12g10160
evm.model.unitig_4.1109	An12g10170
evm.model.unitig_4.111	An15g06540
evm.model.unitig_4.1110	An12g10180
evm.model.unitig_4.1111	An12g10190
evm.model.unitig_4.1112	-
evm.model.unitig_4.1113	An12g10220
evm.model.unitig_4.1114	An12g10230
evm.model.unitig_4.1115	An12g10240

Gene ID of H915-1	Gene ID of 513.88
evm.model.unitig_4.1116	An12g10250
evm.model.unitig_4.1117	An02g11030
evm.model.unitig_4.1118	An02g11030
evm.model.unitig_4.1119	An12g10280
evm.model.unitig_4.1120	An12g10290
evm.model.unitig_4.1121	An12g09260
evm.model.unitig_4.1122	An12g10320
evm.model.unitig_4.1123	An08g07910
evm.model.unitig_4.1124	An12g10330
evm.model.unitig_4.1125	An12g10350
evm.model.unitig_4.1126	An12g10360
evm.model.unitig_4.1127	An12g10380
evm.model.unitig_4.1128	An12g10390
evm.model.unitig_4.1129	An12g10400
evm.model.unitig_4.113	An15g06500
evm.model.unitig_4.1130	An12g10410
evm.model.unitig_4.1131	An12g10430
evm.model.unitig_4.1132	An12g10430
evm.model.unitig_4.1133	An12g10440
evm.model.unitig_4.1134	An12g10450
evm.model.unitig_4.1135	An12g10470
evm.model.unitig_4.1136	An12g10480
evm.model.unitig_4.1137	An12g10490
evm.model.unitig_4.114	An15g06490
evm.model.unitig_4.115	An15g06480
evm.model.unitig_4.116	An15g06470
evm.model.unitig_4.117	An15g06460
evm.model.unitig_4.118	An15g06450
evm.model.unitig_4.119	An15g06440
evm.model.unitig_4.12	An15g07880
evm.model.unitig_4.120	An15g06430
evm.model.unitig_4.121	An15g06420
evm.model.unitig_4.122	An15g06410
evm.model.unitig_4.123	An15g06400
evm.model.unitig_4.124	An15g06390
evm.model.unitig_4.125	An15g06380
evm.model.unitig_4.126	An15g06370
evm.model.unitig_4.127	An15g06360
evm.model.unitig_4.128	An15g06350
evm.model.unitig_4.130	An15g06320
evm.model.unitig_4.131	An15g06310
evm.model.unitig_4.132	An15g06290
evm.model.unitig_4.133	An15g06280

Gene ID of H915-1	Gene ID of 513.88
evm.model.unitig_4.134	An15g06270
evm.model.unitig_4.135	An15g06260
evm.model.unitig_4.136	An15g06250
evm.model.unitig_4.137	An15g06230
evm.model.unitig_4.138	An15g06220
evm.model.unitig_4.139	An15g06210
evm.model.unitig_4.140	An15g06200
evm.model.unitig_4.141	An15g06190
evm.model.unitig_4.142	An15g06180
evm.model.unitig_4.143	An15g06160
evm.model.unitig_4.144	An15g06150
evm.model.unitig_4.145	An15g06140
evm.model.unitig_4.146	An15g06120
evm.model.unitig_4.147	An15g06110
evm.model.unitig_4.148	An15g06080
evm.model.unitig_4.149	An15g06050
evm.model.unitig_4.15	An15g07840
evm.model.unitig_4.150	An15g06040
evm.model.unitig_4.151	An15g06030
evm.model.unitig_4.152	An15g06020
evm.model.unitig_4.153	An15g06010
evm.model.unitig_4.154	An15g05990
evm.model.unitig_4.155	An15g05980
evm.model.unitig_4.156	An15g05970
evm.model.unitig_4.157	An15g05960
evm.model.unitig_4.158	An15g05940
evm.model.unitig_4.159	An15g05920
evm.model.unitig_4.16	An15g07830
evm.model.unitig_4.160	An15g05910
evm.model.unitig_4.161	-
evm.model.unitig_4.162	An15g05890
evm.model.unitig_4.163	An15g05880
evm.model.unitig_4.165	An15g05820
evm.model.unitig_4.166	An15g05810
evm.model.unitig_4.167	An15g05790
evm.model.unitig_4.168	An15g05790
evm.model.unitig_4.169	An15g05770
evm.model.unitig_4.17	An15g07820
evm.model.unitig_4.170	An15g05750
evm.model.unitig_4.171	An15g05740
evm.model.unitig_4.172	An15g05730
evm.model.unitig_4.173	An15g05720
evm.model.unitig_4.174	An15g05710

Gene ID of H915-1	Gene ID of 513.88
evm.model.unitig_4.175	An15g05700
evm.model.unitig_4.176	An15g05690
evm.model.unitig_4.177	An15g05670
evm.model.unitig_4.178	An15g05650
evm.model.unitig_4.179	An15g05630
evm.model.unitig_4.18	An15g07820
evm.model.unitig_4.180	An15g05630
evm.model.unitig_4.182	An15g05580
evm.model.unitig_4.183	An15g05550
evm.model.unitig_4.184	An15g05540
evm.model.unitig_4.185	An15g05530
evm.model.unitig_4.186	An15g05520
evm.model.unitig_4.187	An15g05510
evm.model.unitig_4.188	An15g05500
evm.model.unitig_4.189	An15g05490
evm.model.unitig_4.19	An15g07810
evm.model.unitig_4.190	An15g05490
evm.model.unitig_4.191	An15g05490
evm.model.unitig_4.192	An15g05470
evm.model.unitig_4.193	An15g05460
evm.model.unitig_4.194	An15g05450
evm.model.unitig_4.195	An15g05440
evm.model.unitig_4.196	An15g05410
evm.model.unitig_4.197	An15g05400
evm.model.unitig_4.198	An15g05370
evm.model.unitig_4.199	An15g05360
evm.model.unitig_4.2	An16g07710
evm.model.unitig_4.20	An15g07800
evm.model.unitig_4.200	An15g05350
evm.model.unitig_4.201	An15g05320
evm.model.unitig_4.202	An15g05310
evm.model.unitig_4.203	An15g05290
evm.model.unitig_4.204	An15g05280
evm.model.unitig_4.205	An15g05280
evm.model.unitig_4.206	An15g05270
evm.model.unitig_4.207	An15g05230
evm.model.unitig_4.208	An15g05200
evm.model.unitig_4.209	An15g05180
evm.model.unitig_4.21	An15g07760
evm.model.unitig_4.210	An05g01850
evm.model.unitig_4.211	An15g05130
evm.model.unitig_4.212	An15g05120
evm.model.unitig_4.213	An15g05110

Gene ID of H915-1	Gene ID of 513.88
evm.model.unitig_4.214	An15g05090
evm.model.unitig_4.215	An15g05080
evm.model.unitig_4.216	An15g05070
evm.model.unitig_4.217	An15g05060
evm.model.unitig_4.218	An15g05030
evm.model.unitig_4.219	An15g05020
evm.model.unitig_4.22	An15g07740
evm.model.unitig_4.222	An15g04940
evm.model.unitig_4.223	An15g04930
evm.model.unitig_4.224	An15g04920
evm.model.unitig_4.225	An15g04900
evm.model.unitig_4.226	An15g04880
evm.model.unitig_4.228	An15g04860
evm.model.unitig_4.229	An15g04850
evm.model.unitig_4.23	An15g07730
evm.model.unitig_4.230	An15g04830
evm.model.unitig_4.231	An15g04820
evm.model.unitig_4.232	An15g04810
evm.model.unitig_4.233	An15g04800
evm.model.unitig_4.234	An15g04790
evm.model.unitig_4.235	An15g04780
evm.model.unitig_4.236	An15g04770
evm.model.unitig_4.237	An15g04760
evm.model.unitig_4.238	-
evm.model.unitig_4.239	An15g04750
evm.model.unitig_4.24	An15g07720
evm.model.unitig_4.240	An15g04740
evm.model.unitig_4.241	An15g04720
evm.model.unitig_4.242	-
evm.model.unitig_4.243	An15g04690
evm.model.unitig_4.244	An15g04680
evm.model.unitig_4.245	An15g04670
evm.model.unitig_4.246	An15g04660
evm.model.unitig_4.247	An15g04650
evm.model.unitig_4.248	An15g04640
evm.model.unitig_4.249	An15g04630
evm.model.unitig_4.25	An15g07710
evm.model.unitig_4.250	An15g04620
evm.model.unitig_4.251	An15g04610
evm.model.unitig_4.252	An15g04470
evm.model.unitig_4.253	An15g04480
evm.model.unitig_4.254	An15g04490
evm.model.unitig_4.256	An15g04490

Gene ID of H915-1	Gene ID of 513.88
evm.model.unitig_4.257	An15g04520
evm.model.unitig_4.258	An08g09140
evm.model.unitig_4.259	An15g04550
evm.model.unitig_4.26	An15g07700
evm.model.unitig_4.261	An15g04570
evm.model.unitig_4.262	An15g04580
evm.model.unitig_4.263	An15g04590
evm.model.unitig_4.264	An15g04600
evm.model.unitig_4.265	An15g04440
evm.model.unitig_4.266	An15g04430
evm.model.unitig_4.267	An15g04420
evm.model.unitig_4.268	An15g04410
evm.model.unitig_4.269	An15g04390
evm.model.unitig_4.27	An15g07670
evm.model.unitig_4.270	An15g04380
evm.model.unitig_4.271	An15g04370
evm.model.unitig_4.272	An15g04370
evm.model.unitig_4.273	An15g04360
evm.model.unitig_4.274	An15g04350
evm.model.unitig_4.275	An15g04340
evm.model.unitig_4.276	An15g04320
evm.model.unitig_4.277	An15g04310
evm.model.unitig_4.278	An15g04300
evm.model.unitig_4.279	An15g04290
evm.model.unitig_4.280	An15g04260
evm.model.unitig_4.281	An15g04220
evm.model.unitig_4.282	An02g01120
evm.model.unitig_4.283	An15g04170
evm.model.unitig_4.284	An15g04150
evm.model.unitig_4.285	An15g04140
evm.model.unitig_4.286	An15g04130
evm.model.unitig_4.287	An15g04120
evm.model.unitig_4.289	An15g04100
evm.model.unitig_4.29	An15g07600
evm.model.unitig_4.290	An15g04060
evm.model.unitig_4.291	An15g04050
evm.model.unitig_4.292	An15g04010
evm.model.unitig_4.293	An15g03990
evm.model.unitig_4.294	An15g03980
evm.model.unitig_4.296	An15g03950
evm.model.unitig_4.297	An15g03910
evm.model.unitig_4.298	An15g03900
evm.model.unitig_4.299	An15g03880

Gene ID of H915-1	Gene ID of 513.88
evm.model.unitig_4.3	An16g01190
evm.model.unitig_4.30	An15g07590
evm.model.unitig_4.300	An15g03860
evm.model.unitig_4.301	An15g03820
evm.model.unitig_4.302	An15g03810
evm.model.unitig_4.303	An15g03800
evm.model.unitig_4.304	An15g03780
evm.model.unitig_4.305	An15g03760
evm.model.unitig_4.306	An15g03750
evm.model.unitig_4.307	An15g03740
evm.model.unitig_4.308	An15g03730
evm.model.unitig_4.309	An15g03710
evm.model.unitig_4.31	An15g07580
evm.model.unitig_4.310	An15g03680
evm.model.unitig_4.311	An15g03670
evm.model.unitig_4.312	An15g03650
evm.model.unitig_4.313	An15g03580
evm.model.unitig_4.314	An15g03570
evm.model.unitig_4.315	An15g03550
evm.model.unitig_4.316	An15g03540
evm.model.unitig_4.317	An15g03530
evm.model.unitig_4.318	An15g03500
evm.model.unitig_4.319	An15g03490
evm.model.unitig_4.32	An15g07570
evm.model.unitig_4.320	-
evm.model.unitig_4.321	An15g03420
evm.model.unitig_4.322	An15g03410
evm.model.unitig_4.323	An15g03400
evm.model.unitig_4.324	An15g03390
evm.model.unitig_4.325	An15g03380
evm.model.unitig_4.326	An15g03370
evm.model.unitig_4.327	An15g03350
evm.model.unitig_4.328	An15g03340
evm.model.unitig_4.329	An15g03330
evm.model.unitig_4.33	An15g07560
evm.model.unitig_4.330	An15g03300
evm.model.unitig_4.331	An15g03280
evm.model.unitig_4.332	An15g03240
evm.model.unitig_4.333	An15g03220
evm.model.unitig_4.334	An15g03210
evm.model.unitig_4.335	An15g03200
evm.model.unitig_4.336	An15g03190
evm.model.unitig_4.337	An15g03190

Gene ID of H915-1	Gene ID of 513.88
evm.model.unitig_4.338	An15g03150
evm.model.unitig_4.339	An15g03140
evm.model.unitig_4.34	An15g07550
evm.model.unitig_4.340	An15g03100
evm.model.unitig_4.341	An15g03090
evm.model.unitig_4.342	An15g03080
evm.model.unitig_4.343	An15g03040
evm.model.unitig_4.344	An15g03030
evm.model.unitig_4.345	An15g03020
evm.model.unitig_4.346	An15g02990
evm.model.unitig_4.347	An15g02980
evm.model.unitig_4.348	An15g02960
evm.model.unitig_4.349	An15g02930
evm.model.unitig_4.35	An15g07530
evm.model.unitig_4.350	An15g02900
evm.model.unitig_4.351	An15g02890
evm.model.unitig_4.352	An15g02880
evm.model.unitig_4.354	An15g02860
evm.model.unitig_4.355	An15g02850
evm.model.unitig_4.356	An15g02840
evm.model.unitig_4.357	An15g02830
evm.model.unitig_4.358	An15g02820
evm.model.unitig_4.359	An15g02810
evm.model.unitig_4.36	An15g07520
evm.model.unitig_4.360	An15g02770
evm.model.unitig_4.361	An15g02760
evm.model.unitig_4.362	An15g02750
evm.model.unitig_4.363	An15g02740
evm.model.unitig_4.364	An15g02710
evm.model.unitig_4.365	An15g02700
evm.model.unitig_4.366	An15g02690
evm.model.unitig_4.367	An15g02670
evm.model.unitig_4.368	An15g02670
evm.model.unitig_4.369	An15g02650
evm.model.unitig_4.37	An15g07510
evm.model.unitig_4.370	An15g02640
evm.model.unitig_4.371	An15g02630
evm.model.unitig_4.372	An15g02620
evm.model.unitig_4.373	An15g02610
evm.model.unitig_4.374	An15g02600
evm.model.unitig_4.375	An15g02590
evm.model.unitig_4.377	An15g02550
evm.model.unitig_4.378	An18g01500

Gene ID of H915-1	Gene ID of 513.88
evm.model.unitig_4.379	An15g02520
evm.model.unitig_4.38	An15g07500
evm.model.unitig_4.381	An15g02490
evm.model.unitig_4.382	An15g02470
evm.model.unitig_4.383	An15g02460
evm.model.unitig_4.384	An15g02450
evm.model.unitig_4.385	An15g02440
evm.model.unitig_4.386	An15g02420
evm.model.unitig_4.387	An15g02410
evm.model.unitig_4.388	An15g02400
evm.model.unitig_4.389	An15g02390
evm.model.unitig_4.39	An15g07490
evm.model.unitig_4.390	An15g02380
evm.model.unitig_4.391	An15g02370
evm.model.unitig_4.392	An15g02360
evm.model.unitig_4.393	An15g02350
evm.model.unitig_4.394	An15g02340
evm.model.unitig_4.395	An15g02320
evm.model.unitig_4.396	An15g02300
evm.model.unitig_4.397	An15g02290
evm.model.unitig_4.398	An15g02280
evm.model.unitig_4.40	An15g07480
evm.model.unitig_4.401	An15g02210
evm.model.unitig_4.402	An15g02200
evm.model.unitig_4.403	An15g02190
evm.model.unitig_4.404	An15g02180
evm.model.unitig_4.405	An15g02150
evm.model.unitig_4.406	An15g02140
evm.model.unitig_4.407	An15g02130
evm.model.unitig_4.408	An15g02120
evm.model.unitig_4.409	An15g02110
evm.model.unitig_4.41	An15g07470
evm.model.unitig_4.410	An15g02090
evm.model.unitig_4.411	An15g02080
evm.model.unitig_4.412	An15g02070
evm.model.unitig_4.414	An15g02000
evm.model.unitig_4.415	An15g01990
evm.model.unitig_4.416	An15g01980
evm.model.unitig_4.417	An15g01970
evm.model.unitig_4.418	An15g01960
evm.model.unitig_4.419	An15g01950
evm.model.unitig_4.42	An15g07460
evm.model.unitig_4.420	An15g01940

Gene ID of H915-1	Gene ID of 513.88
evm.model.unitig_4.421	An15g01930
evm.model.unitig_4.422	An15g01920
evm.model.unitig_4.423	An15g01910
evm.model.unitig_4.424	An15g01900
evm.model.unitig_4.425	An15g01890
evm.model.unitig_4.426	An15g01870
evm.model.unitig_4.427	An15g01860
evm.model.unitig_4.428	An15g01850
evm.model.unitig_4.429	An15g01840
evm.model.unitig_4.43	An15g07390
evm.model.unitig_4.430	An15g01830
evm.model.unitig_4.431	An15g01810
evm.model.unitig_4.432	An15g01800
evm.model.unitig_4.433	An15g01790
evm.model.unitig_4.434	An15g01780
evm.model.unitig_4.435	An15g01770
evm.model.unitig_4.436	An15g01760
evm.model.unitig_4.437	An15g01750
evm.model.unitig_4.438	An15g01740
evm.model.unitig_4.439	An15g01720
evm.model.unitig_4.44	An15g07370
evm.model.unitig_4.440	An15g01710
evm.model.unitig_4.441	An15g01700
evm.model.unitig_4.442	An15g01690
evm.model.unitig_4.443	An15g01680
evm.model.unitig_4.444	An15g01670
evm.model.unitig_4.445	An15g01650
evm.model.unitig_4.446	An15g01640
evm.model.unitig_4.447	An15g01620
evm.model.unitig_4.448	An15g01600
evm.model.unitig_4.449	An15g01590
evm.model.unitig_4.45	An15g07360
evm.model.unitig_4.450	An15g01580
evm.model.unitig_4.451	An15g01560
evm.model.unitig_4.452	An15g01550
evm.model.unitig_4.453	An15g01530
evm.model.unitig_4.454	An15g01520
evm.model.unitig_4.455	An15g01510
evm.model.unitig_4.456	An15g01500
evm.model.unitig_4.457	An15g01490
evm.model.unitig_4.458	An15g01480
evm.model.unitig_4.459	An15g01470
evm.model.unitig_4.46	An15g07350

Gene ID of H915-1	Gene ID of 513.88
evm.model.unitig_4.460	An15g01460
evm.model.unitig_4.461	An15g01450
evm.model.unitig_4.462	An15g01440
evm.model.unitig_4.463	An15g01430
evm.model.unitig_4.464	An15g01420
evm.model.unitig_4.465	An15g01410
evm.model.unitig_4.466	An15g01390
evm.model.unitig_4.467	An15g01380
evm.model.unitig_4.468	An15g01370
evm.model.unitig_4.469	An15g01360
evm.model.unitig_4.470	An15g01350
evm.model.unitig_4.471	An15g01340
evm.model.unitig_4.472	An15g01330
evm.model.unitig_4.473	An15g01320
evm.model.unitig_4.474	An15g01310
evm.model.unitig_4.475	An15g01300
evm.model.unitig_4.476	An15g01290
evm.model.unitig_4.477	An15g01280
evm.model.unitig_4.478	An15g01270
evm.model.unitig_4.479	-
evm.model.unitig_4.48	An15g07330
evm.model.unitig_4.480	An15g01240
evm.model.unitig_4.481	An15g01230
evm.model.unitig_4.482	An04g04820
evm.model.unitig_4.483	An15g01200
evm.model.unitig_4.484	An15g01180
evm.model.unitig_4.485	An15g01170
evm.model.unitig_4.486	An15g01160
evm.model.unitig_4.487	An15g01155
evm.model.unitig_4.488	An15g01150
evm.model.unitig_4.489	An15g01140
evm.model.unitig_4.49	An12g01680
evm.model.unitig_4.490	An15g01130
evm.model.unitig_4.491	An15g01120
evm.model.unitig_4.492	An15g01110
evm.model.unitig_4.493	An02g14500
evm.model.unitig_4.494	An15g01020
evm.model.unitig_4.495	An15g01000
evm.model.unitig_4.496	An15g00990
evm.model.unitig_4.497	An15g00980
evm.model.unitig_4.498	An15g00970
evm.model.unitig_4.499	-
evm.model.unitig_4.5	An03g03030

Gene ID of H915-1	Gene ID of 513.88
evm.model.unitig_4.50	An15g07300
evm.model.unitig_4.500	An15g00950
evm.model.unitig_4.501	An15g00940
evm.model.unitig_4.502	An15g00930
evm.model.unitig_4.503	An15g00920
evm.model.unitig_4.504	An15g00910
evm.model.unitig_4.505	An15g00900
evm.model.unitig_4.506	An15g00890
evm.model.unitig_4.507	An15g00880
evm.model.unitig_4.508	An15g00870
evm.model.unitig_4.509	An15g00860
evm.model.unitig_4.510	An15g00850
evm.model.unitig_4.511	An15g00840
evm.model.unitig_4.512	An15g00830
evm.model.unitig_4.513	An15g00820
evm.model.unitig_4.514	An15g00810
evm.model.unitig_4.515	An15g00790
evm.model.unitig_4.516	An15g00770
evm.model.unitig_4.517	An15g00750
evm.model.unitig_4.518	An15g00740
evm.model.unitig_4.519	An15g00730
evm.model.unitig_4.52	An15g07260
evm.model.unitig_4.520	An15g00728
evm.model.unitig_4.521	An15g00700
evm.model.unitig_4.522	An15g00690
evm.model.unitig_4.523	An15g00680
evm.model.unitig_4.524	An15g00670
evm.model.unitig_4.525	An15g00660
evm.model.unitig_4.526	An15g00660
evm.model.unitig_4.527	An15g00650
evm.model.unitig_4.528	An15g00640
evm.model.unitig_4.529	An15g00630
evm.model.unitig_4.53	An15g07250
evm.model.unitig_4.530	An15g00620
evm.model.unitig_4.531	An15g00610
evm.model.unitig_4.532	An15g00600
evm.model.unitig_4.533	An15g00590
evm.model.unitig_4.534	An15g00580
evm.model.unitig_4.535	An15g00570
evm.model.unitig_4.536	An15g00560
evm.model.unitig_4.537	An15g00550
evm.model.unitig_4.538	An15g00540
evm.model.unitig_4.539	An15g00530

Gene ID of H915-1	Gene ID of 513.88
evm.model.unitig_4.54	An15g07240
evm.model.unitig_4.540	An15g00520
evm.model.unitig_4.541	An15g00510
evm.model.unitig_4.542	An15g00500
evm.model.unitig_4.543	An15g00480
evm.model.unitig_4.544	An15g00470
evm.model.unitig_4.545	An15g00460
evm.model.unitig_4.546	An15g00450
evm.model.unitig_4.547	An15g00440
evm.model.unitig_4.548	An15g00420
evm.model.unitig_4.549	An15g00410
evm.model.unitig_4.55	An15g07230
evm.model.unitig_4.550	An15g00400
evm.model.unitig_4.551	An15g00390
evm.model.unitig_4.552	An15g00390
evm.model.unitig_4.553	An15g00360
evm.model.unitig_4.554	An15g00350
evm.model.unitig_4.555	An15g00340
evm.model.unitig_4.556	An15g00330
evm.model.unitig_4.557	An15g00320
evm.model.unitig_4.558	An15g00310
evm.model.unitig_4.559	An15g00300
evm.model.unitig_4.56	An15g07220
evm.model.unitig_4.560	An15g00290
evm.model.unitig_4.561	An15g00280
evm.model.unitig_4.562	An15g00270
evm.model.unitig_4.563	An15g00260
evm.model.unitig_4.564	An15g00250
evm.model.unitig_4.565	An15g00240
evm.model.unitig_4.566	An15g00230
evm.model.unitig_4.567	An15g00220
evm.model.unitig_4.568	An15g00210
evm.model.unitig_4.569	-
evm.model.unitig_4.57	An15g07200
evm.model.unitig_4.570	An15g00200
evm.model.unitig_4.571	An15g00190
evm.model.unitig_4.572	An15g00170
evm.model.unitig_4.573	An15g00160
evm.model.unitig_4.574	An15g00150
evm.model.unitig_4.575	An15g00140
evm.model.unitig_4.576	An15g00120
evm.model.unitig_4.577	An15g00110
evm.model.unitig_4.578	An15g00100

Gene ID of H915-1	Gene ID of 513.88
evm.model.unitig_4.579	An15g00090
evm.model.unitig_4.58	An15g07190
evm.model.unitig_4.580	An15g00080
evm.model.unitig_4.581	An15g00070
evm.model.unitig_4.582	An15g00050
evm.model.unitig_4.583	An15g00030
evm.model.unitig_4.584	An15g00020
evm.model.unitig_4.585	An15g00010
evm.model.unitig_4.586	An02g01240
evm.model.unitig_4.59	An15g07160
evm.model.unitig_4.590	An13g02130
evm.model.unitig_4.591	An04g08800
evm.model.unitig_4.594	An15g05710
evm.model.unitig_4.595	An03g04570
evm.model.unitig_4.597	An14g00490
evm.model.unitig_4.6	An11g03070
evm.model.unitig_4.60	An15g07150
evm.model.unitig_4.600	An05g00010
evm.model.unitig_4.601	An05g00020
evm.model.unitig_4.603	An05g00040
evm.model.unitig_4.604	An05g00050
evm.model.unitig_4.605	An05g00060
evm.model.unitig_4.606	An05g00070
evm.model.unitig_4.607	An05g00080
evm.model.unitig_4.608	An05g00090
evm.model.unitig_4.609	An05g00100
evm.model.unitig_4.61	An15g07140
evm.model.unitig_4.610	An05g00110
evm.model.unitig_4.611	An05g00120
evm.model.unitig_4.612	An05g00130
evm.model.unitig_4.613	An05g00140
evm.model.unitig_4.614	An05g00160
evm.model.unitig_4.615	An05g00170
evm.model.unitig_4.616	An05g00180
evm.model.unitig_4.617	An05g00190
evm.model.unitig_4.618	An05g00200
evm.model.unitig_4.619	An05g00220
evm.model.unitig_4.620	An05g00280
evm.model.unitig_4.621	An05g00290
evm.model.unitig_4.622	An05g00310
evm.model.unitig_4.623	An05g00340
evm.model.unitig_4.624	An05g00350
evm.model.unitig_4.625	An05g00370

Gene ID of H915-1	Gene ID of 513.88
evm.model.unitig_4.626	An05g00390
evm.model.unitig_4.627	-
evm.model.unitig_4.628	An05g00440
evm.model.unitig_4.629	An05g00480
evm.model.unitig_4.63	An15g07120
evm.model.unitig_4.630	-
evm.model.unitig_4.631	An05g00490
evm.model.unitig_4.632	An05g00500
evm.model.unitig_4.633	An05g00510
evm.model.unitig_4.634	An05g00520
evm.model.unitig_4.635	-
evm.model.unitig_4.636	An05g00550
evm.model.unitig_4.637	An05g00570
evm.model.unitig_4.638	An05g00610
evm.model.unitig_4.639	An05g00630
evm.model.unitig_4.64	An15g07110
evm.model.unitig_4.640	An05g00640
evm.model.unitig_4.641	An05g00670
evm.model.unitig_4.642	An05g00690
evm.model.unitig_4.643	An05g00720
evm.model.unitig_4.644	An05g00730
evm.model.unitig_4.645	An05g00760
evm.model.unitig_4.646	An05g00780
evm.model.unitig_4.647	An05g00790
evm.model.unitig_4.648	An05g00820
evm.model.unitig_4.649	An05g00850
evm.model.unitig_4.65	An15g07090
evm.model.unitig_4.650	An05g00870
evm.model.unitig_4.651	An05g00880
evm.model.unitig_4.652	An05g00900
evm.model.unitig_4.653	An05g00930
evm.model.unitig_4.654	An05g00950
evm.model.unitig_4.655	An05g00960
evm.model.unitig_4.656	An05g00970
evm.model.unitig_4.657	An12g03500
evm.model.unitig_4.659	An12g03530
evm.model.unitig_4.66	An15g07080
evm.model.unitig_4.660	An12g03550
evm.model.unitig_4.661	An12g03570
evm.model.unitig_4.662	An12g03580
evm.model.unitig_4.663	An12g03600
evm.model.unitig_4.664	An12g03620
evm.model.unitig_4.665	An12g03650

Gene ID of H915-1	Gene ID of 513.88
evm.model.unitig_4.666	An12g03660
evm.model.unitig_4.667	An12g03670
evm.model.unitig_4.668	An12g03700
evm.model.unitig_4.669	An12g03710
evm.model.unitig_4.67	An15g07070
evm.model.unitig_4.670	An12g03720
evm.model.unitig_4.671	An12g03730
evm.model.unitig_4.672	An12g03760
evm.model.unitig_4.673	An12g03760
evm.model.unitig_4.674	An12g03830
evm.model.unitig_4.675	An12g03850
evm.model.unitig_4.676	An02g14290
evm.model.unitig_4.677	An12g03880
evm.model.unitig_4.678	An12g03890
evm.model.unitig_4.679	An12g03900
evm.model.unitig_4.68	An15g07060
evm.model.unitig_4.680	An12g03910
evm.model.unitig_4.681	An12g03920
evm.model.unitig_4.682	An12g03930
evm.model.unitig_4.683	An12g03940
evm.model.unitig_4.684	An12g03950
evm.model.unitig_4.685	An12g03960
evm.model.unitig_4.686	An12g03970
evm.model.unitig_4.687	An12g03990
evm.model.unitig_4.688	An12g04000
evm.model.unitig_4.689	An12g04010
evm.model.unitig_4.69	An15g07050
evm.model.unitig_4.690	An12g04020
evm.model.unitig_4.691	An12g04030
evm.model.unitig_4.693	An12g04050
evm.model.unitig_4.694	-
evm.model.unitig_4.695	An12g04060
evm.model.unitig_4.696	An12g04070
evm.model.unitig_4.697	An12g04090
evm.model.unitig_4.698	An12g04110
evm.model.unitig_4.699	An12g04120
evm.model.unitig_4.7	An15g07930
evm.model.unitig_4.70	An15g07040
evm.model.unitig_4.701	An12g04140
evm.model.unitig_4.702	An12g04170
evm.model.unitig_4.703	An12g04180
evm.model.unitig_4.704	An12g04190
evm.model.unitig_4.705	An12g04210

Gene ID of H915-1	Gene ID of 513.88
evm.model.unitig_4.706	An12g04220
evm.model.unitig_4.707	An12g04250
evm.model.unitig_4.708	An12g04260
evm.model.unitig_4.709	An12g04280
evm.model.unitig_4.71	An08g10830
evm.model.unitig_4.710	An12g04310
evm.model.unitig_4.711	An12g04330
evm.model.unitig_4.712	An12g04340
evm.model.unitig_4.713	An12g04350
evm.model.unitig_4.714	An12g04360
evm.model.unitig_4.715	An12g04370
evm.model.unitig_4.716	An12g04380
evm.model.unitig_4.717	An12g04390
evm.model.unitig_4.718	An12g04450
evm.model.unitig_4.719	An12g04470
evm.model.unitig_4.72	An15g07000
evm.model.unitig_4.720	An12g04480
evm.model.unitig_4.721	An12g04490
evm.model.unitig_4.722	An12g04500
evm.model.unitig_4.723	An12g04510
evm.model.unitig_4.724	An12g04520
evm.model.unitig_4.725	An12g10020
evm.model.unitig_4.726	An12g10020
evm.model.unitig_4.727	An12g04540
evm.model.unitig_4.728	An12g04560
evm.model.unitig_4.729	An12g04570
evm.model.unitig_4.73	An15g06990
evm.model.unitig_4.730	An12g04580
evm.model.unitig_4.731	An12g04590
evm.model.unitig_4.732	An12g04600
evm.model.unitig_4.733	An12g04610
evm.model.unitig_4.734	An12g04620
evm.model.unitig_4.735	An12g04630
evm.model.unitig_4.736	An12g04640
evm.model.unitig_4.737	An12g04650
evm.model.unitig_4.738	An12g04655
evm.model.unitig_4.739	An12g04660
evm.model.unitig_4.74	An15g06980
evm.model.unitig_4.740	An12g04670
evm.model.unitig_4.741	An12g04680
evm.model.unitig_4.742	An12g04690
evm.model.unitig_4.743	An12g04700
evm.model.unitig_4.744	An12g04710

Gene ID of H915-1	Gene ID of 513.88
evm.model.unitig_4.745	An12g04720
evm.model.unitig_4.746	An12g04730
evm.model.unitig_4.747	An12g04740
evm.model.unitig_4.748	An12g04750
evm.model.unitig_4.749	An12g04760
evm.model.unitig_4.75	An15g06970
evm.model.unitig_4.750	An12g04770
evm.model.unitig_4.751	An12g04780
evm.model.unitig_4.752	An12g04790
evm.model.unitig_4.753	An12g04800
evm.model.unitig_4.754	An12g04810
evm.model.unitig_4.755	An12g04820
evm.model.unitig_4.756	An12g04830
evm.model.unitig_4.757	An12g04840
evm.model.unitig_4.758	An12g04860
evm.model.unitig_4.759	An12g04870
evm.model.unitig_4.760	An12g04880
evm.model.unitig_4.761	An12g04890
evm.model.unitig_4.762	An12g04910
evm.model.unitig_4.763	An12g04920
evm.model.unitig_4.764	An12g04930
evm.model.unitig_4.765	An12g04900
evm.model.unitig_4.766	An12g04940
evm.model.unitig_4.767	An12g04960
evm.model.unitig_4.768	An12g04960
evm.model.unitig_4.769	An12g04990
evm.model.unitig_4.77	An15g06960
evm.model.unitig_4.770	An12g05000
evm.model.unitig_4.771	An12g05010
evm.model.unitig_4.772	An12g05030
evm.model.unitig_4.773	An12g05050
evm.model.unitig_4.774	An12g05060
evm.model.unitig_4.775	An12g05070
evm.model.unitig_4.776	An12g05080
evm.model.unitig_4.777	An12g05090
evm.model.unitig_4.778	An12g05100
evm.model.unitig_4.779	An12g05110
evm.model.unitig_4.780	An12g05150
evm.model.unitig_4.781	An12g05170
evm.model.unitig_4.782	An12g05180
evm.model.unitig_4.783	An12g05190
evm.model.unitig_4.784	An12g05200
evm.model.unitig_4.785	An12g05210

Gene ID of H915-1	Gene ID of 513.88
evm.model.unitig_4.786	An12g05220
evm.model.unitig_4.787	An12g05240
evm.model.unitig_4.788	An12g05250
evm.model.unitig_4.789	An12g05260
evm.model.unitig_4.79	An15g06925
evm.model.unitig_4.790	An12g05280
evm.model.unitig_4.791	An09g01220
evm.model.unitig_4.792	An12g05310
evm.model.unitig_4.793	An12g05320
evm.model.unitig_4.794	An15g00840
evm.model.unitig_4.795	An12g05360
evm.model.unitig_4.796	An12g05370
evm.model.unitig_4.797	An12g05380
evm.model.unitig_4.798	An12g05390
evm.model.unitig_4.799	An12g05400
evm.model.unitig_4.8	An15g07920
evm.model.unitig_4.80	An15g06920
evm.model.unitig_4.800	An12g05420
evm.model.unitig_4.801	An05g01960
evm.model.unitig_4.802	An12g05440
evm.model.unitig_4.803	An18g02760
evm.model.unitig_4.804	An12g05460
evm.model.unitig_4.805	An12g05470
evm.model.unitig_4.806	An12g05490
evm.model.unitig_4.807	An12g05510
evm.model.unitig_4.808	An12g05570
evm.model.unitig_4.809	An12g05580
evm.model.unitig_4.81	An15g06910
evm.model.unitig_4.810	An12g05590
evm.model.unitig_4.811	An12g05600
evm.model.unitig_4.812	An12g05610
evm.model.unitig_4.813	An01g14020
evm.model.unitig_4.814	An13g03050
evm.model.unitig_4.816	An11g09010
evm.model.unitig_4.817	An12g05630
evm.model.unitig_4.818	An12g05640
evm.model.unitig_4.819	An12g05650
evm.model.unitig_4.82	An15g06900
evm.model.unitig_4.820	An12g05660
evm.model.unitig_4.821	An12g05670
evm.model.unitig_4.822	An12g05680
evm.model.unitig_4.823	An12g05700
evm.model.unitig_4.824	An12g05710

Gene ID of H915-1	Gene ID of 513.88
evm.model.unitig_4.825	An12g05720
evm.model.unitig_4.826	An12g05740
evm.model.unitig_4.827	An12g05750
evm.model.unitig_4.828	An12g05820
evm.model.unitig_4.829	An12g05830
evm.model.unitig_4.83	An15g06880
evm.model.unitig_4.830	An12g05840
evm.model.unitig_4.831	-
evm.model.unitig_4.832	An12g05870
evm.model.unitig_4.834	An12g05890
evm.model.unitig_4.836	An12g05930
evm.model.unitig_4.837	An12g05960
evm.model.unitig_4.838	An12g05970
evm.model.unitig_4.839	An12g05990
evm.model.unitig_4.84	An15g06870
evm.model.unitig_4.840	An12g06000
evm.model.unitig_4.841	An12g06010
evm.model.unitig_4.842	An12g06030
evm.model.unitig_4.843	An02g11030
evm.model.unitig_4.844	An12g06050
evm.model.unitig_4.845	An12g06060
evm.model.unitig_4.846	An08g01910
evm.model.unitig_4.847	An03g04820
evm.model.unitig_4.848	An12g06130
evm.model.unitig_4.85	An15g06860
evm.model.unitig_4.852	An12g06180
evm.model.unitig_4.853	An12g06190
evm.model.unitig_4.854	An12g06200
evm.model.unitig_4.855	An12g06250
evm.model.unitig_4.856	An12g06290
evm.model.unitig_4.857	An12g06300
evm.model.unitig_4.858	An12g06310
evm.model.unitig_4.859	An12g06340
evm.model.unitig_4.86	An15g06850
evm.model.unitig_4.860	An12g06350
evm.model.unitig_4.861	-
evm.model.unitig_4.862	An12g06400
evm.model.unitig_4.863	An12g06440
evm.model.unitig_4.864	An12g06480
evm.model.unitig_4.865	An12g06490
evm.model.unitig_4.866	An12g06500
evm.model.unitig_4.867	An12g06510
evm.model.unitig_4.868	An12g06520

Gene ID of H915-1	Gene ID of 513.88
evm.model.unitig_4.869	An12g06530
evm.model.unitig_4.87	An15g06840
evm.model.unitig_4.870	An12g06540
evm.model.unitig_4.871	An12g06550
evm.model.unitig_4.872	An12g06560
evm.model.unitig_4.873	An12g06580
evm.model.unitig_4.874	An12g06590
evm.model.unitig_4.875	An16g01830
evm.model.unitig_4.876	An18g00040
evm.model.unitig_4.877	An01g03030
evm.model.unitig_4.878	An16g00240
evm.model.unitig_4.879	An01g03040
evm.model.unitig_4.88	An15g06820
evm.model.unitig_4.880	An14g01000
evm.model.unitig_4.881	An14g01590
evm.model.unitig_4.882	An02g07550
evm.model.unitig_4.883	An02g07560
evm.model.unitig_4.884	An01g11560
evm.model.unitig_4.886	An11g00680
evm.model.unitig_4.887	An17g00930
evm.model.unitig_4.888	An11g05920
evm.model.unitig_4.889	An12g10950
evm.model.unitig_4.89	-
evm.model.unitig_4.890	An02g08330
evm.model.unitig_4.892	An12g02850
evm.model.unitig_4.893	An01g08550
evm.model.unitig_4.894	An16g02770
evm.model.unitig_4.895	An01g00630
evm.model.unitig_4.896	An09g02880
evm.model.unitig_4.898	An12g07020
evm.model.unitig_4.899	An12g07060
evm.model.unitig_4.9	An15g07910
evm.model.unitig_4.90	An15g06810
evm.model.unitig_4.900	An12g07070
evm.model.unitig_4.901	An12g07070
evm.model.unitig_4.902	An12g07090
evm.model.unitig_4.903	-
evm.model.unitig_4.904	An12g07130
evm.model.unitig_4.905	An12g07140
evm.model.unitig_4.906	An12g07150
evm.model.unitig_4.907	An12g07170
evm.model.unitig_4.908	An12g07180
evm.model.unitig_4.909	An12g07190

Gene ID of H915-1	Gene ID of 513.88
evm.model.unitig_4.91	An15g06790
evm.model.unitig_4.911	An12g07220
evm.model.unitig_4.912	An12g07230
evm.model.unitig_4.913	An12g07240
evm.model.unitig_4.914	An12g07250
evm.model.unitig_4.915	An12g07260
evm.model.unitig_4.916	-
evm.model.unitig_4.917	An12g07280
evm.model.unitig_4.92	An15g06780
evm.model.unitig_4.920	An11g09490
evm.model.unitig_4.922	An12g06860
evm.model.unitig_4.923	-
evm.model.unitig_4.925	An07g01930
evm.model.unitig_4.928	An12g06930
evm.model.unitig_4.929	An12g06670
evm.model.unitig_4.93	An15g06770
evm.model.unitig_4.931	An11g03400
evm.model.unitig_4.933	An12g06610
evm.model.unitig_4.934	An12g06610
evm.model.unitig_4.935	An16g04250
evm.model.unitig_4.936	An12g07300
evm.model.unitig_4.937	An12g07320
evm.model.unitig_4.938	An12g07330
evm.model.unitig_4.939	An12g07360
evm.model.unitig_4.94	An15g06760
evm.model.unitig_4.940	An12g07370
evm.model.unitig_4.941	An12g07380
evm.model.unitig_4.942	An12g07390
evm.model.unitig_4.943	An12g07400
evm.model.unitig_4.944	An12g07420
evm.model.unitig_4.945	An12g07430
evm.model.unitig_4.946	An12g07450
evm.model.unitig_4.947	An12g07490
evm.model.unitig_4.948	An12g07500
evm.model.unitig_4.949	An12g07510
evm.model.unitig_4.95	An15g06750
evm.model.unitig_4.950	An12g07530
evm.model.unitig_4.951	An12g07540
evm.model.unitig_4.952	An12g07550
evm.model.unitig_4.953	An12g07570
evm.model.unitig_4.954	An12g07580
evm.model.unitig_4.955	An12g07590
evm.model.unitig_4.956	An12g07600

Gene ID of H915-1	Gene ID of 513.88
evm.model.unitig_4.957	An12g07620
evm.model.unitig_4.958	An12g07630
evm.model.unitig_4.959	An12g07660
evm.model.unitig_4.96	An15g06740
evm.model.unitig_4.960	An12g07670
evm.model.unitig_4.961	An12g07690
evm.model.unitig_4.963	An12g07710
evm.model.unitig_4.964	An12g07715
evm.model.unitig_4.965	An12g07720
evm.model.unitig_4.966	An12g07730
evm.model.unitig_4.967	An12g07740
evm.model.unitig_4.968	An12g07750
evm.model.unitig_4.969	An12g07760
evm.model.unitig_4.97	An15g06730
evm.model.unitig_4.970	An12g07770
evm.model.unitig_4.971	An12g07780
evm.model.unitig_4.972	An12g07800
evm.model.unitig_4.973	An12g07810
evm.model.unitig_4.974	An12g07830
evm.model.unitig_4.975	An12g07840
evm.model.unitig_4.976	An12g07850
evm.model.unitig_4.977	An12g07860
evm.model.unitig_4.978	An12g07870
evm.model.unitig_4.979	An12g07880
evm.model.unitig_4.98	An15g06720
evm.model.unitig_4.980	An12g07900
evm.model.unitig_4.981	An12g07910
evm.model.unitig_4.982	An12g07950
evm.model.unitig_4.983	An12g07960
evm.model.unitig_4.984	An12g08000
evm.model.unitig_4.985	-
evm.model.unitig_4.986	An12g08020
evm.model.unitig_4.987	An12g08030
evm.model.unitig_4.988	An12g08060
evm.model.unitig_4.989	An12g08090
evm.model.unitig_4.99	An15g06710
evm.model.unitig_4.990	An12g08100
evm.model.unitig_4.991	An12g08110
evm.model.unitig_4.994	An12g08230
evm.model.unitig_4.995	An12g08240
evm.model.unitig_4.996	An12g08260
evm.model.unitig_4.997	An12g08270
evm.model.unitig_4.998	An12g08280

Gene ID of H915-1	Gene ID of 513.88
evm.model.unitig_4.999	An04g09580
evm.model.unitig_5.1	An19g00100
evm.model.unitig_5.10	An18g00280
evm.model.unitig_5.100	An14g06210
evm.model.unitig_5.1000	An09g01110
evm.model.unitig_5.1001	An09g01100
evm.model.unitig_5.1002	An09g01090
evm.model.unitig_5.1003	An09g01080
evm.model.unitig_5.1004	An09g01070
evm.model.unitig_5.1005	An09g01050
evm.model.unitig_5.1006	An09g01030
evm.model.unitig_5.1007	An09g01020
evm.model.unitig_5.1008	An09g01020
evm.model.unitig_5.1009	An09g01010
evm.model.unitig_5.101	An14g06200
evm.model.unitig_5.1010	An09g00800
evm.model.unitig_5.1011	An09g00810
evm.model.unitig_5.1012	An09g00820
evm.model.unitig_5.1013	An09g00830
evm.model.unitig_5.1014	An09g00840
evm.model.unitig_5.1015	An09g00850
evm.model.unitig_5.1016	An09g00860
evm.model.unitig_5.1017	An09g00870
evm.model.unitig_5.1018	An09g00880
evm.model.unitig_5.1019	An09g00890
evm.model.unitig_5.102	-
evm.model.unitig_5.1020	An09g00920
evm.model.unitig_5.1021	An09g00930
evm.model.unitig_5.1022	An09g00950
evm.model.unitig_5.1024	An09g00700
evm.model.unitig_5.1025	An09g00690
evm.model.unitig_5.1026	An09g00680
evm.model.unitig_5.1027	An09g00670
evm.model.unitig_5.1028	An09g00660
evm.model.unitig_5.1029	An09g00650
evm.model.unitig_5.103	An14g06190
evm.model.unitig_5.1030	An09g00640
evm.model.unitig_5.1031	An09g00630
evm.model.unitig_5.1032	An09g00620
evm.model.unitig_5.1033	An09g00610
evm.model.unitig_5.1034	An09g00600
evm.model.unitig_5.1035	An09g00590
evm.model.unitig_5.1036	An09g00580

Gene ID of H915-1	Gene ID of 513.88
evm.model.unitig_5.1037	An09g00570
evm.model.unitig_5.1038	An09g00560
evm.model.unitig_5.1039	An09g00550
evm.model.unitig_5.104	An14g06180
evm.model.unitig_5.1040	An09g00540
evm.model.unitig_5.1041	An09g00530
evm.model.unitig_5.1042	An09g00520
evm.model.unitig_5.1043	An09g00500
evm.model.unitig_5.1044	-
evm.model.unitig_5.1045	An09g00480
evm.model.unitig_5.1046	An09g00470
evm.model.unitig_5.1047	An09g00450
evm.model.unitig_5.1048	-
evm.model.unitig_5.1049	An09g00430
evm.model.unitig_5.105	An14g06170
evm.model.unitig_5.1050	An09g00420
evm.model.unitig_5.1051	An09g00400
evm.model.unitig_5.1052	An09g00390
evm.model.unitig_5.1053	An09g00380
evm.model.unitig_5.1054	An09g00380
evm.model.unitig_5.1055	An09g00380
evm.model.unitig_5.1056	An09g00360
evm.model.unitig_5.1058	An09g00320
evm.model.unitig_5.1059	An09g00310
evm.model.unitig_5.106	An06g01430
evm.model.unitig_5.1060	An09g00260
evm.model.unitig_5.1061	-
evm.model.unitig_5.1062	An16g01250
evm.model.unitig_5.1063	An09g00200
evm.model.unitig_5.1064	An09g00190
evm.model.unitig_5.1065	An09g00170
evm.model.unitig_5.1066	An09g00160
evm.model.unitig_5.1067	An09g00120
evm.model.unitig_5.1068	An09g00100
evm.model.unitig_5.1069	An09g00070
evm.model.unitig_5.107	An14g06110
evm.model.unitig_5.1070	An09g00060
evm.model.unitig_5.1071	An09g00060
evm.model.unitig_5.1072	An09g00030
evm.model.unitig_5.1073	An09g00010
evm.model.unitig_5.108	An14g06100
evm.model.unitig_5.109	An14g06090
evm.model.unitig_5.11	An14g07300

Gene ID of H915-1	Gene ID of 513.88
evm.model.unitig_5.110	An14g06070
evm.model.unitig_5.111	An14g06060
evm.model.unitig_5.112	An14g06050
evm.model.unitig_5.113	An14g06040
evm.model.unitig_5.114	An14g06020
evm.model.unitig_5.115	An14g06010
evm.model.unitig_5.116	An14g06000
evm.model.unitig_5.117	An14g05990
evm.model.unitig_5.118	An14g05980
evm.model.unitig_5.119	An14g05970
evm.model.unitig_5.12	An14g07270
evm.model.unitig_5.120	An14g05960
evm.model.unitig_5.122	An14g05920
evm.model.unitig_5.123	An14g05910
evm.model.unitig_5.124	An14g05900
evm.model.unitig_5.125	An14g05890
evm.model.unitig_5.126	An14g05880
evm.model.unitig_5.127	An14g05870
evm.model.unitig_5.129	An14g05850
evm.model.unitig_5.13	An14g07260
evm.model.unitig_5.130	An14g05840
evm.model.unitig_5.131	An14g05830
evm.model.unitig_5.132	An14g05820
evm.model.unitig_5.133	An14g05810
evm.model.unitig_5.134	An14g05800
evm.model.unitig_5.135	An14g05790
evm.model.unitig_5.136	An14g05780
evm.model.unitig_5.137	An14g05765
evm.model.unitig_5.138	An14g05760
evm.model.unitig_5.139	An14g05750
evm.model.unitig_5.14	An01g14450
evm.model.unitig_5.140	An14g05730
evm.model.unitig_5.141	An14g05670
evm.model.unitig_5.142	An14g05630
evm.model.unitig_5.143	An14g05600
evm.model.unitig_5.144	An14g05590
evm.model.unitig_5.145	An14g05580
evm.model.unitig_5.146	An14g05550
evm.model.unitig_5.147	An14g05540
evm.model.unitig_5.148	An14g05530
evm.model.unitig_5.149	An14g05510
evm.model.unitig_5.15	An14g07240
evm.model.unitig_5.150	An14g05500

Gene ID of H915-1	Gene ID of 513.88
evm.model.unitig_5.151	An14g05490
evm.model.unitig_5.152	An14g05470
evm.model.unitig_5.153	An14g05460
evm.model.unitig_5.154	An14g05450
evm.model.unitig_5.155	An14g05430
evm.model.unitig_5.156	An14g05420
evm.model.unitig_5.157	An14g05410
evm.model.unitig_5.158	An14g05380
evm.model.unitig_5.159	An14g05370
evm.model.unitig_5.16	An14g07230
evm.model.unitig_5.160	An14g05350
evm.model.unitig_5.161	An14g05340
evm.model.unitig_5.162	An14g05330
evm.model.unitig_5.163	An14g05320
evm.model.unitig_5.164	An14g05310
evm.model.unitig_5.165	An14g05300
evm.model.unitig_5.166	An14g05290
evm.model.unitig_5.167	An14g05280
evm.model.unitig_5.168	An14g05260
evm.model.unitig_5.169	An14g05250
evm.model.unitig_5.17	An14g07220
evm.model.unitig_5.170	An14g05190
evm.model.unitig_5.171	An14g05170
evm.model.unitig_5.172	An14g05160
evm.model.unitig_5.173	An14g05150
evm.model.unitig_5.174	An14g05140
evm.model.unitig_5.175	An14g05130
evm.model.unitig_5.176	An14g05120
evm.model.unitig_5.177	An14g05110
evm.model.unitig_5.178	An14g05100
evm.model.unitig_5.18	An14g07210
evm.model.unitig_5.180	An14g05070
evm.model.unitig_5.181	An14g05050
evm.model.unitig_5.182	An14g05020
evm.model.unitig_5.183	An14g05010
evm.model.unitig_5.184	An14g05000
evm.model.unitig_5.185	An14g04990
evm.model.unitig_5.186	An14g04980
evm.model.unitig_5.187	An14g04970
evm.model.unitig_5.188	An14g04960
evm.model.unitig_5.189	An14g04950
evm.model.unitig_5.19	An14g07200
evm.model.unitig_5.190	An14g04940

Gene ID of H915-1	Gene ID of 513.88
evm.model.unitig_5.191	An14g04930
evm.model.unitig_5.192	An14g04920
evm.model.unitig_5.193	An14g04910
evm.model.unitig_5.195	An14g04890
evm.model.unitig_5.196	An14g04880
evm.model.unitig_5.197	An14g04870
evm.model.unitig_5.198	An14g04860
evm.model.unitig_5.199	An14g04840
evm.model.unitig_5.2	An19g00100
evm.model.unitig_5.20	An14g07190
evm.model.unitig_5.200	An14g04830
evm.model.unitig_5.201	An14g04800
evm.model.unitig_5.202	An14g04770
evm.model.unitig_5.203	An14g04760
evm.model.unitig_5.204	An14g04750
evm.model.unitig_5.205	An14g04740
evm.model.unitig_5.206	An14g04730
evm.model.unitig_5.207	An14g04720
evm.model.unitig_5.208	An14g04710
evm.model.unitig_5.209	An14g04680
evm.model.unitig_5.21	An14g07180
evm.model.unitig_5.210	An14g04670
evm.model.unitig_5.211	An14g04650
evm.model.unitig_5.212	An14g04640
evm.model.unitig_5.213	An14g04620
evm.model.unitig_5.214	An14g04590
evm.model.unitig_5.215	An14g04560
evm.model.unitig_5.216	An14g04530
evm.model.unitig_5.217	An04g00830
evm.model.unitig_5.218	An14g04490
evm.model.unitig_5.220	An14g04420
evm.model.unitig_5.221	An14g04410
evm.model.unitig_5.222	An14g04400
evm.model.unitig_5.223	An14g04370
evm.model.unitig_5.224	An14g04340
evm.model.unitig_5.225	An14g04330
evm.model.unitig_5.226	An14g04320
evm.model.unitig_5.227	An14g04300
evm.model.unitig_5.228	An14g04290
evm.model.unitig_5.229	An14g04280
evm.model.unitig_5.23	An14g07160
evm.model.unitig_5.230	An14g04260
evm.model.unitig_5.231	An14g04240

Gene ID of H915-1	Gene ID of 513.88
evm.model.unitig_5.232	An14g04210
evm.model.unitig_5.233	An14g04200
evm.model.unitig_5.234	An14g04190
evm.model.unitig_5.235	An14g04180
evm.model.unitig_5.236	An14g04170
evm.model.unitig_5.237	An14g04160
evm.model.unitig_5.238	An14g04140
evm.model.unitig_5.239	An14g04100
evm.model.unitig_5.24	An14g07150
evm.model.unitig_5.240	An14g04090
evm.model.unitig_5.241	An14g04080
evm.model.unitig_5.242	An14g04070
evm.model.unitig_5.243	An14g04060
evm.model.unitig_5.244	-
evm.model.unitig_5.245	An14g04050
evm.model.unitig_5.246	An14g04040
evm.model.unitig_5.247	An14g04010
evm.model.unitig_5.248	An14g04000
evm.model.unitig_5.249	An14g03990
evm.model.unitig_5.25	An14g07140
evm.model.unitig_5.250	An08g04300
evm.model.unitig_5.251	An14g03950
evm.model.unitig_5.252	An14g03930
evm.model.unitig_5.253	An14g03920
evm.model.unitig_5.254	An14g03910
evm.model.unitig_5.255	An14g03890
evm.model.unitig_5.256	An14g03870
evm.model.unitig_5.257	An14g03850
evm.model.unitig_5.258	An14g03820
evm.model.unitig_5.259	An14g03810
evm.model.unitig_5.26	An14g07130
evm.model.unitig_5.260	An14g03800
evm.model.unitig_5.261	An14g03790
evm.model.unitig_5.262	An14g03780
evm.model.unitig_5.263	An14g03770
evm.model.unitig_5.264	An14g03760
evm.model.unitig_5.265	An14g03740
evm.model.unitig_5.266	An14g03730
evm.model.unitig_5.267	An14g03720
evm.model.unitig_5.268	An14g03710
evm.model.unitig_5.269	An14g03700
evm.model.unitig_5.27	An14g07090
evm.model.unitig_5.270	An14g03660

Gene ID of H915-1	Gene ID of 513.88
evm.model.unitig_5.271	An14g03640
evm.model.unitig_5.272	An14g03630
evm.model.unitig_5.273	An14g03620
evm.model.unitig_5.274	An14g03610
evm.model.unitig_5.275	An14g03600
evm.model.unitig_5.276	An14g03590
evm.model.unitig_5.277	An14g03580
evm.model.unitig_5.278	An14g03570
evm.model.unitig_5.279	An14g03560
evm.model.unitig_5.28	An14g07070
evm.model.unitig_5.280	An14g03550
evm.model.unitig_5.281	An14g03530
evm.model.unitig_5.282	An14g03520
evm.model.unitig_5.283	An14g03510
evm.model.unitig_5.284	An14g03500
evm.model.unitig_5.286	An14g03470
evm.model.unitig_5.287	An14g03460
evm.model.unitig_5.288	An14g03450
evm.model.unitig_5.289	An14g03440
evm.model.unitig_5.29	An14g07060
evm.model.unitig_5.290	An14g03430
evm.model.unitig_5.291	An14g03420
evm.model.unitig_5.292	An14g03410
evm.model.unitig_5.293	An14g03400
evm.model.unitig_5.294	An14g03390
evm.model.unitig_5.295	An14g03370
evm.model.unitig_5.296	An14g03360
evm.model.unitig_5.297	An14g03340
evm.model.unitig_5.298	An14g03330
evm.model.unitig_5.299	An14g03310
evm.model.unitig_5.3	An14g07400
evm.model.unitig_5.30	An14g07050
evm.model.unitig_5.300	An14g03300
evm.model.unitig_5.301	An14g03290
evm.model.unitig_5.302	An14g03280
evm.model.unitig_5.303	An14g03270
evm.model.unitig_5.304	An14g03250
evm.model.unitig_5.305	An14g03240
evm.model.unitig_5.306	An13g03440
evm.model.unitig_5.307	An14g03220
evm.model.unitig_5.308	An14g03210
evm.model.unitig_5.309	An14g03200
evm.model.unitig_5.31	An14g07040

Gene ID of H915-1	Gene ID of 513.88
evm.model.unitig_5.310	An14g03190
evm.model.unitig_5.311	An14g03160
evm.model.unitig_5.312	An14g03140
evm.model.unitig_5.313	An14g03120
evm.model.unitig_5.314	An14g03110
evm.model.unitig_5.315	An14g03100
evm.model.unitig_5.316	An14g03090
evm.model.unitig_5.317	An14g03080
evm.model.unitig_5.318	An14g03070
evm.model.unitig_5.319	An14g03060
evm.model.unitig_5.32	An14g07030
evm.model.unitig_5.320	An14g03050
evm.model.unitig_5.321	An14g03040
evm.model.unitig_5.322	An14g03030
evm.model.unitig_5.323	An14g03020
evm.model.unitig_5.324	An14g03000
evm.model.unitig_5.325	An14g02990
evm.model.unitig_5.326	An14g02980
evm.model.unitig_5.327	An14g02970
evm.model.unitig_5.328	An14g02960
evm.model.unitig_5.329	An14g02950
evm.model.unitig_5.33	An14g07010
evm.model.unitig_5.330	An14g02940
evm.model.unitig_5.331	An14g02570
evm.model.unitig_5.332	An14g02920
evm.model.unitig_5.333	An14g02910
evm.model.unitig_5.335	An14g02900
evm.model.unitig_5.336	An14g02890
evm.model.unitig_5.337	An14g02880
evm.model.unitig_5.338	An14g02870
evm.model.unitig_5.339	An14g02860
evm.model.unitig_5.34	An11g09000
evm.model.unitig_5.340	An14g02840
evm.model.unitig_5.341	An14g02830
evm.model.unitig_5.342	An01g11750
evm.model.unitig_5.343	-
evm.model.unitig_5.346	An02g09120
evm.model.unitig_5.347	An14g02780
evm.model.unitig_5.348	An14g02760
evm.model.unitig_5.349	An14g02750
evm.model.unitig_5.35	An14g06980
evm.model.unitig_5.350	An14g02720
evm.model.unitig_5.351	An14g02700

Gene ID of H915-1	Gene ID of 513.88
evm.model.unitig_5.352	An14g02670
evm.model.unitig_5.353	An14g02660
evm.model.unitig_5.354	An14g02650
evm.model.unitig_5.355	An14g02640
evm.model.unitig_5.356	An14g02630
evm.model.unitig_5.357	An14g02620
evm.model.unitig_5.358	An14g02610
evm.model.unitig_5.359	An14g02590
evm.model.unitig_5.36	An14g06970
evm.model.unitig_5.360	An14g02570
evm.model.unitig_5.361	An14g02560
evm.model.unitig_5.362	An08g12200
evm.model.unitig_5.363	An14g02540
evm.model.unitig_5.366	An14g02500
evm.model.unitig_5.367	An14g02490
evm.model.unitig_5.368	An14g02470
evm.model.unitig_5.369	An14g02460
evm.model.unitig_5.37	An14g06960
evm.model.unitig_5.370	An14g02450
evm.model.unitig_5.371	An14g02420
evm.model.unitig_5.372	An14g02410
evm.model.unitig_5.373	An14g02390
evm.model.unitig_5.374	An14g02370
evm.model.unitig_5.375	An14g02360
evm.model.unitig_5.376	An14g02350
evm.model.unitig_5.378	An14g02320
evm.model.unitig_5.379	An14g02300
evm.model.unitig_5.38	An14g06950
evm.model.unitig_5.380	An14g02290
evm.model.unitig_5.381	An14g02280
evm.model.unitig_5.382	An14g02260
evm.model.unitig_5.383	An14g02250
evm.model.unitig_5.384	An14g02240
evm.model.unitig_5.385	An14g02230
evm.model.unitig_5.386	An14g02210
evm.model.unitig_5.387	An14g02200
evm.model.unitig_5.388	An14g02190
evm.model.unitig_5.389	An14g02180
evm.model.unitig_5.390	An14g02170
evm.model.unitig_5.391	An14g02160
evm.model.unitig_5.392	An14g02150
evm.model.unitig_5.393	-
evm.model.unitig_5.394	An14g02130

Gene ID of H915-1	Gene ID of 513.88
evm.model.unitig_5.395	An14g02100
evm.model.unitig_5.396	An14g02090
evm.model.unitig_5.397	An14g02080
evm.model.unitig_5.398	An14g02070
evm.model.unitig_5.399	An14g02060
evm.model.unitig_5.4	An14g07390
evm.model.unitig_5.40	An14g06940
evm.model.unitig_5.400	An14g02040
evm.model.unitig_5.401	An15g06750
evm.model.unitig_5.402	An14g02030
evm.model.unitig_5.403	An14g02010
evm.model.unitig_5.404	An14g01990
evm.model.unitig_5.405	An14g01980
evm.model.unitig_5.406	An14g01970
evm.model.unitig_5.407	An14g01960
evm.model.unitig_5.408	An14g01950
evm.model.unitig_5.409	An14g01940
evm.model.unitig_5.41	An14g06930
evm.model.unitig_5.410	An14g01910
evm.model.unitig_5.411	An08g08860
evm.model.unitig_5.412	An13g02880
evm.model.unitig_5.413	An14g01880
evm.model.unitig_5.414	An14g01870
evm.model.unitig_5.415	An14g01860
evm.model.unitig_5.416	An14g01850
evm.model.unitig_5.417	An14g01840
evm.model.unitig_5.418	An14g01820
evm.model.unitig_5.419	An14g01810
evm.model.unitig_5.42	An14g06920
evm.model.unitig_5.420	An14g01800
evm.model.unitig_5.421	An14g01770
evm.model.unitig_5.422	An14g01760
evm.model.unitig_5.423	An14g01730
evm.model.unitig_5.424	An14g01710
evm.model.unitig_5.425	An14g01700
evm.model.unitig_5.426	An14g01690
evm.model.unitig_5.427	An14g01685
evm.model.unitig_5.428	An14g01680
evm.model.unitig_5.429	An14g01670
evm.model.unitig_5.43	An14g06910
evm.model.unitig_5.430	An14g01660
evm.model.unitig_5.431	An14g01630
evm.model.unitig_5.432	An14g01620

Gene ID of H915-1	Gene ID of 513.88
evm.model.unitig_5.434	An14g01600
evm.model.unitig_5.435	An14g01590
evm.model.unitig_5.436	An14g01580
evm.model.unitig_5.437	An14g01570
evm.model.unitig_5.438	An14g01560
evm.model.unitig_5.439	An14g01550
evm.model.unitig_5.44	An14g06900
evm.model.unitig_5.440	An14g01540
evm.model.unitig_5.441	An14g01530
evm.model.unitig_5.442	An14g01500
evm.model.unitig_5.443	An14g01490
evm.model.unitig_5.444	An14g01480
evm.model.unitig_5.445	An14g01440
evm.model.unitig_5.446	An14g01450
evm.model.unitig_5.447	An14g01460
evm.model.unitig_5.448	An14g01470
evm.model.unitig_5.449	An14g01430
evm.model.unitig_5.45	An14g06890
evm.model.unitig_5.450	An14g01420
evm.model.unitig_5.451	An14g01410
evm.model.unitig_5.452	An14g01390
evm.model.unitig_5.453	An14g01380
evm.model.unitig_5.454	An14g01370
evm.model.unitig_5.456	An14g01330
evm.model.unitig_5.457	An14g01280
evm.model.unitig_5.458	An14g01255
evm.model.unitig_5.459	An14g01253
evm.model.unitig_5.46	An14g06880
evm.model.unitig_5.460	An14g01245
evm.model.unitig_5.461	An14g01235
evm.model.unitig_5.462	An02g02250
evm.model.unitig_5.463	An14g01210
evm.model.unitig_5.464	An14g01190
evm.model.unitig_5.465	An14g01180
evm.model.unitig_5.466	An14g01180
evm.model.unitig_5.467	An14g01170
evm.model.unitig_5.468	An14g01160
evm.model.unitig_5.469	An14g01150
evm.model.unitig_5.47	-
evm.model.unitig_5.470	An14g01140
evm.model.unitig_5.471	An14g01130
evm.model.unitig_5.472	An14g01120
evm.model.unitig_5.473	An14g01110

Gene ID of H915-1	Gene ID of 513.88
evm.model.unitig_5.474	An14g01080
evm.model.unitig_5.476	An14g01070
evm.model.unitig_5.477	An14g01068
evm.model.unitig_5.478	An14g01060
evm.model.unitig_5.479	An14g01050
evm.model.unitig_5.48	An14g06870
evm.model.unitig_5.480	An14g01030
evm.model.unitig_5.481	An14g01020
evm.model.unitig_5.482	An14g01010
evm.model.unitig_5.483	An14g01000
evm.model.unitig_5.484	An14g00990
evm.model.unitig_5.485	An14g00980
evm.model.unitig_5.486	An14g00970
evm.model.unitig_5.487	An14g00960
evm.model.unitig_5.488	An14g00950
evm.model.unitig_5.489	An14g00940
evm.model.unitig_5.49	An14g06860
evm.model.unitig_5.490	An14g00930
evm.model.unitig_5.491	An14g00920
evm.model.unitig_5.492	An14g00900
evm.model.unitig_5.493	An14g00890
evm.model.unitig_5.494	An14g00880
evm.model.unitig_5.495	An14g00870
evm.model.unitig_5.496	An14g00860
evm.model.unitig_5.497	An14g00850
evm.model.unitig_5.498	An14g00840
evm.model.unitig_5.499	An14g00830
evm.model.unitig_5.5	An14g07380
evm.model.unitig_5.50	An14g06850
evm.model.unitig_5.500	An14g00810
evm.model.unitig_5.501	An14g00800
evm.model.unitig_5.502	An14g00780
evm.model.unitig_5.503	An14g00770
evm.model.unitig_5.504	An14g00760
evm.model.unitig_5.505	An14g00750
evm.model.unitig_5.506	An14g00740
evm.model.unitig_5.507	An14g00710
evm.model.unitig_5.508	An14g00700
evm.model.unitig_5.509	An14g00690
evm.model.unitig_5.51	An14g06840
evm.model.unitig_5.510	An14g00680
evm.model.unitig_5.511	An14g00670
evm.model.unitig_5.512	An14g00660

Gene ID of H915-1	Gene ID of 513.88
evm.model.unitig_5.513	An14g00640
evm.model.unitig_5.514	An14g00630
evm.model.unitig_5.515	An14g00620
evm.model.unitig_5.516	An14g00610
evm.model.unitig_5.517	An14g00600
evm.model.unitig_5.518	An14g00590
evm.model.unitig_5.519	An14g00580
evm.model.unitig_5.52	An14g06830
evm.model.unitig_5.520	An14g00570
evm.model.unitig_5.521	-
evm.model.unitig_5.522	An14g00560
evm.model.unitig_5.524	An14g00550
evm.model.unitig_5.525	An14g00540
evm.model.unitig_5.526	An14g00530
evm.model.unitig_5.527	An14g00520
evm.model.unitig_5.528	An14g00510
evm.model.unitig_5.529	An14g00500
evm.model.unitig_5.53	An14g06820
evm.model.unitig_5.530	An14g00480
evm.model.unitig_5.532	An14g00460
evm.model.unitig_5.534	An14g00440
evm.model.unitig_5.535	An14g00430
evm.model.unitig_5.536	An14g00420
evm.model.unitig_5.537	An14g00410
evm.model.unitig_5.538	An14g00400
evm.model.unitig_5.539	An14g00390
evm.model.unitig_5.54	An14g06810
evm.model.unitig_5.540	An14g00380
evm.model.unitig_5.541	An14g00370
evm.model.unitig_5.542	An14g00360
evm.model.unitig_5.543	An14g00350
evm.model.unitig_5.544	An14g00340
evm.model.unitig_5.545	An14g00330
evm.model.unitig_5.546	An14g00320
evm.model.unitig_5.547	An14g00310
evm.model.unitig_5.548	An14g00300
evm.model.unitig_5.549	An14g00290
evm.model.unitig_5.55	An14g06800
evm.model.unitig_5.550	An14g00280
evm.model.unitig_5.551	An14g00270
evm.model.unitig_5.552	An14g00260
evm.model.unitig_5.553	An14g00250
evm.model.unitig_5.554	An14g00240

Gene ID of H915-1	Gene ID of 513.88
evm.model.unitig_5.555	An14g00230
evm.model.unitig_5.556	An14g00220
evm.model.unitig_5.557	An14g00200
evm.model.unitig_5.558	An14g00190
evm.model.unitig_5.559	An14g00180
evm.model.unitig_5.56	An14g06790
evm.model.unitig_5.560	An14g00170
evm.model.unitig_5.561	An14g00160
evm.model.unitig_5.562	An14g00150
evm.model.unitig_5.563	An14g00130
evm.model.unitig_5.564	An14g00130
evm.model.unitig_5.565	An14g00110
evm.model.unitig_5.566	An14g00100
evm.model.unitig_5.567	An14g00090
evm.model.unitig_5.568	An14g00080
evm.model.unitig_5.569	An14g00070
evm.model.unitig_5.57	An14g06780
evm.model.unitig_5.570	An14g00060
evm.model.unitig_5.571	An14g00040
evm.model.unitig_5.572	An14g00020
evm.model.unitig_5.573	An14g00010
evm.model.unitig_5.575	An03g06410
evm.model.unitig_5.579	An07g07340
evm.model.unitig_5.58	An14g06770
evm.model.unitig_5.580	An09g06870
evm.model.unitig_5.581	An09g06860
evm.model.unitig_5.582	An09g06850
evm.model.unitig_5.583	An09g06840
evm.model.unitig_5.584	An09g06830
evm.model.unitig_5.585	An09g06810
evm.model.unitig_5.586	An09g06800
evm.model.unitig_5.587	An09g06790
evm.model.unitig_5.588	An09g06780
evm.model.unitig_5.589	An09g06770
evm.model.unitig_5.59	An14g06760
evm.model.unitig_5.590	An09g06760
evm.model.unitig_5.591	An09g06750
evm.model.unitig_5.592	An09g06740
evm.model.unitig_5.593	An09g06730
evm.model.unitig_5.594	An09g06720
evm.model.unitig_5.595	An09g06710
evm.model.unitig_5.596	An09g06700
evm.model.unitig_5.597	An09g06680

Gene ID of H915-1	Gene ID of 513.88
evm.model.unitig_5.598	An09g06670
evm.model.unitig_5.599	An09g06650
evm.model.unitig_5.6	An14g07360
evm.model.unitig_5.60	An14g06750
evm.model.unitig_5.600	An09g06640
evm.model.unitig_5.601	An09g06630
evm.model.unitig_5.602	An09g06610
evm.model.unitig_5.603	An09g06590
evm.model.unitig_5.604	An09g06580
evm.model.unitig_5.605	An09g06570
evm.model.unitig_5.606	An09g06550
evm.model.unitig_5.607	An09g06540
evm.model.unitig_5.608	An09g06530
evm.model.unitig_5.609	An09g06520
evm.model.unitig_5.61	An14g06740
evm.model.unitig_5.610	An09g06510
evm.model.unitig_5.611	An09g06500
evm.model.unitig_5.612	An09g06490
evm.model.unitig_5.613	An09g06480
evm.model.unitig_5.614	An09g06460
evm.model.unitig_5.615	An09g06450
evm.model.unitig_5.616	An09g06440
evm.model.unitig_5.617	An09g06430
evm.model.unitig_5.618	An09g06420
evm.model.unitig_5.619	An09g06410
evm.model.unitig_5.62	An14g06730
evm.model.unitig_5.620	An09g06400
evm.model.unitig_5.621	An09g06390
evm.model.unitig_5.622	An09g06380
evm.model.unitig_5.623	An09g06370
evm.model.unitig_5.624	An09g06360
evm.model.unitig_5.625	An09g06350
evm.model.unitig_5.626	An09g06330
evm.model.unitig_5.627	An09g06330
evm.model.unitig_5.628	An09g06320
evm.model.unitig_5.629	An09g06310
evm.model.unitig_5.63	An14g06720
evm.model.unitig_5.630	An09g06290
evm.model.unitig_5.631	An09g06280
evm.model.unitig_5.632	An09g06270
evm.model.unitig_5.633	An09g06260
evm.model.unitig_5.634	An09g06250
evm.model.unitig_5.635	An09g06240

Gene ID of H915-1	Gene ID of 513.88
evm.model.unitig_5.636	An09g06220
evm.model.unitig_5.637	An09g06210
evm.model.unitig_5.638	An09g06180
evm.model.unitig_5.639	An09g06160
evm.model.unitig_5.64	An14g06700
evm.model.unitig_5.640	An09g06150
evm.model.unitig_5.641	An09g06140
evm.model.unitig_5.642	An09g06130
evm.model.unitig_5.643	An09g06120
evm.model.unitig_5.644	An09g06110
evm.model.unitig_5.645	An09g06100
evm.model.unitig_5.646	An03g04890
evm.model.unitig_5.647	An09g06070
evm.model.unitig_5.648	An09g06050
evm.model.unitig_5.649	An09g06040
evm.model.unitig_5.65	An14g06680
evm.model.unitig_5.650	An09g06030
evm.model.unitig_5.651	-
evm.model.unitig_5.652	An09g06020
evm.model.unitig_5.653	An09g06010
evm.model.unitig_5.654	An09g06000
evm.model.unitig_5.655	An09g05990
evm.model.unitig_5.656	An09g05980
evm.model.unitig_5.657	An09g05970
evm.model.unitig_5.658	An09g05950
evm.model.unitig_5.659	An09g05940
evm.model.unitig_5.66	An14g06670
evm.model.unitig_5.660	An09g05920
evm.model.unitig_5.661	An09g05900
evm.model.unitig_5.662	An09g05880
evm.model.unitig_5.663	An09g05870
evm.model.unitig_5.664	An09g05860
evm.model.unitig_5.665	An09g05850
evm.model.unitig_5.666	An09g05840
evm.model.unitig_5.667	An09g05830
evm.model.unitig_5.668	An09g05800
evm.model.unitig_5.669	An09g05780
evm.model.unitig_5.67	An14g06640
evm.model.unitig_5.670	An09g05770
evm.model.unitig_5.671	An09g05760
evm.model.unitig_5.672	An09g05730
evm.model.unitig_5.673	An09g05710
evm.model.unitig_5.674	An09g05700

Gene ID of H915-1	Gene ID of 513.88
evm.model.unitig_5.675	An09g05690
evm.model.unitig_5.676	An09g05680
evm.model.unitig_5.677	An09g05670
evm.model.unitig_5.678	An09g05660
evm.model.unitig_5.679	An09g05655
evm.model.unitig_5.68	An14g06630
evm.model.unitig_5.680	An09g05650
evm.model.unitig_5.681	An09g05640
evm.model.unitig_5.682	An09g05630
evm.model.unitig_5.683	An09g05610
evm.model.unitig_5.684	An09g05600
evm.model.unitig_5.685	An09g05590
evm.model.unitig_5.686	An09g05580
evm.model.unitig_5.687	An09g05550
evm.model.unitig_5.688	An09g05540
evm.model.unitig_5.689	An09g05530
evm.model.unitig_5.69	An14g06610
evm.model.unitig_5.690	An09g05490
evm.model.unitig_5.691	An09g05490
evm.model.unitig_5.692	An09g05480
evm.model.unitig_5.693	An09g05460
evm.model.unitig_5.694	An09g05420
evm.model.unitig_5.695	An09g05410
evm.model.unitig_5.696	An09g05400
evm.model.unitig_5.697	An09g05380
evm.model.unitig_5.698	An09g05370
evm.model.unitig_5.699	An09g05350
evm.model.unitig_5.7	An14g07350
evm.model.unitig_5.70	An14g06600
evm.model.unitig_5.700	An09g05340
evm.model.unitig_5.701	An09g05330
evm.model.unitig_5.702	An09g05320
evm.model.unitig_5.703	An09g05310
evm.model.unitig_5.704	An09g05300
evm.model.unitig_5.705	An09g05290
evm.model.unitig_5.706	An09g05280
evm.model.unitig_5.707	An09g05270
evm.model.unitig_5.708	An09g05260
evm.model.unitig_5.709	An09g05250
evm.model.unitig_5.71	An14g06590
evm.model.unitig_5.710	An09g05240
evm.model.unitig_5.711	An09g05230
evm.model.unitig_5.712	An09g05220

Gene ID of H915-1	Gene ID of 513.88
evm.model.unitig_5.713	An09g05200
evm.model.unitig_5.714	An09g05180
evm.model.unitig_5.715	An09g05170
evm.model.unitig_5.716	An09g05150
evm.model.unitig_5.717	An09g05140
evm.model.unitig_5.718	An09g05130
evm.model.unitig_5.719	An09g05120
evm.model.unitig_5.72	An14g06590
evm.model.unitig_5.720	An09g05110
evm.model.unitig_5.721	An09g05100
evm.model.unitig_5.722	An09g05080
evm.model.unitig_5.723	An09g05070
evm.model.unitig_5.724	An09g05060
evm.model.unitig_5.726	An09g05040
evm.model.unitig_5.727	An09g05010
evm.model.unitig_5.728	An09g05000
evm.model.unitig_5.729	An09g04990
evm.model.unitig_5.73	An14g06580
evm.model.unitig_5.730	An09g04980
evm.model.unitig_5.731	An09g04970
evm.model.unitig_5.732	An09g04950
evm.model.unitig_5.733	-
evm.model.unitig_5.734	An09g04910
evm.model.unitig_5.735	An09g04900
evm.model.unitig_5.736	An09g04890
evm.model.unitig_5.737	An09g04880
evm.model.unitig_5.738	An09g04870
evm.model.unitig_5.739	An09g04860
evm.model.unitig_5.74	An14g06560
evm.model.unitig_5.740	An09g04850
evm.model.unitig_5.741	An09g04840
evm.model.unitig_5.742	An09g04830
evm.model.unitig_5.743	An09g04820
evm.model.unitig_5.744	An09g04810
evm.model.unitig_5.745	An09g04790
evm.model.unitig_5.746	An09g04780
evm.model.unitig_5.747	An09g04730
evm.model.unitig_5.748	An09g04710
evm.model.unitig_5.749	An09g04690
evm.model.unitig_5.75	An14g06550
evm.model.unitig_5.750	An09g04680
evm.model.unitig_5.751	An09g04660
evm.model.unitig_5.752	An09g04650

Gene ID of H915-1	Gene ID of 513.88
evm.model.unitig_5.753	An09g04640
evm.model.unitig_5.754	An09g04620
evm.model.unitig_5.755	An09g04600
evm.model.unitig_5.756	An09g04590
evm.model.unitig_5.757	An09g04580
evm.model.unitig_5.759	An09g04540
evm.model.unitig_5.76	An14g06540
evm.model.unitig_5.760	An09g04530
evm.model.unitig_5.761	An09g04520
evm.model.unitig_5.762	An09g04510
evm.model.unitig_5.763	-
evm.model.unitig_5.764	An09g04470
evm.model.unitig_5.765	An09g04440
evm.model.unitig_5.767	An09g04390
evm.model.unitig_5.768	An09g04380
evm.model.unitig_5.769	An09g04370
evm.model.unitig_5.77	An14g06530
evm.model.unitig_5.770	An09g04360
evm.model.unitig_5.771	An09g04300
evm.model.unitig_5.772	An09g04280
evm.model.unitig_5.773	An09g04250
evm.model.unitig_5.774	An09g04240
evm.model.unitig_5.775	An09g04230
evm.model.unitig_5.776	An09g04200
evm.model.unitig_5.777	An09g04190
evm.model.unitig_5.778	An09g04180
evm.model.unitig_5.779	An09g04170
evm.model.unitig_5.78	An14g06530
evm.model.unitig_5.780	An09g04150
evm.model.unitig_5.781	An09g04130
evm.model.unitig_5.782	An09g04120
evm.model.unitig_5.783	An09g04110
evm.model.unitig_5.784	An09g04100
evm.model.unitig_5.785	An09g04090
evm.model.unitig_5.786	An09g04080
evm.model.unitig_5.787	An09g04070
evm.model.unitig_5.788	An09g04050
evm.model.unitig_5.789	An09g04010
evm.model.unitig_5.79	An14g06500
evm.model.unitig_5.790	An09g04000
evm.model.unitig_5.791	An09g03980
evm.model.unitig_5.792	-
evm.model.unitig_5.793	An09g03970

Gene ID of H915-1	Gene ID of 513.88
evm.model.unitig_5.794	An09g03960
evm.model.unitig_5.795	An09g03950
evm.model.unitig_5.796	An09g03940
evm.model.unitig_5.797	An09g03930
evm.model.unitig_5.798	An09g03920
evm.model.unitig_5.799	An11g05770
evm.model.unitig_5.8	An14g07330
evm.model.unitig_5.80	An14g06480
evm.model.unitig_5.800	An09g03900
evm.model.unitig_5.801	An09g03890
evm.model.unitig_5.802	An09g03880
evm.model.unitig_5.803	An09g03870
evm.model.unitig_5.804	An09g03860
evm.model.unitig_5.805	An09g03850
evm.model.unitig_5.806	An11g02830
evm.model.unitig_5.807	An09g03830
evm.model.unitig_5.808	An09g03820
evm.model.unitig_5.809	An09g03810
evm.model.unitig_5.81	An14g06470
evm.model.unitig_5.810	An09g03800
evm.model.unitig_5.811	An09g03790
evm.model.unitig_5.812	An09g03780
evm.model.unitig_5.813	An09g03770
evm.model.unitig_5.814	An09g03760
evm.model.unitig_5.815	An09g03750
evm.model.unitig_5.816	An09g03740
evm.model.unitig_5.817	An09g03730
evm.model.unitig_5.818	An09g03720
evm.model.unitig_5.819	An09g03710
evm.model.unitig_5.82	An14g06460
evm.model.unitig_5.820	An09g03700
evm.model.unitig_5.821	An09g03660
evm.model.unitig_5.822	An09g03640
evm.model.unitig_5.823	An09g03630
evm.model.unitig_5.824	An09g03620
evm.model.unitig_5.825	-
evm.model.unitig_5.826	An09g03600
evm.model.unitig_5.827	An09g03560
evm.model.unitig_5.828	An09g03530
evm.model.unitig_5.829	An09g03510
evm.model.unitig_5.83	An14g06450
evm.model.unitig_5.830	An11g05350
evm.model.unitig_5.831	An09g03500

Gene ID of H915-1	Gene ID of 513.88
evm.model.unitig_5.832	An09g03490
evm.model.unitig_5.833	An09g03470
evm.model.unitig_5.834	An09g03450
evm.model.unitig_5.835	An09g03440
evm.model.unitig_5.837	An09g03380
evm.model.unitig_5.838	An08g07270
evm.model.unitig_5.839	An09g03350
evm.model.unitig_5.84	An14g06440
evm.model.unitig_5.840	An09g03340
evm.model.unitig_5.841	An09g03330
evm.model.unitig_5.842	An09g03320
evm.model.unitig_5.843	An09g03300
evm.model.unitig_5.844	An09g03290
evm.model.unitig_5.845	An09g03270
evm.model.unitig_5.846	An09g03260
evm.model.unitig_5.847	An09g03250
evm.model.unitig_5.848	An09g03240
evm.model.unitig_5.849	An09g03230
evm.model.unitig_5.85	An14g06430
evm.model.unitig_5.850	An09g03210
evm.model.unitig_5.851	An09g03200
evm.model.unitig_5.852	An09g03170
evm.model.unitig_5.853	An09g03160
evm.model.unitig_5.854	An09g03140
evm.model.unitig_5.855	An09g03130
evm.model.unitig_5.856	An09g03120
evm.model.unitig_5.857	An09g03110
evm.model.unitig_5.858	An09g03100
evm.model.unitig_5.859	An09g03070
evm.model.unitig_5.86	An14g06360
evm.model.unitig_5.860	An09g03050
evm.model.unitig_5.861	An09g03050
evm.model.unitig_5.862	-
evm.model.unitig_5.863	An09g03040
evm.model.unitig_5.864	An09g03030
evm.model.unitig_5.865	An09g03020
evm.model.unitig_5.866	An09g03010
evm.model.unitig_5.867	An09g02990
evm.model.unitig_5.868	An09g02980
evm.model.unitig_5.869	An09g02930
evm.model.unitig_5.87	An14g06350
evm.model.unitig_5.870	An09g02920
evm.model.unitig_5.872	An09g02910

Gene ID of H915-1	Gene ID of 513.88
evm.model.unitig_5.873	-
evm.model.unitig_5.874	An09g02900
evm.model.unitig_5.875	An09g02890
evm.model.unitig_5.876	An09g02880
evm.model.unitig_5.877	An09g02870
evm.model.unitig_5.878	An09g02860
evm.model.unitig_5.879	An09g02840
evm.model.unitig_5.88	An14g06340
evm.model.unitig_5.880	An09g02830
evm.model.unitig_5.881	An09g02820
evm.model.unitig_5.882	An09g02810
evm.model.unitig_5.883	-
evm.model.unitig_5.884	An09g02800
evm.model.unitig_5.885	An09g02790
evm.model.unitig_5.886	An09g02780
evm.model.unitig_5.887	An09g02770
evm.model.unitig_5.888	An09g02740
evm.model.unitig_5.889	An09g02730
evm.model.unitig_5.89	An14g06330
evm.model.unitig_5.890	An09g02720
evm.model.unitig_5.891	An09g02710
evm.model.unitig_5.892	An09g02700
evm.model.unitig_5.893	An09g02660
evm.model.unitig_5.894	An09g02610
evm.model.unitig_5.895	An09g02580
evm.model.unitig_5.896	An09g02580
evm.model.unitig_5.897	An09g02560
evm.model.unitig_5.898	An09g02550
evm.model.unitig_5.899	An09g02540
evm.model.unitig_5.9	An14g07320
evm.model.unitig_5.90	An14g06320
evm.model.unitig_5.900	An09g02530
evm.model.unitig_5.901	An09g02520
evm.model.unitig_5.902	An09g02510
evm.model.unitig_5.903	An09g02490
evm.model.unitig_5.904	An09g02470
evm.model.unitig_5.905	An09g02460
evm.model.unitig_5.907	An09g02440
evm.model.unitig_5.908	An09g02430
evm.model.unitig_5.91	An14g06310
evm.model.unitig_5.910	An09g02410
evm.model.unitig_5.911	An09g02390
evm.model.unitig_5.912	An09g02380

Gene ID of H915-1	Gene ID of 513.88
evm.model.unitig_5.913	An06g02150
evm.model.unitig_5.914	An06g00940
evm.model.unitig_5.915	An11g07050
evm.model.unitig_5.916	An06g00940
evm.model.unitig_5.917	An09g02370
evm.model.unitig_5.918	An09g02340
evm.model.unitig_5.919	An09g02330
evm.model.unitig_5.92	An14g06300
evm.model.unitig_5.920	An09g02320
evm.model.unitig_5.922	An09g02290
evm.model.unitig_5.923	An09g02280
evm.model.unitig_5.924	An09g02270
evm.model.unitig_5.925	An09g02260
evm.model.unitig_5.926	An09g02250
evm.model.unitig_5.927	An09g02240
evm.model.unitig_5.928	An09g02230
evm.model.unitig_5.929	An09g02220
evm.model.unitig_5.93	An14g06290
evm.model.unitig_5.930	An09g02210
evm.model.unitig_5.931	An09g02180
evm.model.unitig_5.932	An09g02160
evm.model.unitig_5.933	An09g02150
evm.model.unitig_5.934	An09g02120
evm.model.unitig_5.935	An01g13720
evm.model.unitig_5.936	An18g00750
evm.model.unitig_5.937	An09g02080
evm.model.unitig_5.938	An09g02060
evm.model.unitig_5.939	An09g02050
evm.model.unitig_5.94	An14g06280
evm.model.unitig_5.940	An09g02040
evm.model.unitig_5.941	An09g02020
evm.model.unitig_5.942	An09g02010
evm.model.unitig_5.943	An09g02000
evm.model.unitig_5.944	An09g01990
evm.model.unitig_5.945	An09g01970
evm.model.unitig_5.946	An09g01950
evm.model.unitig_5.947	An09g01930
evm.model.unitig_5.948	An09g01910
evm.model.unitig_5.949	An09g01870
evm.model.unitig_5.95	An14g06270
evm.model.unitig_5.950	An09g01860
evm.model.unitig_5.951	An09g01840
evm.model.unitig_5.952	An09g01820

Gene ID of H915-1	Gene ID of 513.88
evm.model.unitig_5.953	An09g01810
evm.model.unitig_5.954	An09g01800
evm.model.unitig_5.955	An09g01790
evm.model.unitig_5.956	An09g01740
evm.model.unitig_5.957	An09g01710
evm.model.unitig_5.958	An09g01700
evm.model.unitig_5.959	An09g01690
evm.model.unitig_5.96	An14g06260
evm.model.unitig_5.960	An09g01680
evm.model.unitig_5.962	An09g01650
evm.model.unitig_5.963	An09g01630
evm.model.unitig_5.964	An09g01620
evm.model.unitig_5.965	An09g01560
evm.model.unitig_5.966	An09g01550
evm.model.unitig_5.967	An09g01540
evm.model.unitig_5.968	An09g01520
evm.model.unitig_5.969	An09g01510
evm.model.unitig_5.97	An14g06250
evm.model.unitig_5.970	An09g01510
evm.model.unitig_5.973	An01g01840
evm.model.unitig_5.974	An09g01440
evm.model.unitig_5.975	An09g01430
evm.model.unitig_5.976	An16g05710
evm.model.unitig_5.977	An09g01380
evm.model.unitig_5.978	An09g01370
evm.model.unitig_5.979	An09g01330
evm.model.unitig_5.98	An14g06230
evm.model.unitig_5.980	An09g01320
evm.model.unitig_5.981	An09g01310
evm.model.unitig_5.982	An09g01300
evm.model.unitig_5.983	An09g01290
evm.model.unitig_5.984	An09g01270
evm.model.unitig_5.985	An09g01260
evm.model.unitig_5.987	An09g01240
evm.model.unitig_5.988	An09g01220
evm.model.unitig_5.989	An09g01210
evm.model.unitig_5.99	An14g06220
evm.model.unitig_5.990	An09g01200
evm.model.unitig_5.991	An09g01190
evm.model.unitig_5.992	An09g01180
evm.model.unitig_5.993	An09g01170
evm.model.unitig_5.994	An09g01160
evm.model.unitig_5.995	An09g01150

Gene ID of H915-1	Gene ID of 513.88
evm.model.unitig_5.996	An09g01140
evm.model.unitig_5.997	An09g01130
evm.model.unitig_5.998	An09g01125
evm.model.unitig_5.999	An09g01120
evm.model.unitig_50.1	-
evm.model.unitig_52.1	-
evm.model.unitig_58.2	-
evm.model.unitig_6.1	An10g00010
evm.model.unitig_6.10	An10g00150
evm.model.unitig_6.100	An17g00090
evm.model.unitig_6.101	An17g00120
evm.model.unitig_6.102	An14g01910
evm.model.unitig_6.103	An11g06460
evm.model.unitig_6.104	An17g00170
evm.model.unitig_6.105	An17g00180
evm.model.unitig_6.106	An17g00200
evm.model.unitig_6.107	An17g00210
evm.model.unitig_6.108	An17g00230
evm.model.unitig_6.109	An17g00240
evm.model.unitig_6.11	An10g00150
evm.model.unitig_6.110	An17g00250
evm.model.unitig_6.111	An17g00260
evm.model.unitig_6.112	An17g00270
evm.model.unitig_6.113	An17g00280
evm.model.unitig_6.114	An17g00290
evm.model.unitig_6.115	An17g00300
evm.model.unitig_6.116	An17g00310
evm.model.unitig_6.117	An17g00320
evm.model.unitig_6.118	An17g00330
evm.model.unitig_6.119	An17g00340
evm.model.unitig_6.12	An10g00160
evm.model.unitig_6.120	An17g00370
evm.model.unitig_6.121	An17g00380
evm.model.unitig_6.122	An17g00390
evm.model.unitig_6.123	An17g00390
evm.model.unitig_6.124	An17g00400
evm.model.unitig_6.125	An11g02830
evm.model.unitig_6.126	An17g00420
evm.model.unitig_6.127	An17g00430
evm.model.unitig_6.128	An17g00440
evm.model.unitig_6.129	An17g00450
evm.model.unitig_6.13	An10g00170
evm.model.unitig_6.130	An17g00470

Gene ID of H915-1	Gene ID of 513.88
evm.model.unitig_6.132	An17g00520
evm.model.unitig_6.133	An17g00530
evm.model.unitig_6.134	An17g00550
evm.model.unitig_6.135	An17g00560
evm.model.unitig_6.136	An17g00570
evm.model.unitig_6.137	An17g00580
evm.model.unitig_6.138	An17g00590
evm.model.unitig_6.14	An10g00180
evm.model.unitig_6.140	An17g00600
evm.model.unitig_6.141	An17g00620
evm.model.unitig_6.142	An17g00630
evm.model.unitig_6.143	An17g00640
evm.model.unitig_6.144	An17g00650
evm.model.unitig_6.145	An17g00660
evm.model.unitig_6.146	An17g00670
evm.model.unitig_6.147	An17g00680
evm.model.unitig_6.148	An17g00690
evm.model.unitig_6.149	An17g00700
evm.model.unitig_6.15	An10g00190
evm.model.unitig_6.150	An17g00710
evm.model.unitig_6.151	An17g00720
evm.model.unitig_6.152	An17g00730
evm.model.unitig_6.153	An17g00750
evm.model.unitig_6.154	An17g00760
evm.model.unitig_6.155	An17g00770
evm.model.unitig_6.156	An17g00780
evm.model.unitig_6.157	An17g00790
evm.model.unitig_6.158	An17g00800
evm.model.unitig_6.159	An17g00810
evm.model.unitig_6.16	An10g00210
evm.model.unitig_6.160	An17g00820
evm.model.unitig_6.161	An17g00830
evm.model.unitig_6.162	An17g00840
evm.model.unitig_6.163	An17g00850
evm.model.unitig_6.164	An17g00860
evm.model.unitig_6.165	An17g00870
evm.model.unitig_6.166	An17g00890
evm.model.unitig_6.167	An17g00910
evm.model.unitig_6.168	An17g00930
evm.model.unitig_6.169	An17g00940
evm.model.unitig_6.17	An10g00220
evm.model.unitig_6.170	An17g00970
evm.model.unitig_6.171	An17g01000

Gene ID of H915-1	Gene ID of 513.88
evm.model.unitig_6.172	An17g01040
evm.model.unitig_6.173	An17g01050
evm.model.unitig_6.174	An17g01060
evm.model.unitig_6.175	An17g01070
evm.model.unitig_6.176	An17g01080
evm.model.unitig_6.177	An17g01100
evm.model.unitig_6.178	An17g01120
evm.model.unitig_6.179	An17g01140
evm.model.unitig_6.18	An10g00230
evm.model.unitig_6.180	An17g01160
evm.model.unitig_6.181	An17g01170
evm.model.unitig_6.183	An17g01250
evm.model.unitig_6.184	An17g01260
evm.model.unitig_6.185	An17g01270
evm.model.unitig_6.186	An17g01280
evm.model.unitig_6.187	An17g01300
evm.model.unitig_6.188	An17g01310
evm.model.unitig_6.189	An17g01320
evm.model.unitig_6.19	An10g00240
evm.model.unitig_6.190	An17g01330
evm.model.unitig_6.191	An17g01340
evm.model.unitig_6.192	An17g01350
evm.model.unitig_6.193	An17g01360
evm.model.unitig_6.194	An17g01370
evm.model.unitig_6.195	An17g01390
evm.model.unitig_6.196	An17g01410
evm.model.unitig_6.197	An17g01420
evm.model.unitig_6.198	An17g01430
evm.model.unitig_6.199	An17g01440
evm.model.unitig_6.2	An15g01800
evm.model.unitig_6.20	An10g00250
evm.model.unitig_6.200	An17g01450
evm.model.unitig_6.201	An17g01470
evm.model.unitig_6.202	An17g01480
evm.model.unitig_6.203	An17g01490
evm.model.unitig_6.204	An17g01530
evm.model.unitig_6.205	An17g01540
evm.model.unitig_6.206	An17g01550
evm.model.unitig_6.207	An17g01560
evm.model.unitig_6.208	An17g01580
evm.model.unitig_6.209	An17g01630
evm.model.unitig_6.21	An10g00260
evm.model.unitig_6.210	An17g01640

Gene ID of H915-1	Gene ID of 513.88
evm.model.unitig_6.211	An17g01660
evm.model.unitig_6.212	An17g01670
evm.model.unitig_6.213	An17g01710
evm.model.unitig_6.214	An17g01720
evm.model.unitig_6.215	An17g01730
evm.model.unitig_6.216	An17g01740
evm.model.unitig_6.217	An17g01750
evm.model.unitig_6.218	An17g01770
evm.model.unitig_6.22	An10g00280
evm.model.unitig_6.221	An01g02880
evm.model.unitig_6.222	An17g01795
evm.model.unitig_6.223	An17g01805
evm.model.unitig_6.224	An17g01815
evm.model.unitig_6.225	An17g01825
evm.model.unitig_6.226	An17g01865
evm.model.unitig_6.227	An17g01875
evm.model.unitig_6.228	An17g01925
evm.model.unitig_6.229	An17g01945
evm.model.unitig_6.23	An10g00290
evm.model.unitig_6.230	An17g01965
evm.model.unitig_6.231	An17g01977
evm.model.unitig_6.232	-
evm.model.unitig_6.233	An17g02010
evm.model.unitig_6.234	An17g02020
evm.model.unitig_6.235	An17g02030
evm.model.unitig_6.236	An17g02040
evm.model.unitig_6.237	An17g02060
evm.model.unitig_6.238	An17g02070
evm.model.unitig_6.239	An17g02075
evm.model.unitig_6.24	An10g00300
evm.model.unitig_6.240	An17g02080
evm.model.unitig_6.241	An17g02090
evm.model.unitig_6.242	An17g02100
evm.model.unitig_6.243	An17g02110
evm.model.unitig_6.244	An17g02120
evm.model.unitig_6.245	An17g02130
evm.model.unitig_6.246	An17g02140
evm.model.unitig_6.247	An17g02150
evm.model.unitig_6.248	An17g02160
evm.model.unitig_6.249	An17g02170
evm.model.unitig_6.25	An10g00310
evm.model.unitig_6.250	An17g02180
evm.model.unitig_6.251	An17g02190

Gene ID of H915-1	Gene ID of 513.88
evm.model.unitig_6.252	An17g02210
evm.model.unitig_6.253	An17g02220
evm.model.unitig_6.254	An17g02230
evm.model.unitig_6.255	An17g02240
evm.model.unitig_6.256	An17g02250
evm.model.unitig_6.257	An17g02270
evm.model.unitig_6.258	An17g02280
evm.model.unitig_6.259	-
evm.model.unitig_6.26	An10g00320
evm.model.unitig_6.260	An17g02287
evm.model.unitig_6.261	An17g02290
evm.model.unitig_6.262	An17g02300
evm.model.unitig_6.263	An17g02310
evm.model.unitig_6.264	An17g02330
evm.model.unitig_6.265	An17g02340
evm.model.unitig_6.266	An17g02350
evm.model.unitig_6.267	An17g02370
evm.model.unitig_6.268	An17g02390
evm.model.unitig_6.269	An16g09320
evm.model.unitig_6.27	An10g00330
evm.model.unitig_6.270	An16g09300
evm.model.unitig_6.271	An16g09280
evm.model.unitig_6.272	An16g09260
evm.model.unitig_6.273	An16g09250
evm.model.unitig_6.274	An16g09230
evm.model.unitig_6.275	An16g09210
evm.model.unitig_6.276	An16g09200
evm.model.unitig_6.277	An16g09190
evm.model.unitig_6.278	An16g09180
evm.model.unitig_6.279	-
evm.model.unitig_6.28	An10g00350
evm.model.unitig_6.280	An16g09160
evm.model.unitig_6.281	An16g09150
evm.model.unitig_6.282	An16g09140
evm.model.unitig_6.283	An16g09130
evm.model.unitig_6.284	An16g09090
evm.model.unitig_6.285	An16g09070
evm.model.unitig_6.286	An16g09060
evm.model.unitig_6.287	An16g09050
evm.model.unitig_6.288	An16g09040
evm.model.unitig_6.289	An16g09020
evm.model.unitig_6.29	An10g00360
evm.model.unitig_6.290	An16g09010

Gene ID of H915-1	Gene ID of 513.88
evm.model.unitig_6.291	An16g08980
evm.model.unitig_6.292	An16g08950
evm.model.unitig_6.293	An16g08940
evm.model.unitig_6.294	-
evm.model.unitig_6.295	An16g08920
evm.model.unitig_6.296	An16g08910
evm.model.unitig_6.297	An16g08890
evm.model.unitig_6.298	An16g08880
evm.model.unitig_6.299	An16g08870
evm.model.unitig_6.3	An10g00050
evm.model.unitig_6.30	An10g00370
evm.model.unitig_6.300	An16g08860
evm.model.unitig_6.301	-
evm.model.unitig_6.302	An16g08850
evm.model.unitig_6.303	An16g08840
evm.model.unitig_6.304	An16g08830
evm.model.unitig_6.305	An16g08820
evm.model.unitig_6.306	An16g08810
evm.model.unitig_6.307	An16g08800
evm.model.unitig_6.308	An16g08780
evm.model.unitig_6.309	An16g08760
evm.model.unitig_6.31	An10g00390
evm.model.unitig_6.310	An16g08740
evm.model.unitig_6.311	An16g08730
evm.model.unitig_6.312	An16g08720
evm.model.unitig_6.313	An16g08710
evm.model.unitig_6.314	An16g08700
evm.model.unitig_6.315	An18g04070
evm.model.unitig_6.316	An02g03740
evm.model.unitig_6.317	An16g08680
evm.model.unitig_6.318	An16g08670
evm.model.unitig_6.319	An16g08650
evm.model.unitig_6.32	An10g00400
evm.model.unitig_6.320	An16g08640
evm.model.unitig_6.321	An16g08630
evm.model.unitig_6.322	An16g08620
evm.model.unitig_6.323	An16g08610
evm.model.unitig_6.324	An16g08610
evm.model.unitig_6.325	An16g08590
evm.model.unitig_6.326	An16g08570
evm.model.unitig_6.327	An16g08560
evm.model.unitig_6.328	An16g08540
evm.model.unitig_6.329	An16g08530

Gene ID of H915-1	Gene ID of 513.88
evm.model.unitig_6.33	An10g00410
evm.model.unitig_6.330	An16g08520
evm.model.unitig_6.331	An16g08510
evm.model.unitig_6.332	An16g08500
evm.model.unitig_6.333	An16g08490
evm.model.unitig_6.334	An16g08480
evm.model.unitig_6.335	An16g08470
evm.model.unitig_6.336	An16g08460
evm.model.unitig_6.337	An16g08450
evm.model.unitig_6.338	An16g08440
evm.model.unitig_6.339	An16g08420
evm.model.unitig_6.340	An16g08410
evm.model.unitig_6.341	An16g08390
evm.model.unitig_6.342	An16g08380
evm.model.unitig_6.343	An16g08360
evm.model.unitig_6.344	An16g08330
evm.model.unitig_6.346	An16g08220
evm.model.unitig_6.347	An16g08210
evm.model.unitig_6.348	An16g08200
evm.model.unitig_6.349	An16g08170
evm.model.unitig_6.35	-
evm.model.unitig_6.350	An16g08150
evm.model.unitig_6.351	An16g08140
evm.model.unitig_6.352	An16g08130
evm.model.unitig_6.353	An16g08120
evm.model.unitig_6.354	An16g08110
evm.model.unitig_6.355	An16g08100
evm.model.unitig_6.356	An16g08090
evm.model.unitig_6.357	An16g08080
evm.model.unitig_6.358	An16g08070
evm.model.unitig_6.359	An16g08060
evm.model.unitig_6.36	An10g00450
evm.model.unitig_6.360	An11g01200
evm.model.unitig_6.361	An16g08040
evm.model.unitig_6.362	An16g08020
evm.model.unitig_6.363	An16g08010
evm.model.unitig_6.364	An16g08000
evm.model.unitig_6.365	An16g07990
evm.model.unitig_6.366	An16g07980
evm.model.unitig_6.367	An16g07970
evm.model.unitig_6.368	An16g07960
evm.model.unitig_6.369	An16g07940
evm.model.unitig_6.37	An10g00450

Gene ID of H915-1	Gene ID of 513.88
evm.model.unitig_6.371	An16g07920
evm.model.unitig_6.372	An16g07910
evm.model.unitig_6.373	An16g07900
evm.model.unitig_6.374	An16g07890
evm.model.unitig_6.375	An16g07870
evm.model.unitig_6.376	An16g07860
evm.model.unitig_6.377	An16g07830
evm.model.unitig_6.378	An16g07810
evm.model.unitig_6.379	An16g07800
evm.model.unitig_6.38	An10g00460
evm.model.unitig_6.380	An16g07795
evm.model.unitig_6.381	An16g07720
evm.model.unitig_6.382	An16g07710
evm.model.unitig_6.383	An16g07700
evm.model.unitig_6.384	An16g07690
evm.model.unitig_6.385	An16g07680
evm.model.unitig_6.386	An16g07660
evm.model.unitig_6.387	An16g07650
evm.model.unitig_6.388	An16g07640
evm.model.unitig_6.389	An16g07630
evm.model.unitig_6.39	An10g00470
evm.model.unitig_6.390	An16g07620
evm.model.unitig_6.391	An16g07610
evm.model.unitig_6.392	An16g07580
evm.model.unitig_6.394	An16g07540
evm.model.unitig_6.395	An16g07530
evm.model.unitig_6.396	An16g07520
evm.model.unitig_6.397	An16g07510
evm.model.unitig_6.398	An16g07500
evm.model.unitig_6.399	An16g07490
evm.model.unitig_6.4	An10g00050
evm.model.unitig_6.40	An10g00490
evm.model.unitig_6.400	An16g07480
evm.model.unitig_6.401	An16g07460
evm.model.unitig_6.402	An16g07450
evm.model.unitig_6.403	An16g07440
evm.model.unitig_6.404	An16g07430
evm.model.unitig_6.405	An16g07420
evm.model.unitig_6.406	An16g07410
evm.model.unitig_6.407	An16g07400
evm.model.unitig_6.408	An16g07390
evm.model.unitig_6.409	An16g07380
evm.model.unitig_6.41	An10g00500

Gene ID of H915-1	Gene ID of 513.88
evm.model.unitig_6.410	An16g07370
evm.model.unitig_6.411	An16g07360
evm.model.unitig_6.412	An16g07350
evm.model.unitig_6.414	An16g07340
evm.model.unitig_6.415	An16g07330
evm.model.unitig_6.416	An16g07300
evm.model.unitig_6.417	An16g07290
evm.model.unitig_6.418	An16g07280
evm.model.unitig_6.419	An16g07260
evm.model.unitig_6.42	An10g00510
evm.model.unitig_6.420	An16g07230
evm.model.unitig_6.421	An16g07220
evm.model.unitig_6.422	An16g07210
evm.model.unitig_6.423	An16g07200
evm.model.unitig_6.424	An16g07180
evm.model.unitig_6.425	An16g07150
evm.model.unitig_6.426	An16g07140
evm.model.unitig_6.427	An16g07130
evm.model.unitig_6.428	An16g07120
evm.model.unitig_6.429	An16g07110
evm.model.unitig_6.43	An09g05780
evm.model.unitig_6.430	An16g07090
evm.model.unitig_6.431	An16g07040
evm.model.unitig_6.432	An16g07030
evm.model.unitig_6.433	An04g08040
evm.model.unitig_6.434	An16g06990
evm.model.unitig_6.435	An16g06950
evm.model.unitig_6.436	An16g06940
evm.model.unitig_6.438	An16g06890
evm.model.unitig_6.439	An16g06860
evm.model.unitig_6.44	An10g00530
evm.model.unitig_6.440	An16g06850
evm.model.unitig_6.442	An16g06820
evm.model.unitig_6.443	An16g06810
evm.model.unitig_6.444	An16g06800
evm.model.unitig_6.445	An16g06790
evm.model.unitig_6.446	An16g06780
evm.model.unitig_6.447	An16g06770
evm.model.unitig_6.448	An16g06760
evm.model.unitig_6.449	An16g06750
evm.model.unitig_6.45	An10g00540
evm.model.unitig_6.450	An16g06740
evm.model.unitig_6.451	An16g06720

Gene ID of H915-1	Gene ID of 513.88
evm.model.unitig_6.452	An16g06710
evm.model.unitig_6.453	An16g06690
evm.model.unitig_6.454	An16g06680
evm.model.unitig_6.456	An18g02280
evm.model.unitig_6.457	An16g06630
evm.model.unitig_6.458	An16g06620
evm.model.unitig_6.459	An16g06610
evm.model.unitig_6.46	An10g00560
evm.model.unitig_6.460	An16g06600
evm.model.unitig_6.461	An16g06590
evm.model.unitig_6.462	An16g06580
evm.model.unitig_6.464	An16g06560
evm.model.unitig_6.465	An16g06550
evm.model.unitig_6.466	An16g06540
evm.model.unitig_6.467	An16g06530
evm.model.unitig_6.468	An16g06520
evm.model.unitig_6.469	An16g06510
evm.model.unitig_6.47	An10g00570
evm.model.unitig_6.470	An16g06500
evm.model.unitig_6.471	An16g06490
evm.model.unitig_6.472	An16g06480
evm.model.unitig_6.473	An16g06470
evm.model.unitig_6.474	An16g06460
evm.model.unitig_6.475	An16g06440
evm.model.unitig_6.476	An16g06440
evm.model.unitig_6.477	An16g06420
evm.model.unitig_6.478	An16g06410
evm.model.unitig_6.479	An16g06400
evm.model.unitig_6.48	An10g00600
evm.model.unitig_6.480	An16g06390
evm.model.unitig_6.481	An01g14430
evm.model.unitig_6.482	An16g06370
evm.model.unitig_6.483	An16g06360
evm.model.unitig_6.484	An16g06350
evm.model.unitig_6.486	An16g06300
evm.model.unitig_6.487	An16g06270
evm.model.unitig_6.488	An16g06260
evm.model.unitig_6.489	An16g06240
evm.model.unitig_6.49	An10g00600
evm.model.unitig_6.490	An16g06220
evm.model.unitig_6.491	An16g06210
evm.model.unitig_6.492	An16g06200
evm.model.unitig_6.493	An16g06190

Gene ID of H915-1	Gene ID of 513.88
evm.model.unitig_6.494	An16g06170
evm.model.unitig_6.495	An08g11610
evm.model.unitig_6.496	An16g06140
evm.model.unitig_6.497	An16g06120
evm.model.unitig_6.498	An16g06100
evm.model.unitig_6.499	An16g06090
evm.model.unitig_6.5	An10g00070
evm.model.unitig_6.50	An10g00600
evm.model.unitig_6.500	An16g06080
evm.model.unitig_6.503	An16g06010
evm.model.unitig_6.504	An16g05970
evm.model.unitig_6.505	An16g05960
evm.model.unitig_6.506	An16g05940
evm.model.unitig_6.507	An16g05930
evm.model.unitig_6.508	An16g05920
evm.model.unitig_6.509	An16g05910
evm.model.unitig_6.51	An10g00620
evm.model.unitig_6.510	An16g05880
evm.model.unitig_6.511	An16g05840
evm.model.unitig_6.512	An08g08030
evm.model.unitig_6.513	An16g05810
evm.model.unitig_6.514	An16g05790
evm.model.unitig_6.515	An11g03690
evm.model.unitig_6.516	An16g05770
evm.model.unitig_6.517	An16g05760
evm.model.unitig_6.518	An16g05750
evm.model.unitig_6.519	An16g05740
evm.model.unitig_6.52	An10g00630
evm.model.unitig_6.520	An16g05730
evm.model.unitig_6.521	An16g05710
evm.model.unitig_6.522	An16g05690
evm.model.unitig_6.523	An16g05660
evm.model.unitig_6.524	-
evm.model.unitig_6.525	-
evm.model.unitig_6.526	An16g05580
evm.model.unitig_6.527	An16g05570
evm.model.unitig_6.528	An16g05560
evm.model.unitig_6.529	An16g05550
evm.model.unitig_6.53	An10g00650
evm.model.unitig_6.530	An16g05520
evm.model.unitig_6.531	An16g05510
evm.model.unitig_6.532	An16g05500
evm.model.unitig_6.533	An16g05460

Gene ID of H915-1	Gene ID of 513.88
evm.model.unitig_6.534	An16g05460
evm.model.unitig_6.535	An16g05450
evm.model.unitig_6.536	An16g05440
evm.model.unitig_6.537	An16g05430
evm.model.unitig_6.538	An16g05420
evm.model.unitig_6.539	An16g05400
evm.model.unitig_6.54	An10g00690
evm.model.unitig_6.540	An16g05390
evm.model.unitig_6.541	An16g05380
evm.model.unitig_6.542	An16g05370
evm.model.unitig_6.543	An01g01030
evm.model.unitig_6.544	An16g05350
evm.model.unitig_6.545	An16g05340
evm.model.unitig_6.546	An16g05330
evm.model.unitig_6.547	An16g05320
evm.model.unitig_6.548	An16g05310
evm.model.unitig_6.549	An16g05290
evm.model.unitig_6.55	An10g00710
evm.model.unitig_6.550	An16g05280
evm.model.unitig_6.551	An16g05270
evm.model.unitig_6.552	An16g05190
evm.model.unitig_6.554	An16g05150
evm.model.unitig_6.555	An16g05130
evm.model.unitig_6.556	An16g05120
evm.model.unitig_6.557	An16g05090
evm.model.unitig_6.558	An16g05070
evm.model.unitig_6.559	An16g05060
evm.model.unitig_6.56	-
evm.model.unitig_6.560	An16g05050
evm.model.unitig_6.561	An16g05030
evm.model.unitig_6.562	An16g05020
evm.model.unitig_6.563	An16g04970
evm.model.unitig_6.564	An16g04950
evm.model.unitig_6.565	An16g04940
evm.model.unitig_6.566	An16g04930
evm.model.unitig_6.567	An16g04920
evm.model.unitig_6.568	An16g04910
evm.model.unitig_6.569	An16g04900
evm.model.unitig_6.57	-
evm.model.unitig_6.570	An16g04890
evm.model.unitig_6.571	An16g04880
evm.model.unitig_6.572	An16g04870
evm.model.unitig_6.573	An16g04840

Gene ID of H915-1	Gene ID of 513.88
evm.model.unitig_6.574	An16g04830
evm.model.unitig_6.575	An16g04820
evm.model.unitig_6.576	An16g04790
evm.model.unitig_6.577	An16g04780
evm.model.unitig_6.578	An16g04770
evm.model.unitig_6.579	An16g04760
evm.model.unitig_6.58	An10g00800
evm.model.unitig_6.580	An16g04750
evm.model.unitig_6.581	An16g04730
evm.model.unitig_6.582	An16g04720
evm.model.unitig_6.583	An16g04710
evm.model.unitig_6.584	An16g04700
evm.model.unitig_6.585	An16g04690
evm.model.unitig_6.586	An16g04680
evm.model.unitig_6.587	An16g04670
evm.model.unitig_6.588	An16g04660
evm.model.unitig_6.589	An16g04650
evm.model.unitig_6.59	An10g00820
evm.model.unitig_6.590	An16g04640
evm.model.unitig_6.591	An16g04630
evm.model.unitig_6.592	An16g04600
evm.model.unitig_6.593	An16g04580
evm.model.unitig_6.594	An16g04560
evm.model.unitig_6.595	An16g04550
evm.model.unitig_6.596	An16g04530
evm.model.unitig_6.597	An16g04530
evm.model.unitig_6.598	An16g04520
evm.model.unitig_6.599	An16g04500
evm.model.unitig_6.6	An10g00070
evm.model.unitig_6.60	An10g00830
evm.model.unitig_6.600	An16g04460
evm.model.unitig_6.601	An16g04440
evm.model.unitig_6.602	An16g04430
evm.model.unitig_6.603	An16g04420
evm.model.unitig_6.604	An16g04410
evm.model.unitig_6.605	An16g04400
evm.model.unitig_6.606	An16g04390
evm.model.unitig_6.607	An16g04370
evm.model.unitig_6.608	An16g04350
evm.model.unitig_6.609	An16g04340
evm.model.unitig_6.61	An10g00840
evm.model.unitig_6.610	An16g04330
evm.model.unitig_6.611	An16g04320

Gene ID of H915-1	Gene ID of 513.88
evm.model.unitig_6.612	An16g04310
evm.model.unitig_6.613	An16g04270
evm.model.unitig_6.614	An16g04260
evm.model.unitig_6.615	An16g04250
evm.model.unitig_6.616	An16g04230
evm.model.unitig_6.617	An16g04220
evm.model.unitig_6.618	An16g04210
evm.model.unitig_6.619	An16g04200
evm.model.unitig_6.62	An10g00850
evm.model.unitig_6.620	-
evm.model.unitig_6.621	An16g04180
evm.model.unitig_6.622	An16g04170
evm.model.unitig_6.623	An16g04160
evm.model.unitig_6.624	An16g04150
evm.model.unitig_6.625	An16g04140
evm.model.unitig_6.626	An16g04130
evm.model.unitig_6.627	An16g04120
evm.model.unitig_6.628	-
evm.model.unitig_6.629	An16g04110
evm.model.unitig_6.63	An10g00860
evm.model.unitig_6.630	An16g04100
evm.model.unitig_6.631	An16g04080
evm.model.unitig_6.632	An16g04060
evm.model.unitig_6.633	An16g04050
evm.model.unitig_6.634	An14g02500
evm.model.unitig_6.635	An16g04030
evm.model.unitig_6.636	An16g04020
evm.model.unitig_6.637	An16g04010
evm.model.unitig_6.638	An16g04000
evm.model.unitig_6.639	An16g03980
evm.model.unitig_6.64	An10g00880
evm.model.unitig_6.640	An16g03960
evm.model.unitig_6.641	An16g03950
evm.model.unitig_6.642	An16g03940
evm.model.unitig_6.644	An16g03910
evm.model.unitig_6.645	An16g03890
evm.model.unitig_6.646	An16g03880
evm.model.unitig_6.647	An16g03860
evm.model.unitig_6.648	An16g03850
evm.model.unitig_6.649	An16g03840
evm.model.unitig_6.65	An10g00900
evm.model.unitig_6.650	An16g03830
evm.model.unitig_6.651	An16g03820

Gene ID of H915-1	Gene ID of 513.88
evm.model.unitig_6.652	An16g03810
evm.model.unitig_6.653	An16g03800
evm.model.unitig_6.654	An16g03790
evm.model.unitig_6.655	An16g03760
evm.model.unitig_6.656	An16g03750
evm.model.unitig_6.657	An16g03740
evm.model.unitig_6.658	An16g03720
evm.model.unitig_6.659	An16g03710
evm.model.unitig_6.66	An10g00910
evm.model.unitig_6.660	An16g03700
evm.model.unitig_6.661	An16g03690
evm.model.unitig_6.662	An16g03650
evm.model.unitig_6.663	An16g03640
evm.model.unitig_6.664	An16g03630
evm.model.unitig_6.665	An16g03620
evm.model.unitig_6.666	An16g03610
evm.model.unitig_6.667	An16g03600
evm.model.unitig_6.668	An16g03590
evm.model.unitig_6.669	An16g03580
evm.model.unitig_6.67	An10g00950
evm.model.unitig_6.672	An16g03550
evm.model.unitig_6.673	An16g03540
evm.model.unitig_6.674	An16g03530
evm.model.unitig_6.675	An16g03520
evm.model.unitig_6.676	An16g03510
evm.model.unitig_6.677	An16g03500
evm.model.unitig_6.678	An16g03490
evm.model.unitig_6.679	An16g03450
evm.model.unitig_6.68	An08g04620
evm.model.unitig_6.680	An16g03430
evm.model.unitig_6.681	An16g03420
evm.model.unitig_6.682	An16g03410
evm.model.unitig_6.683	An16g03400
evm.model.unitig_6.684	An16g03390
evm.model.unitig_6.685	An16g03380
evm.model.unitig_6.686	An16g03370
evm.model.unitig_6.687	An16g03340
evm.model.unitig_6.688	An16g03330
evm.model.unitig_6.689	An16g03320
evm.model.unitig_6.69	An10g00970
evm.model.unitig_6.690	An16g03310
evm.model.unitig_6.691	An16g03300
evm.model.unitig_6.692	An16g03290

Gene ID of H915-1	Gene ID of 513.88
evm.model.unitig_6.694	An16g03190
evm.model.unitig_6.695	An16g03140
evm.model.unitig_6.696	An16g03130
evm.model.unitig_6.697	An16g03120
evm.model.unitig_6.698	An16g03110
evm.model.unitig_6.699	An16g03100
evm.model.unitig_6.7	An10g00100
evm.model.unitig_6.70	An10g01000
evm.model.unitig_6.700	An16g03090
evm.model.unitig_6.701	An16g03080
evm.model.unitig_6.702	An16g03070
evm.model.unitig_6.703	An16g03060
evm.model.unitig_6.704	An16g03050
evm.model.unitig_6.705	An16g03040
evm.model.unitig_6.706	An16g03030
evm.model.unitig_6.707	An16g03020
evm.model.unitig_6.708	An16g03010
evm.model.unitig_6.709	An16g03000
evm.model.unitig_6.71	An10g01010
evm.model.unitig_6.710	An16g02990
evm.model.unitig_6.711	An16g02980
evm.model.unitig_6.712	An16g02970
evm.model.unitig_6.713	An16g02960
evm.model.unitig_6.714	An16g02950
evm.model.unitig_6.715	An16g02940
evm.model.unitig_6.716	An16g02930
evm.model.unitig_6.717	An16g02920
evm.model.unitig_6.718	An16g02910
evm.model.unitig_6.719	An16g02890
evm.model.unitig_6.72	An10g01020
evm.model.unitig_6.720	An16g02870
evm.model.unitig_6.721	An16g02850
evm.model.unitig_6.722	An16g02820
evm.model.unitig_6.723	An16g02770
evm.model.unitig_6.724	An16g02760
evm.model.unitig_6.725	An10g00120
evm.model.unitig_6.726	An16g02740
evm.model.unitig_6.727	An16g02740
evm.model.unitig_6.728	An07g03950
evm.model.unitig_6.729	An16g02730
evm.model.unitig_6.73	An10g01040
evm.model.unitig_6.730	An16g02700
evm.model.unitig_6.731	An16g02680

Gene ID of H915-1	Gene ID of 513.88
evm.model.unitig_6.732	An16g02670
evm.model.unitig_6.733	An16g02660
evm.model.unitig_6.734	An16g02650
evm.model.unitig_6.736	An16g02610
evm.model.unitig_6.737	An16g02595
evm.model.unitig_6.738	An16g02590
evm.model.unitig_6.739	An16g02580
evm.model.unitig_6.74	An14g03640
evm.model.unitig_6.740	An16g02570
evm.model.unitig_6.741	An16g02560
evm.model.unitig_6.742	An16g02550
evm.model.unitig_6.743	An16g02520
evm.model.unitig_6.744	An16g02510
evm.model.unitig_6.745	An16g02500
evm.model.unitig_6.746	An16g02490
evm.model.unitig_6.747	An16g02480
evm.model.unitig_6.748	An16g02470
evm.model.unitig_6.749	An16g02460
evm.model.unitig_6.75	An07g01940
evm.model.unitig_6.751	An16g02450
evm.model.unitig_6.752	An16g02440
evm.model.unitig_6.753	An16g02430
evm.model.unitig_6.754	An16g02420
evm.model.unitig_6.755	An16g02400
evm.model.unitig_6.756	An16g02390
evm.model.unitig_6.757	An02g08660
evm.model.unitig_6.758	An16g02370
evm.model.unitig_6.759	An16g02360
evm.model.unitig_6.760	An16g02350
evm.model.unitig_6.761	An16g02330
evm.model.unitig_6.762	An16g02320
evm.model.unitig_6.763	An16g02250
evm.model.unitig_6.764	An16g02240
evm.model.unitig_6.765	An16g02230
evm.model.unitig_6.766	An18g03080
evm.model.unitig_6.767	An16g02210
evm.model.unitig_6.768	An16g02200
evm.model.unitig_6.769	An16g02190
evm.model.unitig_6.77	An11g08220
evm.model.unitig_6.770	An16g02180
evm.model.unitig_6.771	An16g02170
evm.model.unitig_6.772	An16g02160
evm.model.unitig_6.773	An16g02150

Gene ID of H915-1	Gene ID of 513.88
evm.model.unitig_6.774	An16g02140
evm.model.unitig_6.775	An16g02130
evm.model.unitig_6.776	An16g02120
evm.model.unitig_6.777	An16g02100
evm.model.unitig_6.778	An16g02090
evm.model.unitig_6.779	An16g02080
evm.model.unitig_6.78	An07g08940
evm.model.unitig_6.780	An16g02070
evm.model.unitig_6.781	An16g02060
evm.model.unitig_6.782	An16g02040
evm.model.unitig_6.783	An16g02020
evm.model.unitig_6.784	An16g02010
evm.model.unitig_6.785	An16g02000
evm.model.unitig_6.786	An16g01990
evm.model.unitig_6.787	An16g01980
evm.model.unitig_6.788	An16g01950
evm.model.unitig_6.789	An16g01940
evm.model.unitig_6.790	An16g01930
evm.model.unitig_6.791	An16g01920
evm.model.unitig_6.792	An16g01900
evm.model.unitig_6.793	An16g01890
evm.model.unitig_6.794	An16g01880
evm.model.unitig_6.795	An16g01870
evm.model.unitig_6.796	An16g01860
evm.model.unitig_6.797	An16g01850
evm.model.unitig_6.798	An16g01840
evm.model.unitig_6.799	An16g01830
evm.model.unitig_6.8	An10g00120
evm.model.unitig_6.80	An12g05240
evm.model.unitig_6.800	An16g01820
evm.model.unitig_6.801	An16g01810
evm.model.unitig_6.802	An16g01800
evm.model.unitig_6.803	An16g01780
evm.model.unitig_6.804	An16g01770
evm.model.unitig_6.805	An16g01760
evm.model.unitig_6.806	An16g01750
evm.model.unitig_6.807	An16g01740
evm.model.unitig_6.808	An16g01730
evm.model.unitig_6.809	An16g01720
evm.model.unitig_6.81	An12g05610
evm.model.unitig_6.810	An16g01710
evm.model.unitig_6.811	An07g01290
evm.model.unitig_6.812	An16g01690

Gene ID of H915-1	Gene ID of 513.88
evm.model.unitig_6.813	An16g01680
evm.model.unitig_6.814	An16g01670
evm.model.unitig_6.815	An16g01660
evm.model.unitig_6.816	An16g01650
evm.model.unitig_6.817	An16g01640
evm.model.unitig_6.818	An16g01630
evm.model.unitig_6.819	An16g01620
evm.model.unitig_6.82	An16g05190
evm.model.unitig_6.820	An16g01600
evm.model.unitig_6.821	An16g01590
evm.model.unitig_6.822	An16g01580
evm.model.unitig_6.823	An16g01570
evm.model.unitig_6.824	An16g01560
evm.model.unitig_6.825	An16g01550
evm.model.unitig_6.826	An16g01540
evm.model.unitig_6.827	An16g01530
evm.model.unitig_6.828	An16g01520
evm.model.unitig_6.829	An16g01510
evm.model.unitig_6.83	An16g06170
evm.model.unitig_6.830	An16g01500
evm.model.unitig_6.831	An16g01490
evm.model.unitig_6.832	An16g01480
evm.model.unitig_6.833	An16g01470
evm.model.unitig_6.834	An16g01460
evm.model.unitig_6.835	An16g01450
evm.model.unitig_6.836	An16g01440
evm.model.unitig_6.837	An04g03850
evm.model.unitig_6.838	An16g01420
evm.model.unitig_6.839	An16g01410
evm.model.unitig_6.84	An12g10240
evm.model.unitig_6.840	An16g08980
evm.model.unitig_6.841	An16g01390
evm.model.unitig_6.842	An16g01380
evm.model.unitig_6.843	An16g01370
evm.model.unitig_6.844	An16g01360
evm.model.unitig_6.845	An16g01320
evm.model.unitig_6.846	An16g01290
evm.model.unitig_6.847	An16g01280
evm.model.unitig_6.849	An16g01250
evm.model.unitig_6.85	An12g10230
evm.model.unitig_6.851	An16g01230
evm.model.unitig_6.853	An16g01200
evm.model.unitig_6.854	An16g01190

Gene ID of H915-1	Gene ID of 513.88
evm.model.unitig_6.855	An16g01170
evm.model.unitig_6.856	An16g01150
evm.model.unitig_6.857	An16g01110
evm.model.unitig_6.858	An16g01040
evm.model.unitig_6.859	An16g01030
evm.model.unitig_6.86	An03g00940
evm.model.unitig_6.860	An16g01010
evm.model.unitig_6.861	An16g01000
evm.model.unitig_6.862	An16g00990
evm.model.unitig_6.863	An02g08350
evm.model.unitig_6.864	An16g00960
evm.model.unitig_6.865	An16g00930
evm.model.unitig_6.866	An16g00920
evm.model.unitig_6.867	An16g00910
evm.model.unitig_6.868	An16g00900
evm.model.unitig_6.869	An16g00890
evm.model.unitig_6.87	An04g02610
evm.model.unitig_6.870	An16g00850
evm.model.unitig_6.871	An16g00830
evm.model.unitig_6.872	An16g00810
evm.model.unitig_6.873	An16g00800
evm.model.unitig_6.874	An16g00780
evm.model.unitig_6.875	An16g00780
evm.model.unitig_6.876	An16g00090
evm.model.unitig_6.877	An16g00760
evm.model.unitig_6.878	An16g00750
evm.model.unitig_6.879	An16g00740
evm.model.unitig_6.88	An09g00880
evm.model.unitig_6.881	An16g00710
evm.model.unitig_6.882	An16g00700
evm.model.unitig_6.883	An16g00660
evm.model.unitig_6.884	An02g10480
evm.model.unitig_6.885	An16g00600
evm.model.unitig_6.886	An16g00550
evm.model.unitig_6.887	An16g00540
evm.model.unitig_6.888	An16g00530
evm.model.unitig_6.889	An16g00520
evm.model.unitig_6.890	An16g00500
evm.model.unitig_6.891	An16g00460
evm.model.unitig_6.892	An16g00450
evm.model.unitig_6.893	An16g00440
evm.model.unitig_6.894	An16g00430
evm.model.unitig_6.895	An16g00420

Gene ID of H915-1	Gene ID of 513.88
evm.model.unitig_6.896	An16g00410
evm.model.unitig_6.897	An16g00400
evm.model.unitig_6.898	An16g00370
evm.model.unitig_6.899	An16g00350
evm.model.unitig_6.9	An10g00140
evm.model.unitig_6.90	An13g02680
evm.model.unitig_6.900	An16g00320
evm.model.unitig_6.901	An16g00290
evm.model.unitig_6.902	An16g00260
evm.model.unitig_6.903	An16g00250
evm.model.unitig_6.904	An16g00240
evm.model.unitig_6.905	An16g00230
evm.model.unitig_6.906	An16g00210
evm.model.unitig_6.907	An16g00200
evm.model.unitig_6.908	-
evm.model.unitig_6.909	An16g00180
evm.model.unitig_6.910	-
evm.model.unitig_6.911	An16g00090
evm.model.unitig_6.913	An16g00070
evm.model.unitig_6.914	An16g00040
evm.model.unitig_6.915	An16g00030
evm.model.unitig_6.916	An16g00020
evm.model.unitig_6.917	An16g00010
evm.model.unitig_6.918	An09g03800
evm.model.unitig_6.919	An03g01000
evm.model.unitig_6.92	An11g04700
evm.model.unitig_6.923	An13g02410
evm.model.unitig_6.93	An01g13260
evm.model.unitig_6.94	An16g04830
evm.model.unitig_6.97	An17g00010
